# Lewis-Acid-Catalyzed (3+2) Annulation of 2-Indolylmethanols with Propargylic Alcohols to Access Cyclopenta[*b*]indoles

**DOI:** 10.3390/molecules29061251

**Published:** 2024-03-12

**Authors:** Teng-Fei Wu, Zhao-Jie Fu, Yi-Rui Zhang, Zong-Wang Qiu, Bao Qiong Li, Shao-Shuai Chen, Han-Peng Pan, Ai-Jun Ma, Xiang-Zhi Zhang

**Affiliations:** Guangdong Provincial Key Laboratory of Large Animal Models for Biomedicine, School of Pharmacy and Food Engineering, Wuyi University, Jiangmen 529020, China; wtf008@sina.cn (T.-F.W.); fzj18316065165@126.com (Z.-J.F.); zyr348405719@163.com (Y.-R.Z.); qiuzongwang@126.com (Z.-W.Q.); chenshaoshuai1118@126.com (S.-S.C.); panhanp@126.com (H.-P.P.); maaijun@wyu.edu.cn (A.-J.M.)

**Keywords:** (3+2) annulation, cyclopenta[*b*]indoles, propargylic alcohols, 2-indolylmethanols

## Abstract

Herein, a Sc(OTf)_3_-catalyzed (3+2) annulation of 2-indolylmethanols with propargylic alcohols is reported. The reaction proceeds via a Friedel–Crafts-type allenylation/5-exo-annulation cascade. In the reaction, 2-indolylmethanol is used as a three-carbon synthon, and propargyl alcohol is used as a two-carbon synthon. This method provides a direct and high-yield pathway for synthetically useful cyclopenta[*b*]indoles. In general, the method features easily accessible substrates with broad scope and generality, the formation of multiple bonds with high efficiency, and easy scale-up.

## 1. Introduction

**Figure 1:**
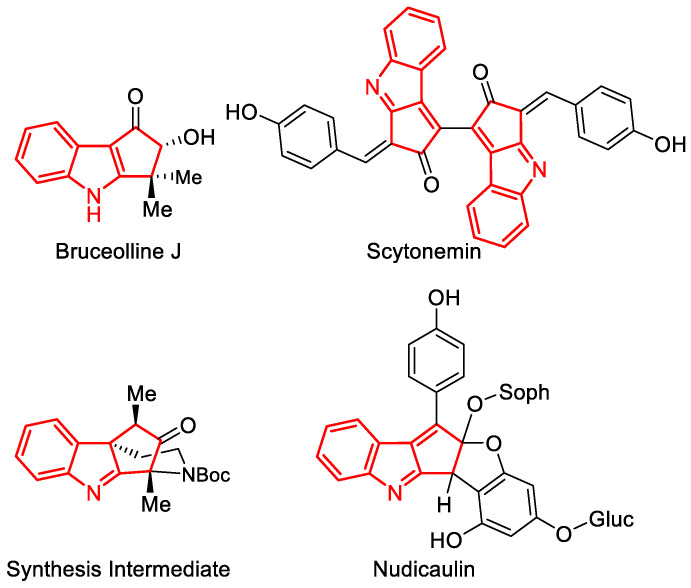
Representative natural product molecules.

Cyclopenta[*b*]indoles, as privileged heterocyclic skeletons, are present in numerous natural alkaloids and pharmaceuticals possessing a wide range of biological activities [1,2,3,4,5,6,7,8]. Accordingly, bruceollines are traditionally used to treat parasitic diseases and malaria [5,6]. Scytonemin is a small-molecule inhibitor of polo-like kinase and has the potential to inhibit other cell cycle regulators (Figure 1) [8]. Additionally, this type of fused tricyclic structure has been used as an intermediate in pharmaceutical chemistry and in the total synthesis of natural products, such as arborisidine and 19-epi-arborisidine [9]. Owing to their important biological properties, various synthetic protocols have been developed for preparing cyclopenta[*b*]indoles. Among these protocols, the (3+2) annulation of 2-indolylmethanols and olefins is an atom-economic strategy for the synthesis of these fused indole derivatives [10,11,12,13,14,15,16,17,18]. In recent years, 2-indolylmethanols have become intriguing reactants because of their unique properties of electrophilicity and nucleophilicity at the C3 position [19,20,21,22,23,24,25,26,27,28]. Despite these impressive achievements, a more efficient and diversity-oriented approach for cyclopenta[*b*]indoles is still required.

**Scheme 1:**
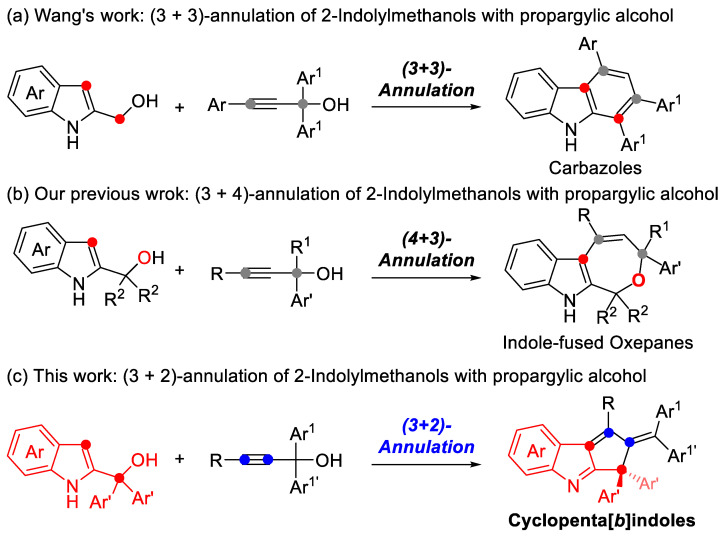
Annulation of 2-indolylmethanols with propargylic alcohols.

Propargylic alcohols have shown promise as feedstocks for the synthesis of carbo- and heterocyclic compounds. Recently, a number of studies have specifically focused on Lewis- or Brønsted-acid-catalyzed tandem annulations of propargylic alcohols to construct diversely structured indole derivatives [29,30,31,32,33,34,35]. However, there have been limited studies on the annulations of propargylic alcohols with 2-indolylmethanols. In 2014, Wang [36] reported a Lewis-acid-catalyzed (3+3) annulation of propargylic alcohols and 2-indolylmethanols, providing an effective approach to obtain polysubstituted carbazole derivatives (Scheme 1a). Recently, our group developed a Brønsted-acid-catalyzed (3+4) annulation of 2-indolylmethanols and propargylic alcohols, providing a convenient method to obtain structurally diverse indole-fused oxepines (Scheme 1b) [37]. However, to the best of our knowledge, no method has been reported for the synthesis of 1,2,3-trisubstituted cyclopenta[*b*]indoles by formal (3+2) annulation between 2-indolylmethanols and propargylic alcohols. With our continuing research interest in propargylic alcohols [38,39,40,41] and the importance of cyclopenta[*b*]indoles, herein, we describe a Sc(OTf)_3_-catalyzed (3+2) annulation of 2-indolylmethanols with propargylic alcohols to synthesize cyclopenta[*b*]indoles (Scheme 1c).

## 2. Results

Initially, 2-indolylmethanol **1a** and propargylic alcohol **2a** were selected as the model substrates to explore the reaction conditions in 1,2-dichloroethane (DCE) at 100 °C (Table 1). First, a range of Lewis acids (entries 1–6) were tested. When Zn(OTf)_2_ was used as the catalyst, the desired cyclopenta[*b*]indole **3a** was isolated in a moderate yield (entry 1, 62%). The fused tricyclic skeleton of product **3a** was determined by X-ray crystallographic analysis (CCDC 2,313,081 (**3a**) contains the supplementary crystallographic data for this paper; for details, see the Supporting Information). Other common triflates, such as Bi(OTf)_3_, Yb(OTf)_3_, Cu(OTf)_2_, and AgNTf_2_, all promoted the reaction and gave the desired cyclopenta[*b*]indole product in moderate yields (entries 2–5, 13–70%). Sc(OTf)_3_ was found to be the best catalyst, and we then further evaluated the effect of the solvent (entries 6–17). Apart from dimethylformamide (DMF) and dimethyl sulfoxide (DMSO) (entries 14 and 15), many common solvents worked well, providing **3a** in moderate yields ranging from 41% to 78% (entries 8–16). Pleasingly, toluene improved the efficiency of the reaction (entry 17, 30 min, 80% yield). Increasing the equivalent amount of propargylic alcohol **2a** had a positive effect on the reaction yield (entry 18, 87% yield). Decreasing the equivalent amount of the catalyst had a negative effect on the reaction yield (entry 19, 44% yield).

**Scheme 2:**
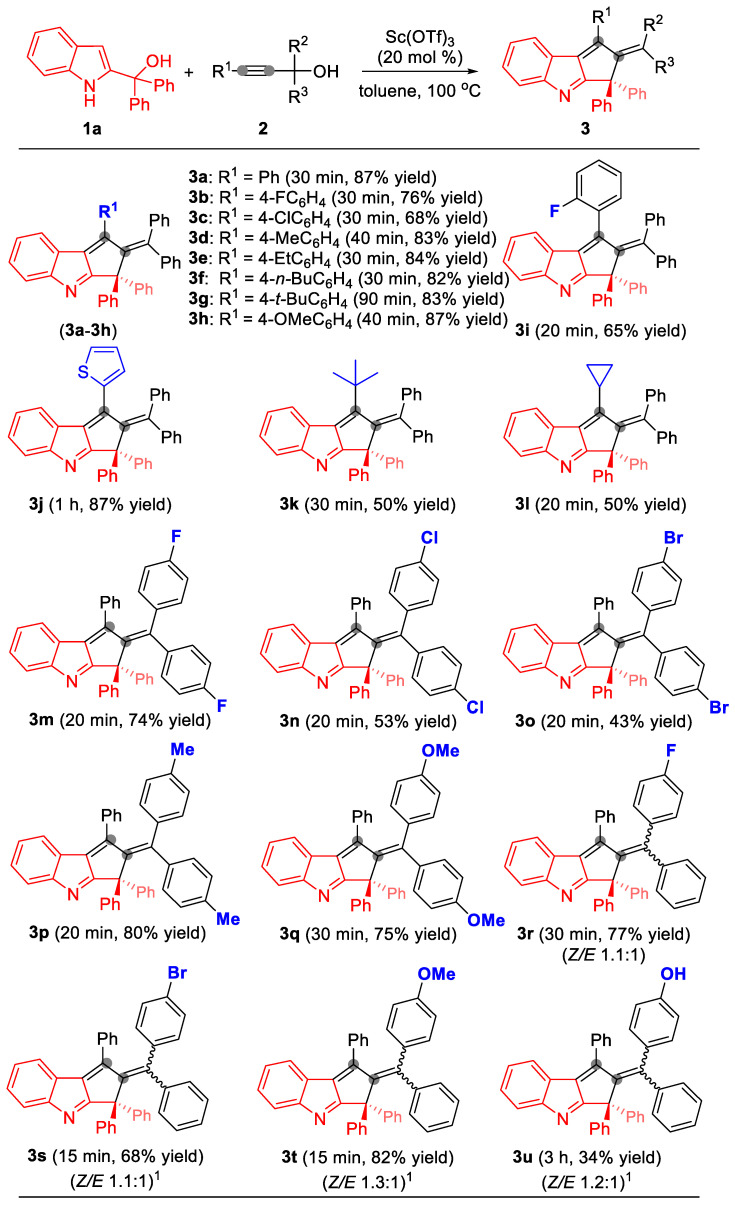
Scope of propargylic alcohols. Performed at 0.2 mmol scale. ^1^ Ratio of isomers determined by crude ^1^H NMR.

With the optimized conditions in hand, we studied the substrate scope of propargylic alcohols **2**. Various propargylic alcohols bearing different aromatic substituents gave the corresponding cyclopenta[*b*]indoles in good yields (Scheme 2). Both electron-withdrawing (Cl and F) and electron-donating (Me, Et, *n*-Bu, *t*-Bu, and MeO) groups were compatible with the reaction, affording the desired products **3a**–**3h** in yields ranging from 68% to 87%. Adjacent steric hindrance is unfavorable for this reaction (**2i**, 2-FC_6_H_4_, 65% yield). Propargylic alcohols with a heteroaryl group (2-thienyl) were also compatible with the reaction, yielding product **3g** in an 83% yield. Alkyl-substituted propargyl alcohols **2k** and **2l** (R^1^= *t*-Bu, cyclopropyl) gave the desired products **3k** and **3l** in a 50% yield. Moreover, propargylic alcohols **2m**–**2q**, which contained the same aromatic groups (R^2^ = R^3^), were compatible with the reaction, yielding the corresponding products **3m**–**3q** in moderate-to-good yields (43–80% yield). Propargylic alcohols with different aromatic groups (**2r**–**2u**, R^2^ ≠ R^3^) provided the *E* and *Z* isomers of products **3r**–**3u** (*Z:E* = 1.1:1 to 1.3:1). Notably, when R^2^ or R^3^ was an alkyl group, no products were detected (for details of the examples, see the Supporting Information).

**Scheme 3:**
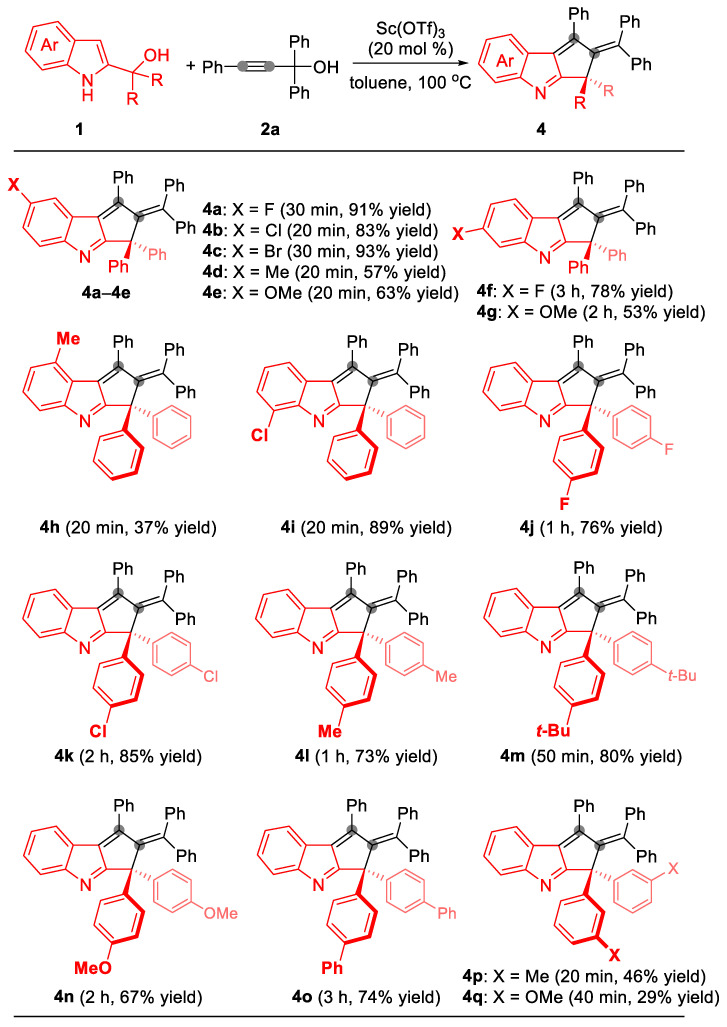
Scope of 2-indolylmethols. Performed at 0.2 mmol scale.

Next, we assessed the substrate scope of 2-indolylmethanols **1** in the (3+2) annulation catalyzed by Sc(OTf)_3_ (Scheme 3). A series of indolylmethanols (**1b**–**1j**) containing electronically diverse groups at different positions of the indole ring served as appropriate reaction partners in the tandem reaction, giving products **4a**–**4i** in moderate-to-good yields (37–93% yield). Moreover, indolylmethanols **1k**–**1r**, bearing electron-donating or electron-withdrawing Ar groups with *para*-or *meta*-substitution patterns, acted as suitable substrates, undergoing (3+2) annulation with propargylic alcohol **2a** to produce hydrobenzo[*b*]carbazoles **4j–4q** in moderate-to-high yields. Notably, when R was an alkyl group, no product was obtained.

**Scheme 4:**
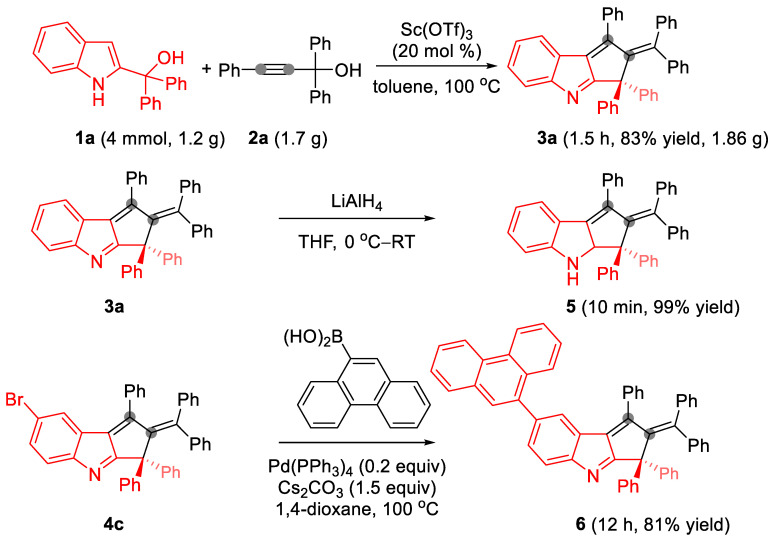
Gram-scale reaction and transformations.

To explore the utility of this methodology, a gram-scale reaction of **3a** was carried out, affording the product **3a** in an 83% yield (Scheme 4). Treatment of **3a** with LiAlH_4_ produced compound **5** in a 99% yield. Compound **6** was obtained in an 81% yield through the Suzuki coupling reaction of **4c** and 9-phenanthrenylboronic acid.

**Scheme 5:**
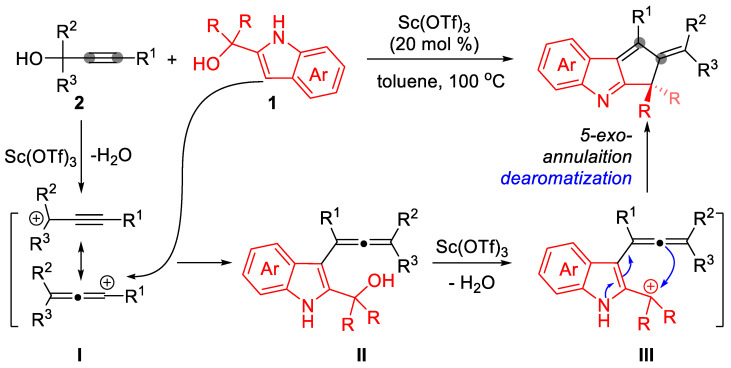
Plausible mechanism.

A plausible mechanism for the reaction is proposed in Scheme 5. First, propargylic alcohol **2** is converted to allene carbocation **I** under Sc(OTf)_3_ catalysis. This carbocation then undergoes a Friedel–Crafts-type reaction with indolylmethanol **1** to give the allene intermediate **II**. This intermediate is transformed to benzylic carbocation **III**. Intermediate **III** undergoes 5-*exo*-annulation to give the final cyclopenta[*b*]indole product.

In conclusion, we have developed a Sc(OTf)_3_-catalyzed (3+2) annulation of 2-indolylmethanols with propargylic alcohols for the synthesis of cyclopenta[*b*]indoles. A Friedel–Crafts-type allenylation/5-*exo*-annulation cascade is involved in the reaction. This method provides a direct and high-yield pathway to synthesize useful cyclopenta[*b*]indoles. In general, this method features easily accessible substrates with a broad scope and generality, the formation of multiple bonds with high efficiency, and easy scale-up. This work not only confirms that indolylmethanol is a good three-carbon synthon, but it also enriches the chemistry of propargyl alcohol as a two-carbon synthon.

## 3. Materials and Methods

Unless otherwise noted, all reagents were purchased as commercial grade (Bide, Energy Chemica, Adamas in China) and used without further purification. All products were purified by flash column chromatography using 200–300 mesh silica gel as a stationary phase. NMR spectra were recorded on a Bruker AVANCE III HD 500 device (500 MHz), using CDCl_3_. Chemical shifts were denoted in ppm (δ) and calibrated by using residual undeuterated solvent CHCl_3_ (7.27 ppm for ^1^H NMR, 77.00 ppm for 13C {1H} NMR) with Me_4_Si (0.00 ppm) as an internal reference. High-resolution mass spectral analysis (HRMS) data were obtained using an Orbitrap instrument equipped with an ESI source. The 2-indolylmethanols and propargylic alcohols **2** were prepared according to the literature-reported procedures [42,43].

### 3.1. General Procedure for the Synthesis of ***3a–4q***

Sc(OTf)_3_ (0.04 mmol, 20 mol%) was added to a solution of 2-indolylmethanols **1** (0.2 mmol, 1.0 equiv) and propargylic alcohol **2** (0.3 mmol, 1.5 equiv) in toluene (2.0 mL) in a glass pressure tube (10 mL). The resulting mixture was stirred vigorously at 100 °C. The solvent was concentrated under reduced pressure, and the resulting residue was purified by flash column chromatography on silica gel eluting with petroleum ether/ethyl acetate, giving the corresponding products **3a**–**4q**. 

### 3.2. Spectroscopic Data for the Products ***3a–4q***

*2-(Diphenylmethylene)-1,3,3-triphenyl-2,3-dihydrocyclopenta[b]indole* (**3a**): (eluent: petroleum ether/ethyl acetate 10:1 to 3:1, 30 min, red foam solid, 95.0 mg, 87% yield, mp 270–274 °C). ^1^H NMR (500 MHz, CDCl_3_) δ 7.64 (d, *J* = 7.5 Hz, 1H), 7.60–7.54 (m, 2H), 7.49 (d, *J* = 7.8 Hz, 1H), 7.42–7.34 (m, 4H), 7.29–7.23 (m, 1H), 7.20–7.04 (m, 9H), 7.01 (t, *J* = 7.3 Hz, 1H), 6.95 (t, *J* = 7.5 Hz, 1H), 6.88–6.80 (m, 5H), 6.77 (t, *J* = 7.8 Hz, 2H), 6.57 (d, *J* = 7.2 Hz, 2H). ^13^C {1H} NMR (126 MHz, CDCl_3_) δ 188.4, 162.9, 156.9, 152.2, 148.0, 142.4, 142.02, 141.97, 141.9, 141.8, 135.3, 130.8, 129.7, 129.6, 129.2, 129.0, 128.6, 127.9, 127.6, 127.4, 127.2, 126.7, 126.2, 125.2, 123.8, 123.0, 120.8, 62.3. IR: *ῡ* = 2921, 2853, 1640, 1400, 1190, 1134, 1077, 756, 686, 626, 467 cm^−1^. HRMS (ESI) *m*/*z*: [M + H]^+^ calcd. for C_42_H_30_N: 548.2373; found: 548.2371.

*2-(Diphenylmethylene)-1-(4-fluorophenyl)-3,3-diphenyl-2,3-dihydrocyclopenta[b]indole* (**3b**): (eluent: petroleum ether/ethyl acetate 10:1 to 3:1, 30 min, red foam solid, 85.9 mg, 76% yield). ^1^H NMR (500 MHz, CDCl_3_) δ 7.59 (d, *J* = 7.6 Hz, 1H), 7.57–7.50 (m, 2H), 7.49 (d, *J* = 7.8 Hz, 1H), 7.41–7.33 (m, 4H), 7.27 (td, *J* = 7.7, 1.0 Hz, 1H), 7.16–7.05 (m, 6H), 7.01 (t, *J* = 7.5 Hz, 1H), 6.95 (t, *J* = 7.4 Hz, 1H), 6.92–6.81 (m, 7H), 6.77 (t, *J* = 7.7 Hz, 2H), 6.57 (d, *J* = 8.0 Hz, 2H). ^13^C NMR (126 MHz, CDCl_3_) δ 188.3, 188.2, 163.0, 161.8 (d, *J_C-F_* = 248.1 Hz), 161.7 (d, *J_C-F_* = 247.3 Hz), 156.5, 156.2, 152.6, 152.5, 146.6, 146.5, 142.1, 142.11, 142.09, 141.8, 141.7, 138.5, 138.0, 135.2, 132.5, 132.4, 130.94, 130.87, 130.7, 129.80, 129.77, 129.5, 129.2, 128.9, 128.7, 128.6, 128.0, 127.9, 127.7, 127.6, 127.5, 127.4, 127.3, 126.9, 126.4, 126.2, 125.2, 125.1, 123.8, 123.0, 120.9, 114.3, 114.19, 114.15, 114.0, 62.3. ^19^F NMR (471 MHz, CDCl_3_) δ –111.3. IR: *ῡ* = 2920, 2851, 1646, 1528, 1401, 1188, 1135, 1075, 757, 672, 469 cm^−1^. HRMS (ESI) *m*/*z*: [M + H]^+^ calcd. for C_42_H_29_FN: 566.2279; found: 566.2273.

*1-(D-chlorophenyl)-2-(diphenylmethylene)-3,3-diphenyl-2,3-dihydrocyclopenta[b]indole* (**3c**): (eluent: petroleum ether/ethyl acetate 10:1 to 3:1, 30 min, red foam solid, 79.0 mg, 68% yield). ^1^H NMR (500 MHz, CDCl_3_) δ 7.64 (d, *J* = 7.4 Hz, 1H), 7.60–7.53 (m, 2H), 7.48 (d, *J* = 7.8 Hz, 1H), 7.42–7.34 (m, 4H), 7.30–7.22 (m, 1H), 7.21–7.05 (m, 9H), 7.03–6.99 (m, 1H), 6.98–6.92 (m, 1H), 6.88–6.80 (m, 5H), 6.77 (t, *J* = 7.8 Hz, 2H), 6.60–6.54 (m, 2H). ^13^C {1H} NMR (126 MHz, CDCl_3_) δ 188.3, 163.0, 155.0, 152.0, 148.0, 142.2, 142.1, 141.69, 141.65, 134.2, 133.8, 130.7, 130.2, 130.0, 129.5, 129.1, 128.1, 127.6, 127.51, 127.47, 127.2, 126.8, 126.3, 125.0, 123.9, 122.9, 121.0, 62.3. IR: *ῡ* = 2920, 2851, 1646, 1534, 1463, 1402, 1188, 1135, 1075, 758, 669, 474cm^−1^. HRMS (ESI) *m*/*z*: [M + H]^+^ calcd. for C_42_H_29_ClNO: 582.1983; found: 582.1978.

*2-(Diphenylmethylene)-3,3-diphenyl-1-(p-tolyl)-2,3-dihydrocyclopenta[b]indole* (**3d**): (eluent: petroleum ether/ethyl acetate 10:1 to 3:1, 40 min, red foam solid, 93.1 mg, 83% yield). ^1^H NMR (500 MHz, CDCl_3_) δ 7.67 (d, *J* = 7.5 Hz, 1H), 7.51–7.42 (m, 3H), 7.42–7.33 (m, 4H), 7.24 (td, *J* = 7.7, 1.3 Hz, 1H), 7.16–7.03 (m, 6H), 7.00 (t, *J* = 7.3 Hz, 1H), 6.97–6.89 (m, 3H), 6.86–6.79 (m, *J* = 3.0 Hz, 5H), 6.76 (t, *J* = 7.7 Hz, 2H), 6.57 (dd, *J* = 8.0, 1.4 Hz, 2H), 2.23 (s, 3H). ^13^C {1H} NMR (126 MHz, CDCl_3_) δ 188.3, 162.9, 157.1, 152.2, 147.9, 142.4, 142.1, 141.9, 141.4, 138.6, 132.3, 130.8, 129.53, 129.45, 129.2, 129.0, 128.5, 127.5, 127.2, 127.1, 127.0, 126.6, 126.1, 125.3, 123.6, 123.0, 120.7, 62.3, 21.3. IR: *ῡ* = 2922, 2852, 1645, 1530,1401, 1191, 1134, 1075, 758, 672, 573, 473 cm^−1^. HRMS (ESI) *m*/*z*: [M + H]^+^ calcd. for C_43_H_32_N: 562.2529; found: 562.2524.

*2-(Diphenylmethylene)-1-(4-ethylphenyl)-3,3-diphenyl-2,3-dihydr*o*cyclopenta[b]indole* (**3e**): (eluent: petroleum ether/ethyl acetate 10:1 to 3:1, 30 min, red foam solid, 96.6 mg, 84% yield). ^1^H NMR (500 MHz, CDCl_3_) δ 7.68 (d, *J* = 7.3 Hz, 1H), 7.52–7.44 (m, 3H), 7.42–7.32 (m, 4H), 7.25 (td, *J* = 7.7, 1.1 Hz, 1H), 7.17–7.03 (m, 6H), 7.01 (td, *J* = 7.6, 0.9 Hz, 1H), 6.99–6.90 (m, 3H), 6.86–6.79 (m, 5H), 6.76 (t, *J* = 7.8 Hz, 2H), 6.57 (dd, *J* = 8.2, 1.1 Hz, 2H), 2.53 (q, *J* = 7.6 Hz, 2H), 1.15 (t, *J* = 7.6 Hz, 3H). ^13^C {1H} NMR (126 MHz, CDCl_3_) δ 188.4, 162.9, 157.1, 152.3, 147.9, 145.0, 142.3, 142.1, 141.9, 141.5, 132.5, 130.8, 129.6, 129.5, 129.21, 129.15, 127.6, 127.4, 127.21, 127.15, 126.6, 126.1, 125.3, 123.7, 123.0, 120.8, 62.3, 28.8, 15.5. IR: *ῡ* = 2921, 2852, 1647, 1529, 1400, 1191, 1133, 1075, 759, 671, 521, 471 cm^−1^. HRMS (ESI) *m*/*z*: [M + H]^+^ calcd. for C_44_H_34_N: 576.2686; found: 576.2685.

*1-(4-Butylphenyl)-2-(diphenylmethylene)-3,3-diphenyl-2,3-dihydrocyclopenta[b]indole* (**3f**): (eluent: petroleum ether/ethyl acetate 10:1 to 3:1, 30 min, red foam solid, 98.9 mg, 82% yield). ^1^H NMR (500 MHz, CDCl_3_) δ 7.68 (d, *J* = 7.4 Hz, 1H), 7.53–7.44 (m, 3H), 7.44–7.32 (m, 4H), 7.24 (td, *J* = 7.7, 1.0 Hz, 1H), 7.17–7.03 (m, 6H), 7.00 (td, *J* = 7.6, 1.1 Hz, 1H), 6.98–6.90 (m, 3H), 6.88–6.78 (m, 5H), 6.76 (t, *J* = 7.8 Hz, 2H), 6.60–6.54 (m, 2H), 2.50 (t, *J* = 7.5 Hz, 2H), 1.58–1.44 (m, 2H), 1.33–1.22 (m, 2H), 0.94 (t, *J* = 7.3 Hz, 3H). ^13^C {1H} NMR (126 MHz, CDCl_3_) δ 188.4, 162.9, 157.2, 152.3, 147.8, 143.6, 142.4, 142.1, 141.9, 141.4, 132.5, 130.8, 129.5, 129.4, 129.2, 129.0, 128.0, 127.5, 127.2, 127.1, 126.6, 126.1, 125.3, 123.6, 123.0, 120.8, 62.3, 35.4, 33.3, 22.0, 13.9. IR: *ῡ* = 2921, 2852, 1646, 1530, 1465, 1400, 1188, 1135, 1075, 756, 672, 470 cm^−1^. HRMS (ESI) *m*/*z*: [M + H]^+^ calcd. for C_46_H_38_N: 604.2999; found: 604.2996.

*1-(4-(tert-Butyl)phenyl)-2-(diphenylmethylene)-3,3-diphenyl-2,3-dihy*dro*cyclopenta[b]indole* (**3g**): (eluent: petroleum ether/ethyl acetate 10:1 to 3:1, 2 h, red foam solid, 100.0 mg, 83% yield). ^1^H NMR (500 MHz, CDCl_3_) δ 7.71 (d, *J* = 7.3 Hz, 1H), 7.53–7.45 (m, 3H), 7.41–7.34 (m, 4H), 7.32–7.22 (m, 1H), 7.18–7.04 (m, 8H), 7.03 (td, *J* = 7.5, 0.9 Hz, 1H), 6.98–6.91 (m, 1H), 6.84–6.73 (m, 7H), 6.57 (dd, *J* = 8.3, 1.3 Hz, 2H), 1.25 (s, 9H). ^13^C {1H} NMR (126 MHz, CDCl_3_) δ 188.4, 162.9, 157.0, 152.4, 151.6, 147.8, 142.3, 142.1, 141.9, 141.4, 132.1, 130.8, 129.6, 129.5, 129.2, 129.0, 127.6, 127.2, 126.6, 126.1, 125.4, 124.8, 123.7, 123.1, 120.8, 62.3, 34.6, 31.0. IR: *ῡ* = 2921, 2852, 1647, 1399, 1188, 1135, 1075, 757, 672, 576, 471 cm^−1^. HRMS (ESI) *m*/*z*: [M + H]^+^ calcd. For C_46_H_38_N: 604.2999; found: 604.2998.

*2-(Diphenylmethylene)-1-(4-methoxyphenyl)-3,3-diphenyl-2,3-dihydrocyclopenta[b]indole* (**3h**): (eluent: petroleum ether/ethyl acetate 10:1 to 3:1, 40 min, red foam solid, 100.4 mg, 87% yield). ^1^H NMR (500 MHz, CDCl_3_) δ 7.70 (d, *J* = 7.5 Hz, 1H), 7.56–7.51 (m, 2H), 7.49 (d, *J* = 7.8 Hz, 1H), 7.39–7.34 (m, 4H), 7.30–7.23 (m, 1H), 7.15–7.04 (m, 6H), 7.03 (td, *J* = 7.6, 0.9 Hz, 1H), 6.99–6.91 (m, 1H), 6.90–6.79 (m, 5H), 6.77 (t, *J* = 7.8 Hz, 2H), 6.72–6.66 (m, 2H), 6.57 (dd, *J* = 8.1, 1.2 Hz, 2H), 3.76 (s, 3H). ^13^C {1H} NMR (126 MHz, CDCl_3_) δ 188.2, 162.9, 159.8, 156.8, 152.3, 147.9, 142.5, 142.2, 142.0, 140.9, 130.9, 130.8, 129.6, 129.4, 129.3, 127.7, 127.6, 127.3, 127.20, 127.17, 126.7, 126.1, 125.4, 123.6, 122.9, 120.8, 113.5, 62.4, 55.3. IR: *ῡ* = 3117, 2920, 2852, 1647, 1529, 1401, 1188, 1135, 1074, 757, 670, 472 cm^−1^. HRMS (ESI) *m*/*z*: [M + H]^+^ calcd. for C_43_H_32_NO: 578.2478; found: 578.2473.

*2-(Diphenylmethylene)-1-(2-fluorophenyl)-3,3-diphenyl-2,3-dihydrocyclopenta[b]indole* (**3i**): (eluent: petroleum ether/ethyl acetate 10:1 to 3:1, 20 min, red foam solid, 73.4 mg, 65% yield). ^1^H NMR (500 MHz, CDCl_3_) δ 7.56 (td, *J* = 7.4, 1.8 Hz, 2H), 7.50–7.30 (m, 5H), 7.30–7.25 (m, 1H), 7.20–7.07 (m, 7H), 7.07–6.97 (m, 2H), 6.97–6.81 (m, 6H), 6.80–6.72 (m, 3H), 6.57 (d, *J* = 7.5 Hz, 2H). ^13^C {1H} NMR (126 MHz, CDCl_3_) δ 188.5, 163.0, 158.1 (d, *J_C-F_* = 249.8 Hz), 151.7, 149.9, 147.7, 143.2, 141.7, 141.6, 130.8, 130.7, 130.44, 130.4, 130.3, 129.9, 129.6, 128.9, 128.4, 127.60, 127.42, 127.2, 126.6, 126.2, 125.0, 123.9, 123.8, 123.63, 123.60, 123.4, 120.9, 115.9, 115.8, 62.2.^19^F NMR (471 MHz, CDCl_3_) δ -108.4. IR: *ῡ* = 2920, 2851, 1646, 1528, 1399, 1188, 1135, 1075, 756, 672, 471 cm^−1^ HRMS (ESI) *m*/*z*: [M + H]^+^ calcd. for C_42_H_29_FN 566.2279; found: 566.2276.

*2-(Diphenylmethylene)-3,3-diphenyl-1-(thiophen-2-yl)-2,3-dihydrocyclopenta[b]indole* (**3j**): (eluent: petroleum ether/ethyl acetate 10:1 to 3:1, 1 h, red foam solid, 96.2 mg, 87% yield). ^1^H NMR (500 MHz, CDCl_3_) δ 8.11 (d, *J* = 7.6 Hz, 1H), 7.50 (d, *J* = 7.8 Hz, 1H), 7.36 (d, *J* = 7.3 Hz, 4H), 7.34–7.24 (m, 3H), 7.14–7.02 (m, 7H), 6.99–6.90 (m, 6H), 6.81–6.73 (m, 3H), 6.60 (d, *J* = 8.0 Hz, 2H). ^13^C {1H} NMR (126 MHz, CDCl_3_) δ 188.3, 162.7, 152.5, 149.0, 147.6, 143.2, 142.2, 141.9, 139.8, 136.7, 131.8, 130.6, 129.7, 129.5, 128.6, 127.6, 127.4, 127.3, 127.1, 126.9, 126.2, 124.8, 123.8, 123.7, 120.8, 62.9. IR: *ῡ* = 2923, 2853, 1693, 1648, 1528, 1399, 1192, 1134, 1075, 757, 671, 474 cm^−1^. HRMS (ESI) *m*/*z*: [M + H]^+^ calcd. for C_40_H_28_NS: 554.1937; found: 554.1934.

*1-(tert-Butyl)-2-(diphenylmethylene)-3,3-diphenyl-2,3-dihydrocyclopenta[b]indole* (**3k**): (eluent: petroleum ether/ethyl acetate 10:1 to 3:1, 30 min, red foam solid, 52.6 mg, 50% yield). ^1^H NMR (500 MHz, CDCl_3_) δ 7.81 (d, *J* = 7.5 Hz, 1H), 7.53 (d, *J* = 7.7 Hz, 1H), 7.42–7.23 (m, 7H), 7.23–6.86 (m, 12H), 6.74 (s, 3H), 1.31 (s, 9H). ^13^C {1H} NMR (126 MHz, CDCl_3_) δ 188.5, 176.7, 162.3, 153.8, 146.7, 145.6, 143.0, 142.6, 142.2, 132.1, 130.1, 128.72, 128.70, 128.24, 128.16, 127.4, 126.9, 126.0, 125.6, 125.5, 123.4, 120.7, 65.7, 37.4, 31.0. IR: *ῡ* = 2921, 2852, 1647, 1529,1401, 1192, 1134, 1074, 758, 671, 566, 468 cm^−1^. HRMS (ESI) *m*/*z*: [M + H]^+^ calcd. for C_40_H_34_N: 528.2686; found: 528.2682.

*1-Cyclopropyl-2-(diphenylmethylene)-3,3-diphenyl-2,3-dihydrocyclopenta[b]indole* (**3l**): (eluent: petroleum ether/ethyl acetate 10:1 to 3:1, 20 min, red foam solid, 51.1 mg, 50% yield). ^1^H NMR (500 MHz, CDCl_3_) δ 7.61 (d, *J* = 7.5 Hz, 1H), 7.47 (d, *J* = 7.7 Hz, 1H), 7.34–7.16 (m, 10H), 7.15–6.99 (m, 7H), 6.98–6.88 (m, 1H), 6.76 (t, *J* = 7.8 Hz, 2H), 6.58–6.54 (m, 2H), 1.49–1.31 (m, 3H), 0.99–0.82 (m, 2H). ^13^C {1H} NMR (126 MHz, CDCl_3_) δ 188.4, 167.1, 162.3, 154.6, 145.8, 144.0, 142.0, 141.8, 138.7, 130.4, 129.6, 129.2, 128.6, 128.02, 128.00, 127.5, 127.2, 126.7, 126.0, 124.4, 123.8, 123.5, 120.7, 62.1, 16.1, 13.0. IR: *ῡ* =2921, 2852, 1646, 1529, 1402, 1191, 1134, 1075, 758, 671, 563, 471 cm^−1^. HRMS (ESI) *m*/*z*: [M + H]^+^ calcd. for C_39_H_30_N: 512.2373; found: 512.2368.

*2-(Bis(4-fluorophenyl)methylene)-1,3,3-triphenyl-2,3-dihydrocyclopenta[b]indole* (**3m**): (eluent: petroleum ether/ethyl acetate 10:1 to 3:1, 20 min, red foam solid, 86.2 mg, 74% yield). ^1^H NMR (500 MHz, CDCl_3_) δ 7.65 (d, *J* = 7.3 Hz, 1H), 7.57–7.52 (m, 2H), 7.48 (d, *J* = 7.8 Hz, 1H), 7.42–7.34 (m, 4H), 7.31–7.08 (m, 11H), 7.05–6.98 (m, 1H), 6.82–6.76 (m, 2H), 6.59–6.43 (m, 6H). ^13^C {1H} NMR (126 MHz, CDCl_3_) δ 188.2, 163.0, 161.9 (d, *J_C-F_* = 248.5 Hz), 161.8 (d, *J_C-F_* = 247.4 Hz), 155.9, 153.0, 145.2, 142.3, 141.7, 138.3, 137.8, 135.1, 132.5, 132.4, 131.0, 130.9, 129.9, 129.5, 128.9, 128.8, 128.1, 127.7, 126.5, 125.1, 123.9, 123.1, 120.9, 114.5, 114.33, 114.28, 114.2, 62.3.^19^F NMR (471 MHz, CDCl_3_) δ –113.7, –114.6. IR: *ῡ* = 2921, 2851, 1647, 1528, 1400, 1192, 1134, 1075, 757, 671, 471 cm^−1^. HRMS (ESI) *m*/*z*: [M + H]^+^ calcd. for C_42_H_28_F_2_N: 584.2184; found: 584.2178.

*2-(Bis(4-chlorophenyl)methylene)-1,3,3-triphenyl-2,3-dihydrocyclopenta[b]indole* (**3n**): (eluent: petroleum ether/ethyl acetate 10:1 to 3:1, 20 min, red foam solid, 65.2 mg, 53% yield). ^1^H NMR (500 MHz, CDCl_3_) δ 7.64 (d, *J* = 7.4 Hz, 1H), 7.55–7.49 (m, 2H), 7.48 (d, *J* = 7.8 Hz, 1H), 7.41–7.33 (m, 4H), 7.27 (td, *J* = 7.7, 1.1 Hz, 1H), 7.24–7.19 (m, 3H), 7.19–7.09 (m, 6H), 7.02 (td, *J* = 7.6, 0.9 Hz, 1H), 6.83–6.70 (m, 6H), 6.52–6.46 (m, 2H). ^13^C {1H} NMR (126 MHz, CDCl_3_) δ 188.0, 163.1, 155.5, 153.5, 144.6, 142.5, 141.5, 140.3, 140.0, 134.9, 133.5, 133.1, 131.9, 130.5, 130.1, 129.5, 128.9, 128.8, 128.2, 127.8, 127.6, 127.5, 126.5, 125.1, 124.0, 123.2, 121.0, 62.3. IR: *ῡ* = 3118, 2920, 2850, 1646, 1528, 1402, 1191, 1136, 1075, 755, 670, 470 cm^−1^. HRMS (ESI) *m*/*z*: [M + H]^+^ calcd. for C_42_H_28_Cl_2_N: 616.1593; found: 616.1589.

*2-(Bis(4-bromophenyl)methylene)-1,3,3-triphenyl-2,3-dihydrocyclopenta[b]indole* (**3o**): (eluent: petroleum ether/ethyl acetate 10:1 to 3:1, 20 min, red foam solid, 60.4 mg, 43% yield). ^1^H NMR (500 MHz, CDCl_3_) δ 7.64 (d, *J* = 7.4 Hz, 1H), 7.55–7.45 (m, 3H), 7.40–7.32 (m, 4H), 7.31–7.26 (m, 1H), 7.24–7.19 (m, 3H), 7.20–7.08 (m, 6H), 7.05–7.01 (m, 1H), 6.98–6.88 (m, 4H), 6.68 (d, *J* = 8.4 Hz, 2H), 6.43 (d, *J* = 8.5 Hz, 2H).^13^C {1H} NMR (126 MHz, CDCl_3_) δ 187.9, 163.1, 155.4, 153.5, 144.6, 142.6, 141.5, 140.6, 140.3, 134.9, 132.1, 130.7,130.52, 130.46,130.1, 129.5, 128.9, 128.8, 128.2, 127.8, 126.6, 125.1, 124.0, 123.2, 121.9, 121.3, 121.0, 62.3. IR: *ῡ* = 2920, 2851, 1647, 1529, 1401, 1195, 1135, 1073, 756, 670, 472 cm^−1^. HRMS (ESI) *m*/*z*: [M + H]^+^ calcd. for C_42_H_28_Br_2_N: 706.0563; found: 706.0561.

*2-(Di-p-tolylmethylene)-1,3,3-triphenyl-2,3-dihydrocyclopenta[b]indole* (**3p**): (eluent: petroleum ether/ethyl acetate 10:1 to 3:1, 20 min, red foam solid, 92.0 mg, 80% yield). ^1^H NMR (500 MHz, CDCl_3_) δ 7.64 (d, *J* = 7.5 Hz, 1H), 7.60–7.51 (m, 2H), 7.48 (d, *J* = 7.8 Hz, 1H), 7.38 (d, *J* = 7.1 Hz, 4H), 7.24 (td, *J* = 7.7, 1.2 Hz, 1H), 7.19–7.04 (m, 9H), 6.99 (t, *J* = 7.5 Hz, 1H), 6.71 (d, *J* = 8.0 Hz, 2H), 6.59 (t, *J* = 7.8 Hz, 4H), 6.46 (d, *J* = 8.0 Hz, 2H), 2.16 (s, 3H), 2.04 (s, 3H). ^13^C {1H} NMR (126 MHz, CDCl_3_) δ 188.5, 162.9, 157.3, 151.7, 148.4, 142.0, 141.5, 139.7, 139.3, 137.1, 136.5, 135.5, 130.8, 129.6, 129.4, 129.2, 129.0, 128.1, 127.9, 127.8, 127.5, 126.1, 125.3, 123.6, 122.9, 120.7, 62.3, 21.0, 20.9. IR: *ῡ* = 3122, 2920, 2852, 1648, 1527, 1401, 1190, 1136, 1074, 756, 671, 471 cm^−1^. HRMS (ESI) *m*/*z*: [M + H]^+^ calcd. for C_44_H_34_N: 576.2686; found: 576.2678.

*2-(Bis(4-methoxyphenyl)methylene)-1,3,3-triphenyl-2,3-dihydrocyclopenta[b]indole* (**3q**): (eluent: petroleum ether/ethyl acetate 10:1 to 3:1, 30 min, red foam solid, 91.0 mg, 75% yield). ^1^H NMR (500 MHz, CDCl_3_) δ 7.67 (d, *J* = 7.5 Hz, 1H), 7.61–7.55 (m, 2H), 7.48 (d, *J* = 7.8 Hz, 1H), 7.43–7.34 (m, 4H), 7.24 (td, *J* = 7.7, 1.2 Hz, 1H), 7.19 (t, *J* = 7.5 Hz, 2H), 7.17–7.05 (m, 7H), 7.00 (td, *J* = 7.5, 1.0 Hz, 1H), 6.75 (d, *J* = 8.7 Hz, 2H), 6.51 (d, *J* = 8.7 Hz, 2H), 6.38–6.29 (m, 4H), 3.65 (s, 3H), 3.58 (s, 3H). ^13^C {1H} NMR (126 MHz, CDCl_3_) δ 188.6, 162.7, 158.9, 158.7, 157.5, 151.4, 147.8, 142.1, 141.1, 137.6, 135.5, 135.4, 134.8, 132.4, 131.6, 130.8, 129.5, 129.3, 129.0, 128.5, 128.4, 128.3, 128.2, 127.9, 127.5, 127.3, 126.1, 125.3, 123.6, 122.8, 120.7, 113.8, 113.4, 112.7, 112.5, 62.4, 55.10, 55.07. IR: *ῡ* = 2921, 2851, 1649, 1529, 1400, 1195, 1135, 1073, 756, 669, 481 cm^−1^. HRMS (ESI) *m*/*z*: [M + H]^+^ calcd. for C_44_H_34_NO_2_: 608.2584; found: 608.2578.

*2-((4-Fluorophenyl)(phenyl)methylene)-1,3,3-triphenyl-2,3-dihydrocyclopenta[b]indole* (**3r**): (eluent: petroleum ether/ethyl acetate 10:1 to 3:1, 30 min, red foam solid, 87.0 mg, 77% yield, Z/E = 1.1:1) ^1^H NMR (500 MHz, CDCl_3_) δ 7.64 (dd, *J* = 7.5, 2.8 Hz, 1H), 7.61–7.52 (m, 2H), 7.48 (d, *J* = 7.8 Hz, 1H), 7.45–7.34 (m, 4H), 7.26 (t, *J* = 7.6 Hz, 1H), 7.25–7.05 (m, 9H), 7.04–6.93 (m, 2H), 6.83 (s, 2H), 6.83–6.75 (m, 2H), 6.60–6.42 (m, 4H). ^13^C {1H} NMR (126 MHz, CDCl_3_) δ 188.3, 188.2, 162.99, 162.97, 161.8 (d, *J_C-F_* = 248.1 Hz), 161.7 (*J_C-F_* = 247.3 Hz), 156.5, 156.2, 152.6, 152.5, 146.6, 146.5, 142.2, 142.13, 142.11, 141.8, 141.7, 138.50, 138.47, 138.04, 138.02, 135.2, 132.5, 132.4, 131.0, 130.9, 130.8, 129.81, 129.79, 129.5, 129.2, 128.9, 128.7, 128.6, 128.1, 127.9, 127.8, 127.7, 127.6, 127.5, 127.4, 127.3, 126.9, 126.4, 126.3, 125.18, 125.16, 123.9, 123.1, 120.9, 114.3, 114.20, 114.15, 114.0, 62.3. ^19^F NMR (471 MHz, CDCl_3_) δ -114.0, -114.9. IR: *ῡ* = 2920, 2851, 1647, 1531, 1401, 1188, 1134, 1075, 758, 672, 471 cm^−1^. HRMS (ESI) *m/z*: [M + H]^+^ calcd. for C_42_H_29_FN: 566.2279; found: 566.2271.

*2-((4-Bromophenyl)(phenyl)methylene)-1,3,3-triphenyl-2,3-dihydr*o*cyclopenta[b]indole* (**3s**): (eluent: petroleum ether/ethyl acetate 10:1 to 3:1, 15 min, red foam solid, 85.1 mg, 68% yield, Z/E = 1.1:1). ^1^H NMR (500 MHz, CDCl_3_) δ 7.64 (t, *J* = 6.6 Hz, 1H), 7.58–7.50 (m, 2H), 7.48 (dd, *J* = 7.7, 1.9 Hz, 1H), 7.43–7.33 (m, 4H), 7.26 (td, *J* = 7.7, 1.0 Hz, 1H), 7.25–7.05 (m, 9H), 7.01 (t, *J* = 7.5 Hz, 1H), 6.99–6.88 (m, 3H), 6.83 (s, 2H), 6.78 (t, *J* = 7.8 Hz, 1H), 6.73–6.65 (m, 1H), 6.58–6.52 (m, 1H), 6.45 (d, *J* = 8.5 Hz, 1H). ^13^C {1H} NMR (126 MHz, CDCl_3_) δ 188.2, 188.1, 163.0, 156.3, 156.0, 152.8, 152.8, 146.2, 142.3, 141.7, 141.7, 141.4, 141.3, 140.9, 135.1, 132.2, 130.8, 130.7 130.4, 130.3, 129.9, 129.5, 129.2, 128.9, 128.7, 128.1, 127.9, 127.8, 127.7, 127.5, 127.5, 127.3, 126.9, 126.5, 126.3, 125.2, 123.9, 123.1, 121.7, 121.1, 120.9, 62.3. IR: *ῡ* = 2920, 2852, 1646, 1531, 1464, 1395, 1190, 1134, 1075, 759, 516, 468 cm^−1^. HRMS (ESI) *m/z*: [M + H]^+^ calcd. for C_42_H_29_BrNO: 626.1478; found: 626.1469.

*2-((4-Methoxyphenyl)(phenyl)methylene)-1,3,3-triphenyl-2,3-dihydrocyclopenta[b]indole* (**3t**): (eluent: petroleum ether/ethyl acetate 10:1 to 3:1, 15 min, red foam solid, 94.6 mg, 82% yield, Z/E = 1.3:1). **^1^**H NMR (500 MHz, CDCl_3_) δ 7.65 (t, *J* = 7.5 Hz, 1H), 7.60–7.54 (m, 2H), 7.48 (d, *J* = 7.8 Hz, 1H), 7.44–7.33 (m, 4H), 7.25 (td, *J* = 7.7, 1.1 Hz, 1H), 7.24–7.04 (m, 9H), 7.04–6.93 (m, 2H), 6.88–6.80 (m, 3H), 6.81–6.72 (m, 2H), 6.57 (dd, *J* = 8.2, 1.3 Hz, 1H), 6.53–6.47 (m, 1H), 6.38–6.28 (m, 2H), 3.71 (s, 3H), 3.64 (s, 3H). ^13^C {1H} NMR (126 MHz, CDCl_3_) δ 188.54, 188.50, 162.9, 158.9, 158.7, 157.1, 152.1, 151.6, 148.0, 147.8, 142.7, 142.1, 142.0, 141.9, 141.6, 135.5, 135.4, 135.1, 134.7, 132.2, 130.9, 130.7, 129.54, 129.51, 129.3, 129.0, 128.5, 128.4, 127.9, 127.8, 127.6, 127.5, 127.4, 127.3, 127.1, 126.7, 126.2, 126.1, 125.28, 125.25, 123.7, 122.9, 120.8, 112.8, 112.6, 62.4, 62.3, 55.10, 55.08. IR: *ῡ* = 2920, 2852, 1645, 1532, 1465, 1395, 1189, 1134, 1075, 758, 672, 468 cm^−1^. HRMS (ESI) *m/z*: [M + H]^+^ calcd. for C_43_H_32_NO: 578.2478; found: 578.2470.

*4-(Phenyl(1,3,3-triphenylcyclopenta[b]indol-2(3H)-ylidene)methyl)phenol* (**3u**): (eluent: petroleum ether/ethyl acetate 10:1 to 3:1, 3 h, red foam solid, 38.3mg, 34% yield, Z/E = 1.2:1). ^1^H NMR (500 MHz, CDCl_3_) δ 7.71–7.54 (m, 5H), 7.52–7.45 (m, 2H), 7.45–7.40 (m, 2H), 7.38–7.32 (m, 2H), 7.32–7.24 (m, 5H), 7.22–7.15 (m, 2H), 7.09–6.95 (m, 4H), 6.94–6.86 (m, 1H), 6.85–6.74 (m, 1H), 6.72–6.66 (m, 1H), 6.58–6.39 (m, 3H), 4.55 (s, 1H). ^13^C NMR (126 MHz, CDCl_3_) δ 153.4, 149.0, 145.7, 145.5, 142.8, 142.3, 141.2, 139.0, 134.3, 133.5, 128.02, 127.96, 127.93, 127.85, 127.4, 127.20, 127.15, 127.1, 126.4, 126.2, 125.6, 122.1, 120.6 119.9, 115.9, 114.3, 111.0, 82.5, 81.5. IR: *ῡ* = 2920, 2851, 1646, 1528, 1401, 1193, 1134, 1073, 755, 670, 471 cm^−1^. HRMS (ESI) *m/z*: [M + H]^+^ calcd. for C_42_H_29_NO: 564.2319; found: 564.2322.

*2-(Biphenylmethylene)-7-fluoro-1,3,3-triphenyl-2,3-dihydrocyclopenta[b]indole* (**4a**): (eluent: petroleum ether/ethyl acetate 10:1 to 3:1, 30 min, red foam solid, 102.8 mg, 91% yield). ^1^H NMR (500 MHz, CDCl_3_) δ 7.57–7.48 (m, 2H), 7.42–7.33 (m, 5H), 7.32 (dd, *J* = 8.6, 2.5 Hz, 1H), 7.22–7.06 (m, 9H), 7.00–6.92 (m, 2H), 6.88–6.81 (m, 5H), 6.78 (t, *J* = 7.8 Hz, 2H), 6.57 (d, *J* = 7.2 Hz, 2H). ^13^C {1H} NMR (126 MHz, CDCl_3_) δ 188.38, 188.36, 159.9 (*J_C-F_* = 240.8 Hz), 159.02, 159.01, 158.0, 152.0, 149.0, 142.2, 141.8, 141.7 141.6, 141.5, 135.0, 130.8, 129.5, 129.2, 128.9, 128.8, 128.1, 127.7, 127.5, 127.4, 127.2, 126.9, 126.3, 126.23, 126.15, 121.14, 121.07, 116.0, 115.8, 110.2, 110.0, 62.4. ^19^F NMR (471 MHz, CDCl_3_) δ -119.2. IR: *ῡ* = 2920, 2851, 1645, 1531, 1407, 1188, 1135, 1073, 755, 672, 470 cm^−1^. HRMS (ESI) *m/z*: [M + H]^+^ calcd. for C_42_H_29_FN: 566.2279; found: 566.2275.

*7-Chloro-2-(diphenylmethylene)-1,3,3-triphenyl-2,3-dih*ydro*cyclopenta[b]indole* (**4b**): (eluent: petroleum ether/ethyl acetate 10:1 to 3:1, 20 min, red foam solid, 96.4 mg, 83% yield). ^1^H NMR (500 MHz, CDCl_3_) δ 7.57 (s, 1H), 7.54 (d, *J* = 7.3 Hz, 2H), 7.43–7.31 (m, 5H), 7.27–7.05 (m, 10H), 7.00–6.92 (m, 1H), 6.84 (s, 5H), 6.81–6.73 (m, 2H), 6.60– 6.51 (m, 2H). ^13^C {1H} NMR (126 MHz, CDCl_3_) δ 188.7, 161.3, 158.5, 152.0, 149.3, 142.2, 141.8, 141.6, 141.1, 135.0, 130.8, 129.5, 129.3, 129.20, 129.17, 129.0, 128.9, 128.1, 127.7, 127.6, 127.4, 127.2, 126.9, 126.6, 126.3, 122.9, 121.5, 62.4. IR: *ῡ* = 2920, 2851, 1646, 1530, 1465, 1404, 1190, 1134, 1074, 759, 672,470 cm^−1^. HRMS (ESI) *m/z*: [M + H]^+^ calcd. for C_42_H_29_ClN: 582.1983; found: 582.1979.

*7-Bromo-2-(diphenylmethylene)-1,3,3-triphenyl-2,3-dihydrocyclopenta[b]indole* (**4c**): (eluent: petroleum ether/ethyl acetate 10:1 to 3:1, 30 min, red foam solid, 116.4 mg, 93% yield). ^1^H NMR (500 MHz, CDCl_3_) δ 7.72 (d, *J* = 1.6 Hz, 1H), 7.57–7.51 (m, 2H), 7.39–7.30 (m, 6H), 7.19 (t, *J* = 7.5 Hz, 2H), 7.17–7.05 (m, 7H), 6.95 (t, *J* = 7.5 Hz, 1H), 6.87–6.80 (m, 5H), 6.77 (t, *J* = 7.8 Hz, 2H), 6.57 (dd, *J* = 8.0, 1.1 Hz, 2H). ^13^C {1H} NMR (126 MHz, CDCl_3_) δ 188.5, 161.6, 158.6, 152.0, 149.3, 142.1, 141.7, 141.5, 140.9, 134.9, 132.0, 130.8, 129.4, 129.1, 129.0, 128.8, 128.1, 127.7, 127.5, 127.4, 127.2, 127.1, 126.9, 126.3, 125.7, 121.9, 117.0, 62.4. IR: *ῡ* = 2920, 2850, 1646, 1529, 1464, 1400, 1188, 1135, 1074, 758, 672, 578, 470 cm^−1^. HRMS (ESI) *m/z*: [M + H]^+^ calcd. for C_42_H_29_BrN: 626.1478; found: 626.1472.

*2-(diphenylmethylene)-7-methyl-1,3,3-triphenyl-2,3-dihydrocyclopenta[b]indole* (**4d**): (eluent: petroleum ether/ethyl acetate 10:1 to 3:1, 20 min, red foam solid, 64.0 mg, 57% yield). ^1^H NMR (500 MHz, CDCl_3_) δ 7.60–7.52 (m, 2H), 7.43 (s, 1H), 7.41–7.32 (m, 5H), 7.17 (t, *J* = 7.5 Hz, 2H), 7.15–7.04 (m, 8H), 6.94 (t, *J* = 7.5 Hz, 1H), 6.88–6.79 (m, 5H), 6.76 (t, *J* = 7.8 Hz, 2H), 6.57 (dd, *J* = 8.1, 1.1 Hz, 2H), 2.27 (s, 3H). ^13^C {1H} NMR (126 MHz, CDCl_3_) δ 187.7, 160.9, 156.2, 152.2, 147.6, 142.4, 142.1, 142.00, 141.95, 135.4, 133.4, 130.8, 130.4, 129.5, 129.2, 129.0, 128.5, 127.9, 127.6, 127.30, 127.26, 127.2, 126.7, 126.1, 125.3, 123.6, 120.4, 62.2, 21.4. IR: *ῡ* = 2920, 2851, 1646, 1530, 1405, 1190, 1133, 1074, 758, 673, 519, 469 cm^−1^. HRMS (ESI) *m/z*: [M + H]^+^ calcd. for C_43_H_32_N: 562.2529; found: 562.2524.

*2-(Diphenylmethylene)-7-methoxy-1,3,3-triphenyl-2,3-dihydrocyclopenta[b]indole* (**4e**): (eluent: petroleum ether/ethyl acetate 10:1 to 3:1, 20 min, red foam solid, 72.6 mg, 63% yield). ^1^H NMR (500 MHz, CDCl_3_) δ 7.54 (d, *J* = 7.2 Hz, 2H), 7.37 (d, *J* = 6.9 Hz, 5H), 7.22 (d, *J* = 2.5 Hz, 1H), 7.19–7.05 (m, 9H), 6.98–6.92 (m, 1H), 6.88–6.79 (m, 6H), 6.77 (t, *J* = 7.8 Hz, 2H), 6.59–6.53 (m, 2H), 3.73 (s, 3H). ^13^C {1H} NMR (126 MHz, CDCl_3_) δ 187.0, 156.8, 152.2, 142.4, 142.0, 135.3, 130.8, 129.5, 129.2, 128.9, 128.6, 127.9, 127.6, 127.4, 127.2, 126.7, 126.2, 120.8, 114.3, 109.8, 62.2, 55.8. IR: *ῡ* = 2921, 2821, 1646, 1530, 1399, 1187, 1134, 1074, 760, 672, 468 cm^−1^. HRMS (ESI) *m/z*: [M + H]^+^ calcd. for C_43_H_32_NO: 578.2478; found: 578.2472.

*2-(Diphenylmethylene)-6-fluoro-1,3,3-triphenyl-2,3-dihydrocyclopenta[b]indole* (**4f**): (eluent: petroleum ether/ethyl acetate 10:1 to 3:1, 3 h, red foam solid, 88.0 mg, 78% yield). ^1^H NMR (500 MHz, CDCl_3_) δ 7.63–7.49 (m, 3H), 7.39–7.33 (m, 4H), 7.22–7.07 (m, 10H), 6.96 (t, J = 7.4 Hz, 1H), 6.84 (s, 4H), 6.78 (t, J = 7.7 Hz, 2H), 6.75–6.67 (m, 1H), 6.60–6.53 (m, 2H). ^13^C {1H} NMR (126 MHz, CDCl_3_) δ 190.3,165.0, 164.7, 164.6, 164.0 (d, *J_C-F_* = 248.2 Hz), 156.8, 151.9, 148.3, 142.3, 141.9, 141.6, 141.0, 135.2, 130.8, 129.5, 129.2, 128.9, 128.7, 128.0, 127.7, 127.5, 127.4, 127.2, 126.8, 126.4, 123.6, 123.5, 121.4, 110.7, 110.5, 108.8, 108.6, 62.4.^19^F NMR (471 MHz, CDCl_3_) δ -109.8. IR: *ῡ* = 2921, 2851, 1647, 1530, 1400, 1193, 1133, 1073, 757, 671, 567, 519 cm^−1^. HRMS (ESI) *m/z*: [M + H]^+^ calcd. for C_42_H_29_FN: 566.2279; found: 566.2273.

*2-(Diphenylmethylene)-6-methoxy-1,3,3-triphenyl-2,3-dihydrocyclopenta[b]indole* (**4g**): (eluent: petroleum ether/ethyl acetate 10:1 to 3:1, 2 h, red foam solid, 61.0 mg, 53% yield). ^1^H NMR (500 MHz, CDCl_3_) δ 7.57–7.51 (m, 3H), 7.41–7.35 (m, 4H), 7.19–7.05 (m, 10H), 6.94 (t, *J* = 7.4 Hz, 1H), 6.88–6.79 (m, 5H), 6.77 (t, *J* = 7.7 Hz, 2H), 6.57 (d, *J* = 8.2 Hz, 3H), 3.78 (s, 3H). ^13^C {1H} NMR (126 MHz, CDCl_3_) δ 189.9, 165.0, 161.7, 154.4, 152.0, 146.7, 142.5, 142.1, 141.9, 141.7, 135.5, 130.8, 129.6, 129.3, 128.9, 128.8, 128.5, 128.4, 128.33, 128.27, 127.8, 127.6, 127.3, 127.22, 127.19, 127.16, 126.64, 126.59, 126.2, 123.7, 117.9, 110.3, 106.3, 62.3, 55.5. IR: *ῡ* = 2920, 2851, 1646, 1531, 1400, 1189, 1135, 1074, 759, 671, 471 cm^−1^. HRMS (ESI) *m/z*: [M + H]^+^ calcd. for C_43_H_32_NO: 578.2478; found: 578.2474.

*2-(diphenylmethylene)-8-methyl-1,3,3-triphenyl-2,3-dihydrocyclopenta[b]indole* (**4h**): (eluent: petroleum ether/ethyl acetate 10:1 to 3:1, 1 h, red foam solid, 41.2 mg, 37% yield). ^1^H NMR (500 MHz, CDCl_3_) δ 7.43–7.35 (m, 4H), 7.30 (d, *J* = 7.7 Hz, 1H), 7.23–7.10 (m, 9H), 7.05–6.98 (m, 3H), 6.91–6.86 (m, 1H), 6.85–6.81 (m, 3H), 6.80–6.76 (m, 2H), 6.75–6.68 (m, 3H), 6.56–6.49 (m, 2H), 1.57 (s, 3H). ^13^C {1H} NMR (126 MHz, CDCl_3_) δ 188.7, 163.1, 155.64, 153.58, 148.1, 144.4, 142.4, 142.1, 141.8, 136.7, 135.7, 130.2, 129.6, 129.5, 129.5, 128.3, 127.6, 127.5, 127.2, 126.7, 126.6, 126.5, 126.3, 125.1, 118.3, 61.4, 21.8. IR: ῡ = 2937, 2924, 1553, 1490, 1441, 1392, 1337, 785, 695, 609, 537 cm^−1^. HRMS (ESI) *m/z*: [M + H]^+^ calcd. for C43H32N: 562.2529; found: 562.2519.

*5-chloro-2-(diphenylmethylene)-1,3,3-triphenyl-2,3-dihydrocyclopenta[b]indole* (**4i**): (eluent: petroleum ether/ethyl acetate 10:1 to 3:1, 2 h, red foam solid, 103 mg, 89% yield). ^1^H NMR (500 MHz, CDCl_3_) δ 7.60–7.48 (m, 3H), 7.42–7.36 (m, 4H), 7.28–7.23 (m, 2H), 7.21–7.05 (m, 9H), 7.00–6.90 (m, 2H), 6.84 (s, 5H), 6.78 (t, *J* = 7.7 Hz, 2H), 6.58–6.52 (m, 2H).13C NMR (126 MHz, CDCl_3_) δ 188.7, 159.2, 158.6, 152.3, 149.2, 142.3, 142.0, 141.8, 141.6, 135.0, 130.8, 129.9, 129.7, 129.2, 129.0, 128.8, 128.0, 127.6, 127.5, 127.4, 127.22, 127.17, 126.9, 126.31, 125.29, 124.4, 121.2, 62.7. IR: ῡ = 2980, 2977, 1631, 1537, 1393, 1337, 1127, 748, 695, 488, 436 cm^−1^. HRMS (ESI) *m/z*: [M + H]^+^ calcd. for C42H29ClN: 582.1983; found: 582.1973.

*2-(Diphenylmethylene)-3,3-bis(4-fluorophenyl)-1-phenyl-2,3-dihydrocyclopenta[b]indole* (**4j**): (eluent: petroleum ether/ethyl acetate 10:1 to 3:1, 1 h, red foam solid, 88.6 mg, 76% yield). ^1^H NMR (500 MHz, CDCl_3_) δ 7.66 (d, *J* = 7.5 Hz, 1H), 7.56–7.52 (m, 2H), 7.49 (d, *J* = 7.8 Hz, 1H), 7.38–7.26 (m, 5H), 7.20–7.15 (m, 2H), 7.14–7.11 (m, 1H), 7.07–6.98 (m, 2H), 6.89–6.79 (m, 11H), 6.60–6.54(m, 2H). ^13^C {1H} NMR (126 MHz, CDCl_3_) δ 187.9, 162.8, 161.3 (d, *J_C-F_* = 245.9 Hz), 156.6, 151.8, 148.2, 142.0, 141.9, 141.7, 137.60, 137.57, 135.0, 131.7, 131.0, 130.9, 130.7, 129.9, 129.0, 128.9, 128.8, 128.6, 128.24, 128.21, 127.9, 127.6, 127.5, 127.44, 127.38, 127.3, 127.1, 126.0, 125.1, 124.0, 123.1, 120.9, 114.6, 114.4, 61.0.^19^F NMR (471 MHz, CDCl_3_) δ -116.2. IR: *ῡ* = 2922, 2852, 1646, 1529, 1399, 1190, 1134, 757,672, 572, 470 cm^−1^. HRMS (ESI) *m/z*: [M + H]^+^ calcd. for C_42_H_28_F_2_N: 584.2184; found: 584.2178.

*3,3-Bis(4-chlorophenyl)-2-(diphenylmethylene)-1-phenyl-2,3-dihydrocyclopenta[b]indole* (**4k**): (eluent: petroleum ether/ethyl acetate 10:1 to 3:1, 2 h, red foam solid, 104.4 mg, 85% yield). ^1^H NMR (500 MHz, CDCl_3_) δ 7.66 (d, *J* = 7.6 Hz, 1H), 7.57–7.51 (m, 2H), 7.49 (d, *J* = 7.8 Hz, 1H), 7.32–7.25 (m, 5H), 7.17 (t, *J* = 7.3 Hz, 2H), 7.15–7.07 (m, 5H), 7.07–6.98 (m, 2H), 6.88–6.80 (m, 7H), 6.57 (d, *J* = 7.3 Hz, 2H). ^13^C {1H} NMR (126 MHz, CDCl_3_) δ 187.2, 162.5, 156.8, 151.2, 148.6, 141.79, 141.75, 140.1, 134.9, 132.4, 130.7, 130.0, 129.0, 128.9, 128.0, 127.9, 127.6, 127.44, 127.38, 127.2, 125.0, 124.2, 123.1, 120.9, 61.3. IR: *ῡ* = 2919, 2851, 1644, 1540, 1406, 1260, 1190, 1133, 1076, 756, 633, 477 cm^−1^. HRMS (ESI) *m/z*: [M + H]^+^ calcd. for C_42_H_28_Cl_2_N: 616.1593; found: 616.1589.

*2-(Diphenylmethylene)-1-phenyl-3,3-di-p-tolyl-2,3-dihydrocyclopenta[b]indole* (**4l**): (eluent: petroleum ether/ethyl acetate 10:1 to 3:1, 1 h min, red foam solid, 83.8 mg, 73% yield). ^1^H NMR (500 MHz, CDCl_3_) δ 7.62 (d, *J* = 7.6 Hz, 1H), 7.57–7.52 (m, 2H), 7.47 (d, *J* = 7.7 Hz, 1H), 7.29–7.20 (m, 6H), 7.15 (t, *J* = 7.4 Hz, 2H), 7.12–7.05 (m, 1H), 6.99 (td, *J* = 7.6, 0.9 Hz, 1H), 6.97–6.90 (m, 5H), 6.86–6.80 (m, 5H), 6.77 (t, *J* = 7.8 Hz, 2H), 6.58 (dd, *J* = 8.2, 1.1 Hz, 2H), 2.24 (s, 6H). ^13^C {1H} NMR (126 MHz, CDCl_3_) δ 188.7, 163.1, 156.6, 152.4, 147.5, 142.4, 142.1, 141.9, 138.9, 135.6, 135.4, 130.8, 129.6, 129.4, 129.2, 128.9, 128.5, 128.3, 127.8, 127.3, 127.2, 127.0, 126.6, 125.3, 123.6, 122.9, 120.8, 61.8, 20.9. IR: *ῡ* = 2921, 2852, 1645, 1542, 1398, 1266, 1187, 1135, 1077, 756, 631, 474 cm^−1^. HRMS (ESI) *m/z*: [M + H]^+^ calcd. for C_44_H_34_N: 576.2686; found: 576.2684.

*3,3-Bis(4-(tert-butyl)phenyl)-2-(diphenylmethylene)-1-phenyl-2,3-dihydrocyclopenta[b]indole* (**4m**): (eluent: petroleum ether/ethyl acetate 10:1 to 3:1, 50 min, red foam solid, 105.4 mg, 80% yield). ^1^H NMR (500 MHz, CDCl_3_) δ 7.65 (d, *J* = 7.5 Hz, 1H), 7.57 (d, *J* = 7.3 Hz, 2H), 7.50 (d, *J* = 7.8 Hz, 1H), 7.32–7.24 (m, 5H), 7.20–7.14 (m, 2H), 7.13–7.08 (m, 5H), 7.01 (t, *J* = 7.5 Hz, 1H), 6.93 (t, *J* = 7.4 Hz, 1H), 6.88–6.79 (m, 5H), 6.75 (t, *J* = 7.7 Hz, 2H), 6.60–6.53 (m, 2H), 1.26 (s, 18H).^13^C {1H} NMR (126 MHz, CDCl_3_) δ 188.8, 163.1, 156.7, 152.6, 148.6, 147.4, 142.5, 142.1, 141.9, 138.8, 135.5, 130.8, 129.6, 129.2, 129.0, 128.5, 127.8, 127.3, 127.2, 127.0, 126.4, 125.4, 124. 4, 123.6, 123.0, 120.8, 61.8, 34.2, 31.3. IR: *ῡ* = 2920, 2853, 1646, 1529, 1398, 1188, 1135, 1076, 755, 674, 472 cm^−1^. HRMS (ESI) *m/z*: [M + H]^+^ calcd. for C_50_H_46_N: 660.3625; found: 660.3621.

*2-(Diphenylmethylene)-3,3-bis(4-methoxyphenyl)-1-phenyl-2,3-dihydrocyclopenta[b]indole* (**4n**): (eluent: petroleum ether/ethyl acetate 10:1 to 3:1, 2 h, red foam solid, 81.2 mg, 67% yield). ^1^H NMR (500 MHz, CDCl_3_) δ 7.63 (d, *J* = 7.4 Hz, 1H), 7.57–7.52 (m, 2H), 7.48 (d, *J* = 7.8 Hz, 1H), 7.30–7.24 (m, 6H), 7.16 (t, *J* = 7.4 Hz, 2H), 7.13–7.05 (m, 1H), 7.03–6.92 (m, 2H), 6.87–6.77 (m, 7H), 6.70–6.63 (m, 4H), 6.60 (dd, *J* = 8.2, 1.3 Hz, 2H), 3.73 (s, 6H). ^13^C {1H} NMR (126 MHz, CDCl_3_) δ 188.8, 163.1, 157.8, 156.5, 152.5, 147.5, 142.4, 142.1, 141.8, 135.4, 134.2, 130.8, 130.5, 129.6, 129.2, 128.9, 128.5, 127.9, 127.30, 127.25, 127.1, 126.7, 125.3, 123.7, 123.0, 120.8, 113.1, 61.0, 55.2. IR: *ῡ* = 2919, 2850, 1643, 1533, 1399, 1264, 1190, 1134, 1077, 629, 471 cm^−1^. HRMS (ESI) *m/z*: [M + H]^+^ calcd. for C_44_H_34_NO_2_: 608.2584; found: 608.2580.

*3,3-Di([1,1’-biphenyl]-4-yl)-2-(diphenylmethylene)-1-phenyl-2,3-dihydrocyclopenta[b]indole* (**4o**): (eluent: petroleum ether/ethyl acetate 10:1 to 3:1, 3 h, red foam solid, 103.4 mg, 74% yield). **^1^**H NMR (500 MHz, CDCl_3_) δ 7.68 (d, *J* = 7.5 Hz, 1H), 7.64–7.58 (m, 2H), 7.57–7.46 (m, 9H), 7.45–7.34 (m, 8H), 7.34–7.24 (m, 3H), 7.21–7.14 (m, 2H), 7.10 (t, *J* = 7.4 Hz, 1H), 7.06–6.95 (m, 2H), 6.94–6.85 (m, 2H), 6.85–6.76 (m, 5H), 6.65 (d, *J* = 7.3 Hz, 2H). ^13^C {1H} NMR (126 MHz, CDCl_3_) δ 188.2, 163.0, 156.7, 152.1, 147.9, 142.2, 142.1, 142.0, 141.0, 138.9, 135.3, 130.7, 129.9, 129.8, 129.2, 129.0, 128.7, 127.9, 127.4, 127.17, 127.16, 127.1, 126.9, 126.7, 126.4, 125.3, 123.9, 123.1, 120.9, 61.9. IR: *ῡ* = 2921, 2851, 1646, 1528, 1401, 1188, 1134, 1071, 756, 673, 470 cm^−1^. HRMS (ESI) *m/z*: [M + H]^+^ calcd. for C_54_H_38_N: 700.2999; found: 700.2996.

*2-(Diphenylmethylene)-1-phenyl-3,3-di-m-tolyl-2,3-dihydrocyclopenta[b]indole* (**4p**): (eluent: petroleum ether/ethyl acetate 10:1 to 3:1, 20 min, red foam solid, 52.8 mg, 46% yield). ^1^H NMR (500 MHz, CDCl_3_) δ 7.64 (d, *J* = 7.5 Hz, 1H), 7.58–7.52 (m, 2H), 7.48 (d, *J* = 7.8 Hz, 1H), 7.30–7.19 (m, 4H), 7.19–7.14 (m, 4H), 7.10 (t, *J* = 7.4 Hz, 1H), 7.07–6.98 (m, 3H), 6.95 (s, 1H), 6.90 (d, *J* = 7.5 Hz, 2H), 6.87–6.75 (m, 7H), 6.59 (d, *J* = 7.3 Hz, 2H), 2.20 (s, 6H). ^13^C {1H} NMR (126 MHz, CDCl_3_) δ 188.6, 163.0, 156.8, 152.4, 147.6, 142.5, 142.0, 141.8, 141.7, 136.8, 135.5, 130.8, 130.6, 129.6, 129.3, 128.9, 128.5, 127.9, 127.5, 127.32, 127.27, 127.02, 126.99, 126.73, 126.67, 125.3, 123.7, 123.0, 120.8, 62.2, 21.5. IR: *ῡ* = 2921, 2851, 1645, 1531, 1465, 1416, 1190, 1135, 1073, 758,673, 470 cm^−1^. HRMS (ESI) *m/z*: [M + H]^+^ calcd. for C_44_H_34_N: 576.2686; found: 576.2682.

*2-(Diphenylmethylene)-3,3-bis(3-methoxyphenyl)-1-phenyl-2,3-dihydrocyclopenta[b]indole* (**4q**): (eluent: petroleum ether/ethyl acetate 10:1 to 3:1, 40 min, red foam solid, 35.2 mg, 29% yield). ^1^H NMR (500 MHz, CDCl_3_) δ 7.64 (d, *J* = 7.5 Hz, 1H), 7.59–7.52 (m, 2H), 7.48 (d, *J* = 7.8 Hz, 1H), 7.31–7.23 (m, 1H), 7.16 (t, *J* = 7.4 Hz, 2H), 7.13–6.91 (m, 9H), 6.89–6.78 (m, 7H), 6.69–6.61 (m, 4H), 3.67 (s, 6H). ^13^C {1H} NMR (126 MHz, CDCl_3_) δ 188.1, 163.0, 158.8, 156.6, 152.0, 147.8, 143.2, 142.3, 142.0, 141.9, 135.3, 130.8, 129.7, 129.2, 129.0, 128.6, 128.5, 127.9, 127.4, 127.1, 126.7, 125.3, 123.8, 123.0, 122.2, 120.9, 115.8, 112.0, 62.3, 55.1. IR: *ῡ* = 2921, 2852, 1645, 1534, 1405, 1258, 1192, 1133, 1076, 756, 670, 473 cm^−1^. HRMS (ESI) *m/z*: [M + H]^+^ calcd. for C_44_H_34_NO_2_: 608.2584; found: 608.2575.

### 3.3. General Procedure for the Gram-Scale Synthesis of ***3a***

To a solution of Sc(OTf)_3_ (393.7 mg, 0.8 mmol) in toluene (40 mL) was added of **1a** (1.2 g, 4.0 mmol) and **2a** (1.7 g, 6 mmol) in glass pressure tube (100 mL). The reaction mixture was stirred for 1.5 h at 100 °C. The solvent was concentrated under reduced pressure, and the resulting residue was purified by flash column chromatography on silica gel eluting with petroleum ether/ethyl acetate (petroleum ether/ethyl acetate 10:1 to 3:1), giving the corresponding product **3a** (1.86 g, 83% yield).

### 3.4. General Procedure for the Synthesis of ***5***

LiAlH_4_ (11.4 mg, 0.3 mmol) was added to a solution of **3a** (54.7 mg, 0.1 mmol) in dry THF (1 mL) at 0 ℃. The reaction mixture was stirred for 10 min. Then, water (2 mL) was added. The layers were separated, and the aqueous layer was extracted with CH_2_Cl_2_ (5 mL × 3). The combined organic layers were washed with brine (10 mL), dried over anhydrous Na_2_SO_4_, filtered, and concentrated. The residue was purified by flash chromatography on silica gel, giving the product **5** (eluent: petroleum ether/ethyl acetate 10:1 to 3:1, 10 min, red foam solid, 54.5 mg, 99% yield).

*2-(Diphenylmethylene)-1,3,3-triphenyl-2,3,3a,4-tetrahydrocyclopenta[b]indole* (**5**): ^1^H NMR (500 MHz, CDCl3) δ 7.64 (s, 1H), 7.41–7.29 (m, 2H), 7.19 (tt, J = 8.2, 6.6 Hz, 7H), 7.11–7.06 (m, 2H), 7.06–6.97 (m, 8H), 6.90 (ddd, J = 15.1, 11.5, 4.2 Hz, 3H), 6.83 (t, J = 7.4 Hz, 1H), 6.72 (t, J = 7.7 Hz, 2H), 6.54 (d, J = 7.2 Hz, 2H), 6.49 (d, J = 7.2 Hz, 2H), 5.65 (s, 1H).^13^C {1H} NMR (126 MHz, CDCl_3_) δ 153.0, 147.6, 145.0, 143.9, 143.6, 143.1, 142.9, 141.6, 140.8, 130.0, 128.93, 128.89, 128.8, 128.5, 128.4, 128.1, 127.44, 127.41, 127.3, 126.4, 126.3, 125.7, 125.6, 125.4, 123.3, 121.5, 119.8, 119.4, 119.1, 111.7, 62.3, 49.1. IR: *ῡ* = 3471, 3110, 2920, 2852, 1648, 1527, 1402, 1243, 1190, 1136, 1073, 1027, 752, 671, 467 cm^−1^. HRMS (ESI) *m/z*: [M + H]^+^ calcd. for C_42_H_32_N: 550.2529; found: 550.2525.

### 3.5. General Procedure for the Synthesis of ***6***

Pd(PPh_3_)_4_ (46.2 mg, 0.2 mmol), Cs_2_CO_3_ (97.7 mg, 0.15 mmol), and phenanthren-9-ylboronic acid(66.6 mg, 0.15 mmol) in 1,4-dioxane (2 mL) were added to a solution of **4c** (125.3 mg, 0.3.1.2 mmol). The reaction mixture was stirred for 12 h. The residue was purified by flash chromatography on silica gel, giving the product **6** (eluent: petroleum ether/ethyl acetate 10:1 to 3:1, 12 h, red foam solid, 117.2 mg, 81% yield).

*2-(Diphenylmethylene)-7-(phenanthren-9-yl)-1,3,3-triphenyl-2,3-dihydrocyclopenta[b]indole* (**6**): ^1^H NMR (500 MHz, CDCl_3_) δ 8.70 (d, *J* = 8.3 Hz, 1H), 8.65 (d, *J* = 8.2 Hz, 1H), 7.90 (d, *J* = 8.0 Hz, 1H), 7.85–7.77 (m, 2H), 7.67–7.50 (m, 7H), 7.51–7.38 (m, 6H), 7.22–7.07 (m, 7H), 7.01 (t, *J* = 7.5 Hz, 2H), 6.94 (q, *J* = 7.4 Hz, 2H), 6.89–6.82 (m, 2H), 6.82–6.74 (m, 5H), 6.61 (d, *J* = 7.3 Hz, 2H). ^13^C {1H} NMR (126 MHz, CDCl_3_) δ 188.8, 162.3, 157.4, 152.3, 148.3, 142.3, 142.0, 141.9, 141.8, 138.7, 136.4, 135.1, 131.5, 131.4, 131.3, 130.8, 130.5, 129.8, 129.6, 129.5, 129.22, 129.17, 128.9, 128.6, 128.5, 127.9, 127.64, 127.58, 127.4, 127.3, 127.2, 126.9, 126.77, 126.75, 126.5, 126.42, 126.39, 126.3, 126.2, 125.4, 124.6, 122.8, 122.4, 120.4, 62.5. IR: *ῡ* = 3117, 2922, 2852, 1649, 1529, 1400, 1191, 1136, 1073, 755, 671, 469 cm^−1^. HRMS (ESI) *m/z*: [M + H]^+^ calcd. for C_56_H_38_N: 724.2999; found: 724.2993.

## Figures and Tables

**Table 1:** Optimization of the reaction conditions for (3+2) annulation of **1a** and **2a**.

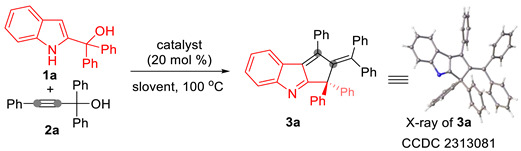				
Entry	Catalyst	Solvent	Time	Yield ^1^ (%)
1	Zn(OTf)_2_	DCE	2 h	62
2	Bi(OTf)_3_	DCE	3 h	70
3	Yb(OTf)_3_	DCE	2 h	64
4	Cu(OTf)_2_	DCE	2 h	63
5	AgNTf_2_	DCE	6 h	13
6	Sc(OTf)_3_	DCE	2 h	74
7	Sc(OTf)_3_	CHCl_3_	1 h	74
8	Sc(OTf)_3_	1,4-dioxane	4 h	76
9	Sc(OTf)_3_	THF	12 h	75
10	Sc(OTf)_3_	EtOH	1 h	41
11	Sc(OTf)_3_	MeCN	6 h	63
12	Sc(OTf)_3_	EA	4 h	78
13	Sc(OTf)_3_	acetone	12 h	58
14	Sc(OTf)_3_	DMF	12 h	N.R.
15	Sc(OTf)_3_	DMSO	12 h	trace
16	Sc(OTf)_3_	PhCl	40 min	78
17	Sc(OTf)_3_	toluene	30 min	80
18 ^2^	Sc(OTf)_3_	toluene	30 min	87
19 ^3^	Sc(OTf)_3_	toluene	24 h	44

Performed with **1a** (0.1 mmol) and **2a** (0.11 mmol) in the presence of catalyst (20 mol %) in solvent at 100 °C. ^1^ Yield of the isolated product. ^2^ **2a** (1.5 equiv) was used. ^3^ Sc(OTf)_3_ (10 mol %) was used.

## Data Availability

The data for the synthesized compounds presented in this study are available in the Supplementary Information.

## Supplementary Materials

The following supporting information can be downloaded at: https://www.mdpi.com/article/10.3390/molecules29061251/s1, X-ray data for products **3a**; copies of ^1^H NMR, ^13^C {1H} NMR spectra.
