# Supplementary material for: Lewis-Acid-Catalyzed (3+2) Annulation of 2-Indolylmethanols with Propargylic Alcohols to Access Cyclopenta[b]indoles
Source: Molecules. 2024 Mar 12;29(6):1251. doi: 10.3390/molecules29061251 (PMC10974089; doi:10.3390/molecules29061251)
Supplement: Supplementary file 1 [file molecules-29-01251-s001.zip › molecules-2875189-supplementary.pdf]

# SUPPORTING INFORMATION

## Lewis Acid-Catalyzed (3+2)-Annulation of 2-Indolylmethanols with Propargylic Alcohols to Access Cyclopenta[*b*]indoles

**Teng-Fei Wu[1,+], Zhao-Jie Fu[1,+], Yi-Rui Zhang [1], Zong-Wang Qiu [1], Bao Qiong Li [1,*] Shao-Shuai Chen [1], Han-Peng Pan[1], Ai-Jun Ma[1], and Xiang-Zhi Zhang [1,*]**

[1] Guangdong Provincial Key Laboratory of Large Animal Models for Biomedicine, School of Pharmacy and Food Engineering, Wuyi University, Jiangmen 529020, P. R. China;

* Correspondence: libq201406@163.com (B. Q. L.); wyuchemzxz@126.com (Z.-X. Z)

† These authors contributed equally to this work.

Contents

1. Failed Substrates ........ S2

2. Structure of **3a** by X-Ray Crystallographic Analysis ........ S2

3. Copies of NMR Spectra ........ S3

## 1. Failed Substrates

These substrates are not suitable for the reaction system and cannot provide corresponding products.

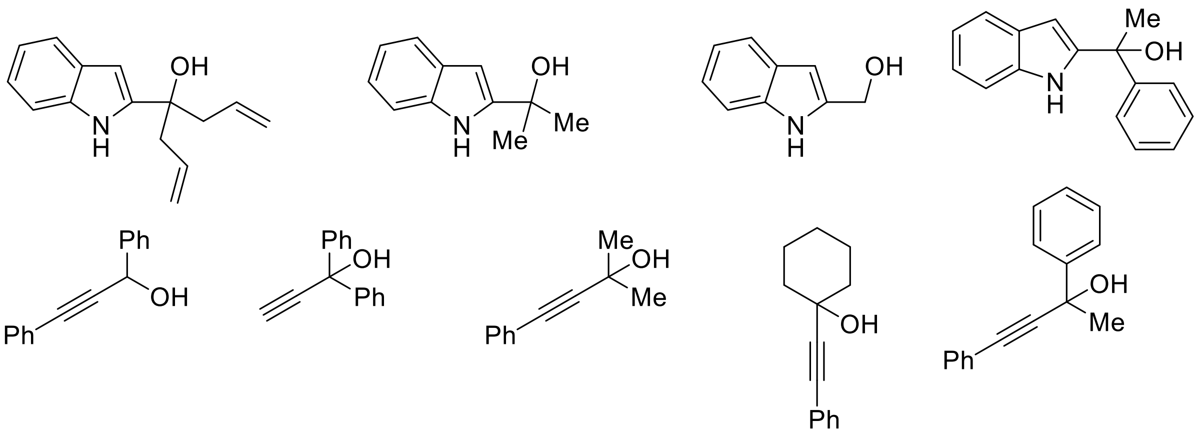


## 2. Structure of 3a by X-Ray Crystallographic Analysis

The single crystal of **3a** which was used for the determination of its relative configurations via X-ray crystallography (see below), was recrystallized from dichloromethane and *n*-hexane. The X-ray single-crystaldetermination was performed on a Bruker APEX II X-ray singlecrystal diffractometer. Compound **3a** monoclinic, space group $P2_1/c$ (no. 14), $a$ = 15.308(2) Å, $b$ = 11.5141(16) Å, $c$ = 16.891(2) Å, $\beta$ = 104.378(4)°, $V$ = 2883.9(7) Å$^3$, $Z$ = 4, $T$ = 150 K, μ(MoKα) = 0.072 mm$^{-1}$, $Dcalc$ = 1.261 g/cm$^3$, 18471 reflections measured (4.326° ⩽ 2Θ ⩽ 52.752°), 5837 unique ($R_{int}$ = 0.0937, $R_{sigma}$ = 0.1137) which were used in all calculations. The final $R_1$ was 0.0657 (I > 2σ(I)) and $wR_2$ was 0.1464 (all data).

### *Crystal structure of 3a*

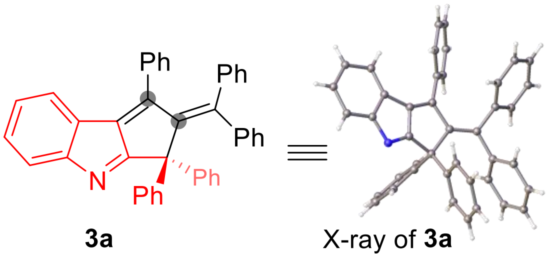


(Displacement ellipsoids are drawn at the 30% probability level.)

### *X-ray crystallographic data of 3a*

| | |
|---|---|
| CCDC | 2313081 |
| Empirical formula | $C_{42}H_{29}N$ |
| Formula weight | 547.66 |
| Temperature/K | 150 |

| | |
|---|---|
| Crystal system | monoclinic |
| Space group | $P2_1/c$ |
| a/Å | 15.308(2) |
| b/Å | 11.5141(16) |
| c/Å | 16.891(2) |
| α/° | 90 |
| β/° | 104.378(4) |
| γ/° | 90 |
| Volume/Å$^3$ | 2883.9(7) |
| Z | 4 |
| $\rho_{calc}$g/cm$^3$ | 1.261 |
| μ/mm$^{-1}$ | 0.072 |
| F(000) | 1152.0 |
| Crystal size/mm$^3$ | 0.12 × 0.08 × 0.05 |
| Radiation | MoKα (λ = 0.71073) |
| 2Θ range for data collection/° | 4.326 to 52.752 |
| Index ranges | $-19 \leq h \leq 18$, $-14 \leq k \leq 13$, $-20 \leq l \leq 21$ |
| Reflections collected | 18471 |
| Independent reflections | 5837 [$R_{int}$ = 0.0937, $R_{sigma}$ = 0.1137] |
| Data/restraints/parameters | 5837/0/388 |
| Goodness-of-fit on F$^2$ | 1.079 |
| Final R indexes [I>=2σ (I)] | $R_1$ = 0.0657, $wR_2$ = 0.1117 |
| Final R indexes [all data] | $R_1$ = 0.1399, $wR_2$ = 0.1464 |
| Largest diff. peak/hole / e Å$^{-3}$ | 0.25/-0.32 |

## 3. Copies of NMR Spectra

(Please see the next page!)

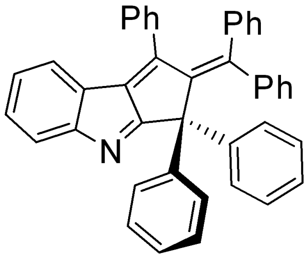


**3a**

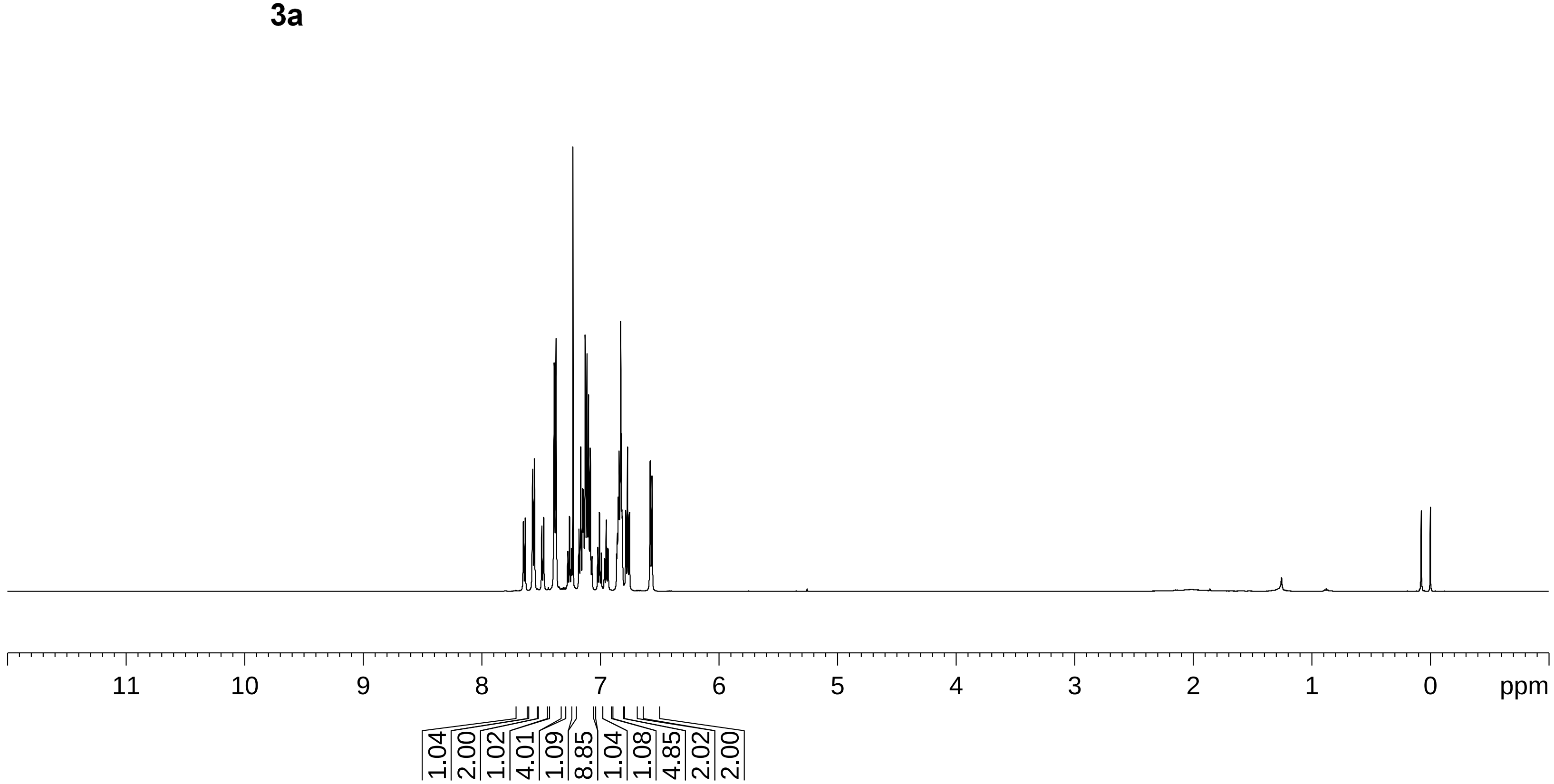


| | |
|---|---|
| NAME | qzw_639 |
| EXPNO | 20 |
| PROCNO | 1 |
| Date_ | 20210604 |
| Time | 18.39 h |
| INSTRUM | Avance NEO 500 |
| PROBHD | Z119470_0332 ( |
| PULPROG | zg30 |
| TD | 65536 |
| SOLVENT | CDCl3 |
| NS | 4 |
| DS | 2 |
| SWH | 10000.000 Hz |
| FIDRES | 0.305176 Hz |
| AQ | 3.2768500 sec |
| RG | 90.4348 |
| DW | 50.000 usec |
| DE | 10.84 usec |
| TE | 296.1 K |
| D1 | 1.00000000 sec |
| TD0 | 1 |
| SFO1 | 500.1530884 MHz |
| NUC1 | 1H |
| P0 | 3.24 usec |
| P1 | 9.72 usec |
| SI | 65536 |
| SF | 500.1500261 MHz |
| WDW | EM |
| SSB | 0 |
| LB | 0.30 Hz |
| GB | 0 |
| PC | 1.00 |

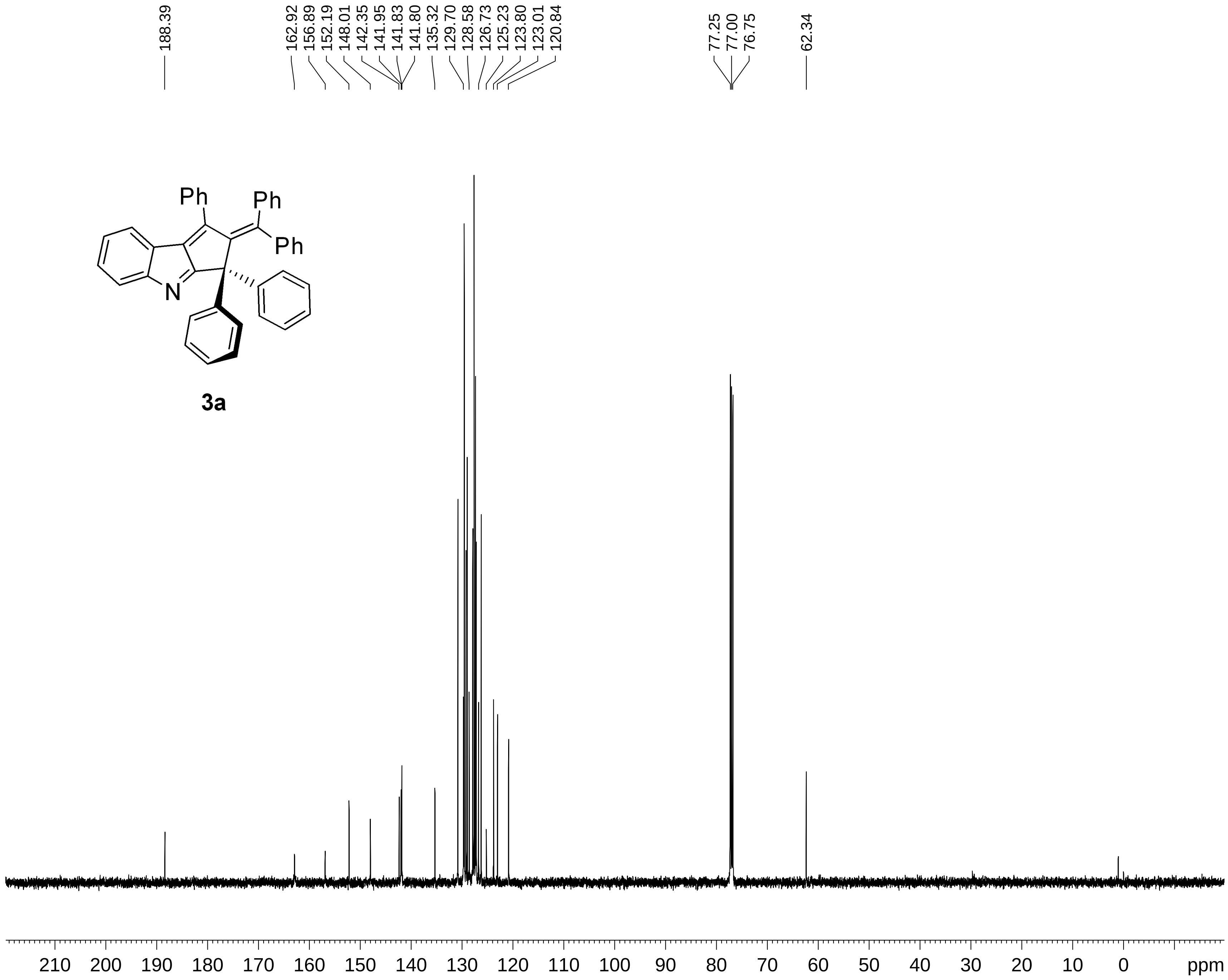


**3a**

| | |
|---|---|
| NAME | qzw_639 |
| EXPNO | 50 |
| PROCNO | 1 |
| Date_ | 20210622 |
| Time | 23.02 h |
| INSTRUM | Avance NEO 500 |
| PROBHD | Z119470_0332 ( |
| PULPROG | zgpg30 |
| TD | 65536 |
| SOLVENT | CDCl3 |
| NS | 400 |
| DS | 4 |
| SWH | 30120.482 Hz |
| FIDRES | 0.919204 Hz |
| AQ | 1.0879476 sec |
| RG | 101 |
| DW | 16.600 usec |
| DE | 6.50 usec |
| TE | 296.2 K |
| D1 | 2.00000000 sec |
| D11 | 0.03000000 sec |
| TD0 | 1 |
| SFO1 | 125.7753938 MHz |
| NUC1 | 13C |
| P0 | 3.33 usec |
| P1 | 10.00 usec |
| SI | 32768 |
| SF | 125.7628270 MHz |
| WDW | EM |
| SSB | 0 |
| LB | 1.00 Hz |
| GB | 0 |
| PC | 1.40 |

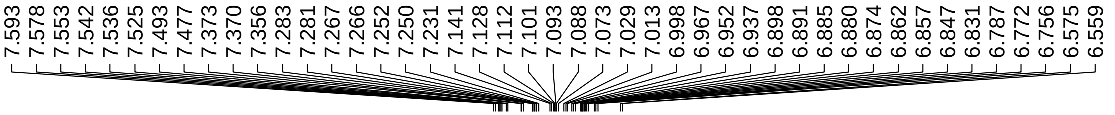


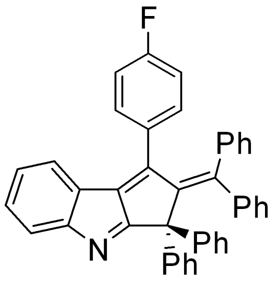


**3b**

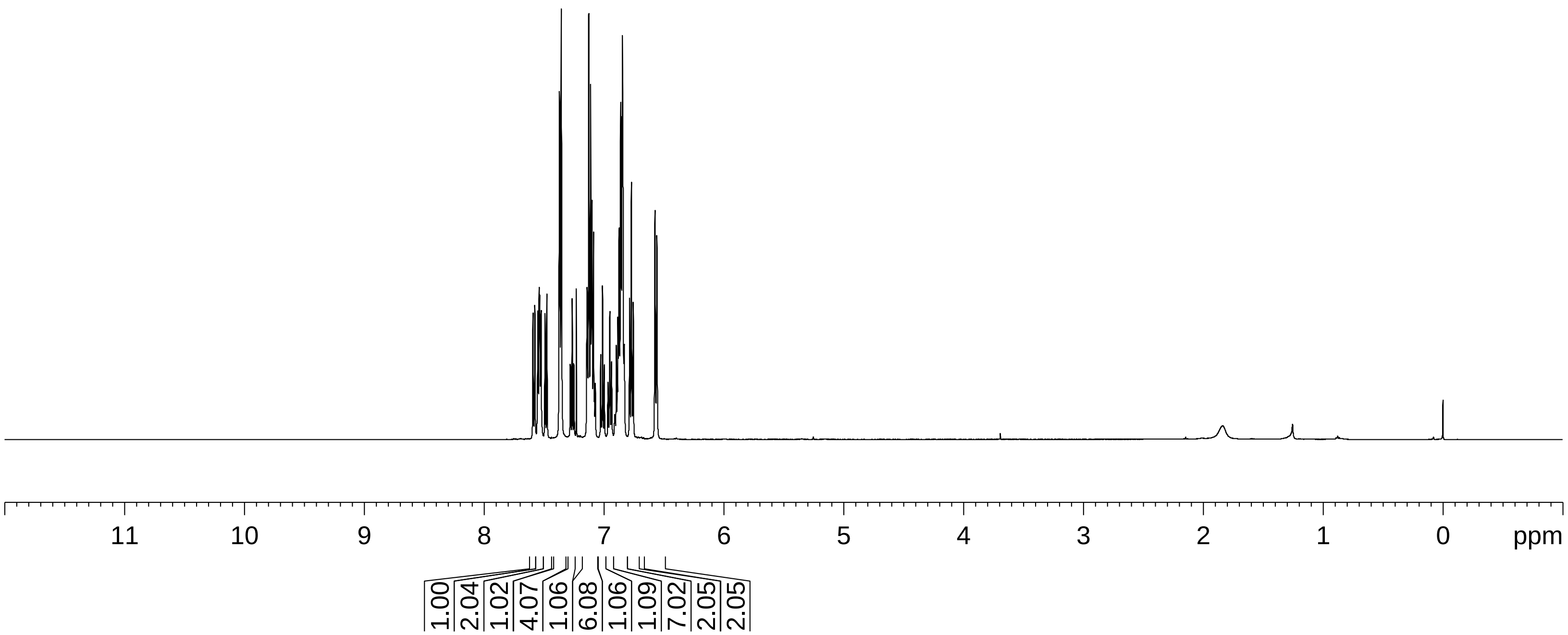


| | |
|---|---|
| NAME | qzw_692_1 |
| EXPNO | 10 |
| PROCNO | 1 |
| Date_ | 20210629 |
| Time | 3.38 h |
| INSTRUM | Avance NEO 500 |
| PROBHD | Z119470_0332 ( |
| PULPROG | zg30 |
| TD | 65536 |
| SOLVENT | CDCl3 |
| NS | 4 |
| DS | 2 |
| SWH | 10000.000 Hz |
| FIDRES | 0.305176 Hz |
| AQ | 3.2768500 sec |
| RG | 83.2 |
| DW | 50.000 usec |
| DE | 10.84 usec |
| TE | 296.1 K |
| D1 | 1.00000000 sec |
| TD0 | 1 |
| SFO1 | 500.1530884 MHz |
| NUC1 | 1H |
| P0 | 3.24 usec |
| P1 | 9.72 usec |
| SI | 65536 |
| SF | 500.1500263 MHz |
| WDW | EM |
| SSB | 0 |
| LB | 0.30 Hz |
| GB | 0 |
| PC | 1.00 |

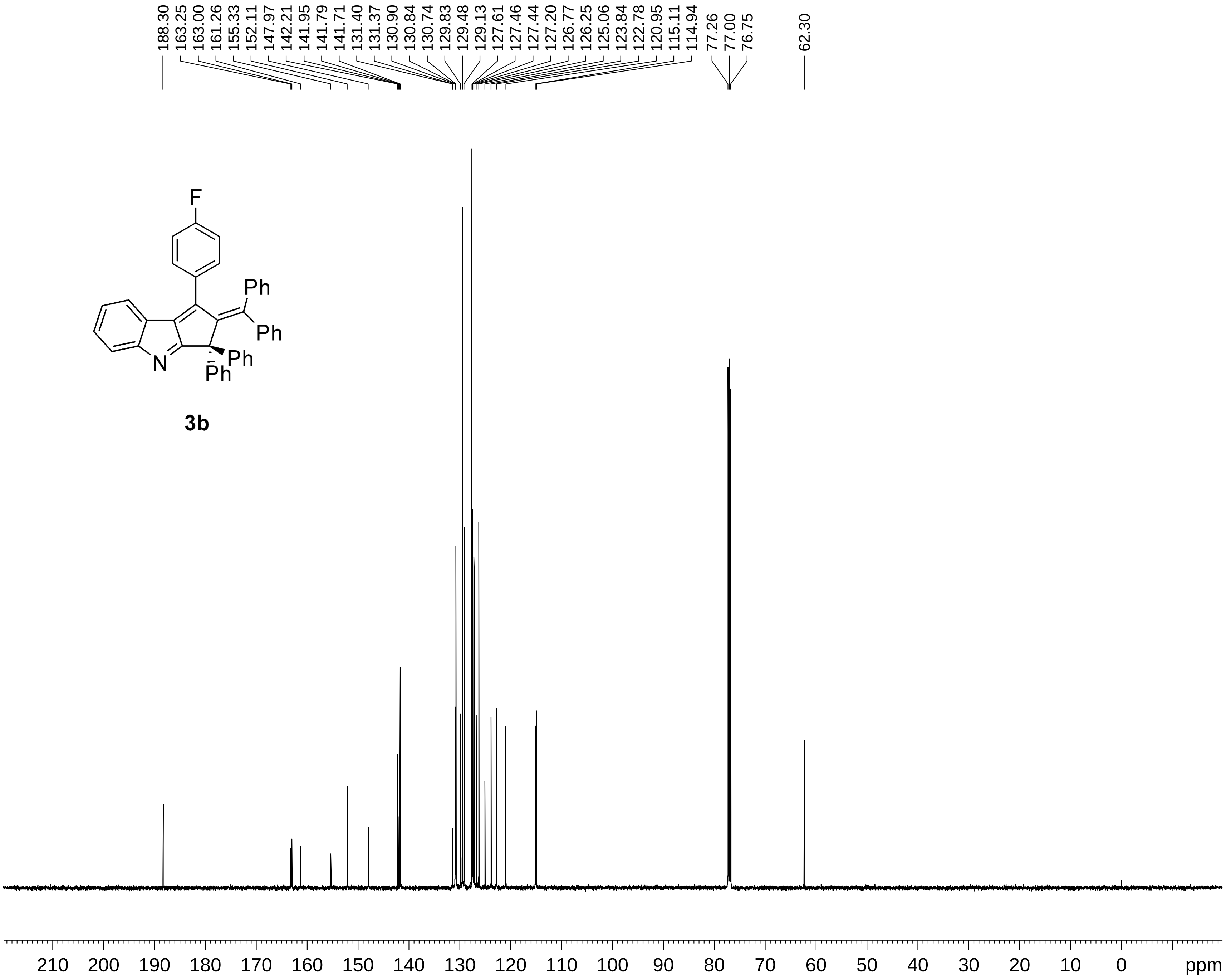


| | |
|---|---|
| NAME | qzw_692_1 |
| EXPNO | 11 |
| PROCNO | 1 |
| Date_ | 20210629 |
| Time | 4.06 h |
| INSTRUM | Avance NEO 500 |
| PROBHD | Z119470_0332 ( |
| PULPROG | zgpg30 |
| TD | 65536 |
| SOLVENT | CDCl3 |
| NS | 520 |
| DS | 4 |
| SWH | 30120.482 Hz |
| FIDRES | 0.919204 Hz |
| AQ | 1.0879476 sec |
| RG | 101 |
| DW | 16.600 usec |
| DE | 6.50 usec |
| TE | 296.2 K |
| D1 | 2.00000000 sec |
| D11 | 0.03000000 sec |
| TD0 | 1 |
| SFO1 | 125.7753938 MHz |
| NUC1 | 13C |
| P0 | 3.33 usec |
| P1 | 10.00 usec |
| SI | 32768 |
| SF | 125.7628298 MHz |
| WDW | EM |
| SSB | 0 |
| LB | 1.00 Hz |
| GB | 0 |
| PC | 1.40 |

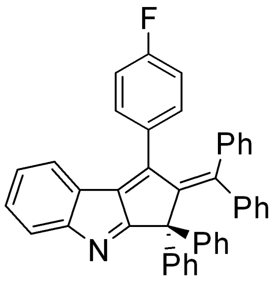


**3b**

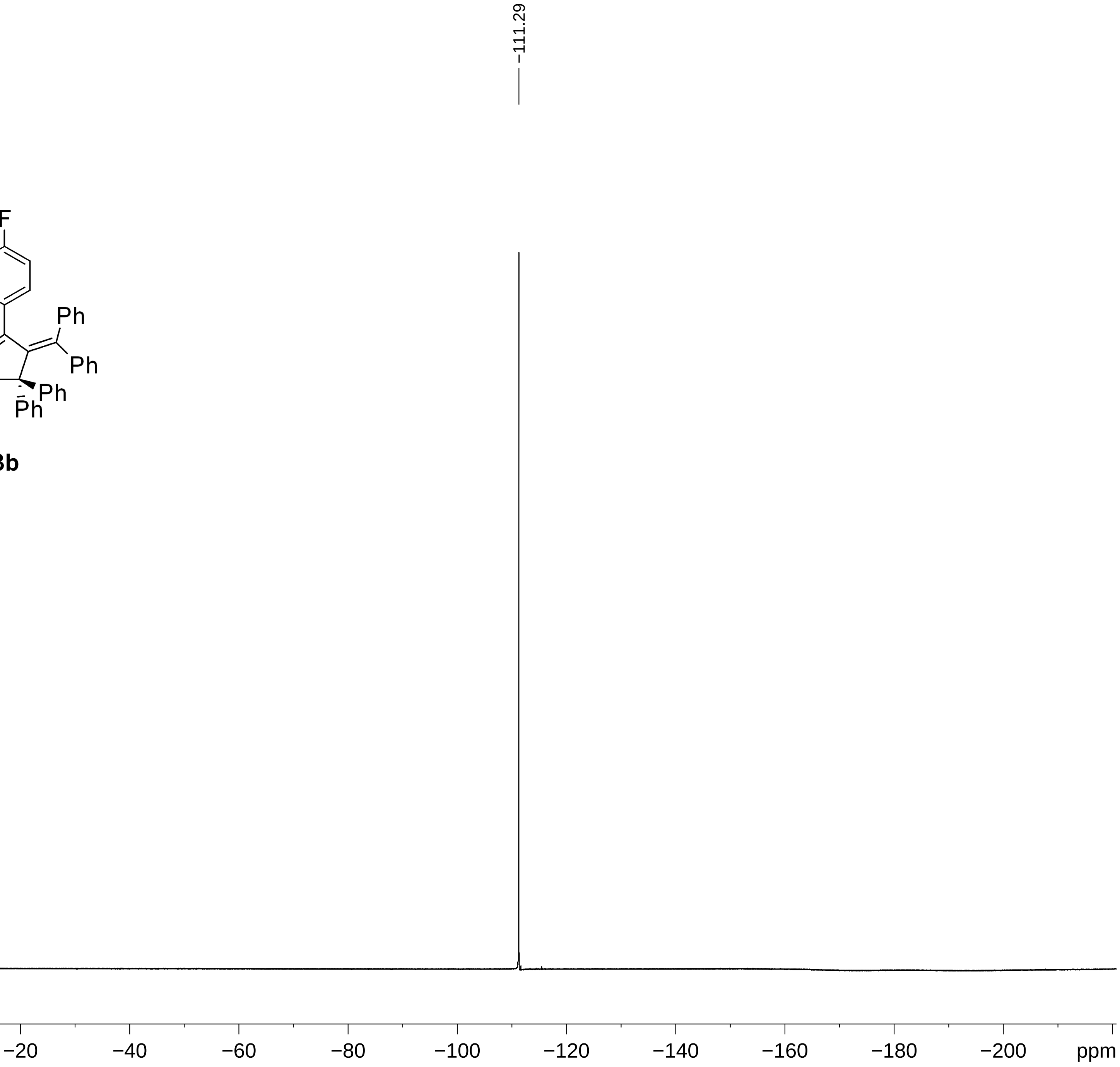


| | |
|---|---|
| NAME | qzw_692_1 |
| EXPNO | 12 |
| PROCNO | 1 |
| Date_ | 20210629 |
| Time | 4.08 h |
| INSTRUM | Avance NEO 500 |
| PROBHD | Z119470_0332 ( |
| PULPROG | zgig |
| TD | 131072 |
| SOLVENT | CDCl3 |
| NS | 16 |
| DS | 4 |
| SWH | 113636.367 Hz |
| FIDRES | 1.733953 Hz |
| AQ | 0.5767668 sec |
| RG | 101 |
| DW | 4.400 usec |
| DE | 6.50 usec |
| TE | 296.2 K |
| D1 | 1.00000000 sec |
| D11 | 0.03000000 sec |
| TD0 | 1 |
| SFO1 | 470.5641349 MHz |
| NUC1 | 19F |
| P1 | 15.00 usec |
| SI | 65536 |
| SF | 470.6111960 MHz |
| WDW | EM |
| SSB | 0 |
| LB | 0.30 Hz |
| GB | 0 |
| PC | 1.00 |

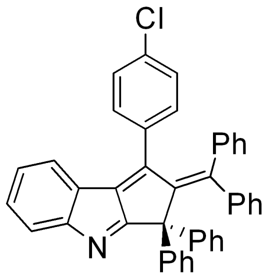

3c

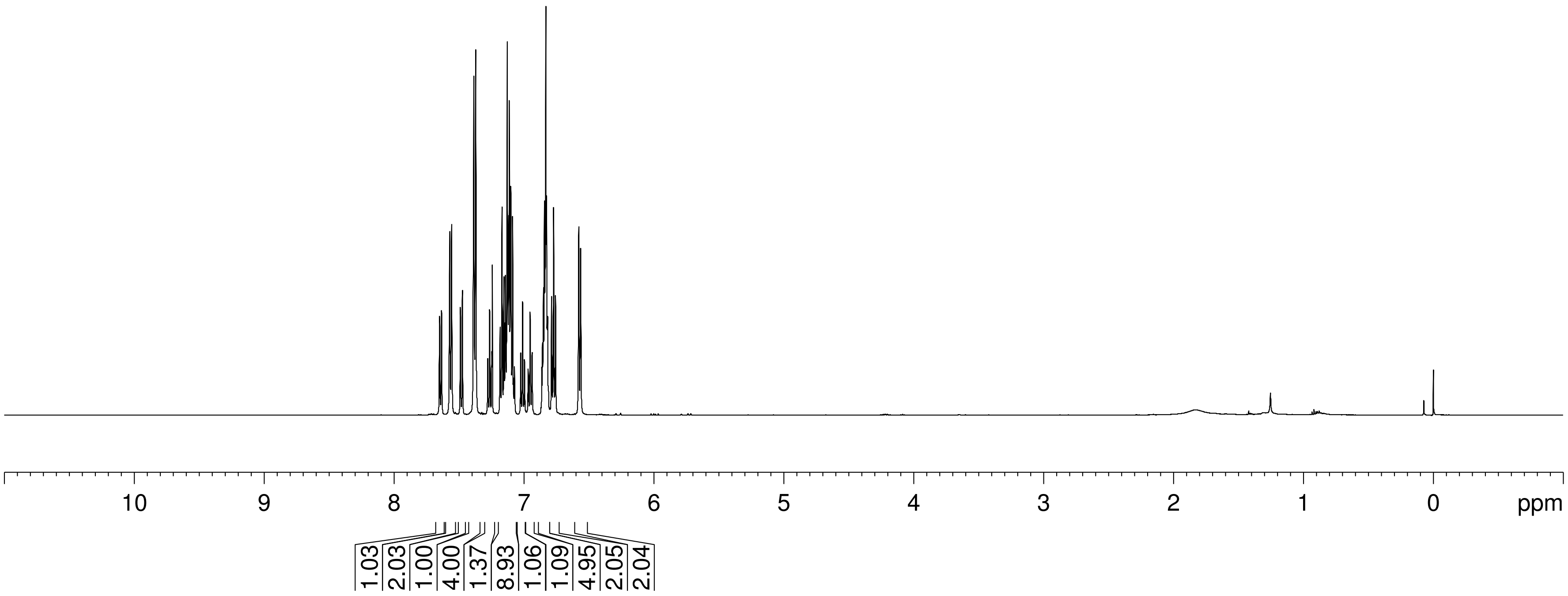

7.572 7.558 7.490 7.475 7.387 7.373 7.280 7.278 7.265 7.263 7.250 7.247 7.244 7.184 7.170 7.154 7.146 7.143 7.139 7.129 7.122 7.114 7.108 7.104 7.102 7.099 7.093 7.088 7.073 7.027 7.025 7.012 7.010 6.997 6.995 6.968 6.953 6.938 6.850 6.843 6.839 6.832 6.827 6.789 6.773 6.757 6.581 6.579 6.565
NAME m_qzw_691_1
EXPNO 20
PROCNO 1
Date_ 20240215
Time 19.46 h
INSTRUM Avance NEO 500
PROBHD Z119470_0332 (
PULPROG zg30
TD 65536
SOLVENT CDCl3
NS 8
DS 2
SWH 10000.000 Hz
FIDRES 0.305176 Hz
AQ 3.2768500 sec
RG 101
DW 50.000 usec
DE 10.84 usec
TE 295.0 K
D1 1.00000000 sec
TD0 1
SFO1 500.1530884 MHz
NUC1 1H
P0 3.24 usec
P1 9.72 usec
SI 65536
SF 500.1500200 MHz
WDW EM
SSB 0
LB 0.30 Hz
GB 0
PC 1.00
10 9 8 7 6 5 4 3 2 1 0 ppm
1.03 2.03 1.00 4.00 1.37 8.93 1.06 1.09 4.95 2.05 2.04

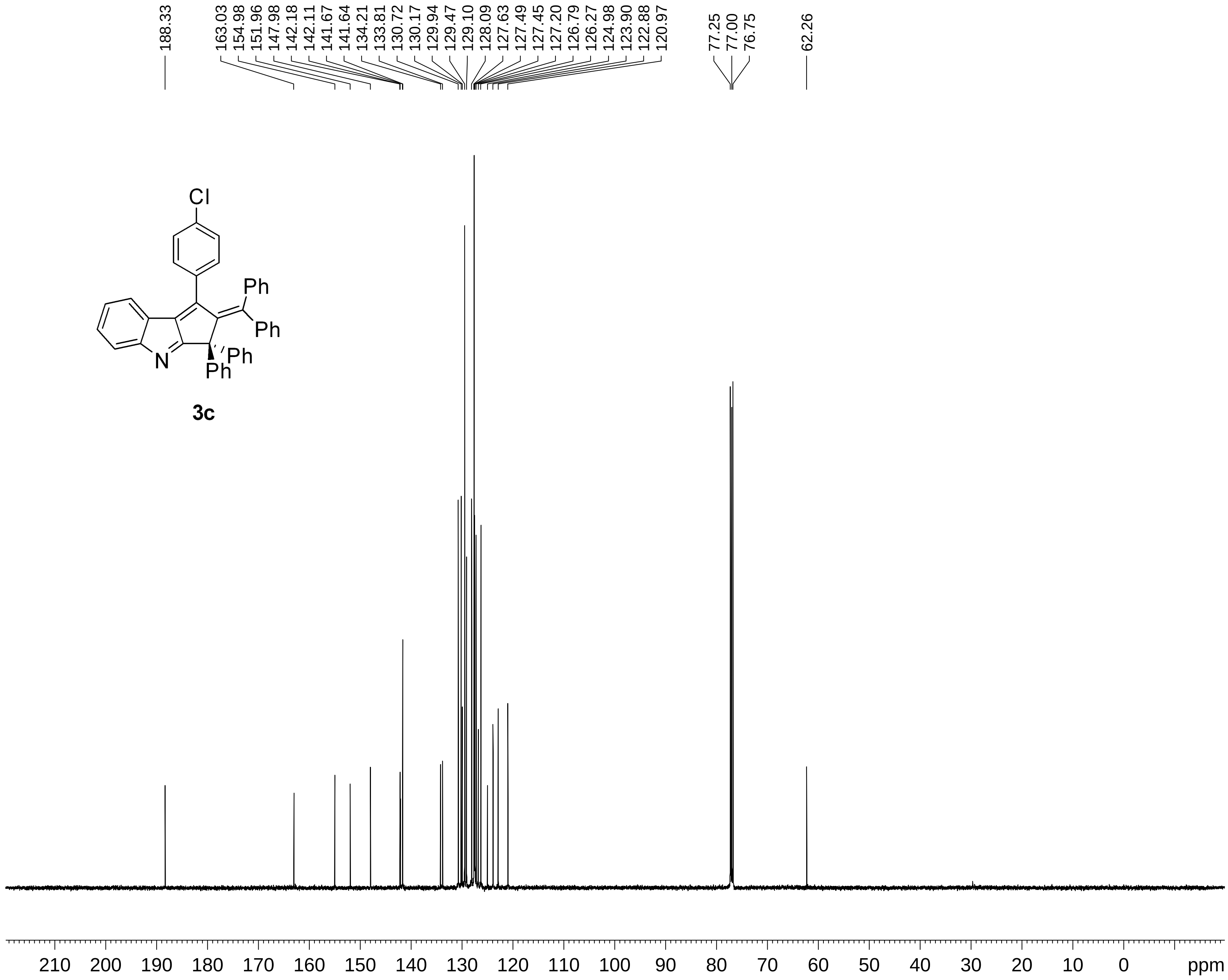


**3c**

| | |
|---|---|
| NAME | qzw_691_1 |
| EXPNO | 20 |
| PROCNO | 1 |
| Date_ | 20210709 |
| Time | 21.34 h |
| INSTRUM | Avance NEO 500 |
| PROBHD | Z119470_0332 ( |
| PULPROG | zgpg30 |
| TD | 65536 |
| SOLVENT | CDCl3 |
| NS | 600 |
| DS | 4 |
| SWH | 30120.482 Hz |
| FIDRES | 0.919204 Hz |
| AQ | 1.0879476 sec |
| RG | 101 |
| DW | 16.600 usec |
| DE | 6.50 usec |
| TE | 296.2 K |
| D1 | 2.00000000 sec |
| D11 | 0.03000000 sec |
| TD0 | 1 |
| SFO1 | 125.7753938 MHz |
| NUC1 | 13C |
| P0 | 3.33 usec |
| P1 | 10.00 usec |
| SI | 32768 |
| SF | 125.7628302 MHz |
| WDW | EM |
| SSB | 0 |
| LB | 1.00 Hz |
| GB | 0 |
| PC | 1.40 |

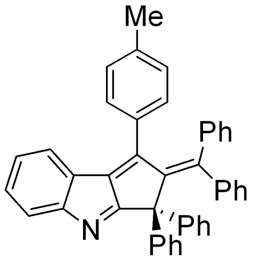


**3d**

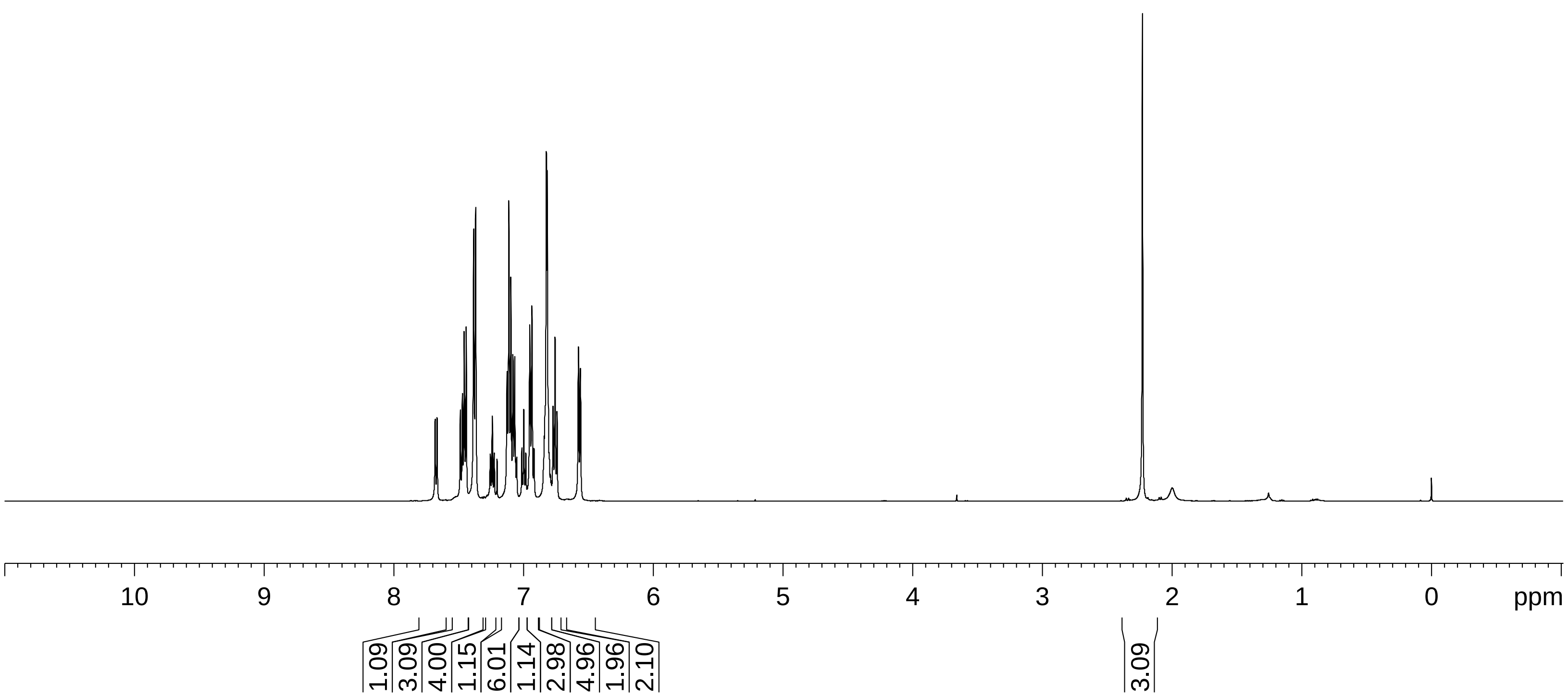


| | |
|---|---|
| NAME | qzw_691_2 |
| EXPNO | 10 |
| PROCNO | 1 |
| Date_ | 20210627 |
| Time | 23.54 h |
| INSTRUM | Avance NEO 500 |
| PROBHD | Z119470_0332 ( |
| PULPROG | zg30 |
| TD | 65536 |
| SOLVENT | CDCl3 |
| NS | 16 |
| DS | 2 |
| SWH | 10000.000 Hz |
| FIDRES | 0.305176 Hz |
| AQ | 3.2768500 sec |
| RG | 53.3333 |
| DW | 50.000 usec |
| DE | 10.84 usec |
| TE | 296.1 K |
| D1 | 1.00000000 sec |
| TD0 | 1 |
| SFO1 | 500.1530884 MHz |
| NUC1 | 1H |
| P0 | 3.24 usec |
| P1 | 9.72 usec |
| SI | 65536 |
| SF | 500.1500400 MHz |
| WDW | EM |
| SSB | 0 |
| LB | 0.30 Hz |
| GB | 0 |
| PC | 1.00 |

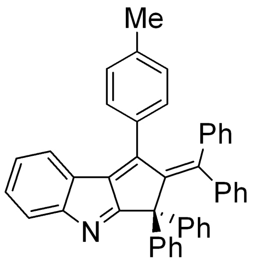


**3d**

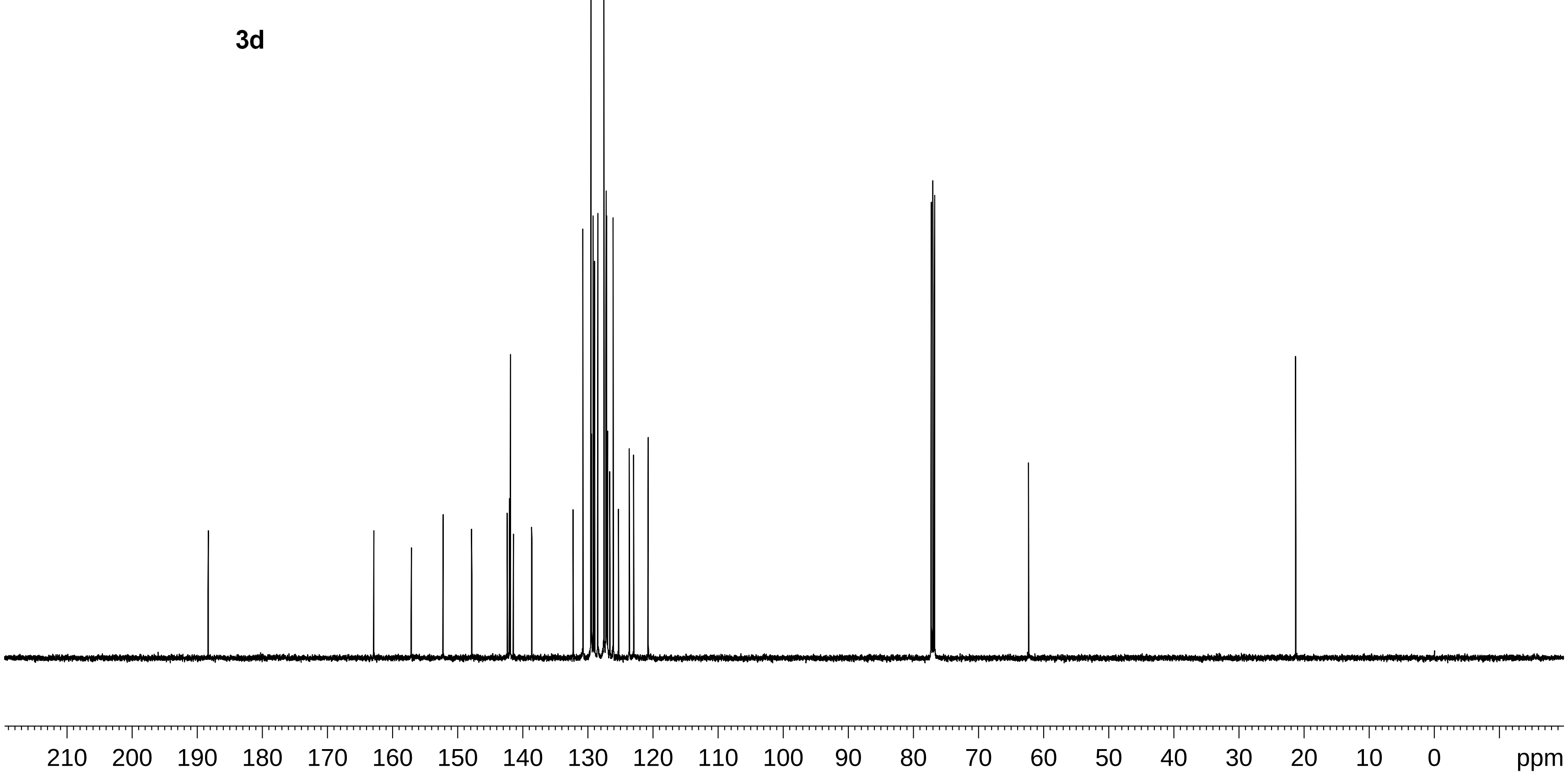


NAME qzw_691_2
EXPNO 11
PROCNO 1
Date_ 20210628
Time 0.06 h
INSTRUM Avance NEO 500
PROBHD Z119470_0332 (
PULPROG zgpg30
TD 65536
SOLVENT CDCl3
NS 200
DS 4
SWH 30120.482 Hz
FIDRES 0.919204 Hz
AQ 1.0879476 sec
RG 101
DW 16.600 usec
DE 6.50 usec
TE 296.1 K
D1 2.00000000 sec
D11 0.03000000 sec
TD0 1
SFO1 125.7753938 MHz
NUC1 13C
P0 3.33 usec
P1 10.00 usec
SI 32768
SF 125.7628371 MHz
WDW EM
SSB 0
LB 1.00 Hz
GB 0
PC 1.40

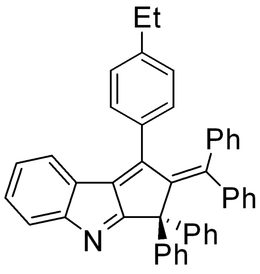

Et
Ph
Ph
N
Ph
Ph


**3e**

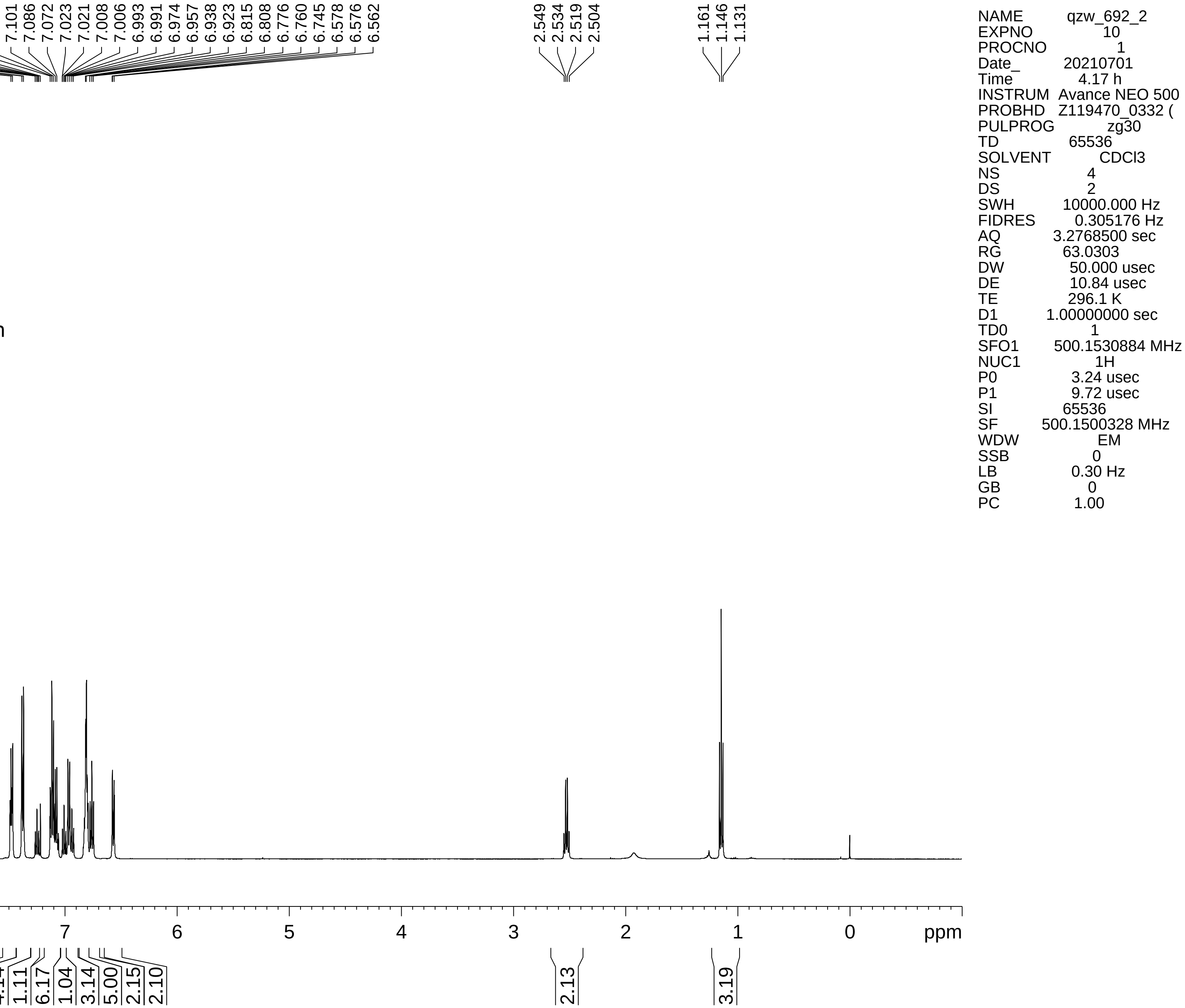

7.692
7.677
7.482
7.466
7.384
7.370
7.266
7.263
7.250
7.248
7.235
7.233
7.218
7.130
7.117
7.101
7.086
7.072
7.023
7.021
7.008
7.006
6.993
6.991
6.974
6.957
6.938
6.923
6.815
6.808
6.776
6.760
6.745
6.578
6.576
6.562
2.549
2.534
2.519
2.504
1.161
1.146
1.131
NAME qzw_692_2
EXPNO 10
PROCNO 1
Date_ 20210701
Time 4.17 h
INSTRUM Avance NEO 500
PROBHD Z119470_0332 (
PULPROG zg30
TD 65536
SOLVENT CDCl3
NS 4
DS 2
SWH 10000.000 Hz
FIDRES 0.305176 Hz
AQ 3.2768500 sec
RG 63.0303
DW 50.000 usec
DE 10.84 usec
TE 296.1 K
D1 1.00000000 sec
TD0 1
SFO1 500.1530884 MHz
NUC1 1H
P0 3.24 usec
P1 9.72 usec
SI 65536
SF 500.1500328 MHz
WDW EM
SSB 0
LB 0.30 Hz
GB 0
PC 1.00
9
8
7
6
5
4
3
2
1
0
ppm
1.06
3.09
4.14
1.11
6.17
1.04
3.14
5.00
2.15
2.10
2.13
3.19

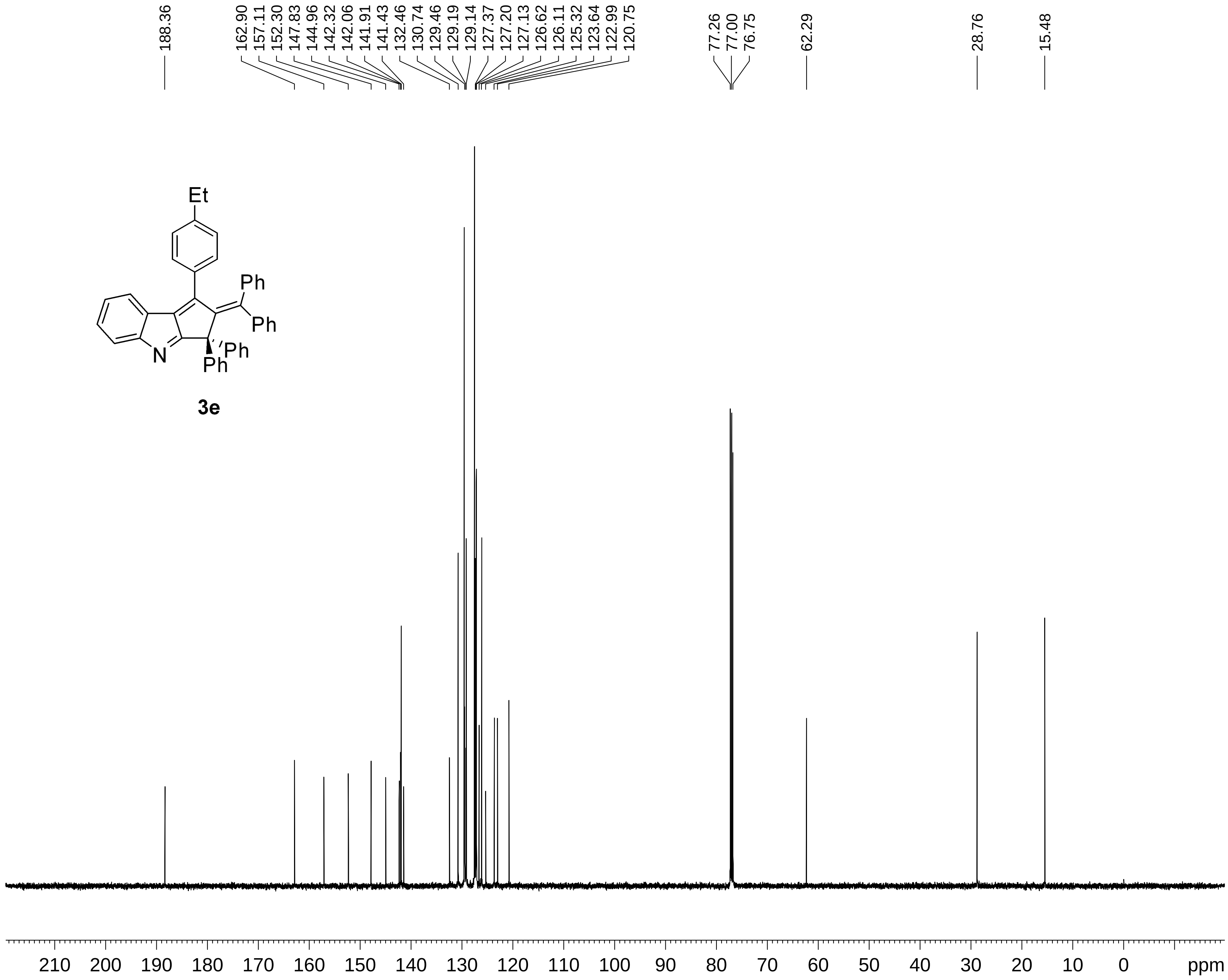


**3e**

| | |
|---|---|
| NAME | qzw_692_2 |
| EXPNO | 11 |
| PROCNO | 1 |
| Date_ | 20210701 |
| Time | 4.29 h |
| INSTRUM | Avance NEO 500 |
| PROBHD | Z119470_0332 ( |
| PULPROG | zgpg30 |
| TD | 65536 |
| SOLVENT | CDCl3 |
| NS | 200 |
| DS | 4 |
| SWH | 30120.482 Hz |
| FIDRES | 0.919204 Hz |
| AQ | 1.0879476 sec |
| RG | 101 |
| DW | 16.600 usec |
| DE | 6.50 usec |
| TE | 296.1 K |
| D1 | 2.00000000 sec |
| D11 | 0.03000000 sec |
| TD0 | 1 |
| SFO1 | 125.7753938 MHz |
| NUC1 | 13C |
| P0 | 3.33 usec |
| P1 | 10.00 usec |
| SI | 32768 |
| SF | 125.7628325 MHz |
| WDW | EM |
| SSB | 0 |
| LB | 1.00 Hz |
| GB | 0 |
| PC | 1.40 |

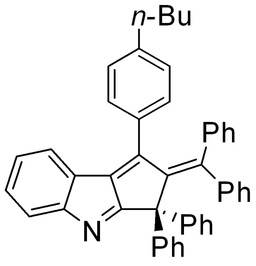


**3f**

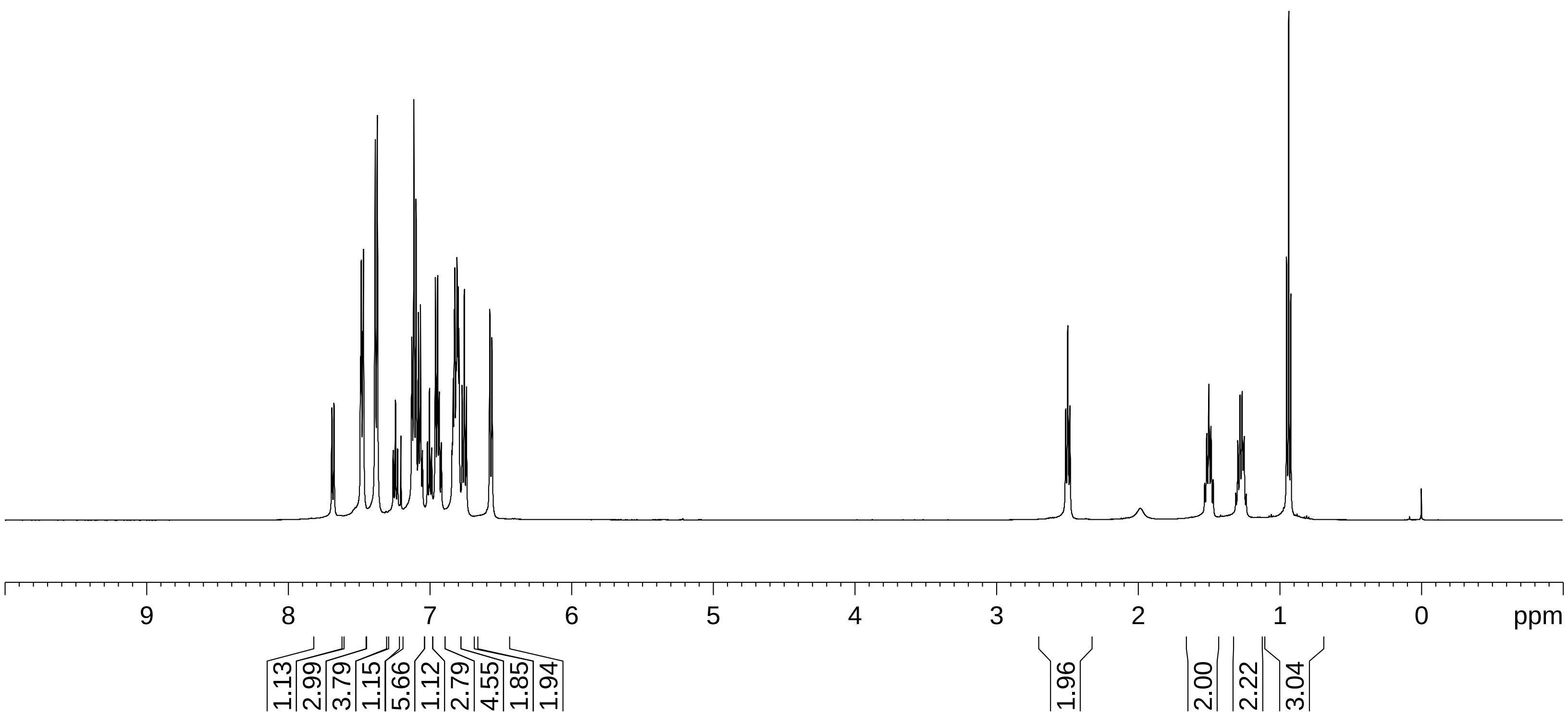


| | |
|---|---|
| NAME | qzw_694_2 |
| EXPNO | 10 |
| PROCNO | 1 |
| Date_ | 20210701 |
| Time | 5.14 h |
| INSTRUM | Avance NEO 500 |
| PROBHD | Z119470_0332 ( |
| PULPROG | zg30 |
| TD | 65536 |
| SOLVENT | CDCl3 |
| NS | 4 |
| DS | 2 |
| SWH | 10000.000 Hz |
| FIDRES | 0.305176 Hz |
| AQ | 3.2768500 sec |
| RG | 46.2222 |
| DW | 50.000 usec |
| DE | 10.84 usec |
| TE | 296.1 K |
| D1 | 1.00000000 sec |
| TD0 | 1 |
| SFO1 | 500.1530884 MHz |
| NUC1 | 1H |
| P0 | 3.24 usec |
| P1 | 9.72 usec |
| SI | 65536 |
| SF | 500.1500404 MHz |
| WDW | EM |
| SSB | 0 |
| LB | 0.30 Hz |
| GB | 0 |
| PC | 1.00 |

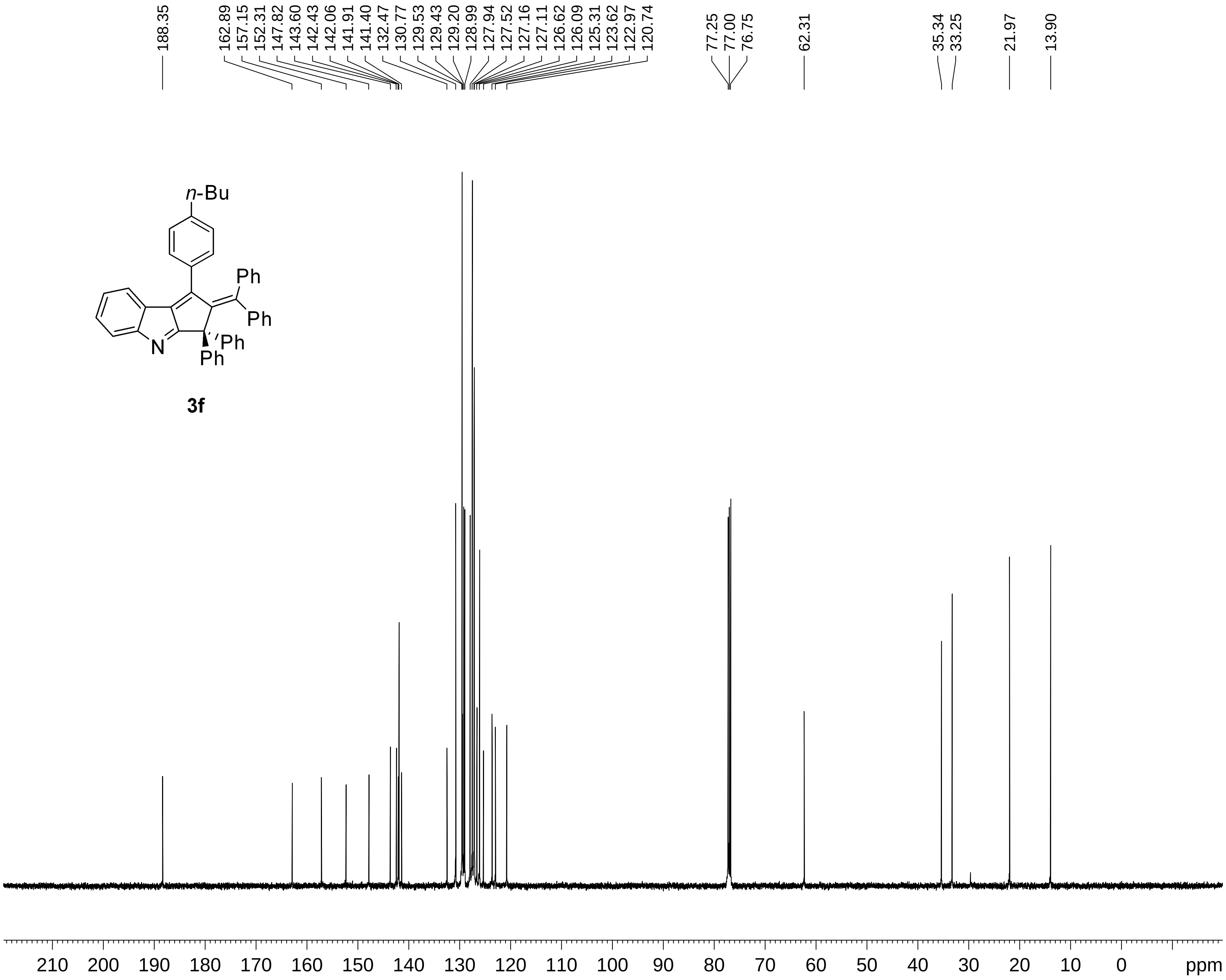


| | |
|---|---|
| NAME | qzw_694_2 |
| EXPNO | 11 |
| PROCNO | 1 |
| Date_ | 20210701 |
| Time | 5.25 h |
| INSTRUM | Avance NEO 500 |
| PROBHD | Z119470_0332 ( |
| PULPROG | zgpg30 |
| TD | 65536 |
| SOLVENT | CDCl3 |
| NS | 200 |
| DS | 4 |
| SWH | 30120.482 Hz |
| FIDRES | 0.919204 Hz |
| AQ | 1.0879476 sec |
| RG | 101 |
| DW | 16.600 usec |
| DE | 6.50 usec |
| TE | 296.2 K |
| D1 | 2.00000000 sec |
| D11 | 0.03000000 sec |
| TD0 | 1 |
| SFO1 | 125.7753938 MHz |
| NUC1 | 13C |
| P0 | 3.33 usec |
| P1 | 10.00 usec |
| SI | 32768 |
| SF | 125.7628366 MHz |
| WDW | EM |
| SSB | 0 |
| LB | 1.00 Hz |
| GB | 0 |
| PC | 1.40 |

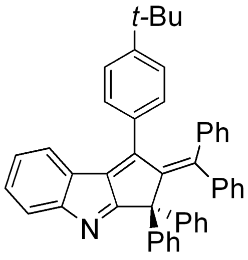


**3g**

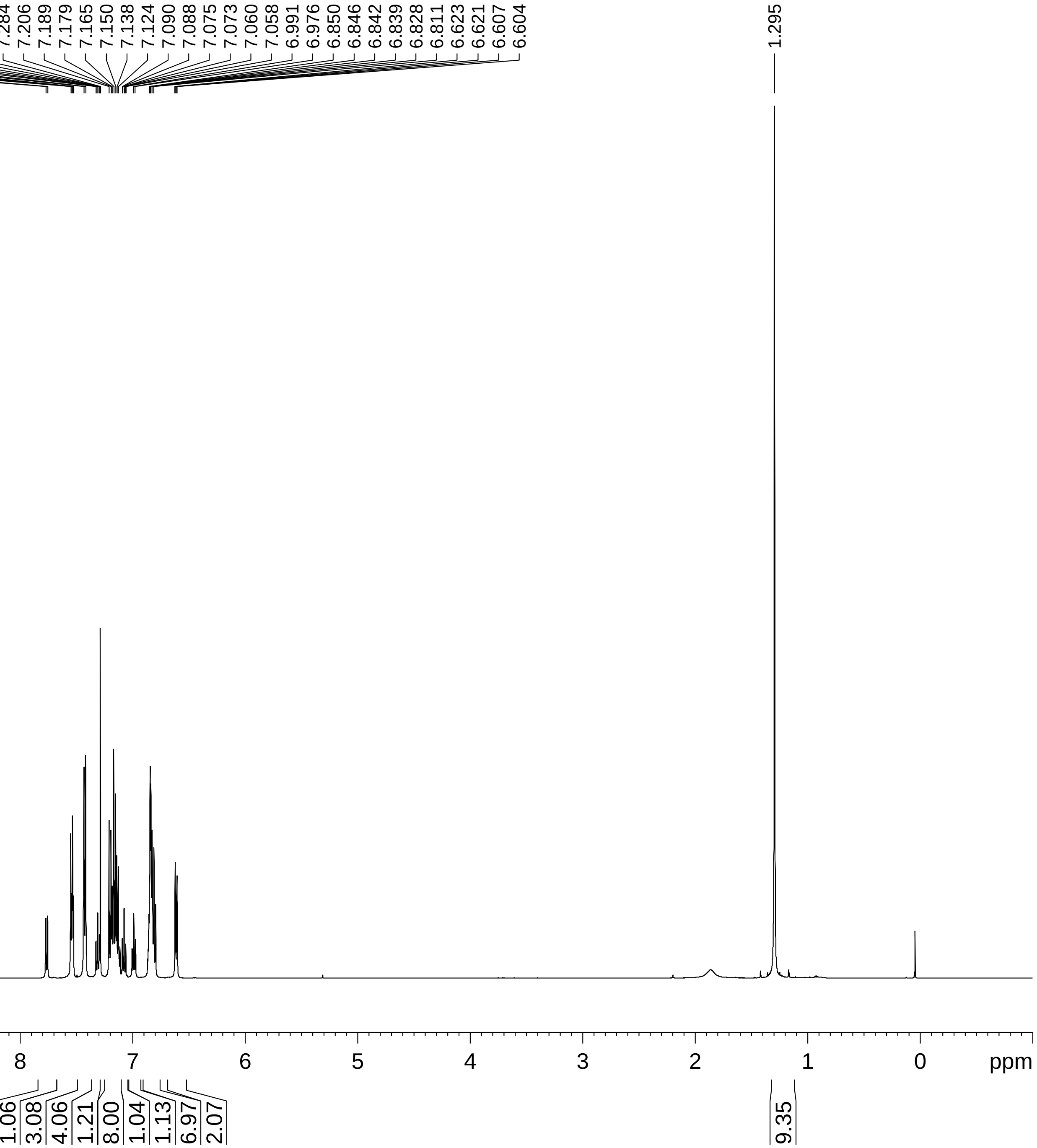


| | |
|---|---|
| NAME | qzw_692_3 |
| EXPNO | 10 |
| PROCNO | 1 |
| Date_ | 20210701 |
| Time | 4.33 h |
| INSTRUM | Avance NEO 500 |
| PROBHD | Z119470_0332 ( |
| PULPROG | zg30 |
| TD | 65536 |
| SOLVENT | CDCl3 |
| NS | 4 |
| DS | 2 |
| SWH | 10000.000 Hz |
| FIDRES | 0.305176 Hz |
| AQ | 3.2768500 sec |
| RG | 86.6667 |
| DW | 50.000 usec |
| DE | 10.84 usec |
| TE | 296.1 K |
| D1 | 1.00000000 sec |
| TD0 | 1 |
| SFO1 | 500.1530884 MHz |
| NUC1 | 1H |
| P0 | 3.24 usec |
| P1 | 9.72 usec |
| SI | 65536 |
| SF | 500.1500000 MHz |
| WDW | EM |
| SSB | 0 |
| LB | 0.30 Hz |
| GB | 0 |
| PC | 1.00 |

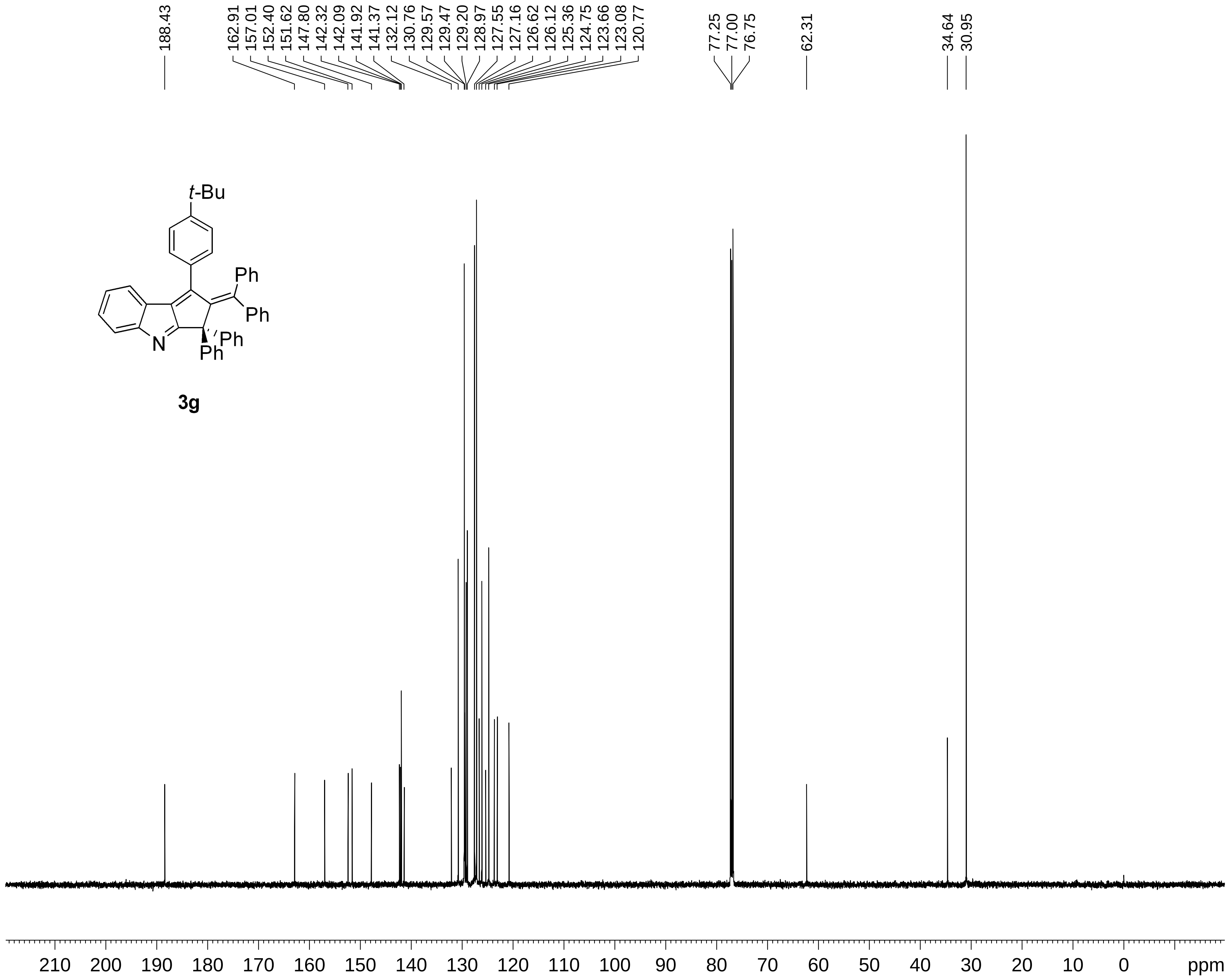


| | |
|---|---|
| NAME | qzw_692_3 |
| EXPNO | 11 |
| PROCNO | 1 |
| Date_ | 20210701 |
| Time | 4.55 h |
| INSTRUM | Avance NEO 500 |
| PROBHD | Z119470_0332 ( |
| PULPROG | zgpg30 |
| TD | 65536 |
| SOLVENT | CDCl3 |
| NS | 400 |
| DS | 4 |
| SWH | 30120.482 Hz |
| FIDRES | 0.919204 Hz |
| AQ | 1.0879476 sec |
| RG | 101 |
| DW | 16.600 usec |
| DE | 6.50 usec |
| TE | 296.2 K |
| D1 | 2.00000000 sec |
| D11 | 0.03000000 sec |
| TD0 | 1 |
| SFO1 | 125.7753938 MHz |
| NUC1 | 13C |
| P0 | 3.33 usec |
| P1 | 10.00 usec |
| SI | 32768 |
| SF | 125.7628274 MHz |
| WDW | EM |
| SSB | 0 |
| LB | 1.00 Hz |
| GB | 0 |
| PC | 1.40 |

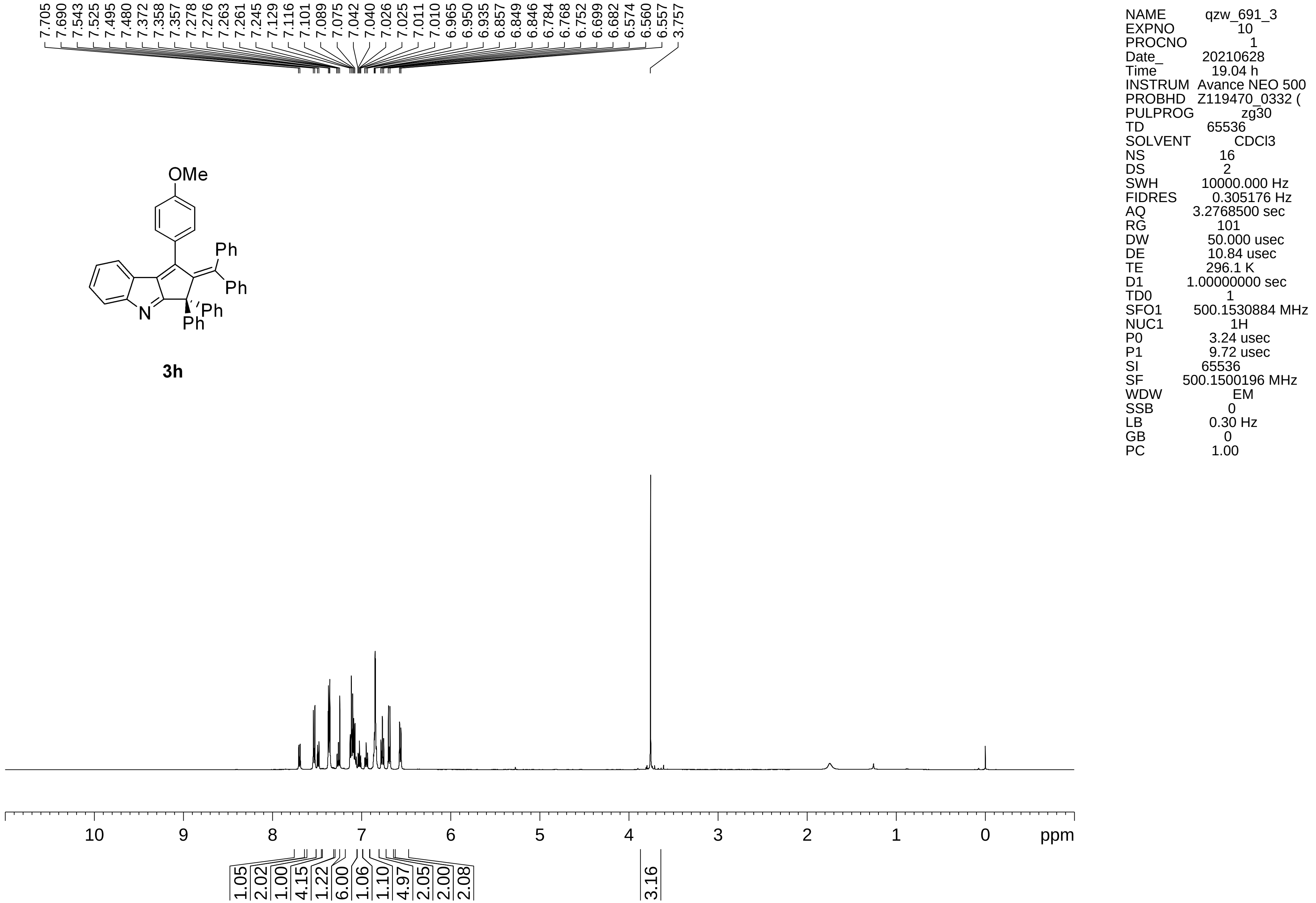
7.705
7.690
7.543
7.525
7.495
7.480
7.372
7.358
7.357
7.278
7.276
7.263
7.261
7.245
7.129
7.116
7.101
7.089
7.075
7.042
7.040
7.026
7.025
7.011
7.010
6.965
6.950
6.935
6.857
6.849
6.846
6.784
6.768
6.752
6.699
6.682
6.574
6.560
6.557
3.757
OMe
Ph
Ph
Ph
Ph
N
3h
NAME qzw_691_3
EXPNO 10
PROCNO 1
Date_ 20210628
Time 19.04 h
INSTRUM Avance NEO 500
PROBHD Z119470_0332 (
PULPROG zg30
TD 65536
SOLVENT CDCl3
NS 16
DS 2
SWH 10000.000 Hz
FIDRES 0.305176 Hz
AQ 3.2768500 sec
RG 101
DW 50.000 usec
DE 10.84 usec
TE 296.1 K
D1 1.00000000 sec
TD0 1
SFO1 500.1530884 MHz
NUC1 1H
P0 3.24 usec
P1 9.72 usec
SI 65536
SF 500.1500196 MHz
WDW EM
SSB 0
LB 0.30 Hz
GB 0
PC 1.00
10
9
8
7
6
5
4
3
2
1
0
ppm
1.05
2.02
1.00
4.15
1.22
6.00
1.06
1.10
4.97
2.05
2.00
2.08
3.16

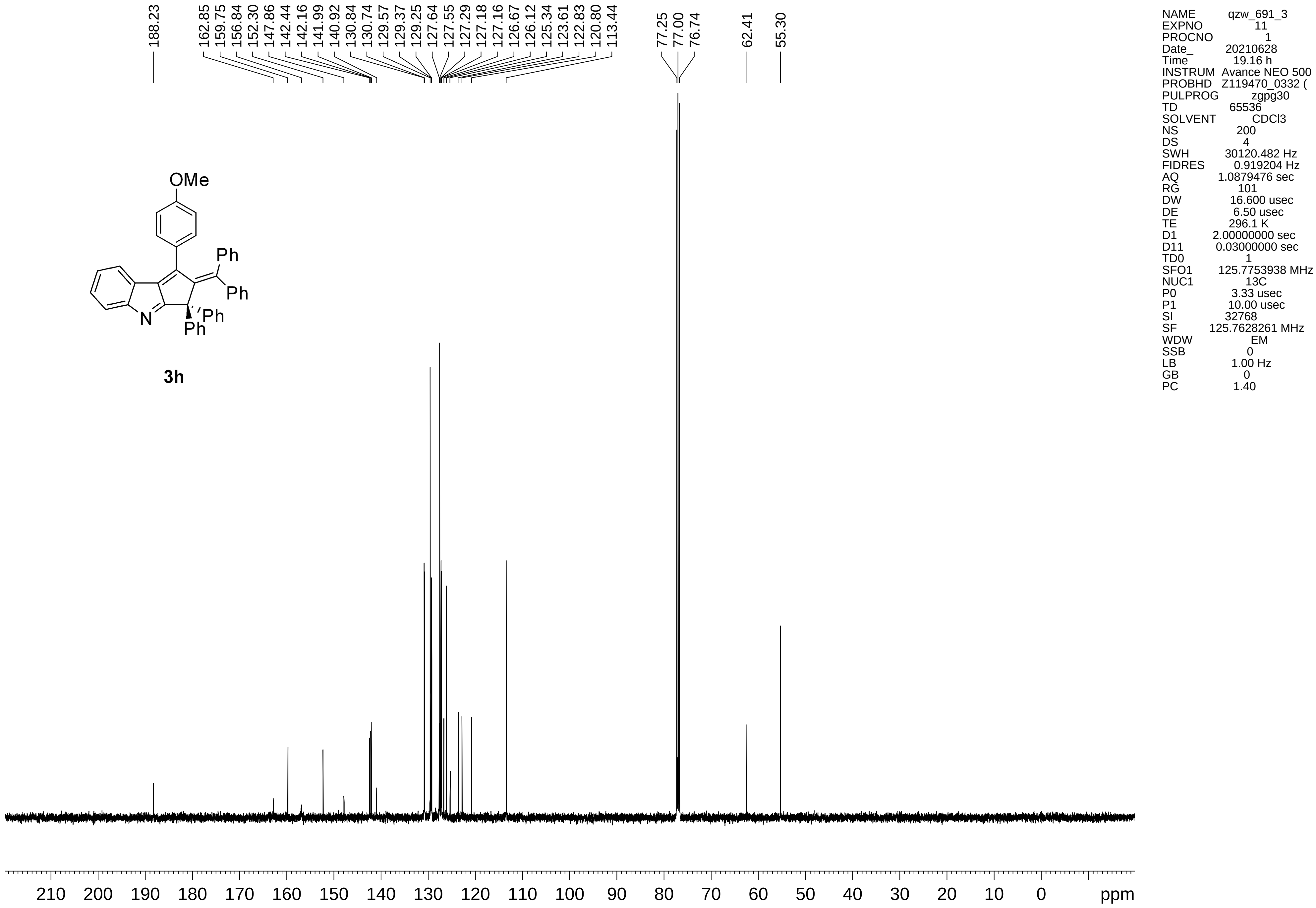
188.23
162.85
159.75
156.84
152.30
147.86
142.44
142.16
141.99
140.92
130.84
130.74
129.57
129.37
129.25
127.64
127.55
127.29
127.18
127.16
126.67
126.12
125.34
123.61
122.83
120.80
113.44
77.25
77.00
76.74
62.41
55.30
OMe
Ph
Ph
N
Ph
Ph
NAME qzw_691_3
EXPNO 11
PROCNO 1
Date_ 20210628
Time 19.16 h
INSTRUM Avance NEO 500
PROBHD Z119470_0332 (
PULPROG zgpg30
TD 65536
SOLVENT CDCl3
NS 200
DS 4
SWH 30120.482 Hz
FIDRES 0.919204 Hz
AQ 1.0879476 sec
RG 101
DW 16.600 usec
DE 6.50 usec
TE 296.1 K
D1 2.00000000 sec
D11 0.03000000 sec
TD0 1
SFO1 125.7753938 MHz
NUC1 13C
P0 3.33 usec
P1 10.00 usec
SI 32768
SF 125.7628261 MHz
WDW EM
SSB 0
LB 1.00 Hz
GB 0
PC 1.40
210 200 190 180 170 160 150 140 130 120 110 100 90 80 70 60 50 40 30 20 10 0 ppm

**3h**

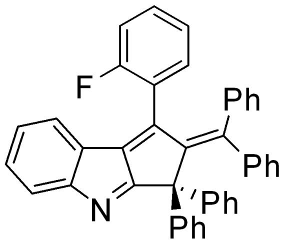

3i

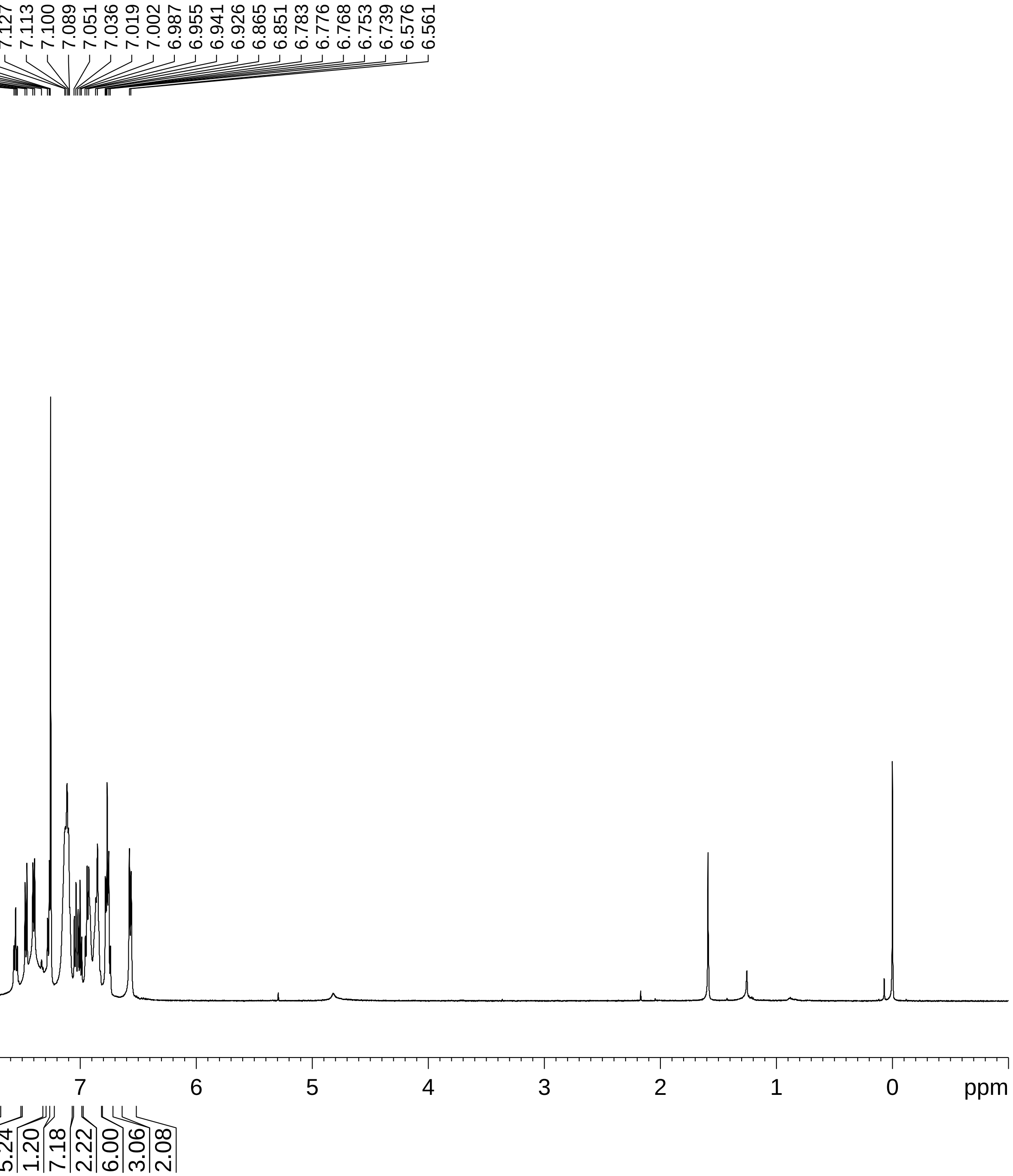


NAME qzw_694_5
EXPNO 31
PROCNO 1
Date_ 20210707
Time 14.20 h
INSTRUM Avance NEO 500
PROBHD Z119470_0332 (
PULPROG zg30
TD 65536
SOLVENT CDCl3
NS 4
DS 2
SWH 10000.000 Hz
FIDRES 0.305176 Hz
AQ 3.2768500 sec
RG 101
DW 50.000 usec
DE 10.84 usec
TE 296.2 K
D1 1.00000000 sec
TD0 1
SFO1 500.1530884 MHz
NUC1 1H
P0 3.24 usec
P1 9.72 usec
SI 65536
SF 500.1500144 MHz
WDW EM
SSB 0
LB 0.30 Hz
GB 0
PC 1.00

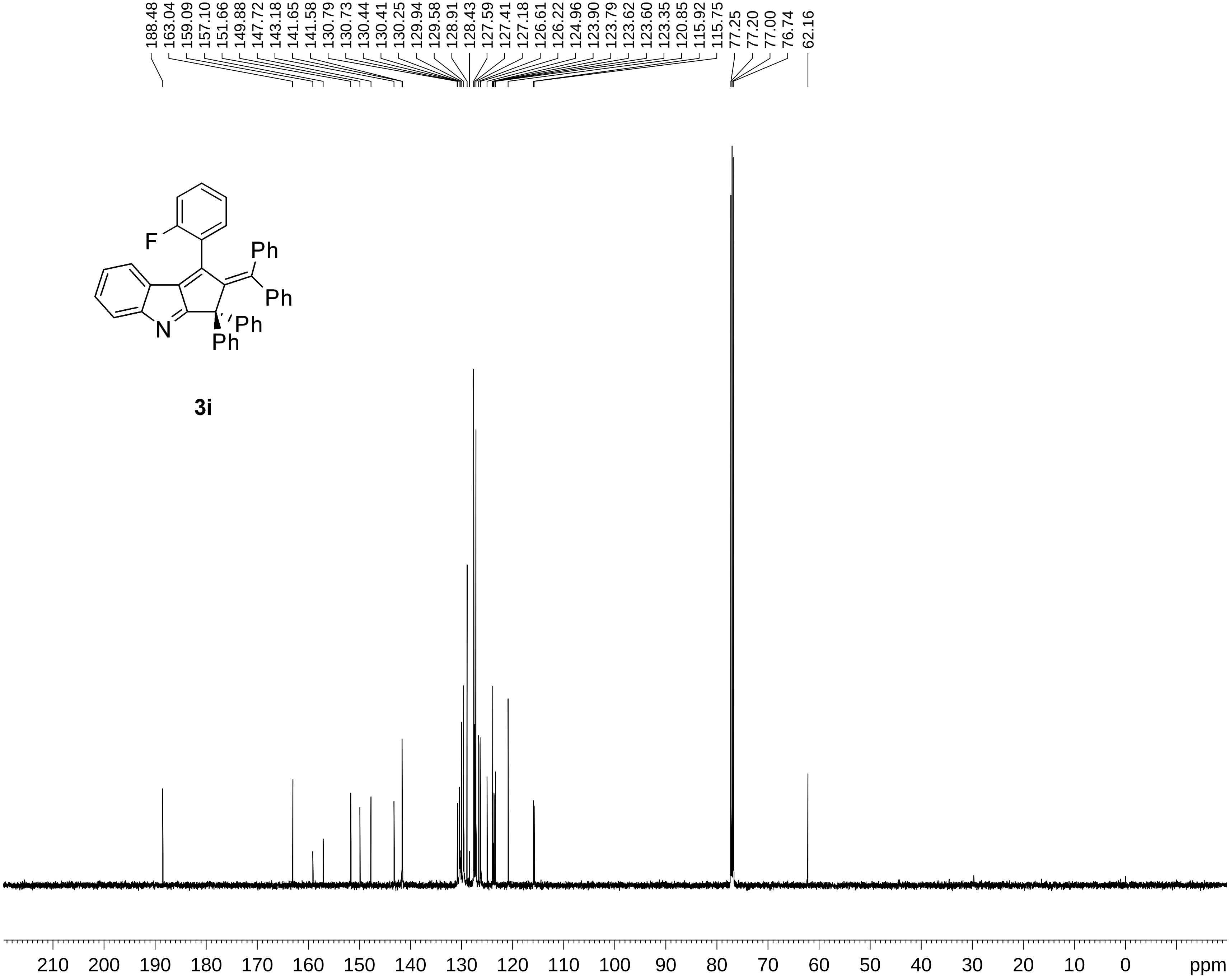


| | |
|---|---|
| NAME | qzw_694_5 |
| EXPNO | 11 |
| PROCNO | 1 |
| Date_ | 20210702 |
| Time | 20.16 h |
| INSTRUM | Avance NEO 500 |
| PROBHD | Z119470_0332 ( |
| PULPROG | zgpg30 |
| TD | 65536 |
| SOLVENT | CDCl3 |
| NS | 320 |
| DS | 4 |
| SWH | 30120.482 Hz |
| FIDRES | 0.919204 Hz |
| AQ | 1.0879476 sec |
| RG | 101 |
| DW | 16.600 usec |
| DE | 6.50 usec |
| TE | 296.1 K |
| D1 | 2.00000000 sec |
| D11 | 0.03000000 sec |
| TD0 | 1 |
| SFO1 | 125.7753938 MHz |
| NUC1 | 13C |
| P0 | 3.33 usec |
| P1 | 10.00 usec |
| SI | 32768 |
| SF | 125.7628279 MHz |
| WDW | EM |
| SSB | 0 |
| LB | 1.00 Hz |
| GB | 0 |
| PC | 1.40 |

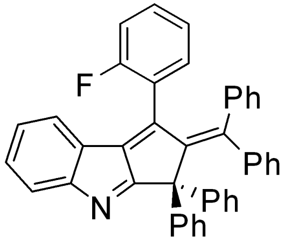


**3i**

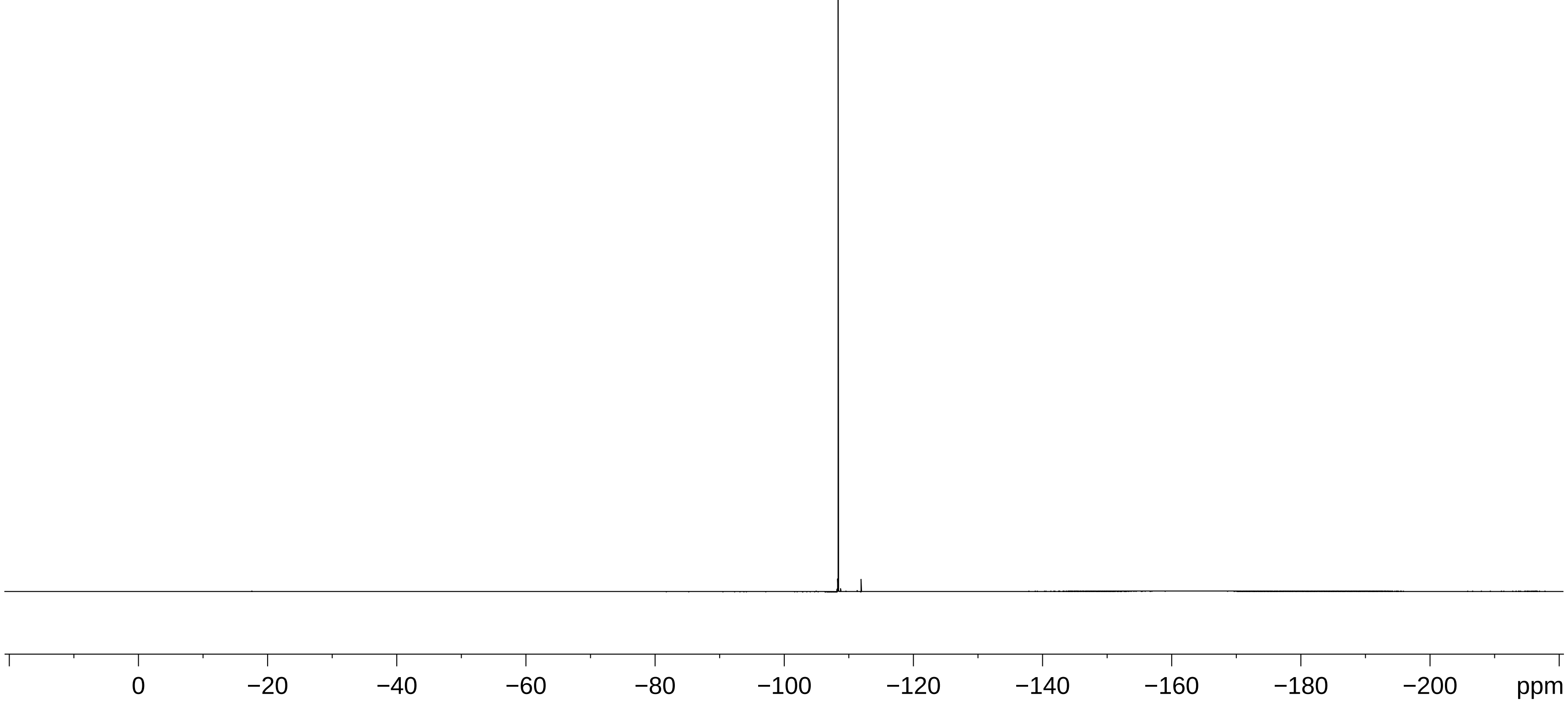


| | |
|---|---|
| NAME | qzw_694_5 |
| EXPNO | 12 |
| PROCNO | 1 |
| Date_ | 20210702 |
| Time | 20.18 h |
| INSTRUM | Avance NEO 500 |
| PROBHD | Z119470_0332 ( |
| PULPROG | zgig |
| TD | 131072 |
| SOLVENT | CDCl3 |
| NS | 16 |
| DS | 4 |
| SWH | 113636.367 Hz |
| FIDRES | 1.733953 Hz |
| AQ | 0.5767668 sec |
| RG | 101 |
| DW | 4.400 usec |
| DE | 6.50 usec |
| TE | 296.2 K |
| D1 | 1.00000000 sec |
| D11 | 0.03000000 sec |
| TD0 | 1 |
| SFO1 | 470.5641349 MHz |
| NUC1 | 19F |
| P1 | 15.00 usec |
| SI | 65536 |
| SF | 470.6111960 MHz |
| WDW | EM |
| SSB | 0 |
| LB | 0.30 Hz |
| GB | 0 |
| PC | 1.00 |

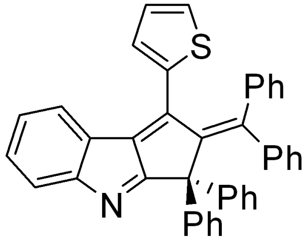

**3j**

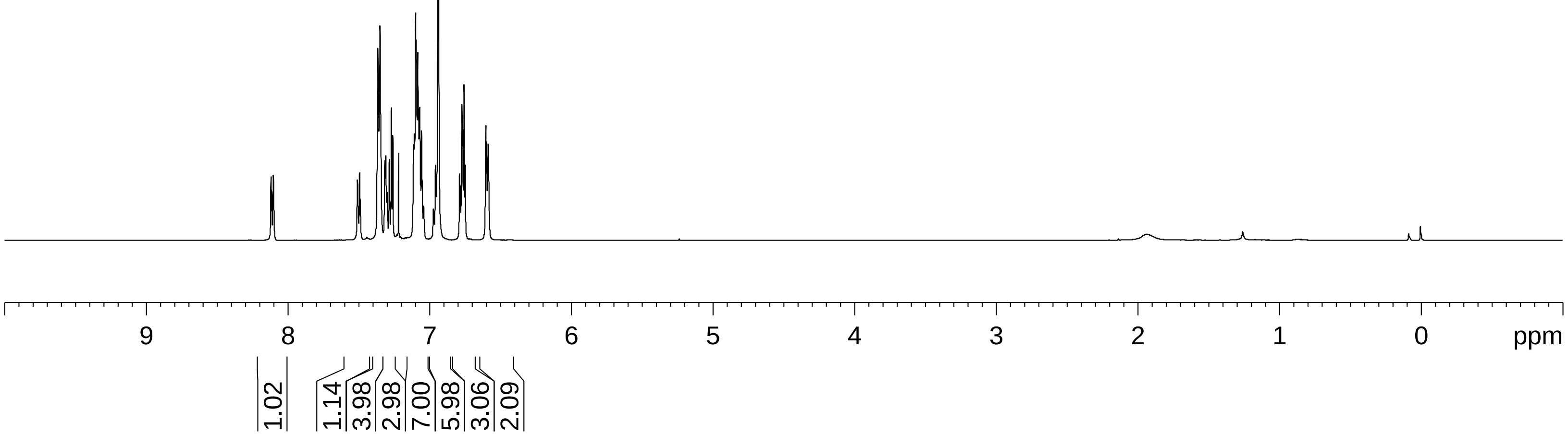


| | |
|---|---|
| NAME | qzw_692_4 |
| EXPNO | 10 |
| PROCNO | 1 |
| Date_ | 20210701 |
| Time | 4.58 h |
| INSTRUM | Avance NEO 500 |
| PROBHD | Z119470_0332 ( |
| PULPROG | zg30 |
| TD | 65536 |
| SOLVENT | CDCl3 |
| NS | 4 |
| DS | 2 |
| SWH | 10000.000 Hz |
| FIDRES | 0.305176 Hz |
| AQ | 3.2768500 sec |
| RG | 67.0968 |
| DW | 50.000 usec |
| DE | 10.84 usec |
| TE | 296.2 K |
| D1 | 1.00000000 sec |
| TD0 | 1 |
| SFO1 | 500.1530884 MHz |
| NUC1 | 1H |
| P0 | 3.24 usec |
| P1 | 9.72 usec |
| SI | 65536 |
| SF | 500.1500322 MHz |
| WDW | EM |
| SSB | 0 |
| LB | 0.30 Hz |
| GB | 0 |
| PC | 1.00 |

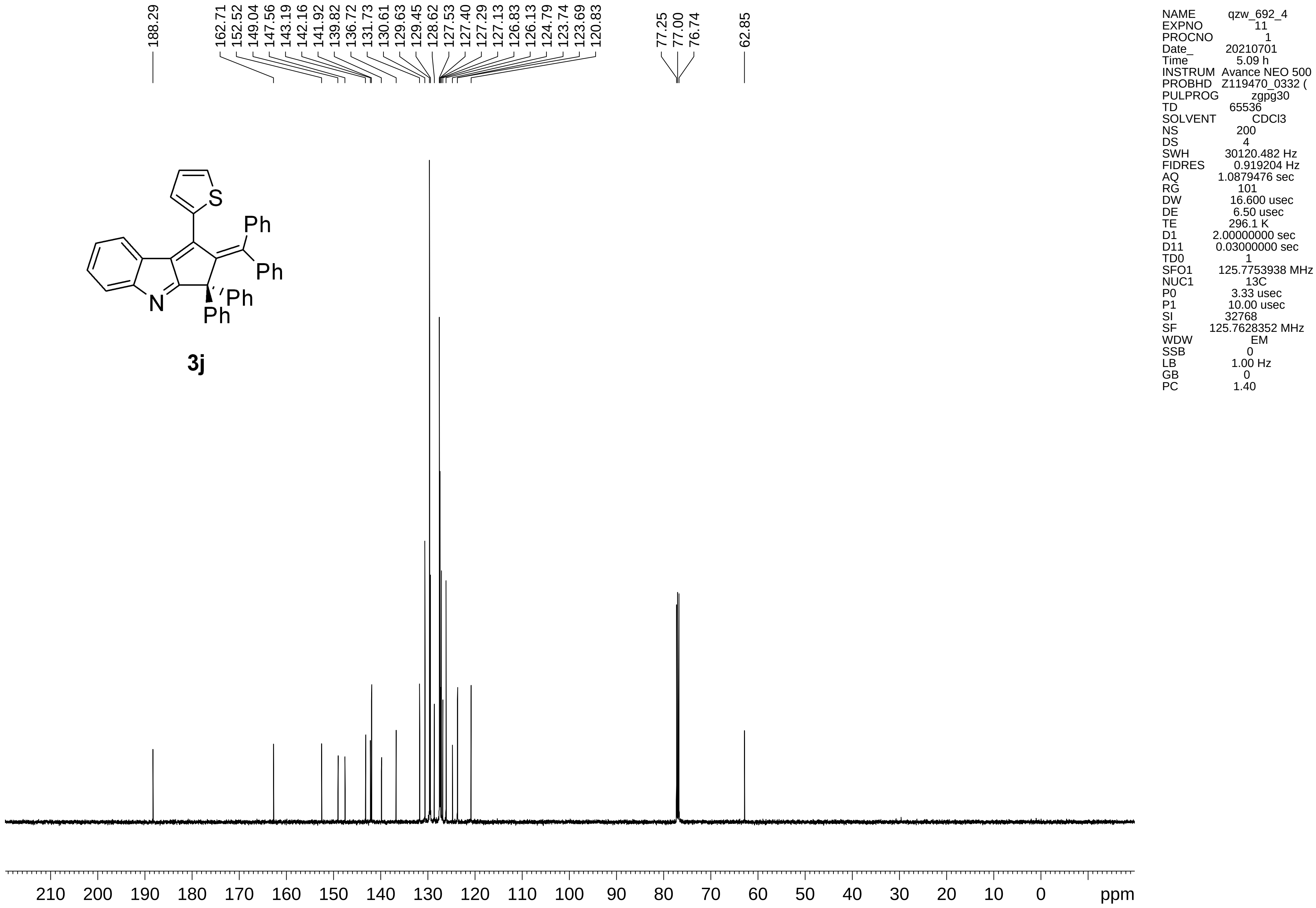

188.29
162.71
152.52
149.04
147.56
143.19
142.16
141.92
139.82
136.72
131.73
130.61
129.63
129.45
128.62
127.53
127.40
127.29
127.13
126.83
126.13
124.79
123.74
123.69
120.83
77.25
77.00
76.74
62.85
S
Ph
Ph
Ph
Ph
N
3j
NAME qzw_692_4
EXPNO 11
PROCNO 1
Date_ 20210701
Time 5.09 h
INSTRUM Avance NEO 500
PROBHD Z119470_0332 (
PULPROG zgpg30
TD 65536
SOLVENT CDCl3
NS 200
DS 4
SWH 30120.482 Hz
FIDRES 0.919204 Hz
AQ 1.0879476 sec
RG 101
DW 16.600 usec
DE 6.50 usec
TE 296.1 K
D1 2.00000000 sec
D11 0.03000000 sec
TD0 1
SFO1 125.7753938 MHz
NUC1 13C
P0 3.33 usec
P1 10.00 usec
SI 32768
SF 125.7628352 MHz
WDW EM
SSB 0
LB 1.00 Hz
GB 0
PC 1.40
210 200 190 180 170 160 150 140 130 120 110 100 90 80 70 60 50 40 30 20 10 0 ppm

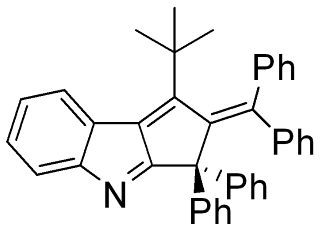


**3k**

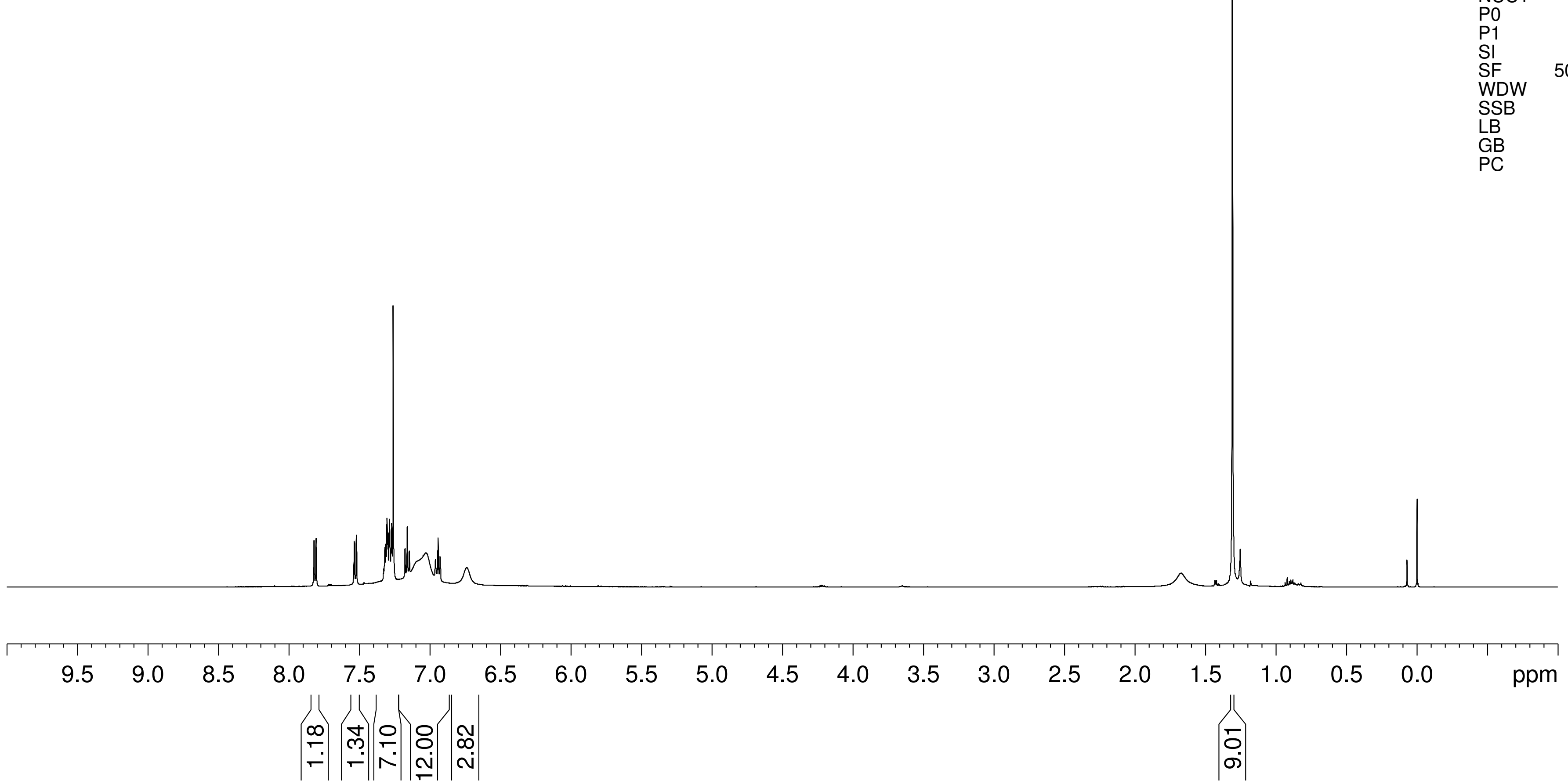


| | |
|---|---|
| NAME | m_wtf_883_1 |
| EXPNO | 10 |
| PROCNO | 1 |
| Date_ | 20240216 |
| Time | 18.24 h |
| INSTRUM | Avance NEO 500 |
| PROBHD | Z119470_0332 ( |
| PULPROG | zg30 |
| TD | 65536 |
| SOLVENT | CDCl3 |
| NS | 16 |
| DS | 2 |
| SWH | 10000.000 Hz |
| FIDRES | 0.305176 Hz |
| AQ | 3.2768500 sec |
| RG | 101 |
| DW | 50.000 usec |
| DE | 10.84 usec |
| TE | 294.7 K |
| D1 | 1.00000000 sec |
| TD0 | 1 |
| SFO1 | 500.1530884 MHz |
| NUC1 | 1H |
| P0 | 3.24 usec |
| P1 | 9.72 usec |
| SI | 65536 |
| SF | 500.1500131 MHz |
| WDW | EM |
| SSB | 0 |
| LB | 0.30 Hz |
| GB | 0 |
| PC | 1.00 |

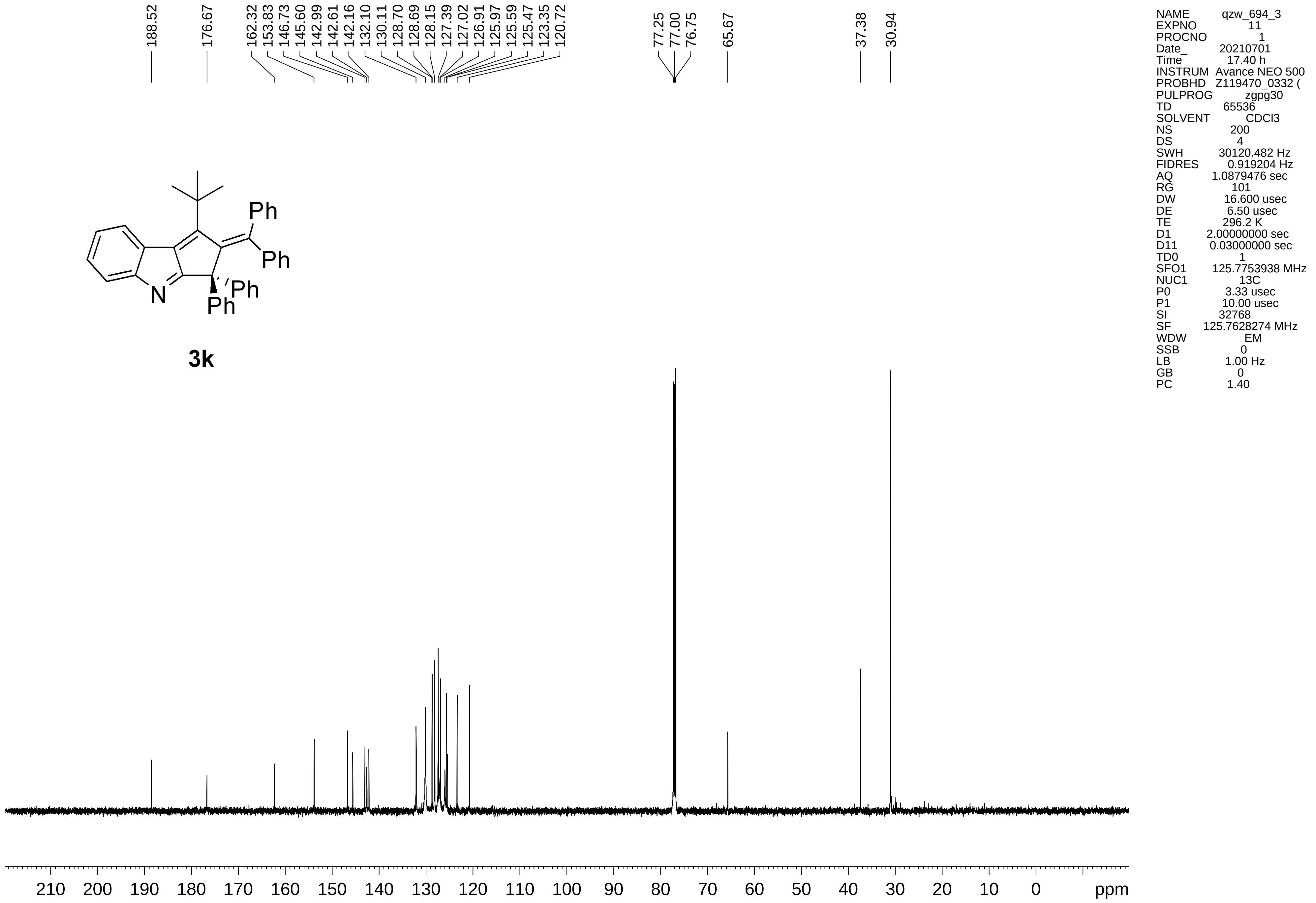
188.52
176.67
162.32
153.83
146.73
145.60
142.99
142.61
142.16
132.10
130.11
128.70
128.69
128.15
127.39
127.02
126.91
125.97
125.59
125.47
123.35
120.72
77.25
77.00
76.75
65.67
37.38
30.94
Ph
Ph
N
Ph
Ph
3k
210 200 190 180 170 160 150 140 130 120 110 100 90 80 70 60 50 40 30 20 10 0 ppm
NAME qzw_694_3
EXPNO 11
PROCNO 1
Date_ 20210701
Time 17.40 h
INSTRUM Avance NEO 500
PROBHD Z119470_0332 (
PULPROG zgpg30
TD 65536
SOLVENT CDCl3
NS 200
DS 4
SWH 30120.482 Hz
FIDRES 0.919204 Hz
AQ 1.0879476 sec
RG 101
DW 16.600 usec
DE 6.50 usec
TE 296.2 K
D1 2.00000000 sec
D11 0.03000000 sec
TD0 1
SFO1 125.7753938 MHz
NUC1 13C
P0 3.33 usec
P1 10.00 usec
SI 32768
SF 125.7628274 MHz
WDW EM
SSB 0
LB 1.00 Hz
GB 0
PC 1.40

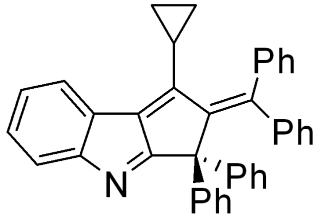

**3l**

NAME m_wtf_883_2
EXPNO 30
PROCNO 1
Date_ 20240217
Time 17.03 h
INSTRUM Avance NEO 500
PROBHD Z119470_0332 (
PULPROG zg30
TD 65536
SOLVENT CDCl3
NS 16
DS 2
SWH 10000.000 Hz
FIDRES 0.305176 Hz
AQ 3.2768500 sec
RG 101
DW 50.000 usec
DE 10.84 usec
TE 295.4 K
D1 1.00000000 sec
TD0 1
SFO1 500.1530884 MHz
NUC1 1H
P0 3.24 usec
P1 9.72 usec
SI 65536
SF 500.1500157 MHz
WDW EM
SSB 0
LB 0.30 Hz
GB 0
PC 1.00

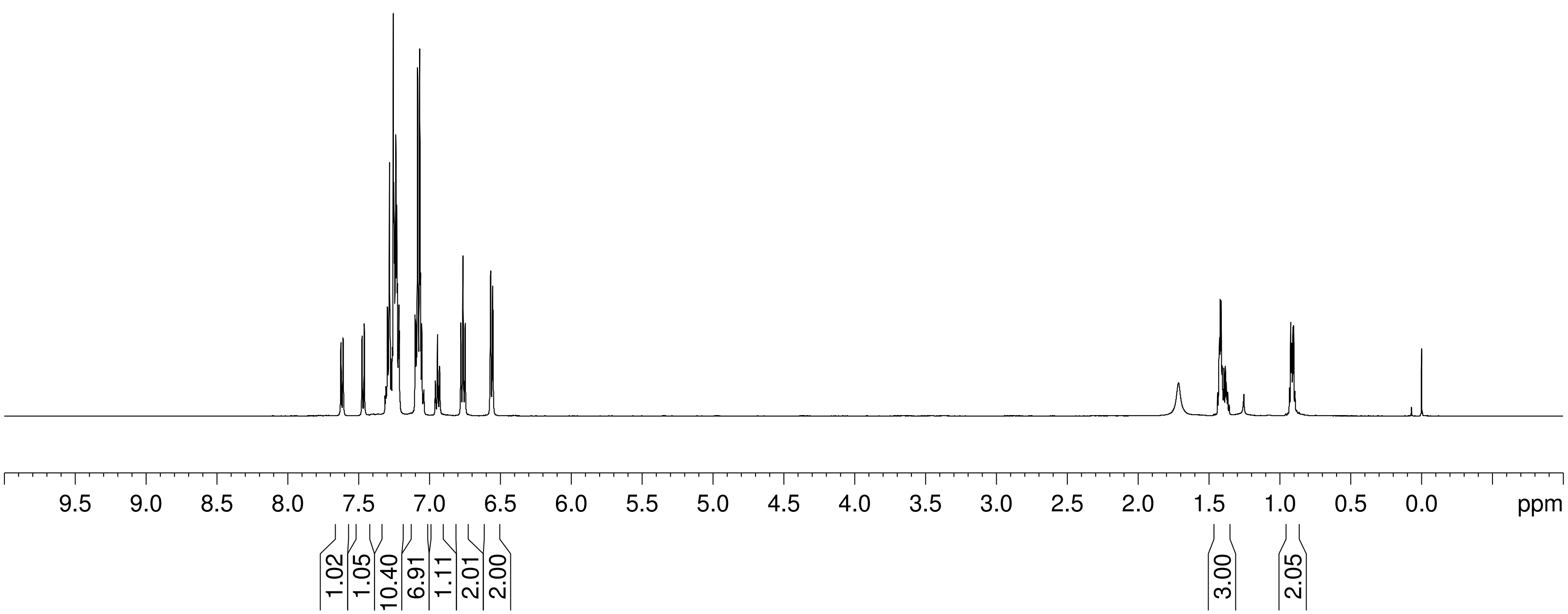

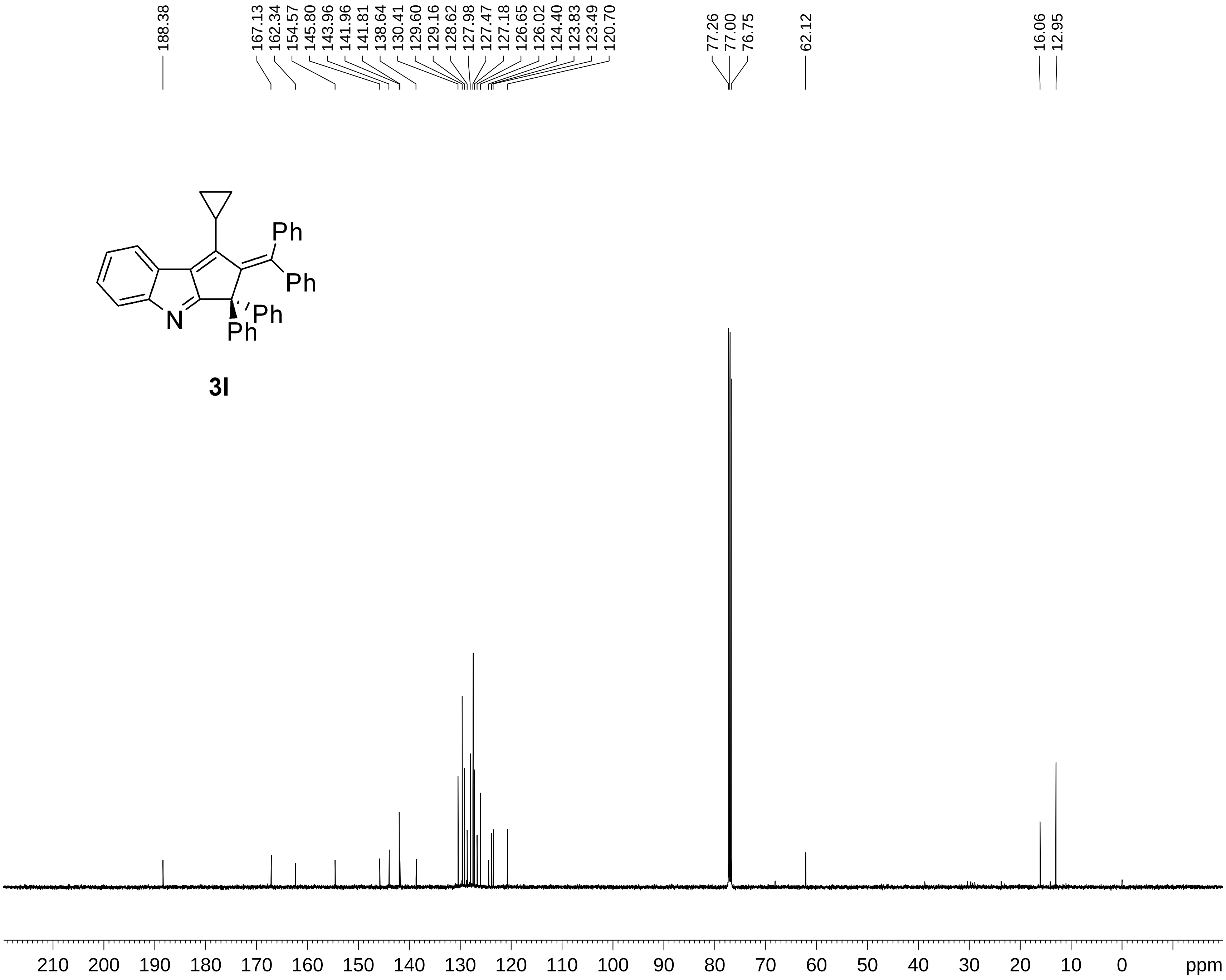


**3l**

| | |
|---|---|
| NAME | qzw_691_4 |
| EXPNO | 11 |
| PROCNO | 1 |
| Date_ | 20210630 |
| Time | 23.54 h |
| INSTRUM | Avance NEO 500 |
| PROBHD | Z119470_0332 ( |
| PULPROG | zgpg30 |
| TD | 65536 |
| SOLVENT | CDCl3 |
| NS | 600 |
| DS | 4 |
| SWH | 30120.482 Hz |
| FIDRES | 0.919204 Hz |
| AQ | 1.0879476 sec |
| RG | 101 |
| DW | 16.600 usec |
| DE | 6.50 usec |
| TE | 296.1 K |
| D1 | 2.00000000 sec |
| D11 | 0.03000000 sec |
| TD0 | 1 |
| SFO1 | 125.7753938 MHz |
| NUC1 | 13C |
| P0 | 3.33 usec |
| P1 | 10.00 usec |
| SI | 32768 |
| SF | 125.7628233 MHz |
| WDW | EM |
| SSB | 0 |
| LB | 1.00 Hz |
| GB | 0 |
| PC | 1.40 |

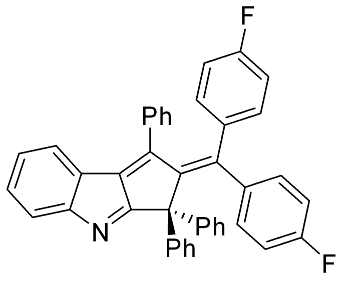

**3m**

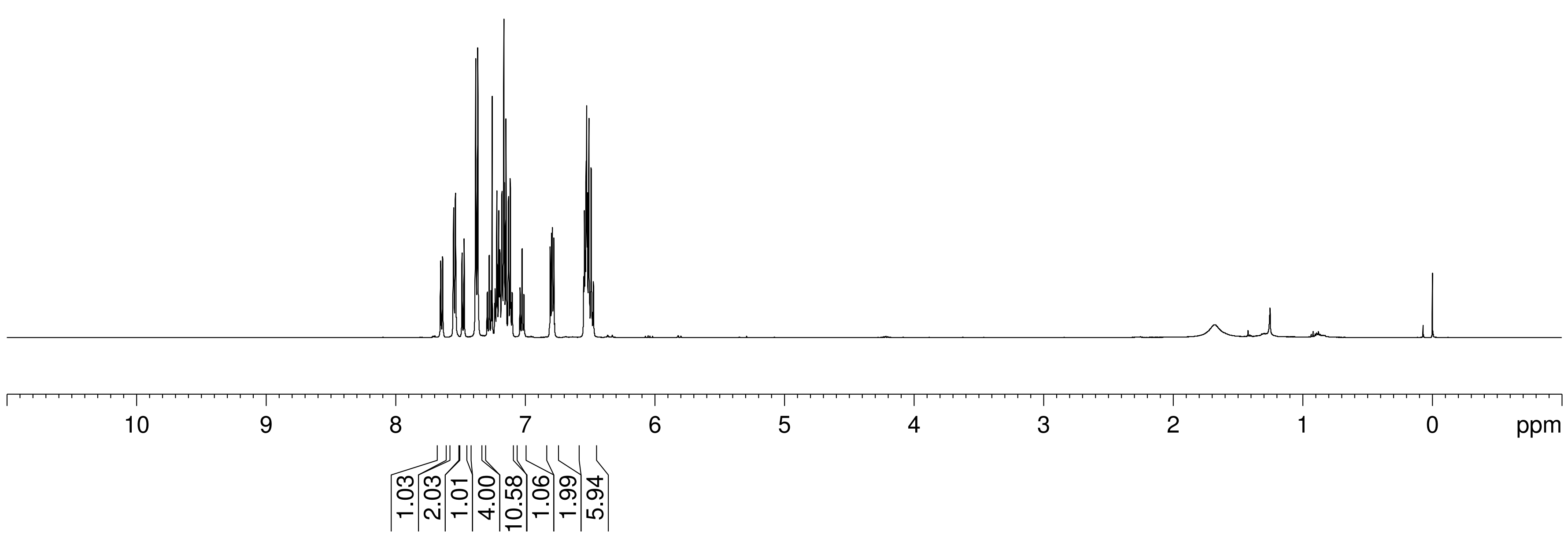


NAME m_qzw_695_1
EXPNO 20
PROCNO 1
Date_ 20240215
Time 18.54 h
INSTRUM Avance NEO 500
PROBHD Z119470_0332 (
PULPROG zg30
TD 65536
SOLVENT CDCl3
NS 16
DS 2
SWH 10000.000 Hz
FIDRES 0.305176 Hz
AQ 3.2768500 sec
RG 101
DW 50.000 usec
DE 10.84 usec
TE 294.7 K
D1 1.00000000 sec
TD0 1
SFO1 500.1530884 MHz
NUC1 1H
P0 3.24 usec
P1 9.72 usec
SI 65536
SF 500.1500141 MHz
WDW EM
SSB 0
LB 0.30 Hz
GB 0
PC 1.00

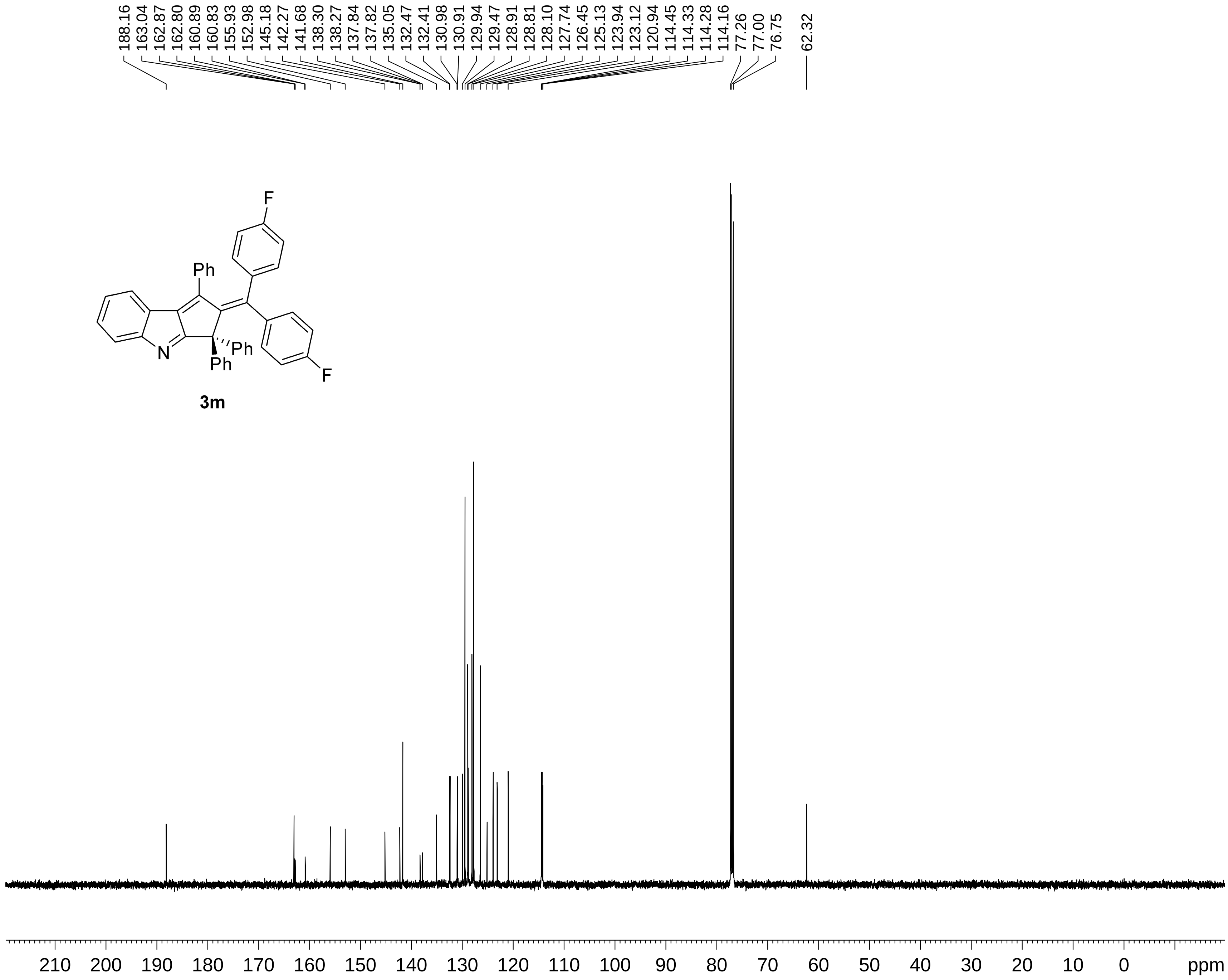


| | |
|---|---|
| NAME | qzw_695_1 |
| EXPNO | 11 |
| PROCNO | 1 |
| Date_ | 20210704 |
| Time | 0.39 h |
| INSTRUM | Avance NEO 500 |
| PROBHD | Z119470_0332 ( |
| PULPROG | zgpg30 |
| TD | 65536 |
| SOLVENT | CDCl3 |
| NS | 320 |
| DS | 4 |
| SWH | 30120.482 Hz |
| FIDRES | 0.919204 Hz |
| AQ | 1.0879476 sec |
| RG | 101 |
| DW | 16.600 usec |
| DE | 6.50 usec |
| TE | 296.1 K |
| D1 | 2.00000000 sec |
| D11 | 0.03000000 sec |
| TD0 | 1 |
| SFO1 | 125.7753938 MHz |
| NUC1 | 13C |
| P0 | 3.33 usec |
| P1 | 10.00 usec |
| SI | 32768 |
| SF | 125.7628243 MHz |
| WDW | EM |
| SSB | 0 |
| LB | 1.00 Hz |
| GB | 0 |
| PC | 1.40 |

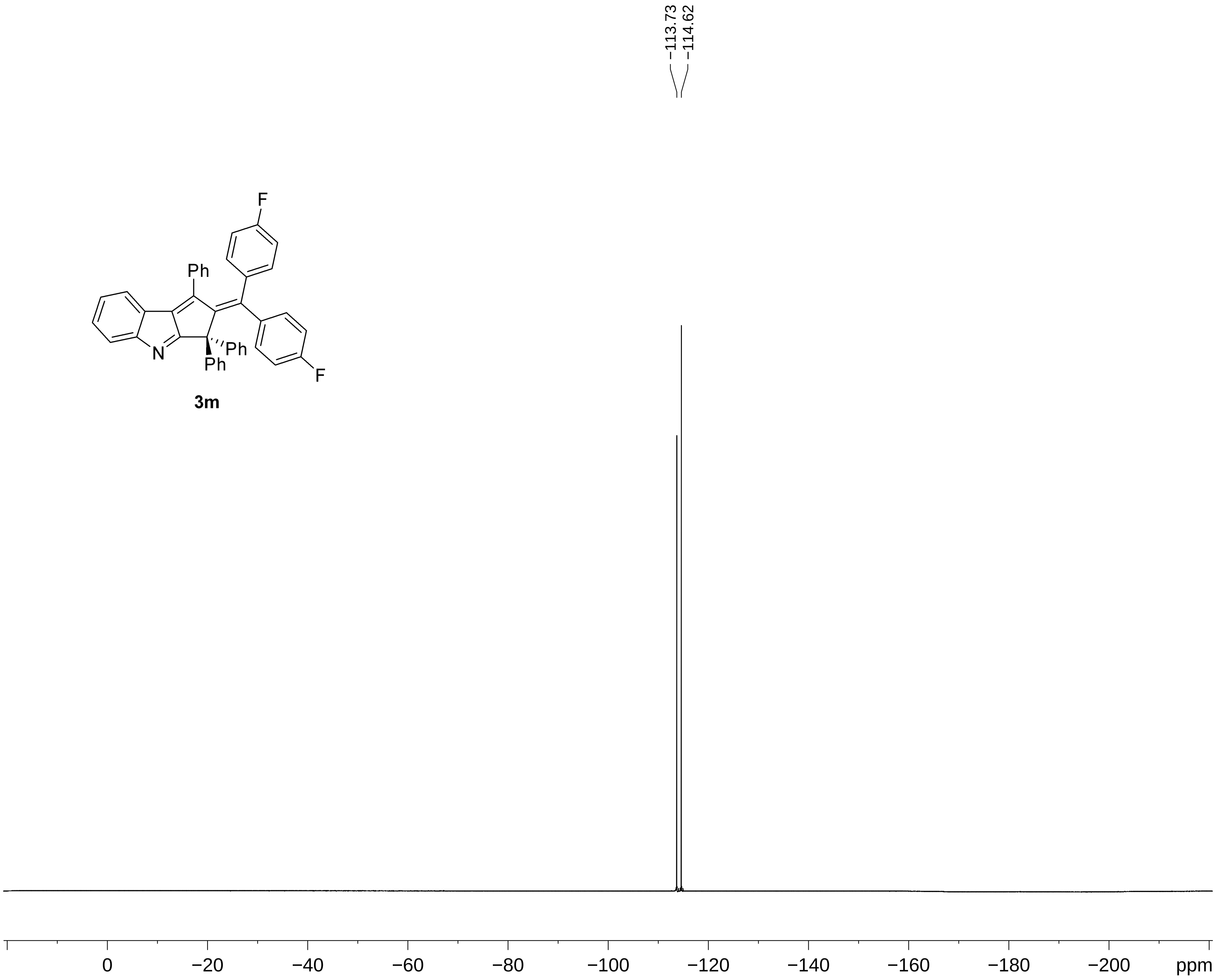


NAME qzw_695_1
EXPNO 12
PROCNO 1
Date_ 20210704
Time 0.50 h
INSTRUM Avance NEO 500
PROBHD Z119470_0332 (
PULPROG zgig
TD 131072
SOLVENT CDCl3
NS 16
DS 4
SWH 113636.367 Hz
FIDRES 1.733953 Hz
AQ 0.5767668 sec
RG 101
DW 4.400 usec
DE 6.50 usec
TE 296.2 K
D1 1.00000000 sec
D11 0.03000000 sec
TD0 1
SFO1 470.5641349 MHz
NUC1 19F
P1 15.00 usec
SI 65536
SF 470.6111960 MHz
WDW EM
SSB 0
LB 0.30 Hz
GB 0
PC 1.00

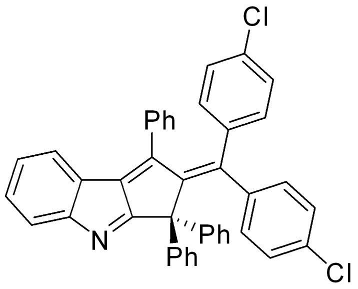

**3n**

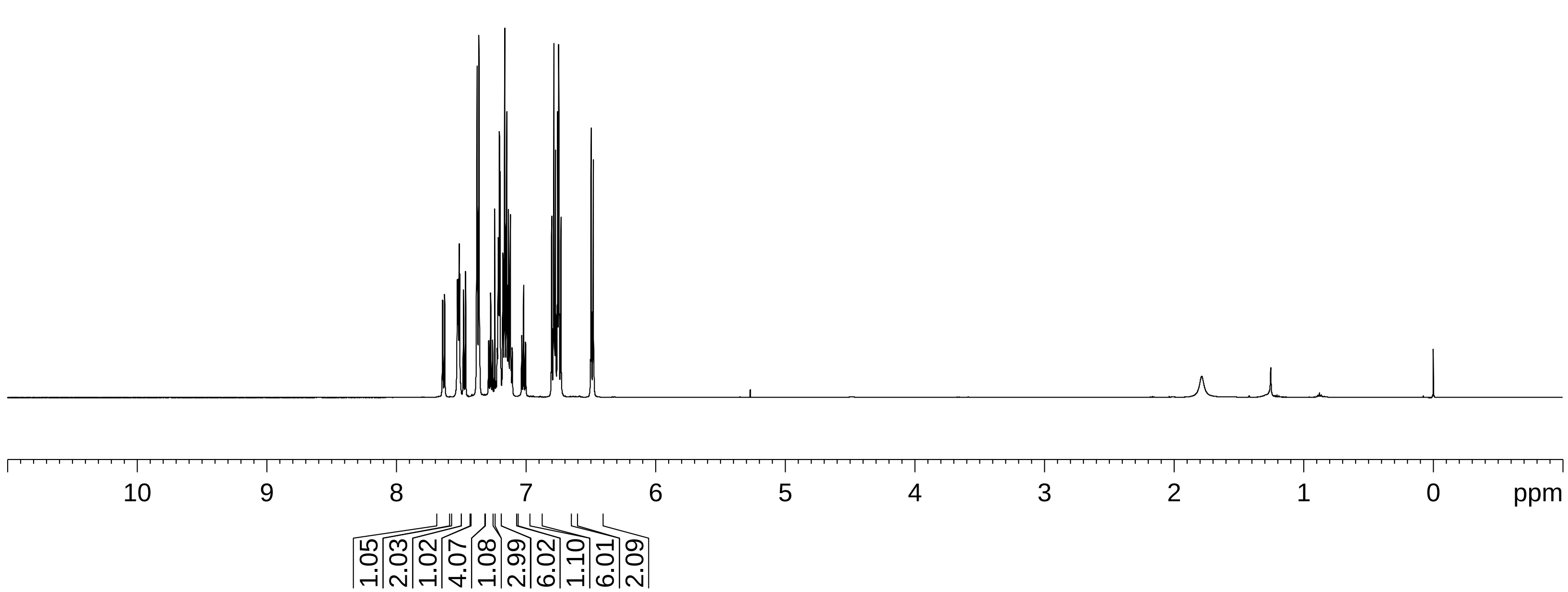


NAME qzw_695_2
EXPNO 10
PROCNO 1
Date_ 20210704
Time 0.54 h
INSTRUM Avance NEO 500
PROBHD Z119470_0332 (
PULPROG zg30
TD 65536
SOLVENT CDCl3
NS 16
DS 2
SWH 10000.000 Hz
FIDRES 0.305176 Hz
AQ 3.2768500 sec
RG 101
DW 50.000 usec
DE 10.84 usec
TE 296.2 K
D1 1.00000000 sec
TD0 1
SFO1 500.1530884 MHz
NUC1 1H
P0 3.24 usec
P1 9.72 usec
SI 65536
SF 500.1500209 MHz
WDW EM
SSB 0
LB 0.30 Hz
GB 0
PC 1.00

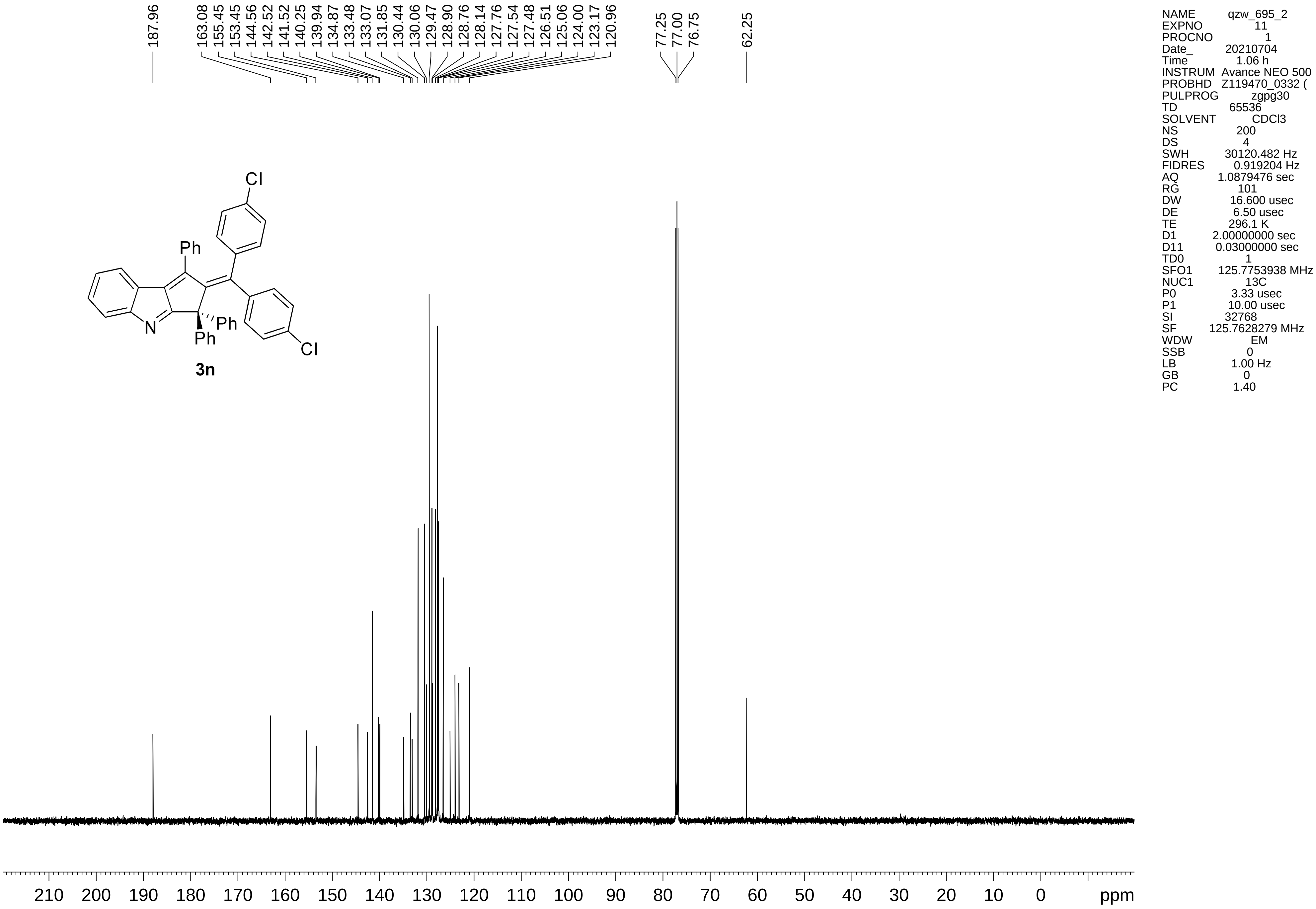
187.96
163.08
155.45
153.45
144.56
142.52
141.52
140.25
139.94
134.87
133.48
133.07
131.85
130.44
130.06
129.47
128.90
128.76
128.14
127.76
127.54
127.48
126.51
125.06
124.00
123.17
120.96
77.25
77.00
76.75
62.25
Cl
Ph
Ph
Ph
N
Cl
3n
NAME qzw_695_2
EXPNO 11
PROCNO 1
Date_ 20210704
Time 1.06 h
INSTRUM Avance NEO 500
PROBHD Z119470_0332 (
PULPROG zgpg30
TD 65536
SOLVENT CDCl3
NS 200
DS 4
SWH 30120.482 Hz
FIDRES 0.919204 Hz
AQ 1.0879476 sec
RG 101
DW 16.600 usec
DE 6.50 usec
TE 296.1 K
D1 2.00000000 sec
D11 0.03000000 sec
TD0 1
SFO1 125.7753938 MHz
NUC1 13C
P0 3.33 usec
P1 10.00 usec
SI 32768
SF 125.7628279 MHz
WDW EM
SSB 0
LB 1.00 Hz
GB 0
PC 1.40
210 200 190 180 170 160 150 140 130 120 110 100 90 80 70 60 50 40 30 20 10 0 ppm

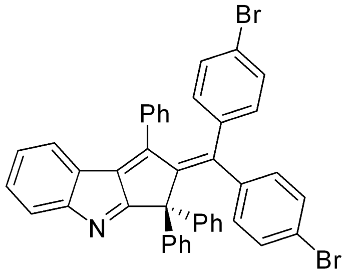


**3o**

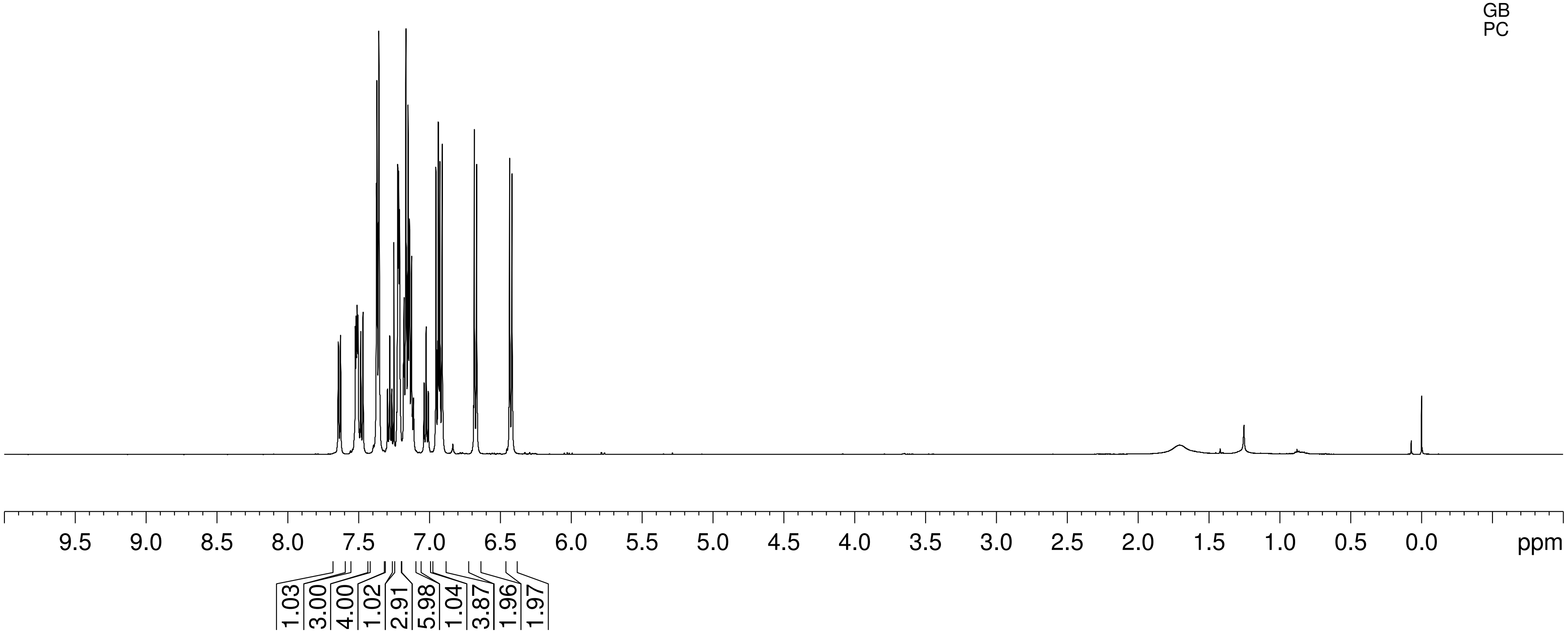


| | |
|---|---|
| NAME | m_qzw_695_3 |
| EXPNO | 10 |
| PROCNO | 1 |
| Date_ | 20240214 |
| Time | 19.08 h |
| INSTRUM | Avance NEO 500 |
| PROBHD | Z119470_0332 ( |
| PULPROG | zg30 |
| TD | 65536 |
| SOLVENT | CDCl3 |
| NS | 8 |
| DS | 2 |
| SWH | 10000.000 Hz |
| FIDRES | 0.305176 Hz |
| AQ | 3.2768500 sec |
| RG | 101 |
| DW | 50.000 usec |
| DE | 10.84 usec |
| TE | 294.9 K |
| D1 | 1.00000000 sec |
| TD0 | 1 |
| SFO1 | 500.1530884 MHz |
| NUC1 | 1H |
| P0 | 3.24 usec |
| P1 | 9.72 usec |
| SI | 65536 |
| SF | 500.1500162 MHz |
| WDW | EM |
| SSB | 0 |
| LB | 0.30 Hz |
| GB | 0 |
| PC | 1.00 |

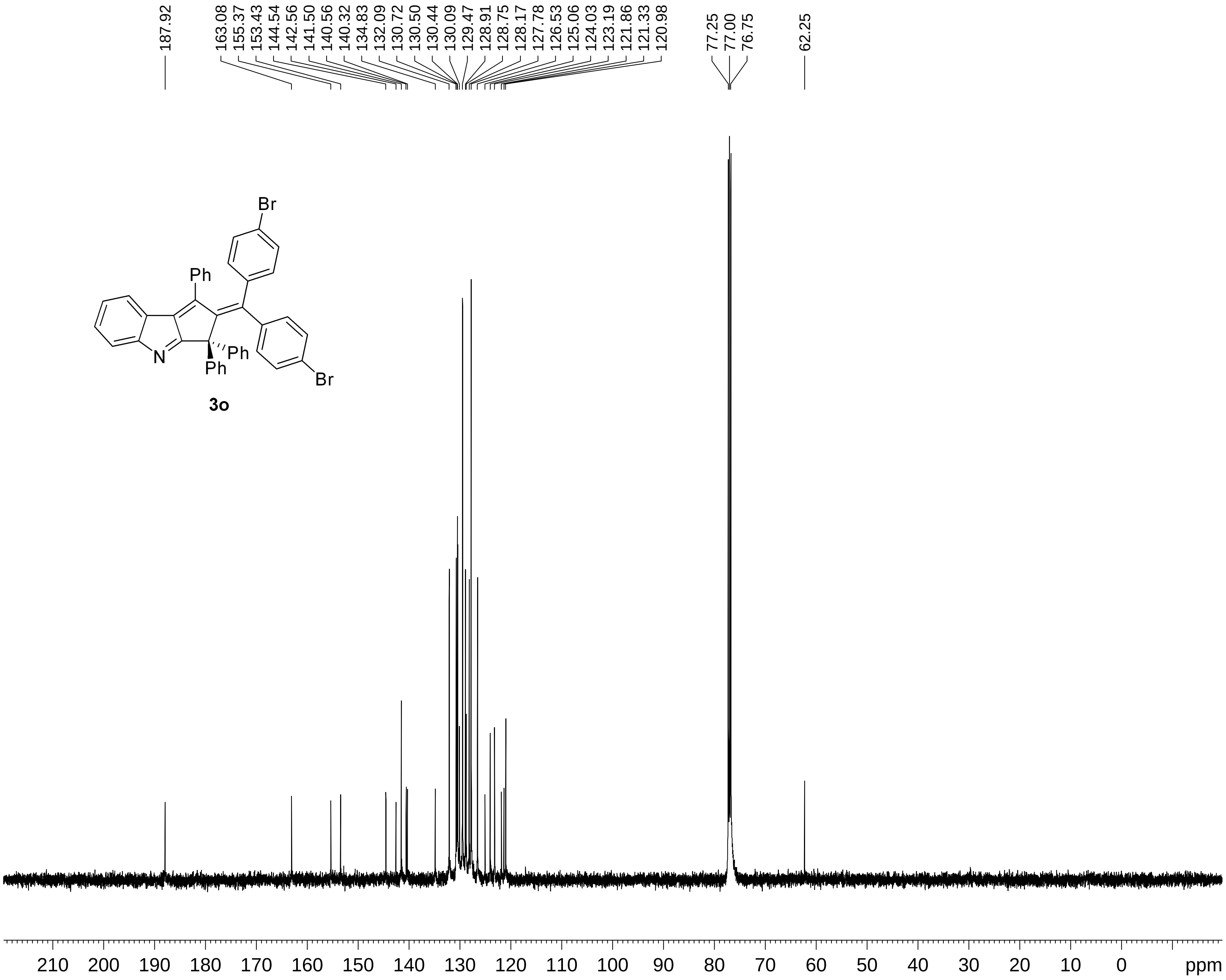


**3o**

| | |
|---|---|
| NAME | qzw_695_3 |
| EXPNO | 11 |
| PROCNO | 1 |
| Date_ | 20210704 |
| Time | 1.33 h |
| INSTRUM | Avance NEO 500 |
| PROBHD | Z119470_0332 ( |
| PULPROG | zgpg30 |
| TD | 65536 |
| SOLVENT | CDCl3 |
| NS | 400 |
| DS | 4 |
| SWH | 30120.482 Hz |
| FIDRES | 0.919204 Hz |
| AQ | 1.0879476 sec |
| RG | 101 |
| DW | 16.600 usec |
| DE | 6.50 usec |
| TE | 296.2 K |
| D1 | 2.00000000 sec |
| D11 | 0.03000000 sec |
| TD0 | 1 |
| SFO1 | 125.7753938 MHz |
| NUC1 | 13C |
| P0 | 3.33 usec |
| P1 | 10.00 usec |
| SI | 32768 |
| SF | 125.7628270 MHz |
| WDW | EM |
| SSB | 0 |
| LB | 1.00 Hz |
| GB | 0 |
| PC | 1.40 |

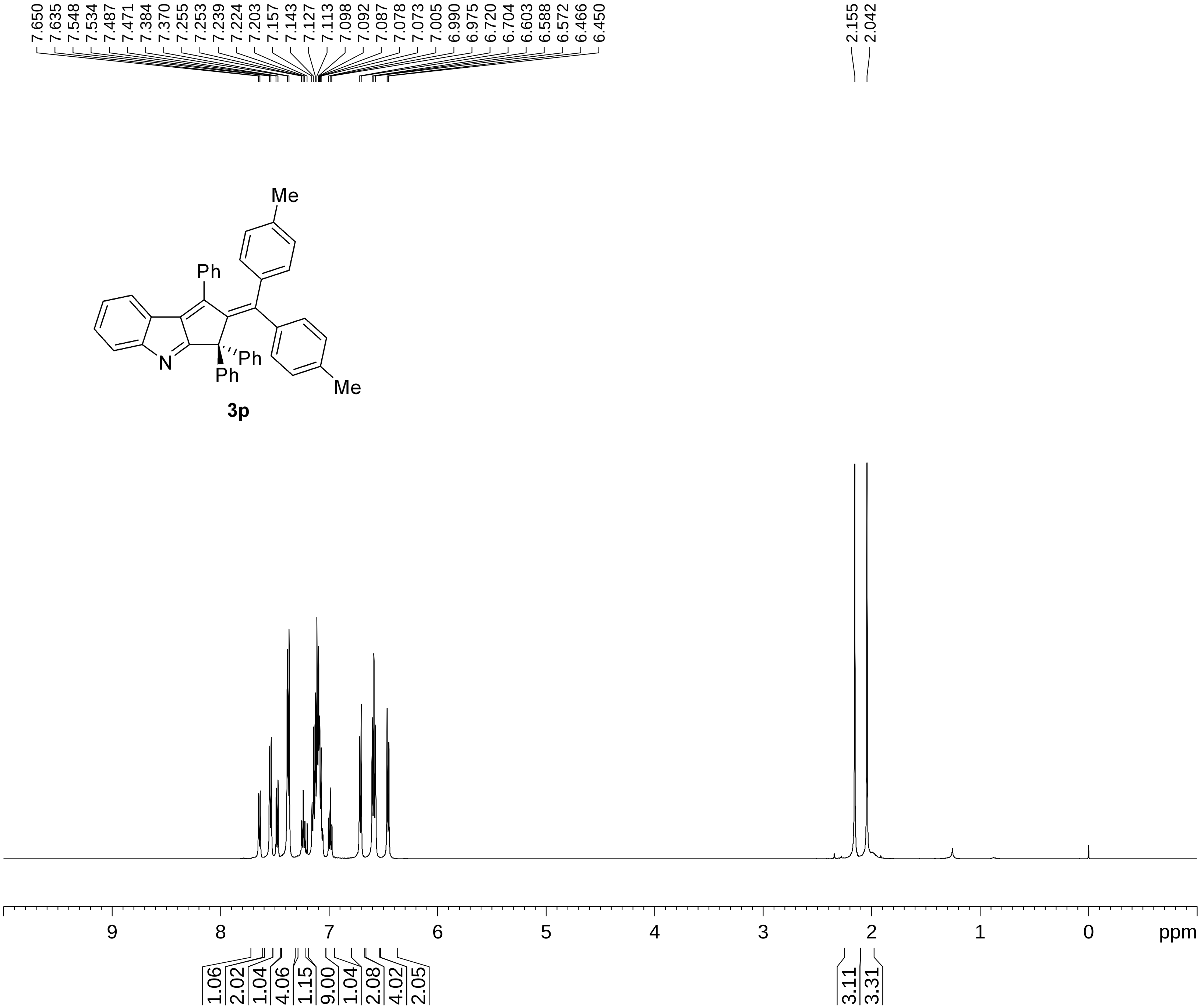


**3p**

| | |
|---|---|
| NAME | qzw_695_4 |
| EXPNO | 22 |
| PROCNO | 1 |
| Date_ | 20210704 |
| Time | 19.07 h |
| INSTRUM | Avance NEO 500 |
| PROBHD | Z119470_0332 ( |
| PULPROG | zg30 |
| TD | 65536 |
| SOLVENT | CDCl3 |
| NS | 2 |
| DS | 2 |
| SWH | 10000.000 Hz |
| FIDRES | 0.305176 Hz |
| AQ | 3.2768500 sec |
| RG | 53.3333 |
| DW | 50.000 usec |
| DE | 10.84 usec |
| TE | 296.1 K |
| D1 | 1.00000000 sec |
| TD0 | 1 |
| SFO1 | 500.1530884 MHz |
| NUC1 | 1H |
| P0 | 3.24 usec |
| P1 | 9.72 usec |
| SI | 65536 |
| SF | 500.1500409 MHz |
| WDW | EM |
| SSB | 0 |
| LB | 0.30 Hz |
| GB | 0 |
| PC | 1.00 |

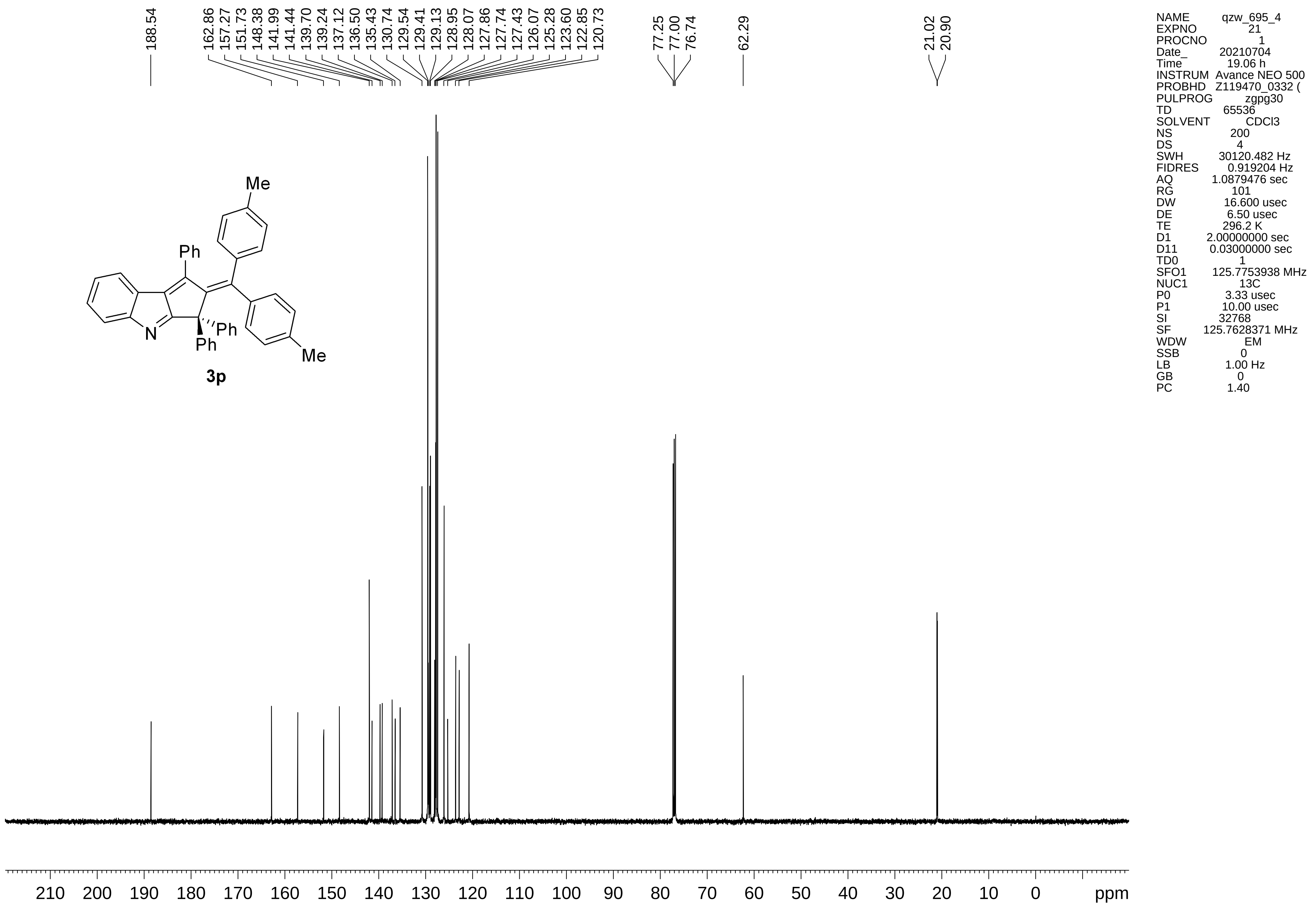
188.54
162.86
157.27
151.73
148.38
141.99
141.44
139.70
139.24
137.12
136.50
135.43
130.74
129.54
129.41
129.13
128.95
128.07
127.86
127.74
127.43
126.07
125.28
123.60
122.85
120.73
77.25
77.00
76.74
62.29
21.02
20.90
Me
Ph
N
Ph
Ph
Me
3p
NAME qzw_695_4
EXPNO 21
PROCNO 1
Date_ 20210704
Time 19.06 h
INSTRUM Avance NEO 500
PROBHD Z119470_0332 (
PULPROG zgpg30
TD 65536
SOLVENT CDCl3
NS 200
DS 4
SWH 30120.482 Hz
FIDRES 0.919204 Hz
AQ 1.0879476 sec
RG 101
DW 16.600 usec
DE 6.50 usec
TE 296.2 K
D1 2.00000000 sec
D11 0.03000000 sec
TD0 1
SFO1 125.7753938 MHz
NUC1 13C
P0 3.33 usec
P1 10.00 usec
SI 32768
SF 125.7628371 MHz
WDW EM
SSB 0
LB 1.00 Hz
GB 0
PC 1.40
210 200 190 180 170 160 150 140 130 120 110 100 90 80 70 60 50 40 30 20 10 0 ppm

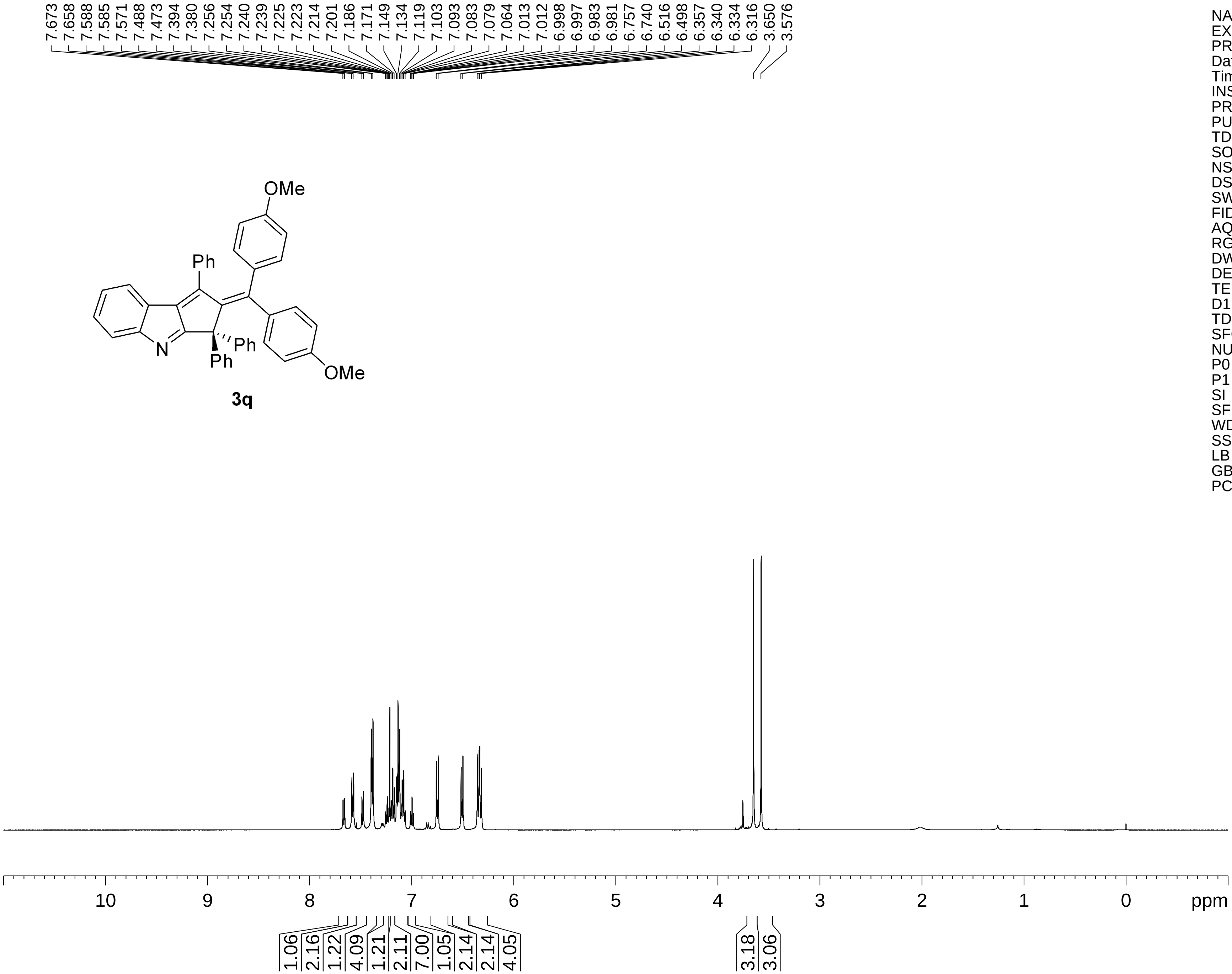


| | |
|---|---|
| NAME | qzw_695_5 |
| EXPNO | 10 |
| PROCNO | 1 |
| Date_ | 20210704 |
| Time | 19.10 h |
| INSTRUM | Avance NEO 500 |
| PROBHD | Z119470_0332 ( |
| PULPROG | zg30 |
| TD | 65536 |
| SOLVENT | CDCl3 |
| NS | 4 |
| DS | 2 |
| SWH | 10000.000 Hz |
| FIDRES | 0.305176 Hz |
| AQ | 3.2768500 sec |
| RG | 50.7317 |
| DW | 50.000 usec |
| DE | 10.84 usec |
| TE | 296.2 K |
| D1 | 1.00000000 sec |
| TD0 | 1 |
| SFO1 | 500.1530884 MHz |
| NUC1 | 1H |
| P0 | 3.24 usec |
| P1 | 9.72 usec |
| SI | 65536 |
| SF | 500.1500349 MHz |
| WDW | EM |
| SSB | 0 |
| LB | 0.30 Hz |
| GB | 0 |
| PC | 1.00 |

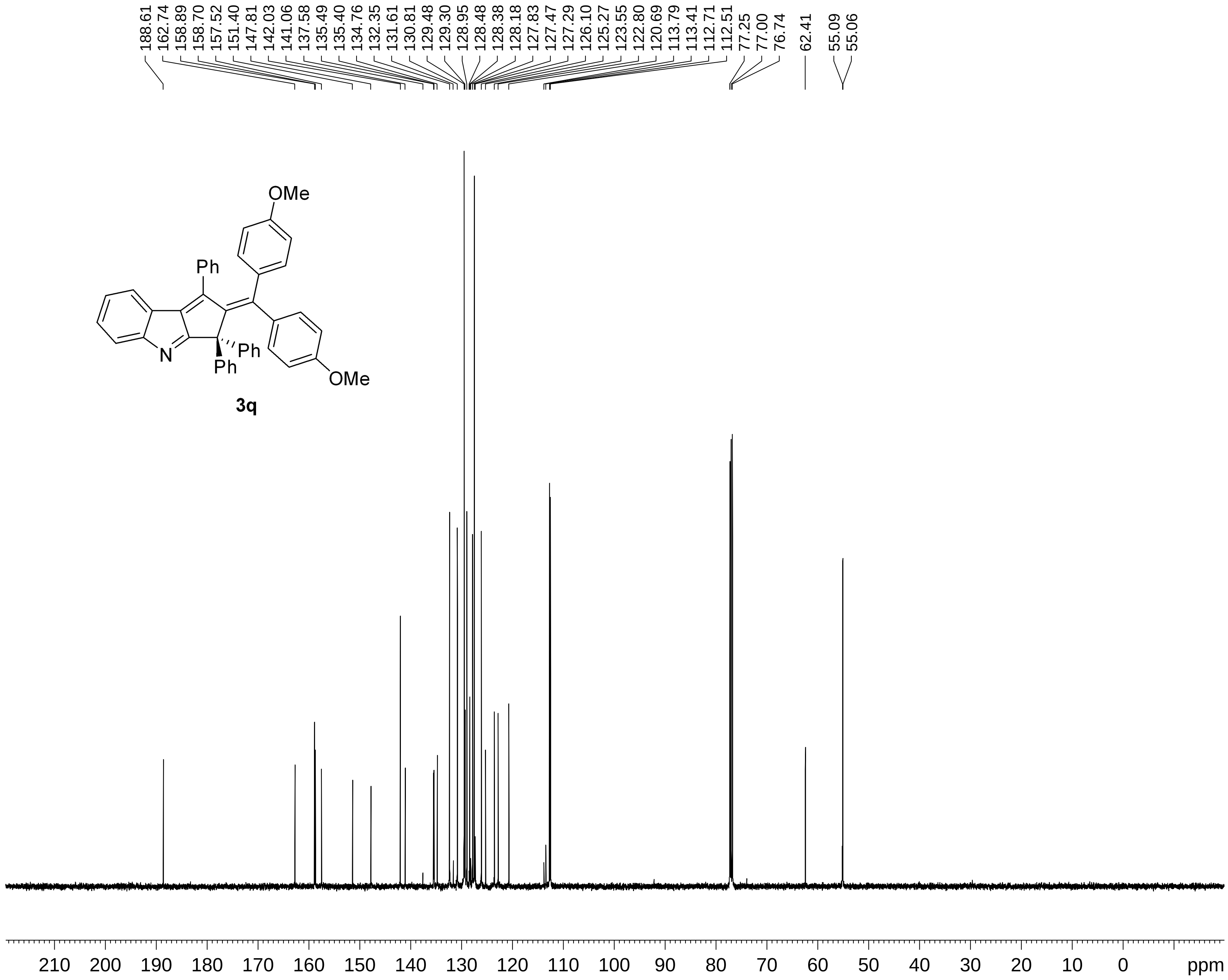


| | |
|---|---|
| NAME | qzw_695_5 |
| EXPNO | 11 |
| PROCNO | 1 |
| Date_ | 20210704 |
| Time | 19.21 h |
| INSTRUM | Avance NEO 500 |
| PROBHD | Z119470_0332 ( |
| PULPROG | zgpg30 |
| TD | 65536 |
| SOLVENT | CDCl3 |
| NS | 200 |
| DS | 4 |
| SWH | 30120.482 Hz |
| FIDRES | 0.919204 Hz |
| AQ | 1.0879476 sec |
| RG | 101 |
| DW | 16.600 usec |
| DE | 6.50 usec |
| TE | 296.1 K |
| D1 | 2.00000000 sec |
| D11 | 0.03000000 sec |
| TD0 | 1 |
| SFO1 | 125.7753938 MHz |
| NUC1 | 13C |
| P0 | 3.33 usec |
| P1 | 10.00 usec |
| SI | 32768 |
| SF | 125.7628380 MHz |
| WDW | EM |
| SSB | 0 |
| LB | 1.00 Hz |
| GB | 0 |
| PC | 1.40 |

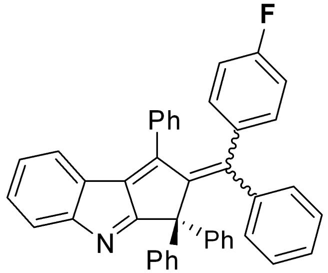


**3r**

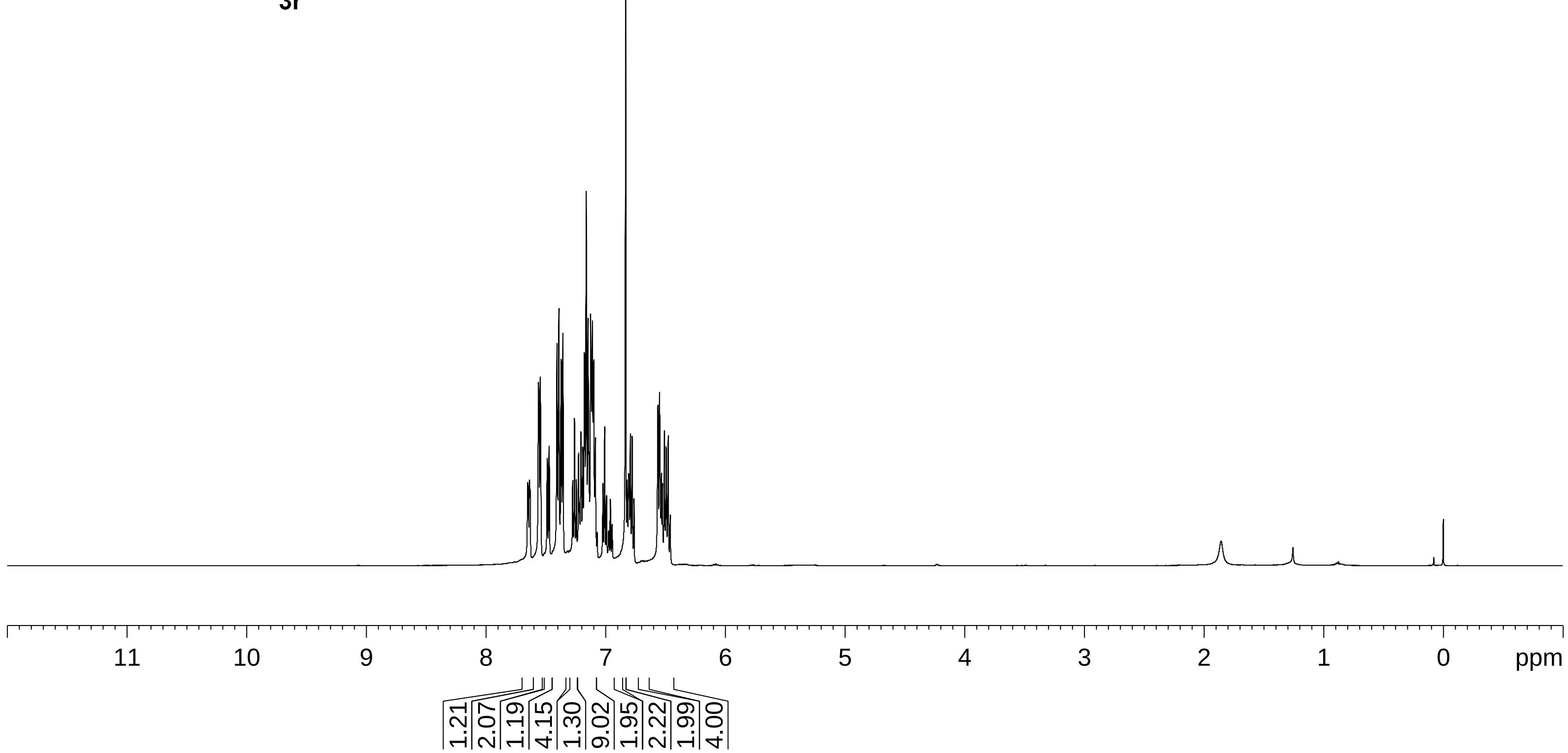


| | |
|---|---|
| NAME | qzw_688_2 |
| EXPNO | 10 |
| PROCNO | 1 |
| Date_ | 20210626 |
| Time | 20.33 h |
| INSTRUM | Avance NEO 500 |
| PROBHD | Z119470_0332 ( |
| PULPROG | zg30 |
| TD | 65536 |
| SOLVENT | CDCl3 |
| NS | 4 |
| DS | 2 |
| SWH | 10000.000 Hz |
| FIDRES | 0.305176 Hz |
| AQ | 3.2768500 sec |
| RG | 74.2857 |
| DW | 50.000 usec |
| DE | 10.84 usec |
| TE | 296.1 K |
| D1 | 1.00000000 sec |
| TD0 | 1 |
| SFO1 | 500.1530884 MHz |
| NUC1 | 1H |
| P0 | 3.24 usec |
| P1 | 9.72 usec |
| SI | 65536 |
| SF | 500.1500285 MHz |
| WDW | EM |
| SSB | 0 |
| LB | 0.30 Hz |
| GB | 0 |
| PC | 1.00 |

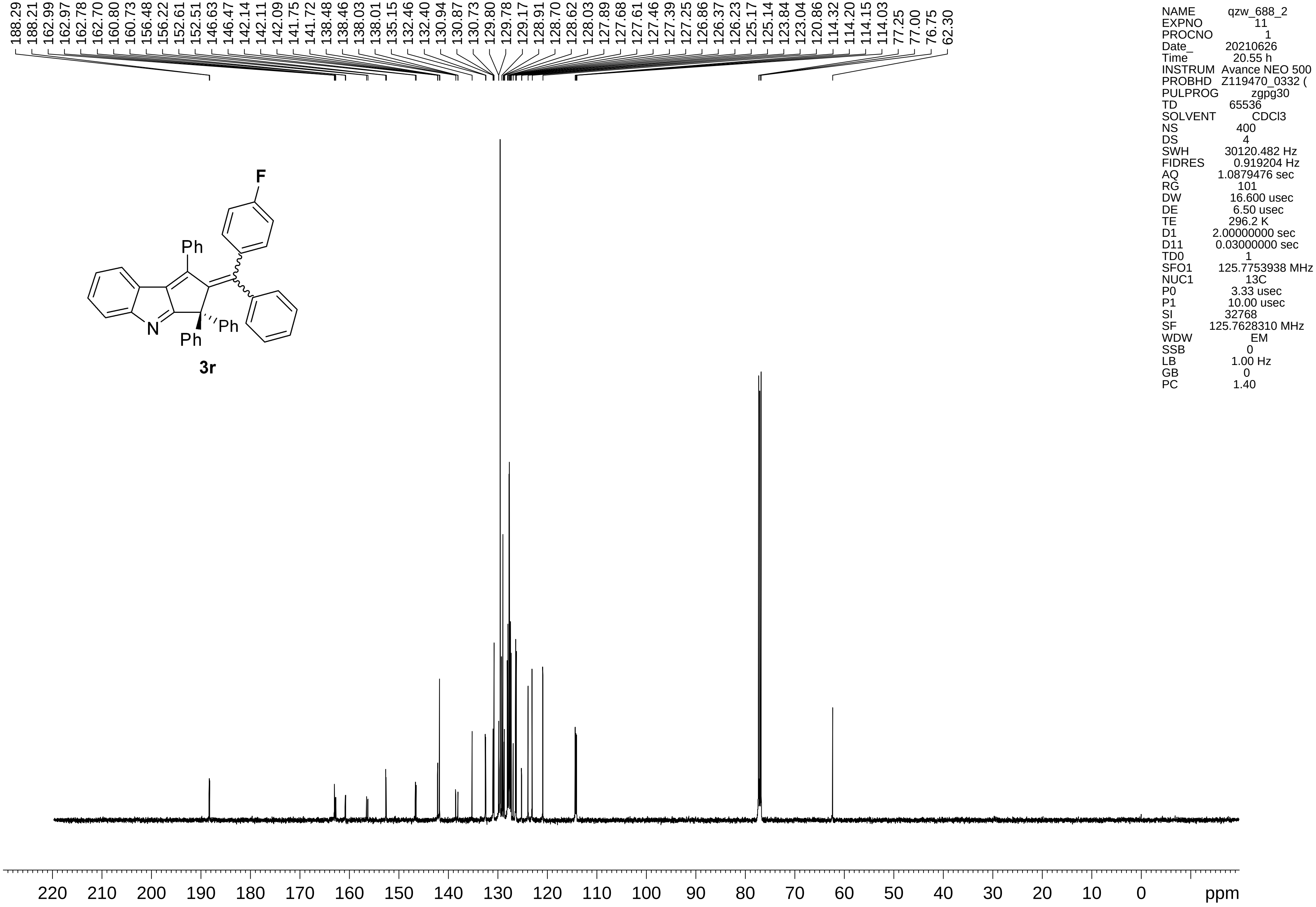
188.29
188.21
162.99
162.97
162.78
162.70
160.80
160.73
156.48
156.22
152.61
152.51
146.63
146.47
142.14
142.11
142.09
141.75
141.72
138.48
138.46
138.03
138.01
135.15
132.46
132.40
130.94
130.87
130.73
129.80
129.78
129.17
128.91
128.70
128.62
128.03
127.89
127.68
127.61
127.46
127.39
127.25
126.86
126.37
126.23
125.17
125.14
123.84
123.04
120.86
114.32
114.20
114.15
114.03
77.25
77.00
76.75
62.30
NAME qzw_688_2
EXPNO 11
PROCNO 1
Date_ 20210626
Time 20.55 h
INSTRUM Avance NEO 500
PROBHD Z119470_0332 (
PULPROG zgpg30
TD 65536
SOLVENT CDCl3
NS 400
DS 4
SWH 30120.482 Hz
FIDRES 0.919204 Hz
AQ 1.0879476 sec
RG 101
DW 16.600 usec
DE 6.50 usec
TE 296.2 K
D1 2.00000000 sec
D11 0.03000000 sec
TD0 1
SFO1 125.7753938 MHz
NUC1 13C
P0 3.33 usec
P1 10.00 usec
SI 32768
SF 125.7628310 MHz
WDW EM
SSB 0
LB 1.00 Hz
GB 0
PC 1.40
F
Ph
N
Ph
Ph
3r
220 210 200 190 180 170 160 150 140 130 120 110 100 90 80 70 60 50 40 30 20 10 0 ppm

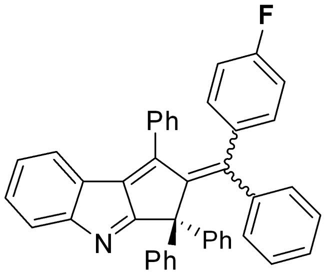


**3r**

−113.99
−114.90

0 −20 −40 −60 −80 −100 −120 −140 −160 −180 −200 ppm

| | |
|---|---|
| NAME | qzw_688_2 |
| EXPNO | 12 |
| PROCNO | 1 |
| Date_ | 20210626 |
| Time | 20.56 h |
| INSTRUM | Avance NEO 500 |
| PROBHD | Z119470_0332 ( |
| PULPROG | zgig |
| TD | 131072 |
| SOLVENT | CDCl3 |
| NS | 4 |
| DS | 4 |
| SWH | 113636.367 Hz |
| FIDRES | 1.733953 Hz |
| AQ | 0.5767668 sec |
| RG | 101 |
| DW | 4.400 usec |
| DE | 6.50 usec |
| TE | 296.2 K |
| D1 | 1.00000000 sec |
| D11 | 0.03000000 sec |
| TD0 | 1 |
| SFO1 | 470.5641349 MHz |
| NUC1 | 19F |
| P1 | 15.00 usec |
| SI | 65536 |
| SF | 470.6111960 MHz |
| WDW | EM |
| SSB | 0 |
| LB | 0.30 Hz |
| GB | 0 |
| PC | 1.00 |

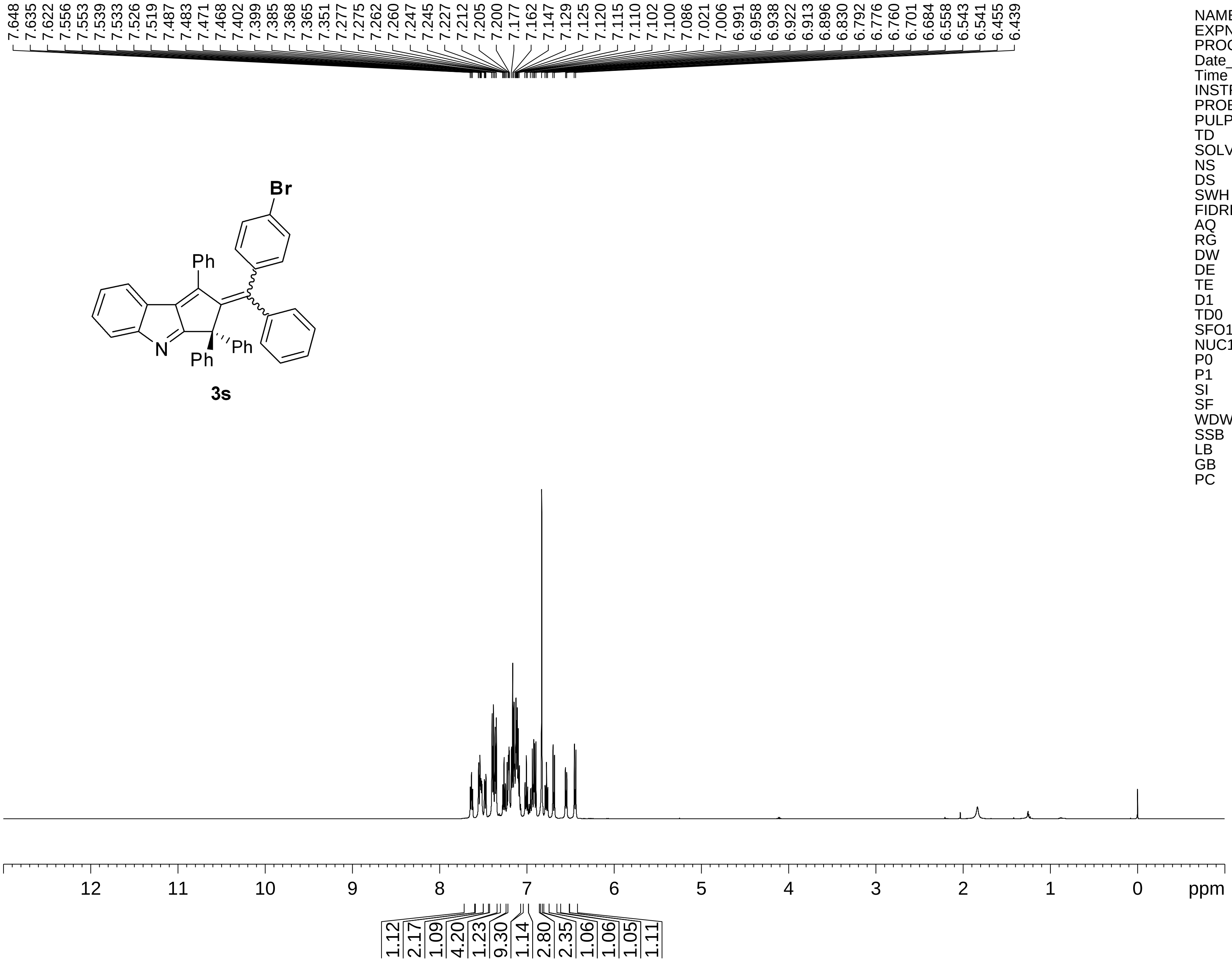


| | |
|---|---|
| NAME | qzw_688_3 |
| EXPNO | 10 |
| PROCNO | 1 |
| Date_ | 20210627 |
| Time | 2.01 h |
| INSTRUM | Avance NEO 500 |
| PROBHD | Z119470_0332 ( |
| PULPROG | zg30 |
| TD | 65536 |
| SOLVENT | CDCl3 |
| NS | 4 |
| DS | 2 |
| SWH | 10000.000 Hz |
| FIDRES | 0.305176 Hz |
| AQ | 3.2768500 sec |
| RG | 77.037 |
| DW | 50.000 usec |
| DE | 10.84 usec |
| TE | 296.1 K |
| D1 | 1.00000000 sec |
| TD0 | 1 |
| SFO1 | 500.1530884 MHz |
| NUC1 | 1H |
| P0 | 3.24 usec |
| P1 | 9.72 usec |
| SI | 65536 |
| SF | 500.1500285 MHz |
| WDW | EM |
| SSB | 0 |
| LB | 0.30 Hz |
| GB | 0 |
| PC | 1.00 |

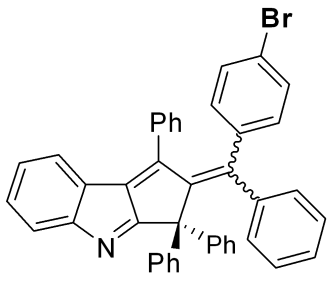

3s

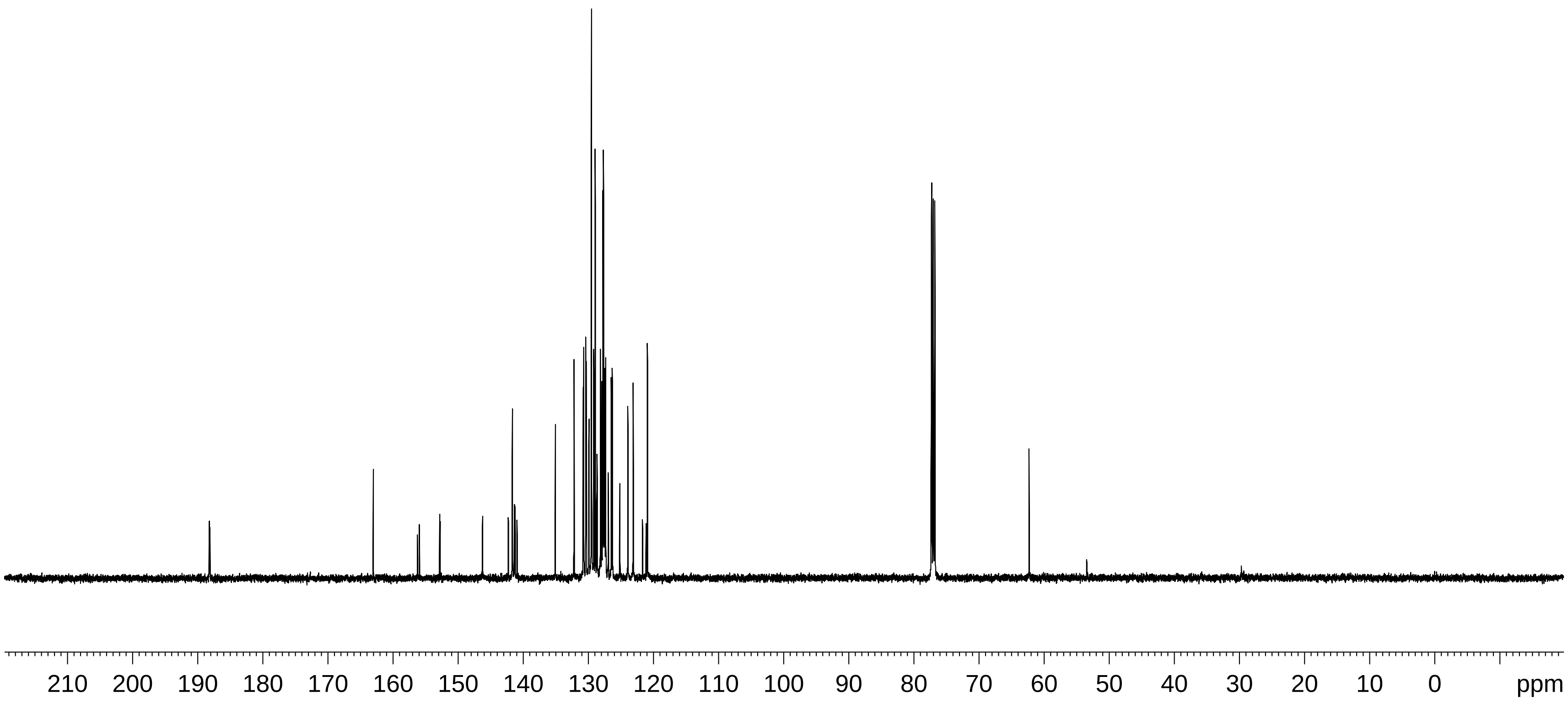


| | |
|---|---|
| NAME | qzw_688_3 |
| EXPNO | 30 |
| PROCNO | 1 |
| Date_ | 20210704 |
| Time | 6.34 h |
| INSTRUM | Avance NEO 500 |
| PROBHD | Z119470_0332 ( |
| PULPROG | zgpg30 |
| TD | 65536 |
| SOLVENT | CDCl3 |
| NS | 880 |
| DS | 4 |
| SWH | 30120.482 Hz |
| FIDRES | 0.919204 Hz |
| AQ | 1.0879476 sec |
| RG | 101 |
| DW | 16.600 usec |
| DE | 6.50 usec |
| TE | 296.1 K |
| D1 | 2.00000000 sec |
| D11 | 0.03000000 sec |
| TD0 | 1 |
| SFO1 | 125.7753938 MHz |
| NUC1 | 13C |
| P0 | 3.33 usec |
| P1 | 10.00 usec |
| SI | 32768 |
| SF | 125.7628279 MHz |
| WDW | EM |
| SSB | 0 |
| LB | 1.00 Hz |
| GB | 0 |
| PC | 1.40 |

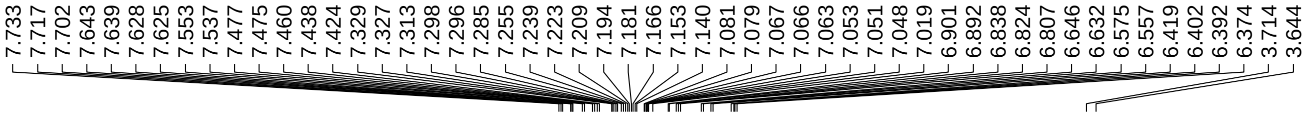


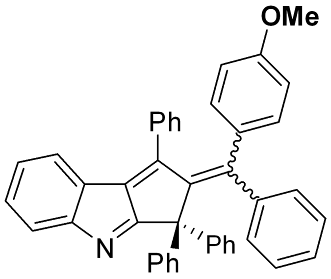


**3t**

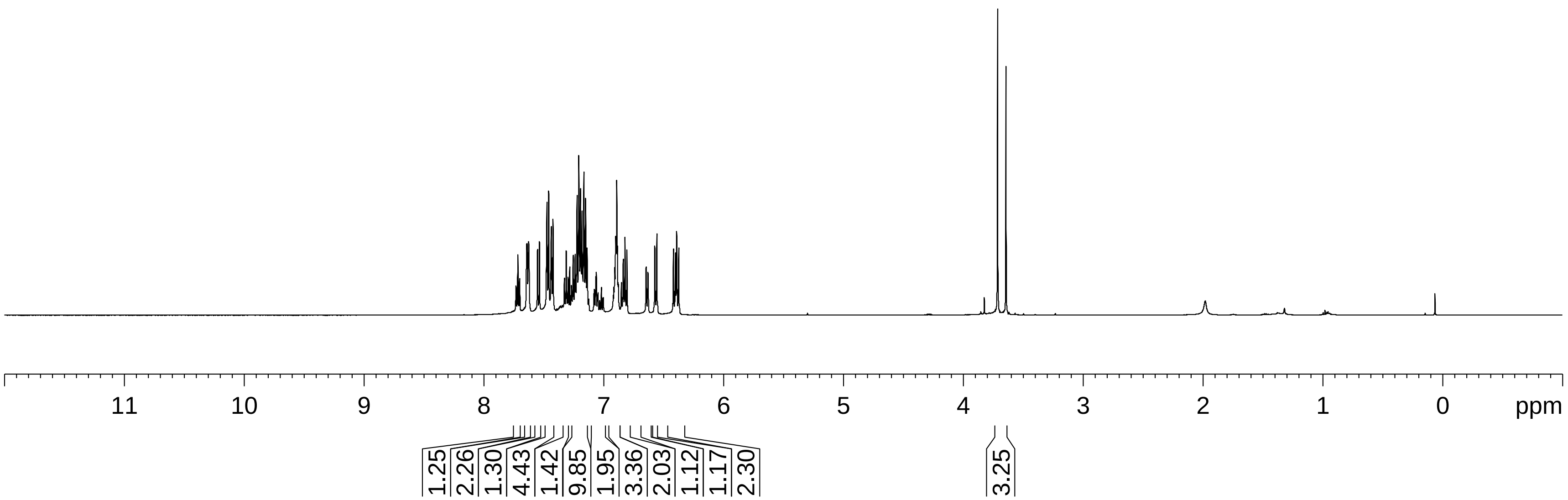


NAME qzw_688_1
EXPNO 20
PROCNO 1
Date_ 20210627
Time 12.35 h
INSTRUM Avance NEO 500
PROBHD Z119470_0332 (
PULPROG zg30
TD 65536
SOLVENT CDCl3
NS 2
DS 2
SWH 10000.000 Hz
FIDRES 0.305176 Hz
AQ 3.2768500 sec
RG 59.4286
DW 50.000 usec
DE 10.84 usec
TE 296.2 K
D1 1.00000000 sec
TD0 1
SFO1 500.1530884 MHz
NUC1 1H
P0 3.24 usec
P1 9.72 usec
SI 65536
SF 500.1500000 MHz
WDW EM
SSB 0
LB 0.30 Hz
GB 0
PC 1.00

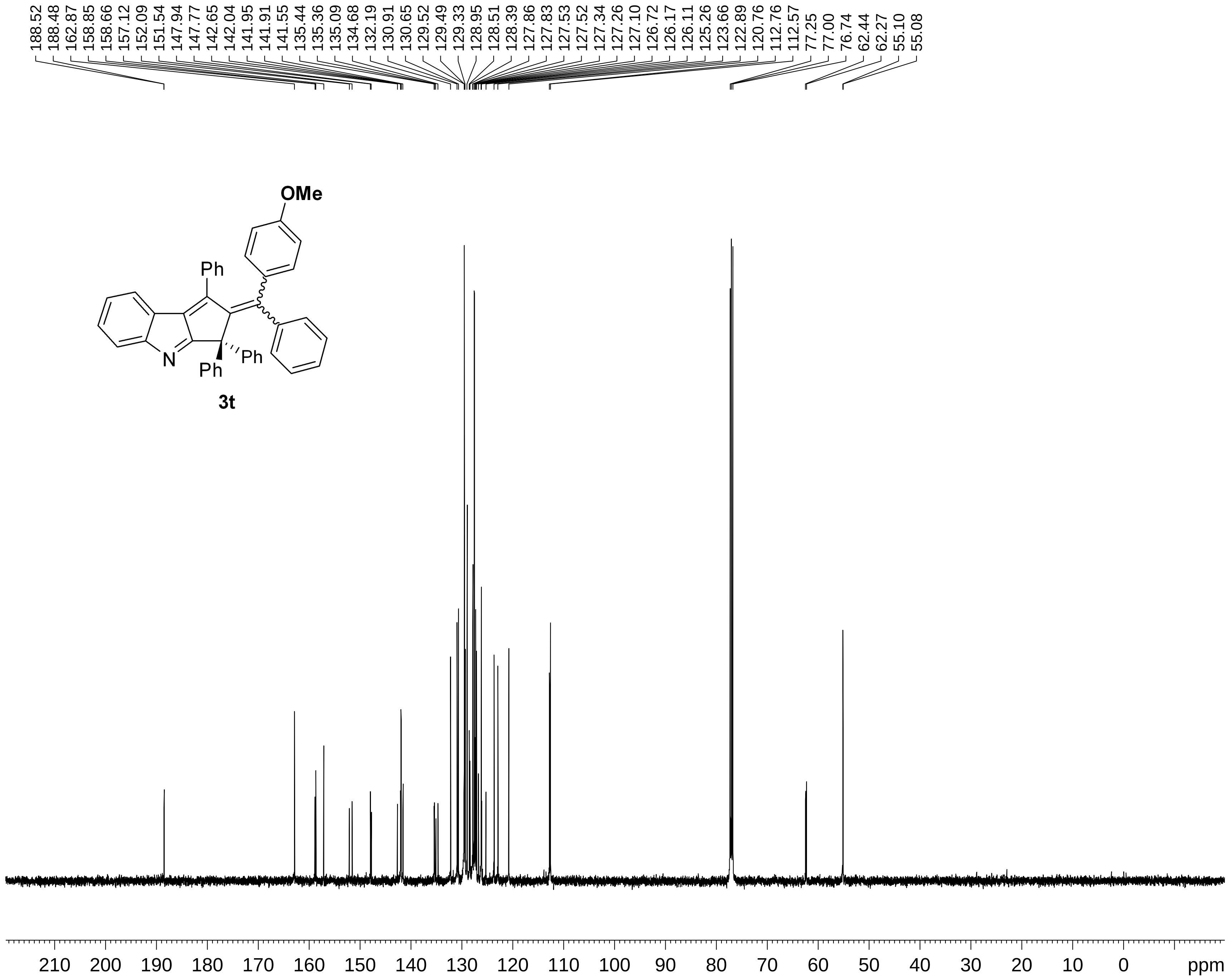


| | |
|---|---|
| NAME | qzw_688_1 |
| EXPNO | 21 |
| PROCNO | 1 |
| Date_ | 20210627 |
| Time | 12.47 h |
| INSTRUM | Avance NEO 500 |
| PROBHD | Z119470_0332 ( |
| PULPROG | zgpg30 |
| TD | 65536 |
| SOLVENT | CDCl3 |
| NS | 200 |
| DS | 4 |
| SWH | 30120.482 Hz |
| FIDRES | 0.919204 Hz |
| AQ | 1.0879476 sec |
| RG | 101 |
| DW | 16.600 usec |
| DE | 6.50 usec |
| TE | 296.2 K |
| D1 | 2.00000000 sec |
| D11 | 0.03000000 sec |
| TD0 | 1 |
| SFO1 | 125.7753938 MHz |
| NUC1 | 13C |
| P0 | 3.33 usec |
| P1 | 10.00 usec |
| SI | 32768 |
| SF | 125.7628343 MHz |
| WDW | EM |
| SSB | 0 |
| LB | 1.00 Hz |
| GB | 0 |
| PC | 1.40 |

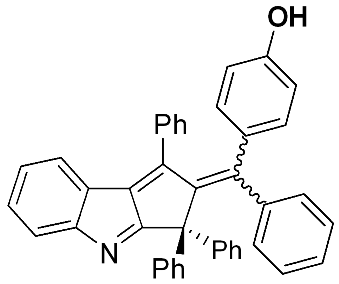


**3u**

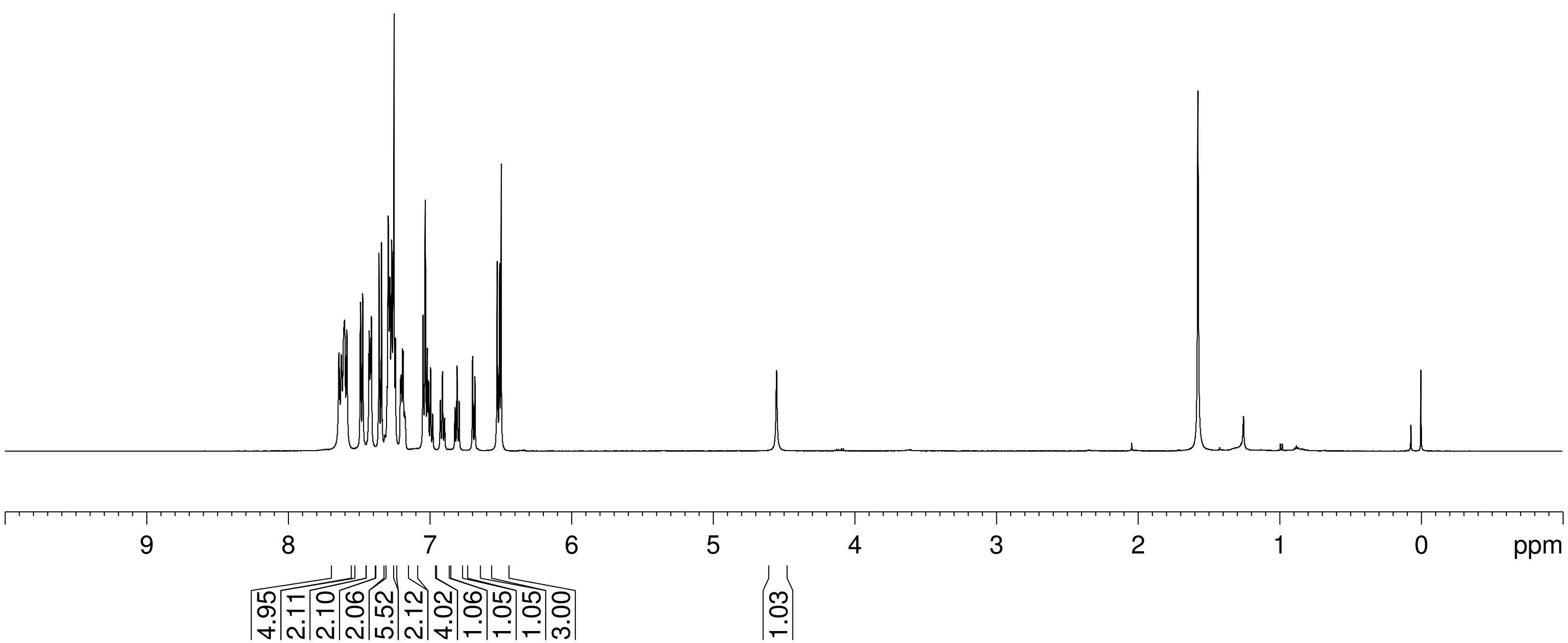


| | |
|---|---|
| NAME | m_wtf_894_2 |
| EXPNO | 10 |
| PROCNO | 1 |
| Date_ | 20240219 |
| Time | 13.01 h |
| INSTRUM | Avance NEO 500 |
| PROBHD | Z119470_0332 ( |
| PULPROG | zg30 |
| TD | 65536 |
| SOLVENT | CDCl3 |
| NS | 16 |
| DS | 2 |
| SWH | 10000.000 Hz |
| FIDRES | 0.305176 Hz |
| AQ | 3.2768500 sec |
| RG | 101 |
| DW | 50.000 usec |
| DE | 10.84 usec |
| TE | 295.0 K |
| D1 | 1.00000000 sec |
| TD0 | 1 |
| SFO1 | 500.1530884 MHz |
| NUC1 | 1H |
| P0 | 3.24 usec |
| P1 | 9.72 usec |
| SI | 65536 |
| SF | 500.1500159 MHz |
| WDW | EM |
| SSB | 0 |
| LB | 0.30 Hz |
| GB | 0 |
| PC | 1.00 |

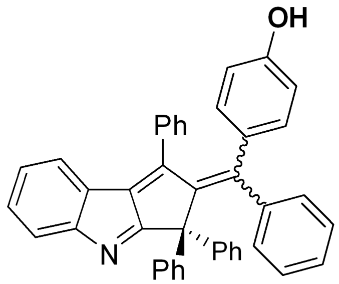


**3u**

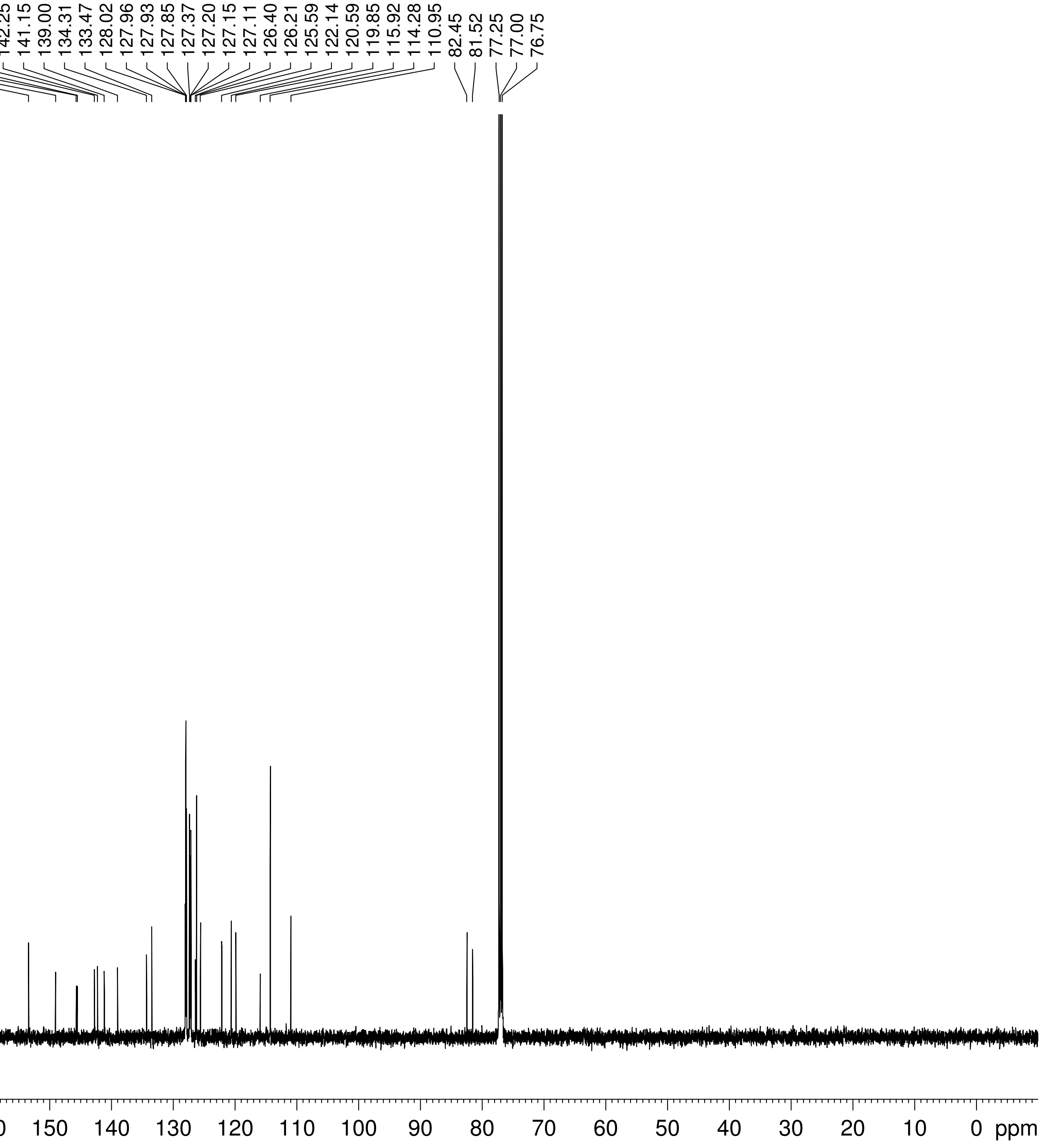


| | |
|---|---|
| NAME | m_wtf_894_2 |
| EXPNO | 11 |
| PROCNO | 1 |
| Date_ | 20240219 |
| Time | 13.32 h |
| INSTRUM | Avance NEO 500 |
| PROBHD | Z119470_0332 ( |
| PULPROG | zgpg30 |
| TD | 65536 |
| SOLVENT | CDCl3 |
| NS | 560 |
| DS | 4 |
| SWH | 30120.482 Hz |
| FIDRES | 0.919204 Hz |
| AQ | 1.0879476 sec |
| RG | 101 |
| DW | 16.600 usec |
| DE | 6.50 usec |
| TE | 296.0 K |
| D1 | 2.00000000 sec |
| D11 | 0.03000000 sec |
| TD0 | 1 |
| SFO1 | 125.7753938 MHz |
| NUC1 | 13C |
| P0 | 3.33 usec |
| P1 | 10.00 usec |
| SI | 32768 |
| SF | 125.7628227 MHz |
| WDW | EM |
| SSB | 0 |
| LB | 1.00 Hz |
| GB | 0 |
| PC | 1.40 |

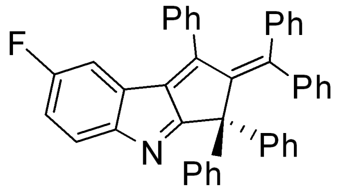


**4a**

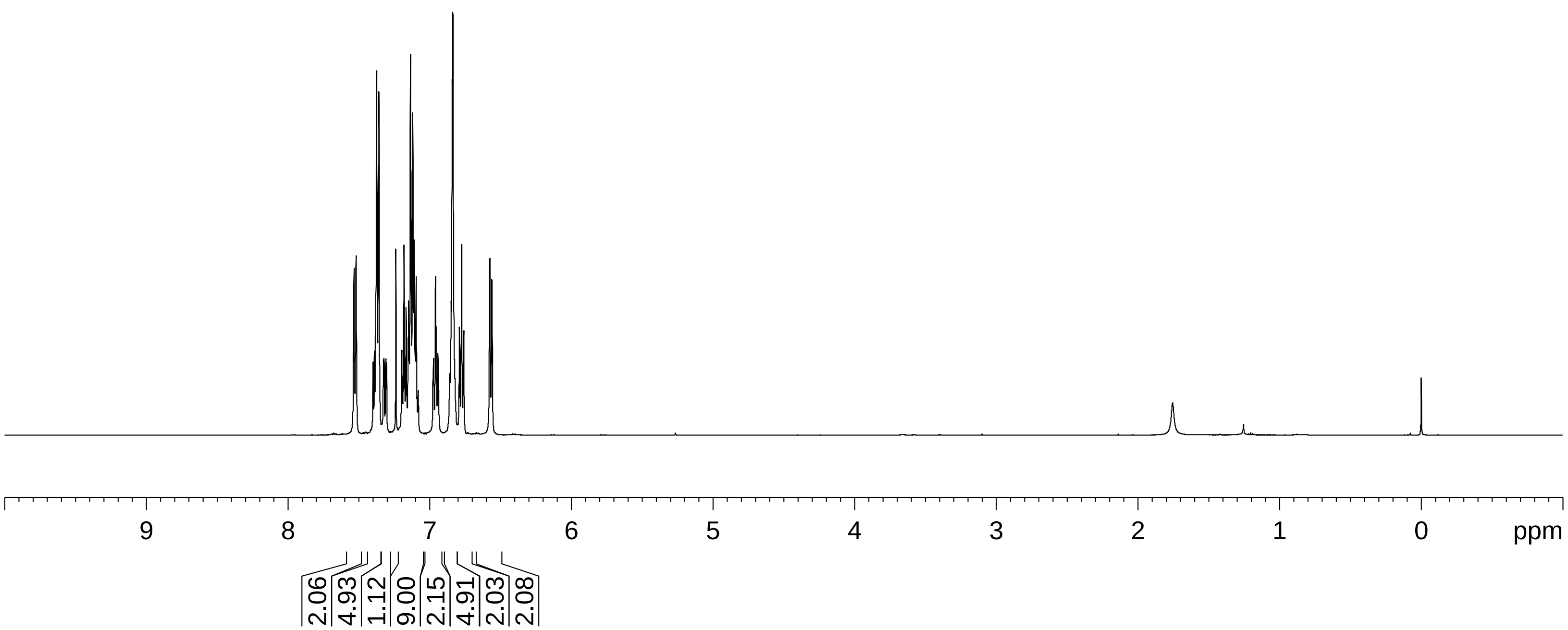


| | |
|---|---|
| NAME | qzw_686_1 |
| EXPNO | 10 |
| PROCNO | 1 |
| Date_ | 20210622 |
| Time | 23.06 h |
| INSTRUM | Avance NEO 500 |
| PROBHD | Z119470_0332 ( |
| PULPROG | zg30 |
| TD | 65536 |
| SOLVENT | CDCl3 |
| NS | 4 |
| DS | 2 |
| SWH | 10000.000 Hz |
| FIDRES | 0.305176 Hz |
| AQ | 3.2768500 sec |
| RG | 99.0476 |
| DW | 50.000 usec |
| DE | 10.84 usec |
| TE | 296.2 K |
| D1 | 1.00000000 sec |
| TD0 | 1 |
| SFO1 | 500.1530884 MHz |
| NUC1 | 1H |
| P0 | 3.24 usec |
| P1 | 9.72 usec |
| SI | 65536 |
| SF | 500.1500225 MHz |
| WDW | EM |
| SSB | 0 |
| LB | 0.30 Hz |
| GB | 0 |
| PC | 1.00 |

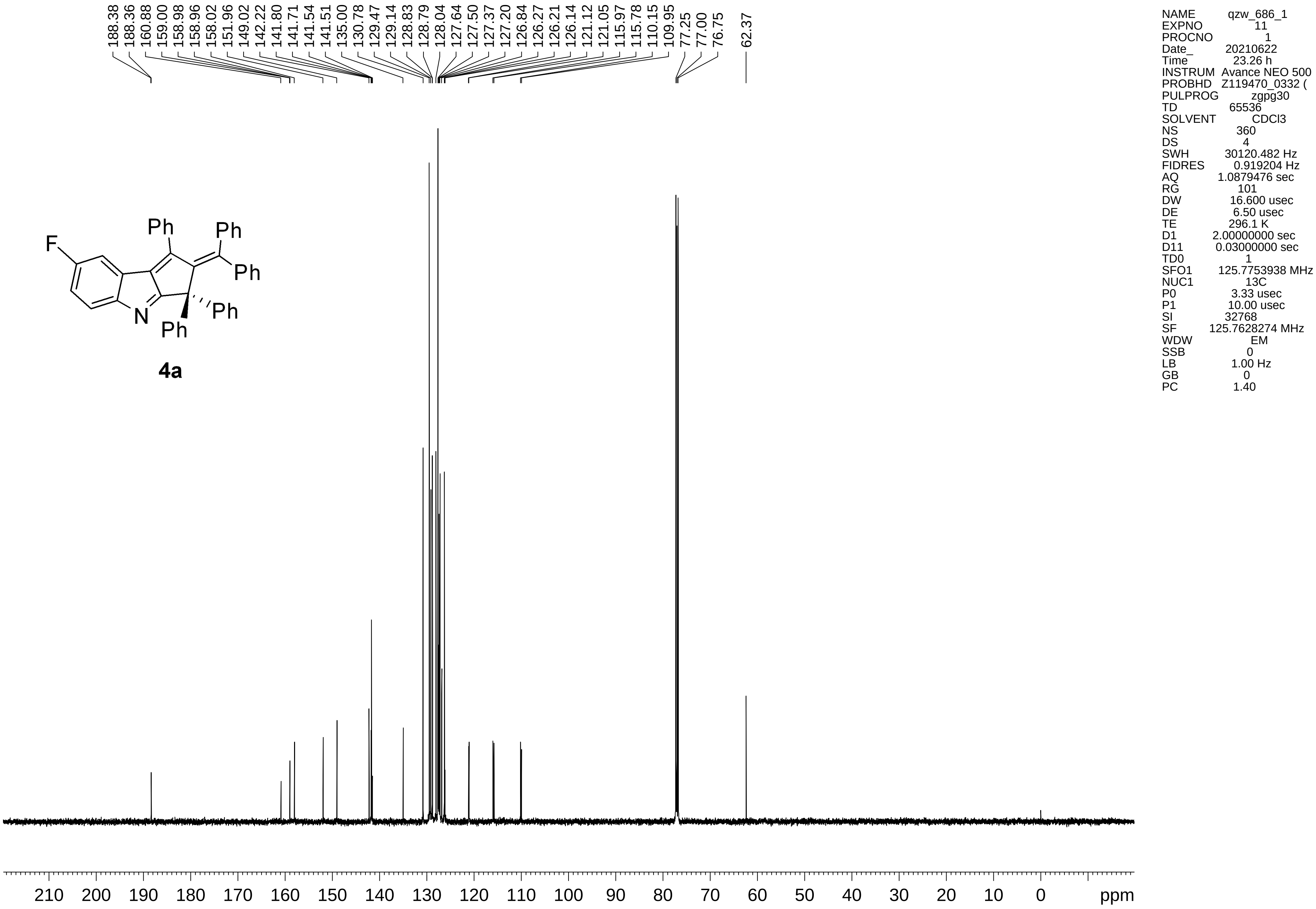
F
Ph
Ph
Ph
N
Ph
Ph
4a
188.38
188.36
160.88
159.00
158.98
158.96
158.02
151.96
149.02
142.22
141.80
141.71
141.54
141.51
135.00
130.78
129.47
129.14
128.83
128.79
128.04
127.64
127.50
127.37
127.20
126.84
126.27
126.21
126.14
121.12
121.05
115.97
115.78
110.15
109.95
77.25
77.00
76.75
62.37
210 200 190 180 170 160 150 140 130 120 110 100 90 80 70 60 50 40 30 20 10 0 ppm
NAME qzw_686_1
EXPNO 11
PROCNO 1
Date_ 20210622
Time 23.26 h
INSTRUM Avance NEO 500
PROBHD Z119470_0332 (
PULPROG zgpg30
TD 65536
SOLVENT CDCl3
NS 360
DS 4
SWH 30120.482 Hz
FIDRES 0.919204 Hz
AQ 1.0879476 sec
RG 101
DW 16.600 usec
DE 6.50 usec
TE 296.1 K
D1 2.00000000 sec
D11 0.03000000 sec
TD0 1
SFO1 125.7753938 MHz
NUC1 13C
P0 3.33 usec
P1 10.00 usec
SI 32768
SF 125.7628274 MHz
WDW EM
SSB 0
LB 1.00 Hz
GB 0
PC 1.40

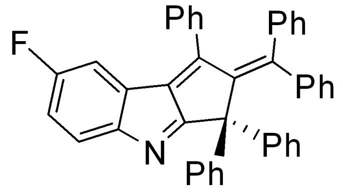


**4a**

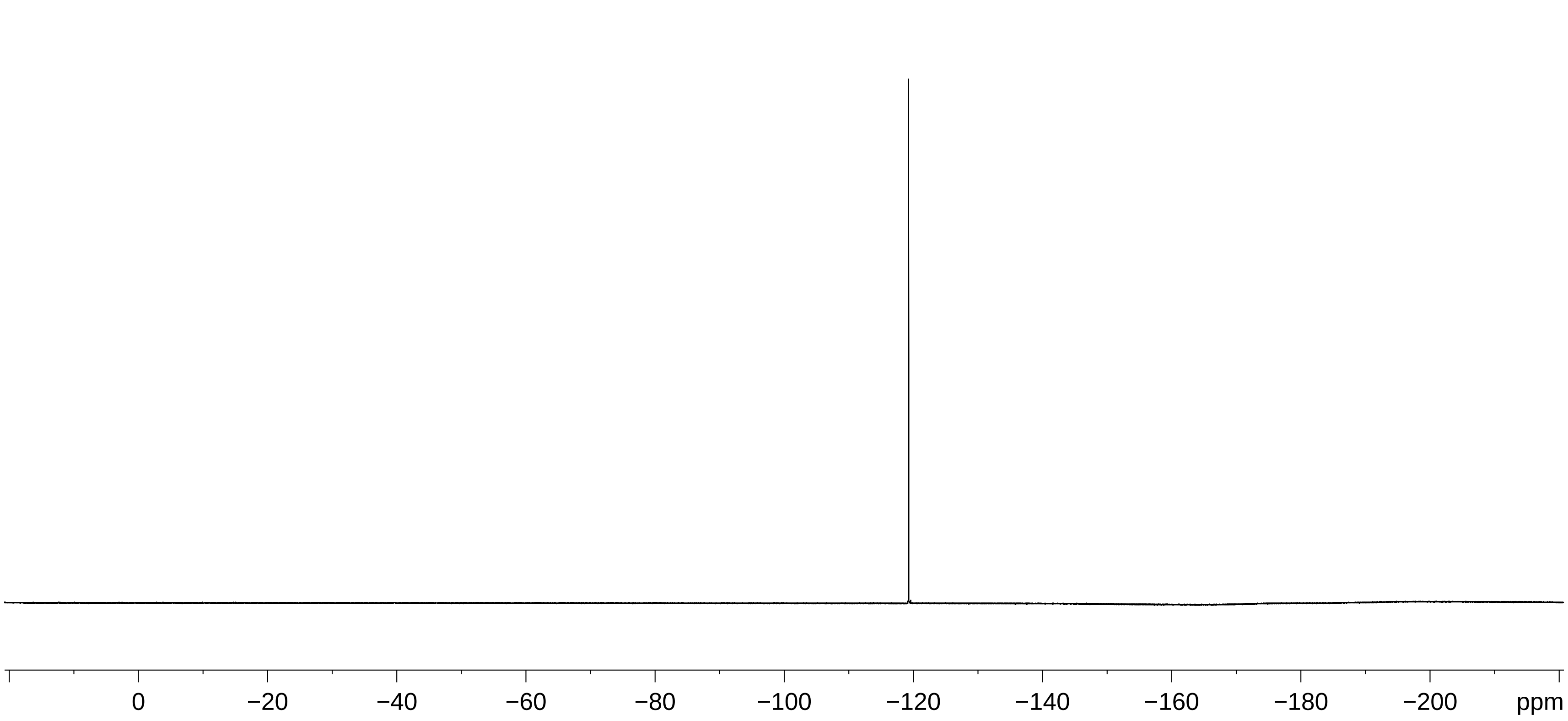


| | |
|---|---|
| NAME | qzw_686_1 |
| EXPNO | 12 |
| PROCNO | 1 |
| Date_ | 20210622 |
| Time | 23.27 h |
| INSTRUM | Avance NEO 500 |
| PROBHD | Z119470_0332 ( |
| PULPROG | zg |
| TD | 131072 |
| SOLVENT | CDCl3 |
| NS | 8 |
| DS | 4 |
| SWH | 113636.367 Hz |
| FIDRES | 1.733953 Hz |
| AQ | 0.5767668 sec |
| RG | 101 |
| DW | 4.400 usec |
| DE | 6.50 usec |
| TE | 296.1 K |
| D1 | 1.00000000 sec |
| TD0 | 1 |
| SFO1 | 470.5641349 MHz |
| NUC1 | 19F |
| P1 | 15.00 usec |
| SI | 65536 |
| SF | 470.6111960 MHz |
| WDW | EM |
| SSB | 0 |
| LB | 0.30 Hz |
| GB | 0 |
| PC | 1.00 |

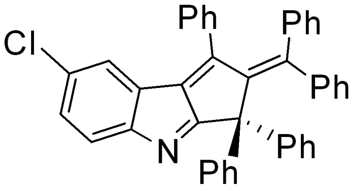


**4b**

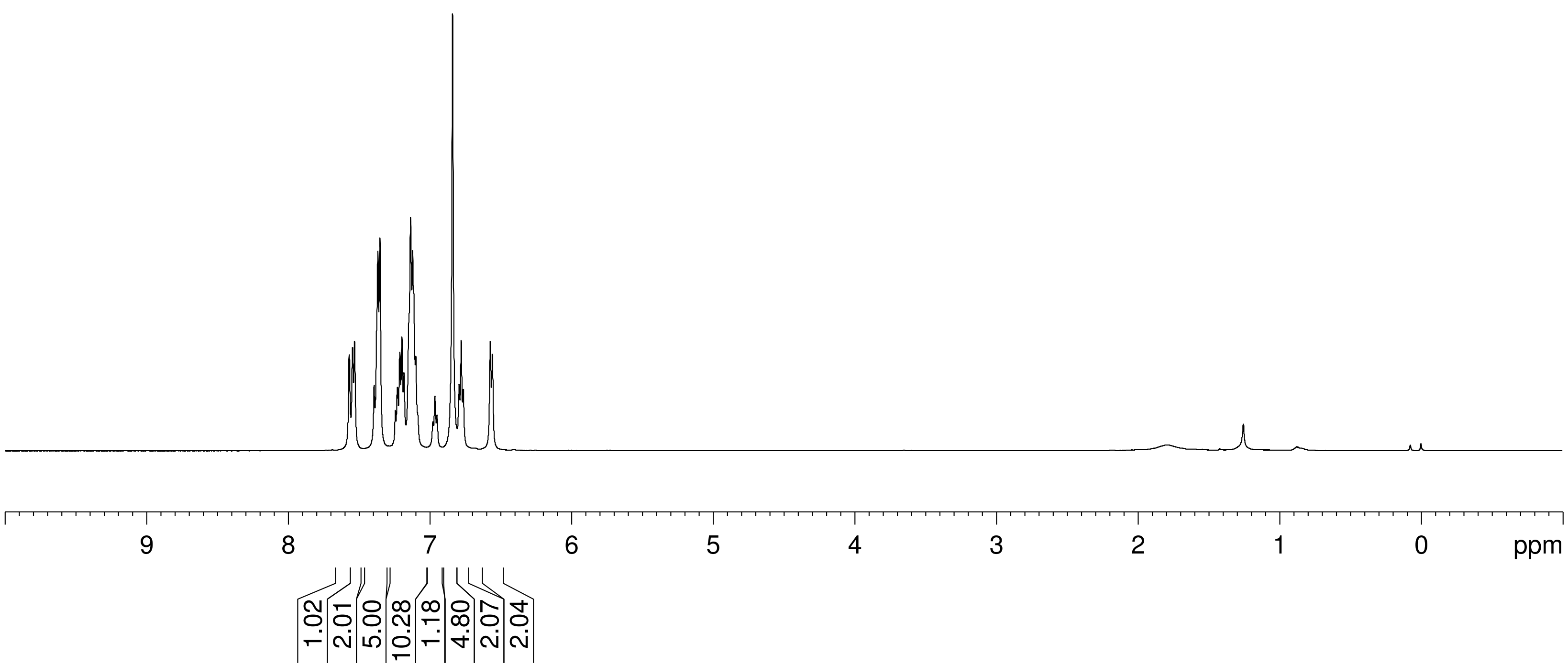


NAME m_qzw_686_2
EXPNO 10
PROCNO 1
Date_ 20240214
Time 11.55 h
INSTRUM Avance NEO 500
PROBHD Z119470_0332 (
PULPROG zg30
TD 65536
SOLVENT CDCl3
NS 8
DS 2
SWH 10000.000 Hz
FIDRES 0.305176 Hz
AQ 3.2768500 sec
RG 101
DW 50.000 usec
DE 10.84 usec
TE 294.7 K
D1 1.00000000 sec
TD0 1
SFO1 500.1530884 MHz
NUC1 1H
P0 3.24 usec
P1 9.72 usec
SI 65536
SF 500.1500203 MHz
WDW EM
SSB 0
LB 0.30 Hz
GB 0
PC 1.00

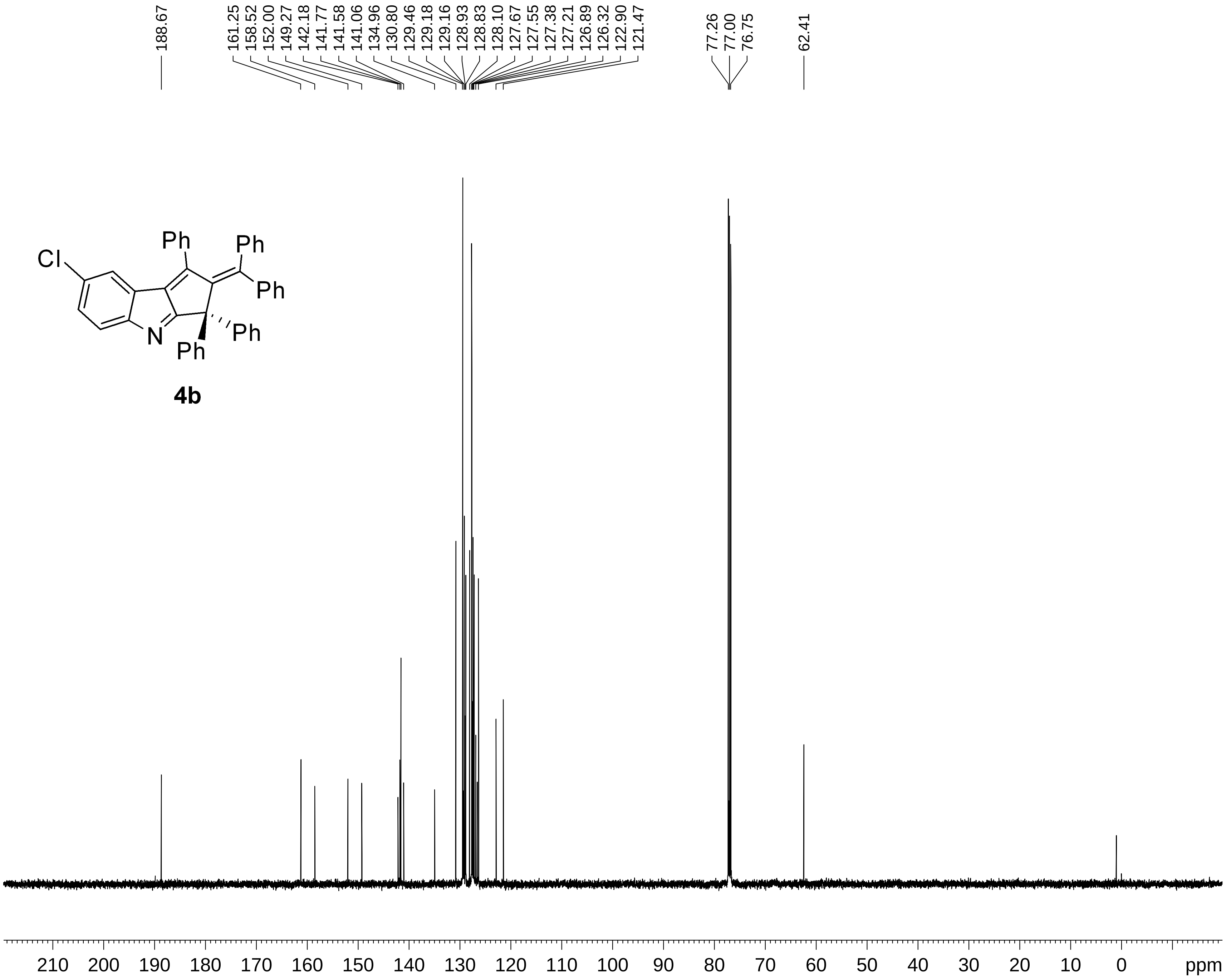


**4b**

| | |
|---|---|
| NAME | qzw_686_2 |
| EXPNO | 11 |
| PROCNO | 1 |
| Date_ | 20210623 |
| Time | 7.19 h |
| INSTRUM | Avance NEO 500 |
| PROBHD | Z119470_0332 ( |
| PULPROG | zgpg30 |
| TD | 65536 |
| SOLVENT | CDCl3 |
| NS | 200 |
| DS | 4 |
| SWH | 30120.482 Hz |
| FIDRES | 0.919204 Hz |
| AQ | 1.0879476 sec |
| RG | 101 |
| DW | 16.600 usec |
| DE | 6.50 usec |
| TE | 296.2 K |
| D1 | 2.00000000 sec |
| D11 | 0.03000000 sec |
| TD0 | 1 |
| SFO1 | 125.7753938 MHz |
| NUC1 | 13C |
| P0 | 3.33 usec |
| P1 | 10.00 usec |
| SI | 32768 |
| SF | 125.7628270 MHz |
| WDW | EM |
| SSB | 0 |
| LB | 1.00 Hz |
| GB | 0 |
| PC | 1.40 |

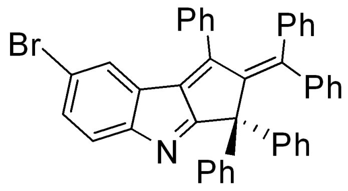


**4c**

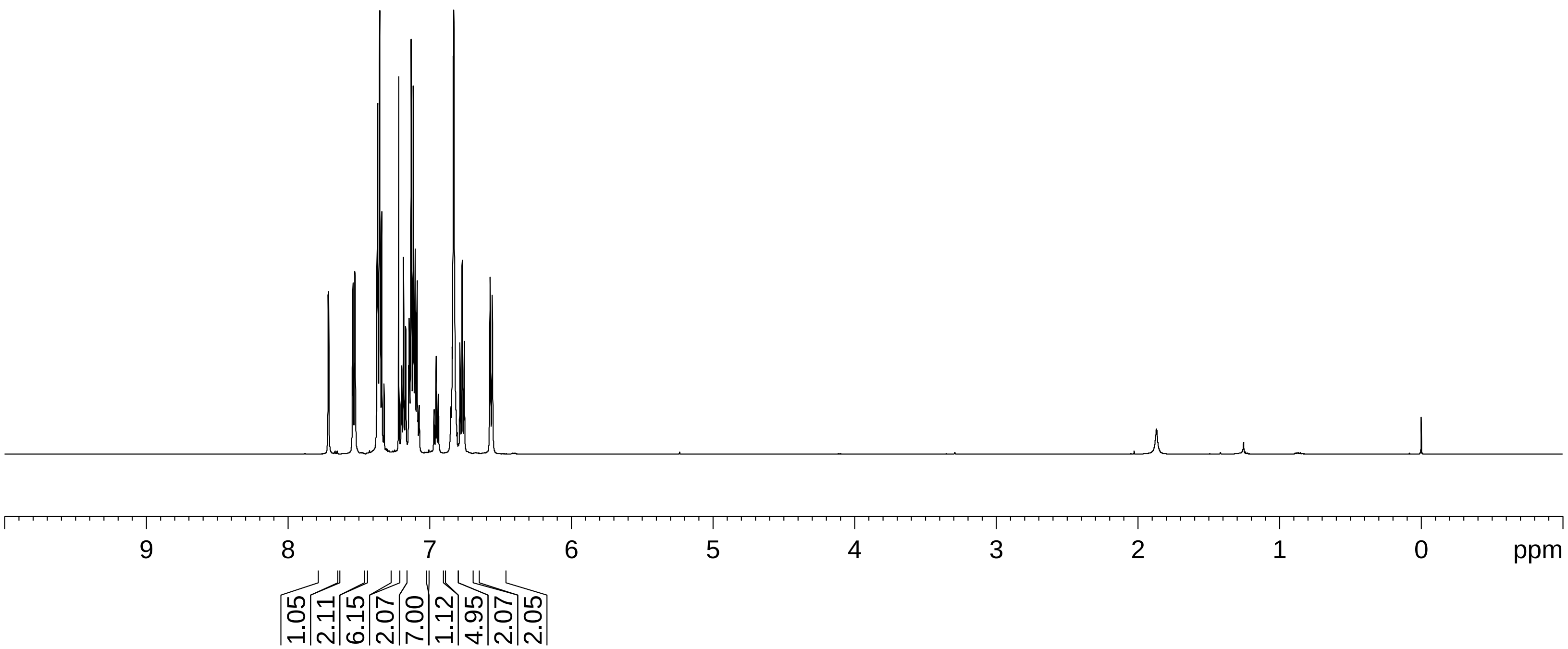


| | |
|---|---|
| NAME | qzw_686_3 |
| EXPNO | 10 |
| PROCNO | 1 |
| Date_ | 20210623 |
| Time | 7.22 h |
| INSTRUM | Avance NEO 500 |
| PROBHD | Z119470_0332 ( |
| PULPROG | zg30 |
| TD | 65536 |
| SOLVENT | CDCl3 |
| NS | 4 |
| DS | 2 |
| SWH | 10000.000 Hz |
| FIDRES | 0.305176 Hz |
| AQ | 3.2768500 sec |
| RG | 61.1765 |
| DW | 50.000 usec |
| DE | 10.84 usec |
| TE | 296.1 K |
| D1 | 1.00000000 sec |
| TD0 | 1 |
| SFO1 | 500.1530884 MHz |
| NUC1 | 1H |
| P0 | 3.24 usec |
| P1 | 9.72 usec |
| SI | 65536 |
| SF | 500.1500326 MHz |
| WDW | EM |
| SSB | 0 |
| LB | 0.30 Hz |
| GB | 0 |
| PC | 1.00 |

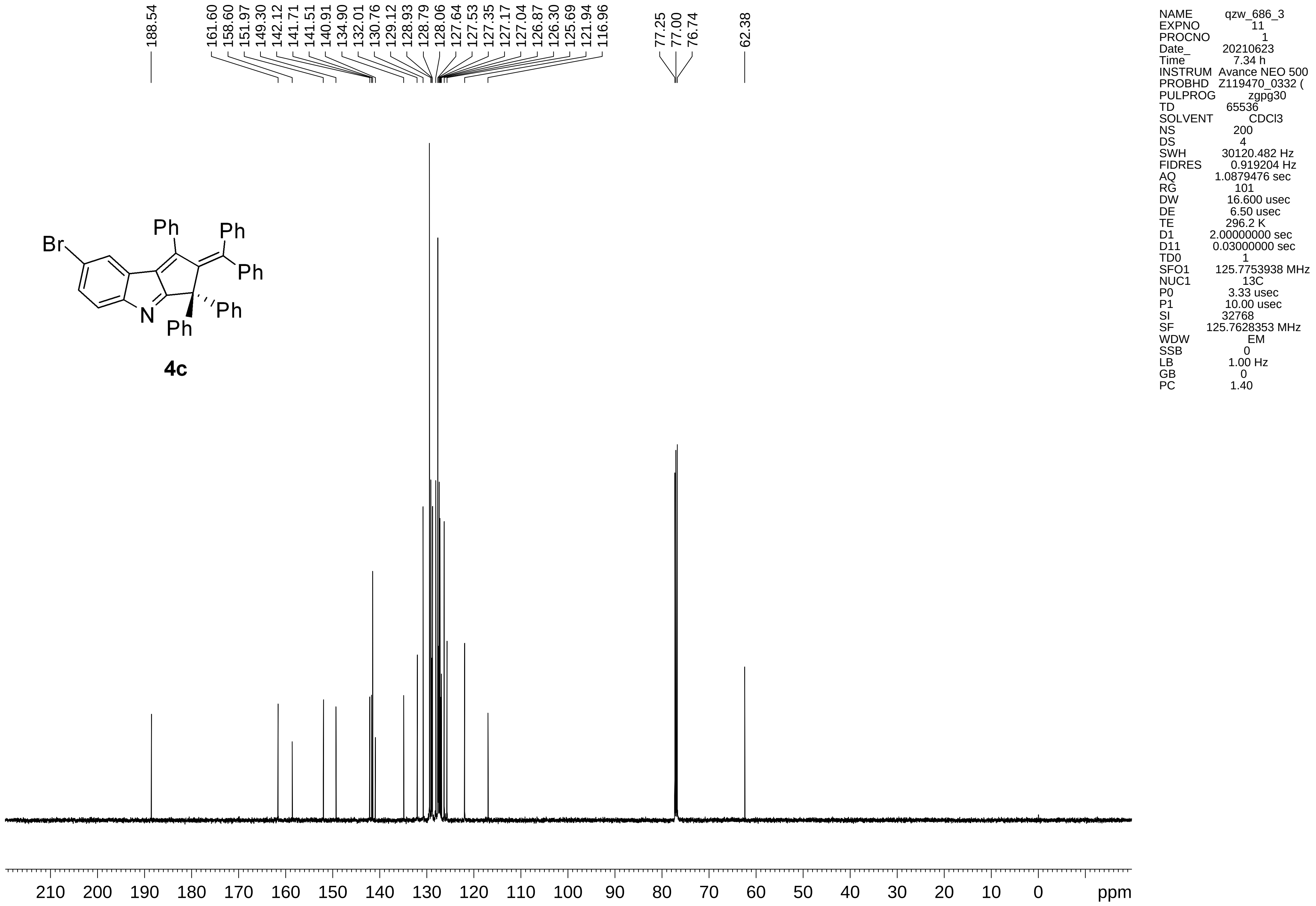
188.54
161.60
158.60
151.97
149.30
142.12
141.71
141.51
140.91
134.90
132.01
130.76
129.12
128.93
128.79
128.06
127.64
127.53
127.35
127.17
127.04
126.87
126.30
125.69
121.94
116.96
77.25
77.00
76.74
62.38
Br
Ph
Ph
Ph
N
Ph
Ph
4c
NAME qzw_686_3
EXPNO 11
PROCNO 1
Date_ 20210623
Time 7.34 h
INSTRUM Avance NEO 500
PROBHD Z119470_0332 (
PULPROG zgpg30
TD 65536
SOLVENT CDCl3
NS 200
DS 4
SWH 30120.482 Hz
FIDRES 0.919204 Hz
AQ 1.0879476 sec
RG 101
DW 16.600 usec
DE 6.50 usec
TE 296.2 K
D1 2.00000000 sec
D11 0.03000000 sec
TD0 1
SFO1 125.7753938 MHz
NUC1 13C
P0 3.33 usec
P1 10.00 usec
SI 32768
SF 125.7628353 MHz
WDW EM
SSB 0
LB 1.00 Hz
GB 0
PC 1.40
210 200 190 180 170 160 150 140 130 120 110 100 90 80 70 60 50 40 30 20 10 0 ppm

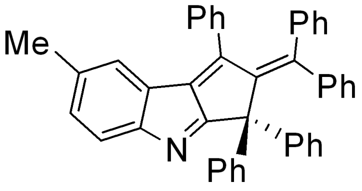


**4d**

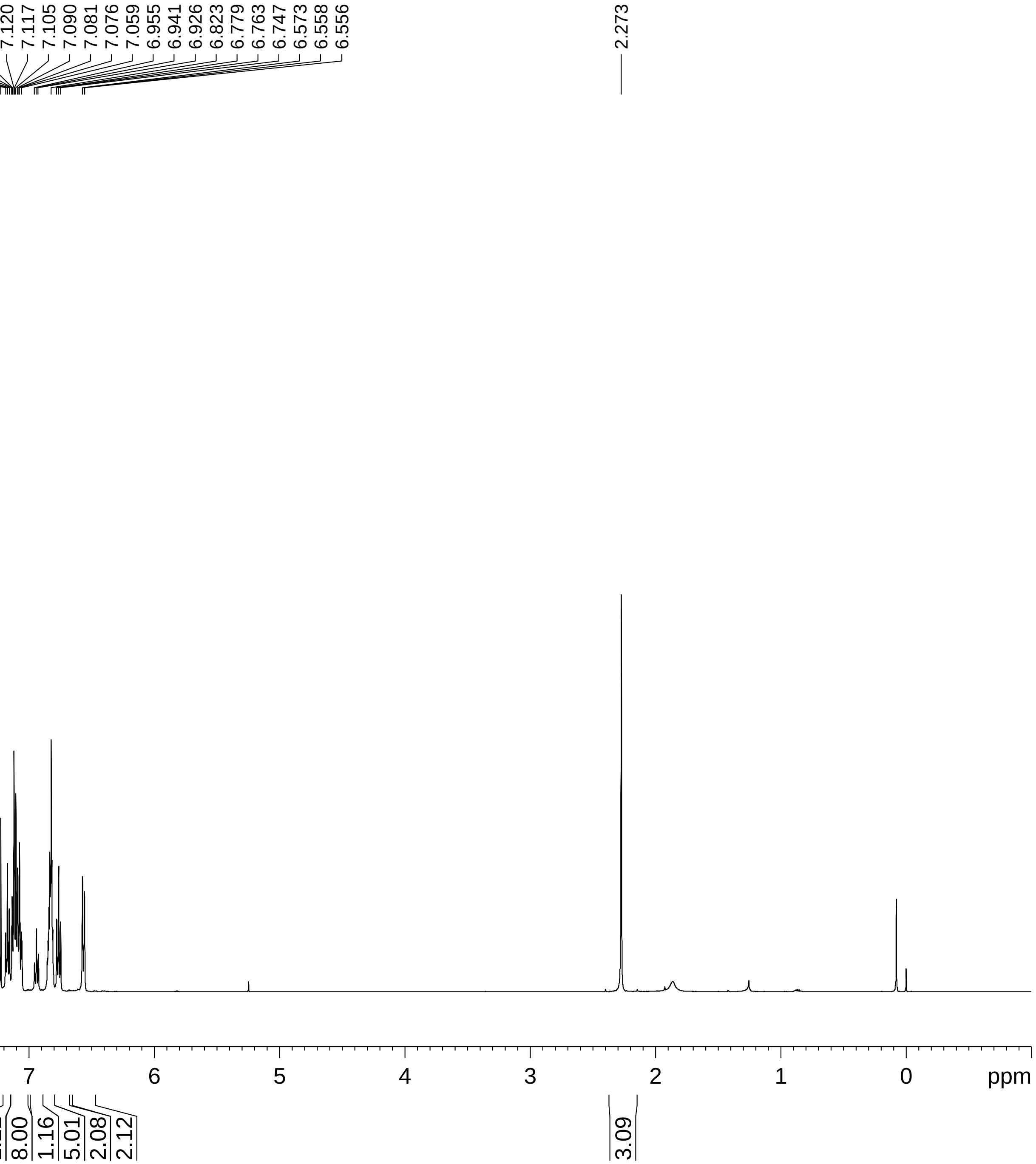


| | |
|---|---|
| NAME | qzw_686_4 |
| EXPNO | 10 |
| PROCNO | 1 |
| Date_ | 20210623 |
| Time | 18.14 h |
| INSTRUM | Avance NEO 500 |
| PROBHD | Z119470_0332 ( |
| PULPROG | zg30 |
| TD | 65536 |
| SOLVENT | CDCl3 |
| NS | 4 |
| DS | 2 |
| SWH | 10000.000 Hz |
| FIDRES | 0.305176 Hz |
| AQ | 3.2768500 sec |
| RG | 74.2857 |
| DW | 50.000 usec |
| DE | 10.84 usec |
| TE | 296.1 K |
| D1 | 1.00000000 sec |
| TD0 | 1 |
| SFO1 | 500.1530884 MHz |
| NUC1 | 1H |
| P0 | 3.24 usec |
| P1 | 9.72 usec |
| SI | 65536 |
| SF | 500.1500295 MHz |
| WDW | EM |
| SSB | 0 |
| LB | 0.30 Hz |
| GB | 0 |
| PC | 1.00 |

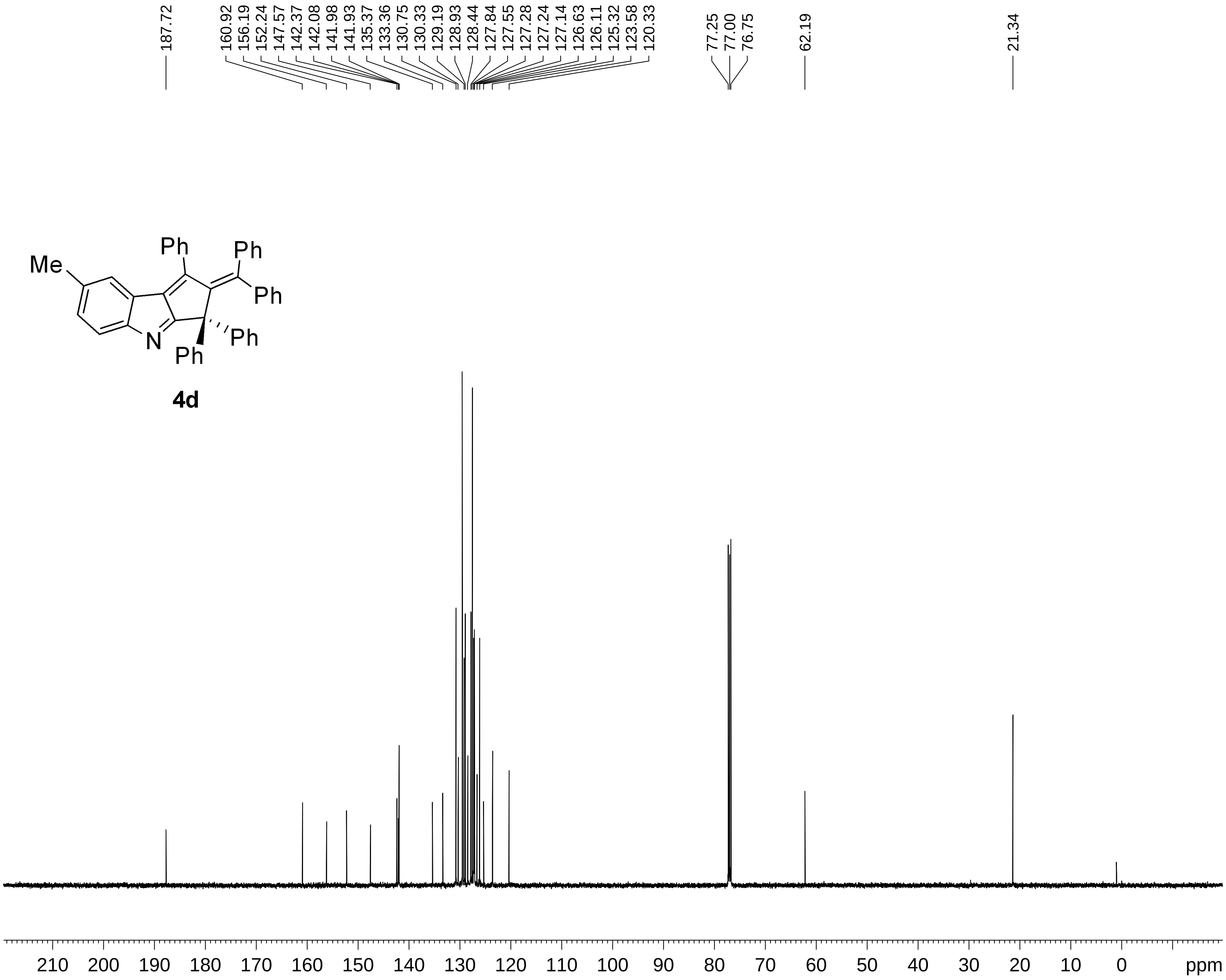


**4d**

| | |
|---|---|
| NAME | qzw_686_4 |
| EXPNO | 11 |
| PROCNO | 1 |
| Date_ | 20210623 |
| Time | 18.26 h |
| INSTRUM | Avance NEO 500 |
| PROBHD | Z119470_0332 ( |
| PULPROG | zgpg30 |
| TD | 65536 |
| SOLVENT | CDCl3 |
| NS | 200 |
| DS | 4 |
| SWH | 30120.482 Hz |
| FIDRES | 0.919204 Hz |
| AQ | 1.0879476 sec |
| RG | 101 |
| DW | 16.600 usec |
| DE | 6.50 usec |
| TE | 296.2 K |
| D1 | 2.00000000 sec |
| D11 | 0.03000000 sec |
| TD0 | 1 |
| SFO1 | 125.7753938 MHz |
| NUC1 | 13C |
| P0 | 3.33 usec |
| P1 | 10.00 usec |
| SI | 32768 |
| SF | 125.7628311 MHz |
| WDW | EM |
| SSB | 0 |
| LB | 1.00 Hz |
| GB | 0 |
| PC | 1.40 |

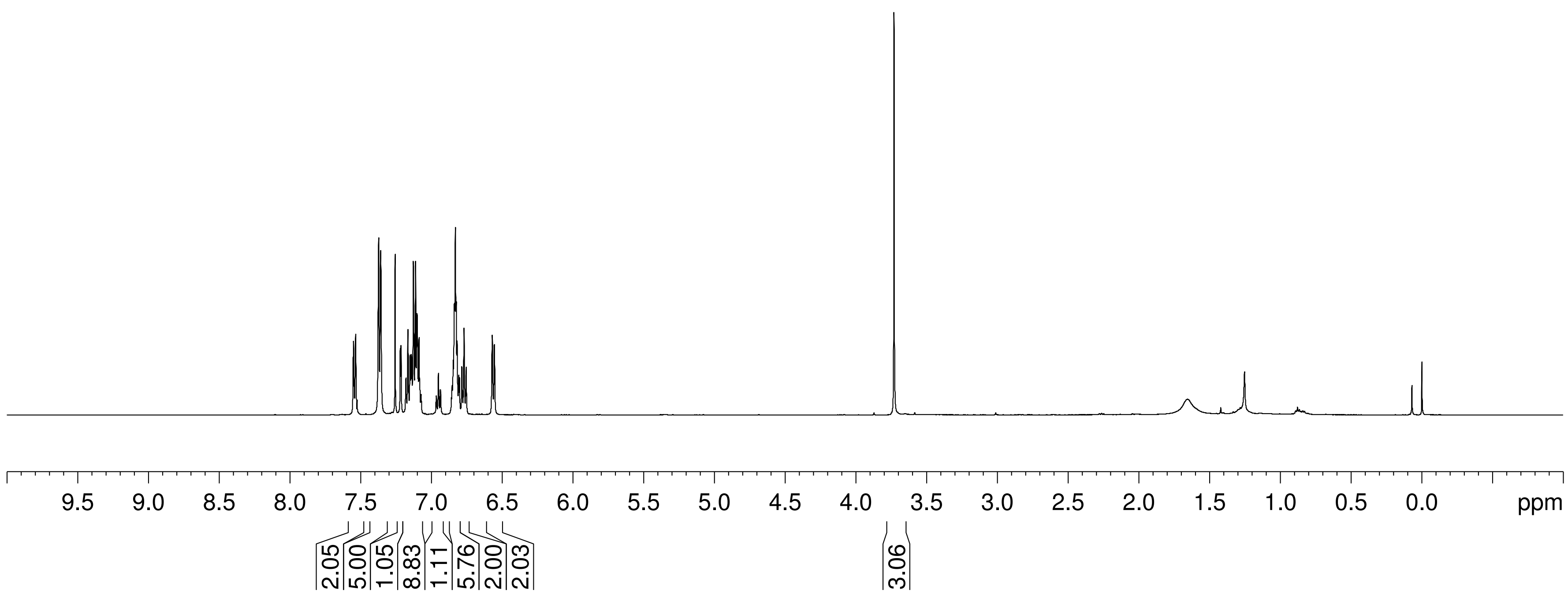


| | |
|---|---|
| NAME | m_qzw_686_5 |
| EXPNO | 10 |
| PROCNO | 1 |
| Date_ | 20240214 |
| Time | 12.17 h |
| INSTRUM | Avance NEO 500 |
| PROBHD | Z119470_0332 ( |
| PULPROG | zg30 |
| TD | 65536 |
| SOLVENT | CDCl3 |
| NS | 16 |
| DS | 2 |
| SWH | 10000.000 Hz |
| FIDRES | 0.305176 Hz |
| AQ | 3.2768500 sec |
| RG | 101 |
| DW | 50.000 usec |
| DE | 10.84 usec |
| TE | 294.6 K |
| D1 | 1.00000000 sec |
| TD0 | 1 |
| SFO1 | 500.1530884 MHz |
| NUC1 | 1H |
| P0 | 3.24 usec |
| P1 | 9.72 usec |
| SI | 65536 |
| SF | 500.1500136 MHz |
| WDW | EM |
| SSB | 0 |
| LB | 0.30 Hz |
| GB | 0 |
| PC | 1.00 |

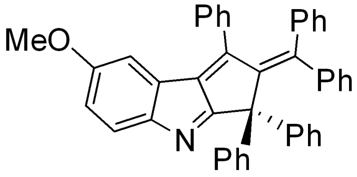


**4e**

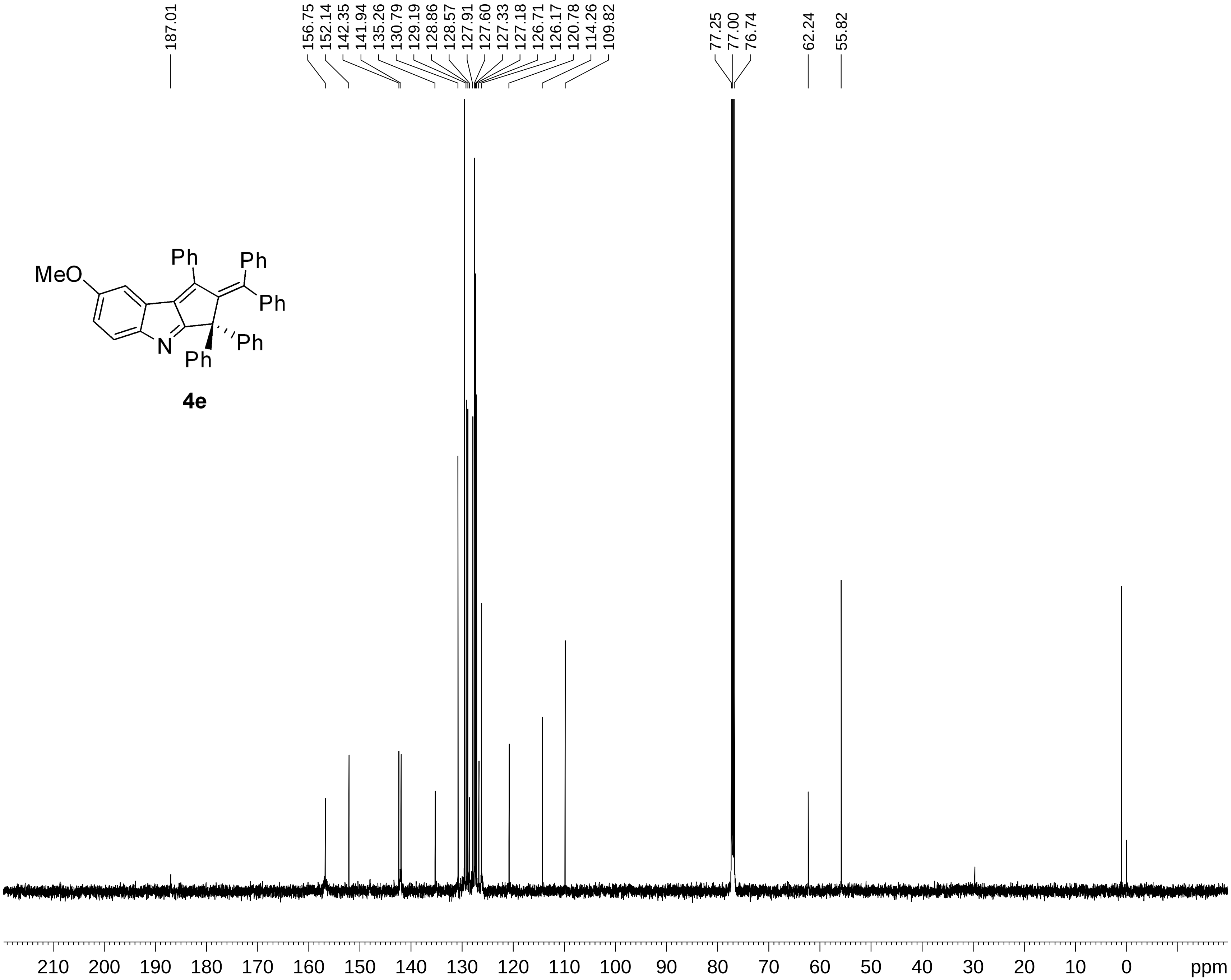


**4e**

| | |
|---|---|
| NAME | qzw_686_5 |
| EXPNO | 40 |
| PROCNO | 1 |
| Date_ | 20210711 |
| Time | 4.48 h |
| INSTRUM | Avance NEO 500 |
| PROBHD | Z119470_0332 ( |
| PULPROG | zgpg30 |
| TD | 65536 |
| SOLVENT | CDCl3 |
| NS | 1400 |
| DS | 4 |
| SWH | 30120.482 Hz |
| FIDRES | 0.919204 Hz |
| AQ | 1.0879476 sec |
| RG | 101 |
| DW | 16.600 usec |
| DE | 6.50 usec |
| TE | 296.2 K |
| D1 | 2.00000000 sec |
| D11 | 0.03000000 sec |
| TD0 | 1 |
| SFO1 | 125.7753938 MHz |
| NUC1 | 13C |
| P0 | 3.33 usec |
| P1 | 10.00 usec |
| SI | 32768 |
| SF | 125.7628233 MHz |
| WDW | EM |
| SSB | 0 |
| LB | 1.00 Hz |
| GB | 0 |
| PC | 1.40 |

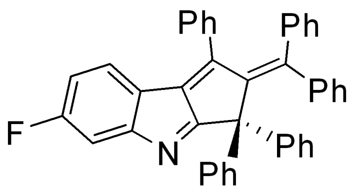


**4f**

NAME qzw_687_1_m
EXPNO 20
PROCNO 1
Date_ 20240218
Time 16.01 h
INSTRUM Avance NEO 500
PROBHD Z119470_0332 (
PULPROG zg30
TD 65536
SOLVENT CDCl3
NS 16
DS 2
SWH 10000.000 Hz
FIDRES 0.305176 Hz
AQ 3.2768500 sec
RG 101
DW 50.000 usec
DE 10.84 usec
TE 294.9 K
D1 1.00000000 sec
TD0 1
SFO1 500.1530884 MHz
NUC1 1H
P0 3.24 usec
P1 9.72 usec
SI 65536
SF 500.1500135 MHz
WDW EM
SSB 0
LB 0.30 Hz
GB 0
PC 1.00

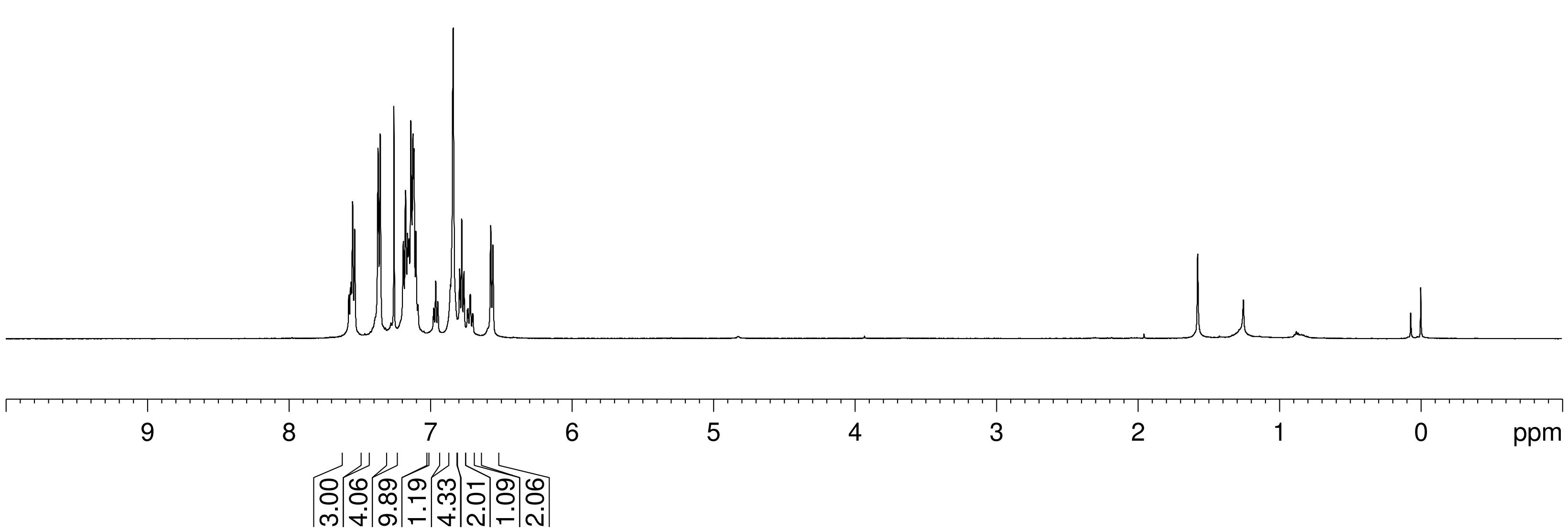

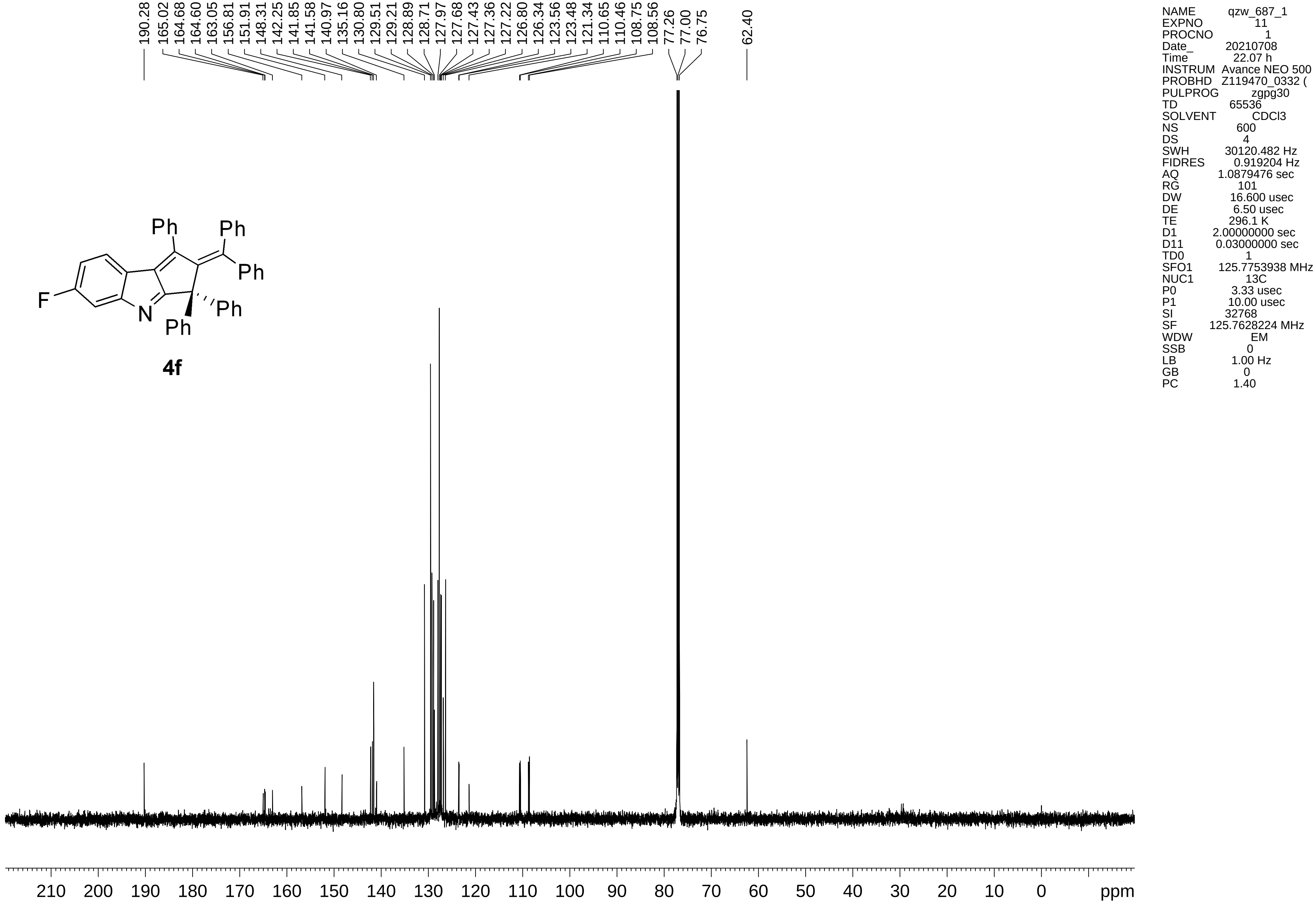
Ph
Ph
Ph
F
N
Ph
Ph
4f
190.28
165.02
164.68
164.60
163.05
156.81
151.91
148.31
142.25
141.85
141.58
140.97
135.16
130.80
129.51
129.21
128.89
128.71
127.97
127.68
127.43
127.36
127.22
126.80
126.34
123.56
123.48
121.34
110.65
110.46
108.75
108.56
77.26
77.00
76.75
62.40
210 200 190 180 170 160 150 140 130 120 110 100 90 80 70 60 50 40 30 20 10 0 ppm
NAME qzw_687_1
EXPNO 11
PROCNO 1
Date_ 20210708
Time 22.07 h
INSTRUM Avance NEO 500
PROBHD Z119470_0332 (
PULPROG zgpg30
TD 65536
SOLVENT CDCl3
NS 600
DS 4
SWH 30120.482 Hz
FIDRES 0.919204 Hz
AQ 1.0879476 sec
RG 101
DW 16.600 usec
DE 6.50 usec
TE 296.1 K
D1 2.00000000 sec
D11 0.03000000 sec
TD0 1
SFO1 125.7753938 MHz
NUC1 13C
P0 3.33 usec
P1 10.00 usec
SI 32768
SF 125.7628224 MHz
WDW EM
SSB 0
LB 1.00 Hz
GB 0
PC 1.40

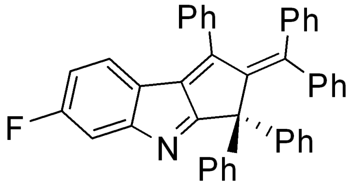


**4f**

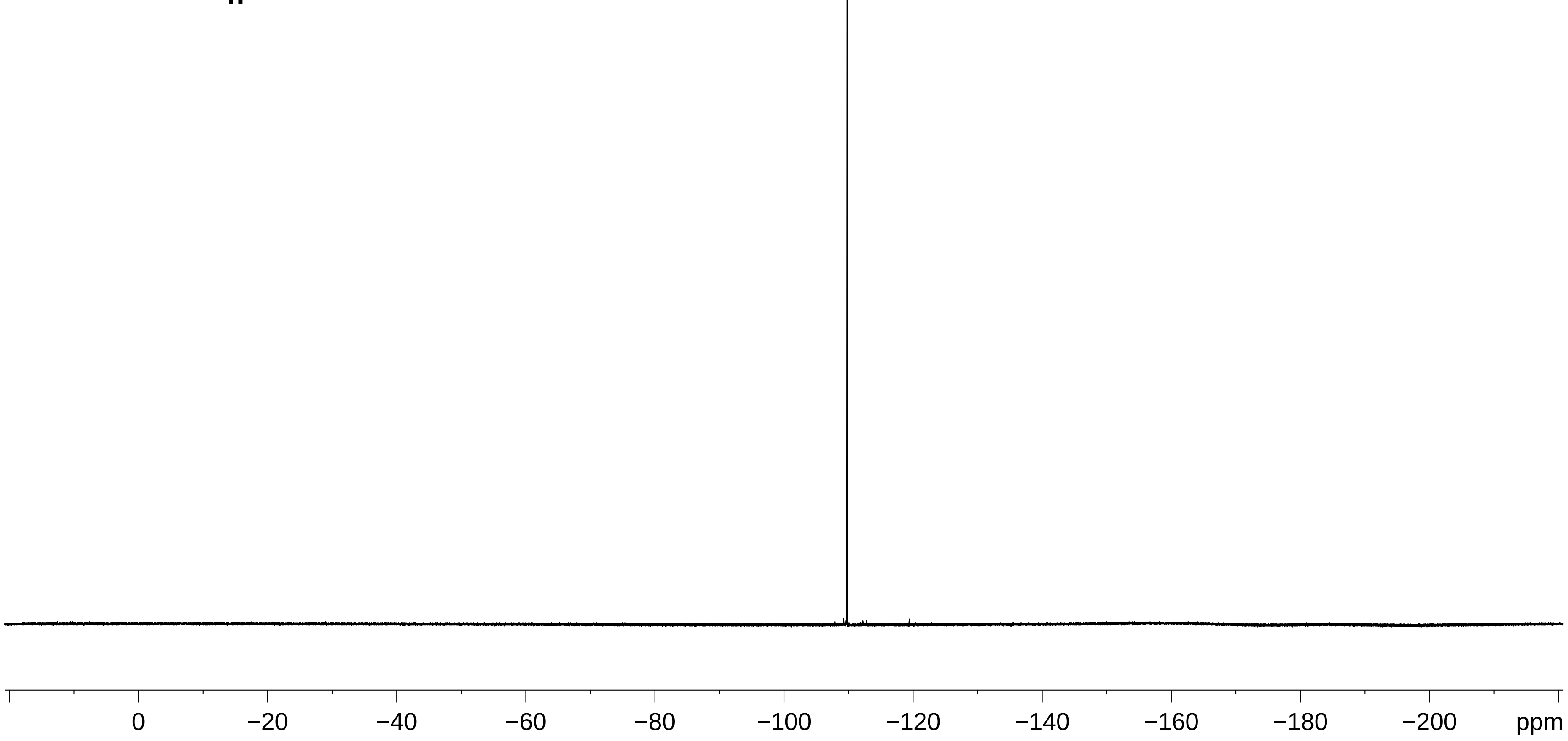


| | |
|---|---|
| NAME | qzw_687_1 |
| EXPNO | 12 |
| PROCNO | 1 |
| Date_ | 20210708 |
| Time | 22.09 h |
| INSTRUM | Avance NEO 500 |
| PROBHD | Z119470_0332 ( |
| PULPROG | zgig |
| TD | 131072 |
| SOLVENT | CDCl3 |
| NS | 4 |
| DS | 4 |
| SWH | 113636.367 Hz |
| FIDRES | 1.733953 Hz |
| AQ | 0.5767668 sec |
| RG | 101 |
| DW | 4.400 usec |
| DE | 6.50 usec |
| TE | 296.2 K |
| D1 | 1.00000000 sec |
| D11 | 0.03000000 sec |
| TD0 | 1 |
| SFO1 | 470.5641349 MHz |
| NUC1 | 19F |
| P1 | 15.00 usec |
| SI | 65536 |
| SF | 470.6111960 MHz |
| WDW | EM |
| SSB | 0 |
| LB | 0.30 Hz |
| GB | 0 |
| PC | 1.00 |

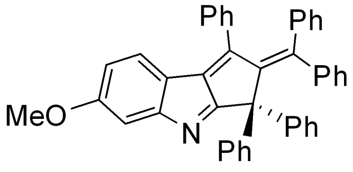


**4g**

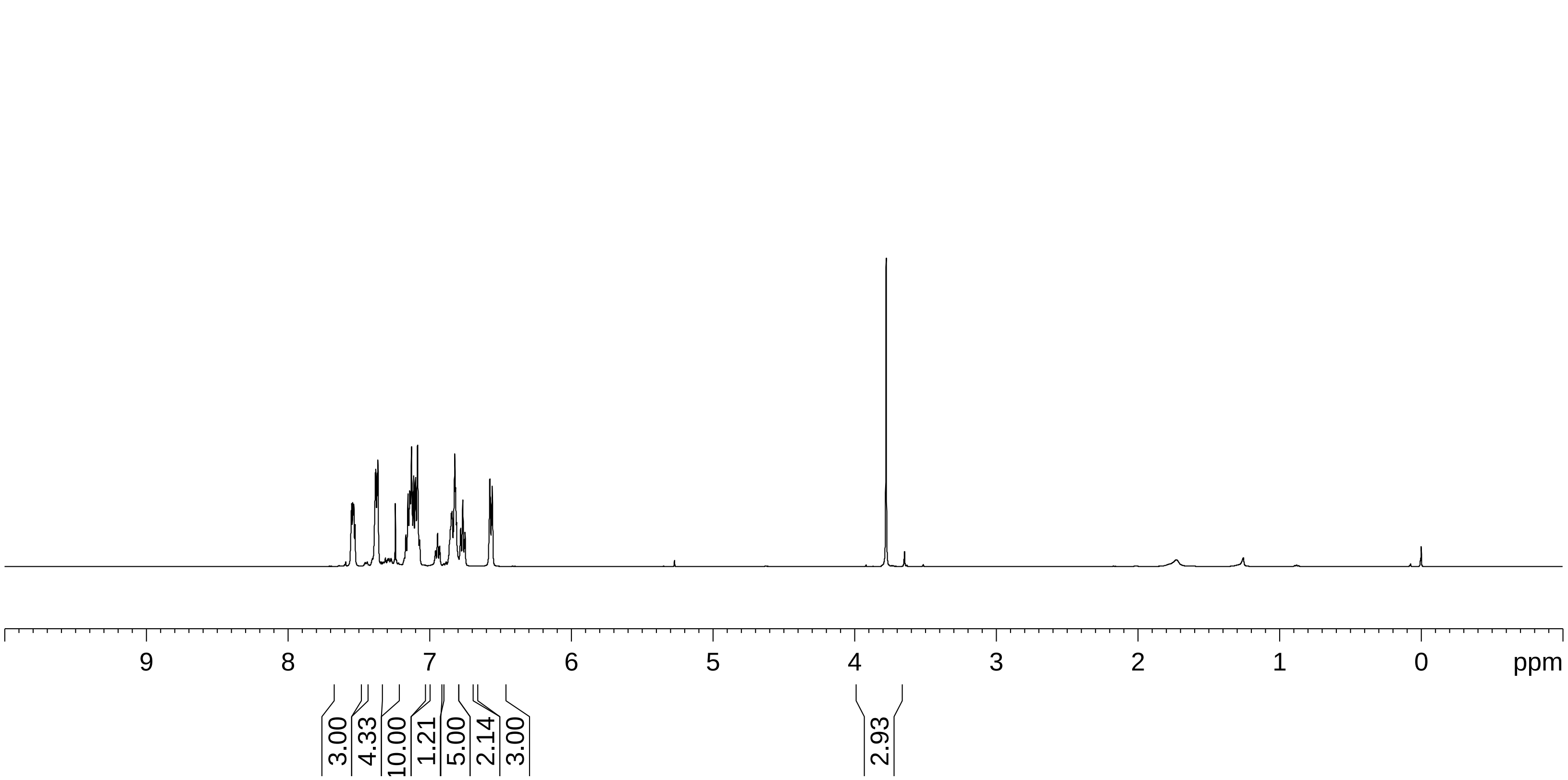


| | |
|---|---|
| NAME | qzw_687_2 |
| EXPNO | 10 |
| PROCNO | 1 |
| Date_ | 20210624 |
| Time | 20.11 h |
| INSTRUM | Avance NEO 500 |
| PROBHD | Z119470_0332 ( |
| PULPROG | zg30 |
| TD | 65536 |
| SOLVENT | CDCl3 |
| NS | 4 |
| DS | 2 |
| SWH | 10000.000 Hz |
| FIDRES | 0.305176 Hz |
| AQ | 3.2768500 sec |
| RG | 101 |
| DW | 50.000 usec |
| DE | 10.84 usec |
| TE | 296.2 K |
| D1 | 1.00000000 sec |
| TD0 | 1 |
| SFO1 | 500.1530884 MHz |
| NUC1 | 1H |
| P0 | 3.24 usec |
| P1 | 9.72 usec |
| SI | 65536 |
| SF | 500.1500206 MHz |
| WDW | EM |
| SSB | 0 |
| LB | 0.30 Hz |
| GB | 0 |
| PC | 1.00 |

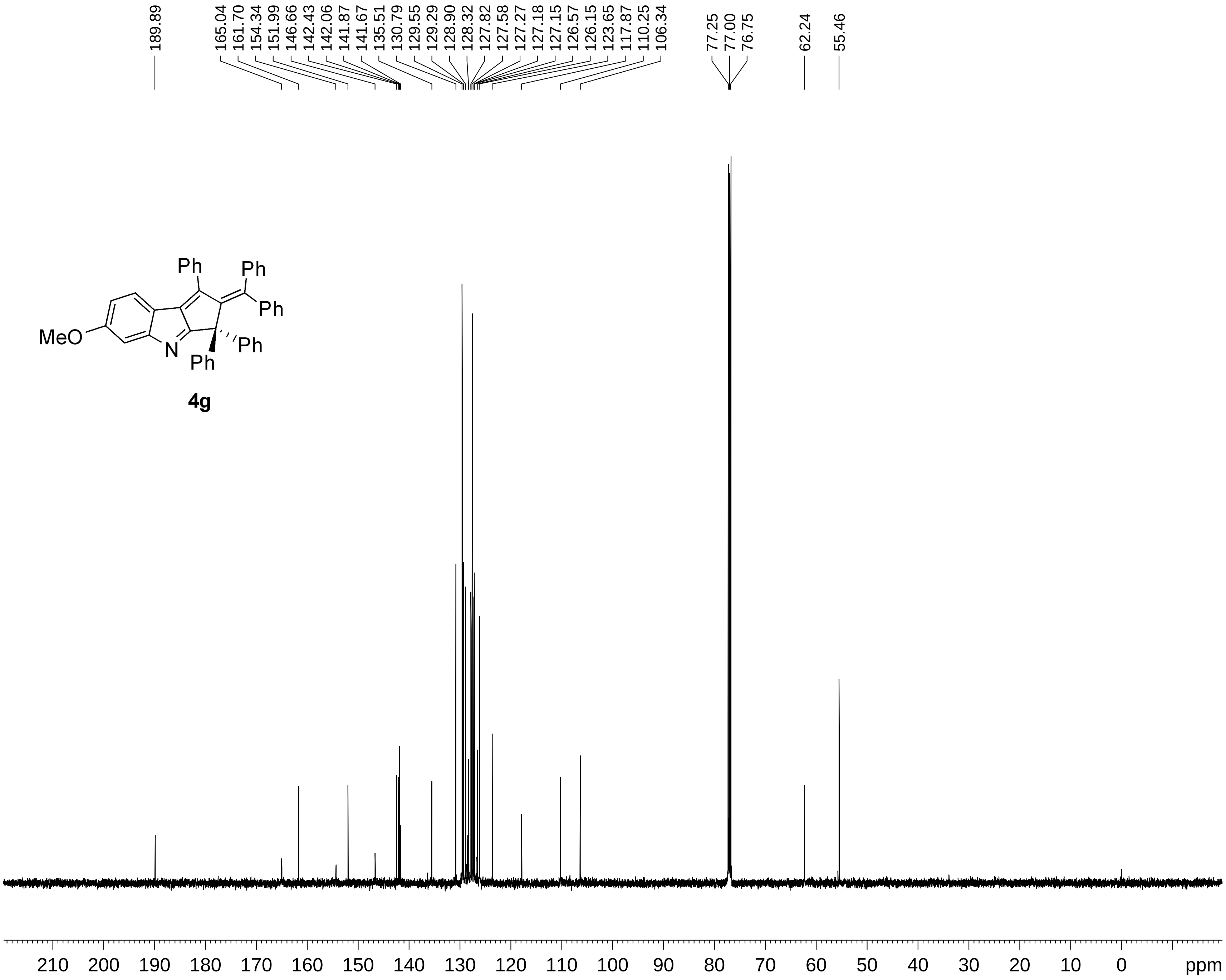


**4g**



| | |
|---|---|
| NAME | qzw_687_2 |
| EXPNO | 11 |
| PROCNO | 1 |
| Date_ | 20210624 |
| Time | 20.29 h |
| INSTRUM | Avance NEO 500 |
| PROBHD | Z119470_0332 ( |
| PULPROG | zgpg30 |
| TD | 65536 |
| SOLVENT | CDCl3 |
| NS | 320 |
| DS | 4 |
| SWH | 30120.482 Hz |
| FIDRES | 0.919204 Hz |
| AQ | 1.0879476 sec |
| RG | 101 |
| DW | 16.600 usec |
| DE | 6.50 usec |
| TE | 296.2 K |
| D1 | 2.00000000 sec |
| D11 | 0.03000000 sec |
| TD0 | 1 |
| SFO1 | 125.7753938 MHz |
| NUC1 | 13C |
| P0 | 3.33 usec |
| P1 | 10.00 usec |
| SI | 32768 |
| SF | 125.7628266 MHz |
| WDW | EM |
| SSB | 0 |
| LB | 1.00 Hz |
| GB | 0 |
| PC | 1.40 |

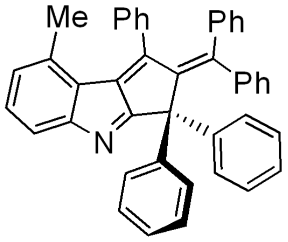
Me
Ph
Ph
Ph
N

**4h**

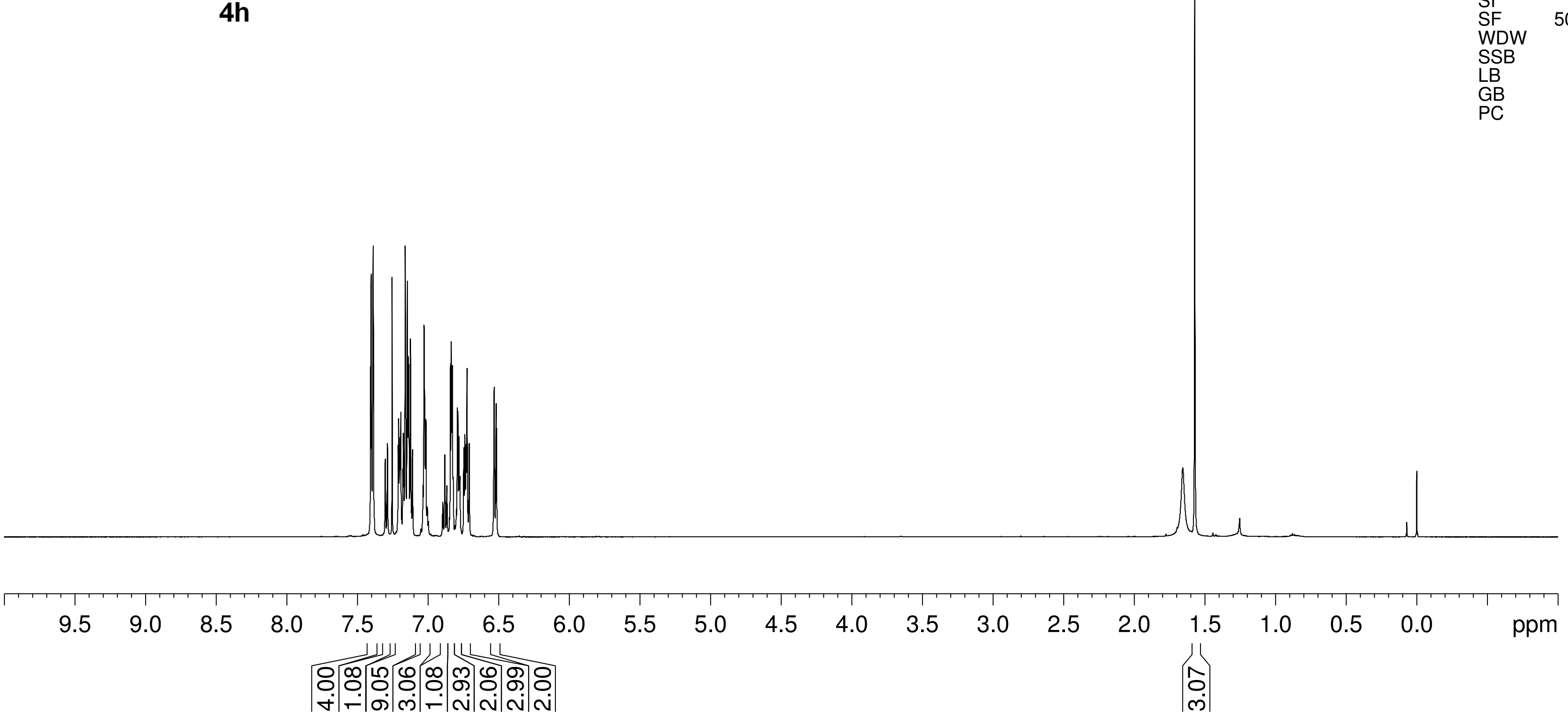
7.404
7.390
7.303
7.288
7.212
7.209
7.199
7.193
7.175
7.162
7.147
7.141
7.138
7.135
7.126
7.111
7.029
7.027
7.015
6.897
6.882
6.867
6.842
6.837
6.829
6.794
6.789
6.782
6.774
6.747
6.740
6.732
6.724
6.709
6.532
6.518
1.572
NAME m_wtf_891_1
EXPNO 10
PROCNO 1
Date_ 20240217
Time 18.14 h
INSTRUM Avance NEO 500
PROBHD Z119470_0332 (
PULPROG zg30
TD 65536
SOLVENT CDCl3
NS 16
DS 2
SWH 10000.000 Hz
FIDRES 0.305176 Hz
AQ 3.2768500 sec
RG 101
DW 50.000 usec
DE 10.84 usec
TE 294.7 K
D1 1.00000000 sec
TD0 1
SFO1 500.1530884 MHz
NUC1 1H
P0 3.24 usec
P1 9.72 usec
SI 65536
SF 500.1500146 MHz
WDW EM
SSB 0
LB 0.30 Hz
GB 0
PC 1.00
9.5
9.0
8.5
8.0
7.5
7.0
6.5
6.0
5.5
5.0
4.5
4.0
3.5
3.0
2.5
2.0
1.5
1.0
0.5
0.0
ppm
4.00
1.08
9.05
3.06
1.08
2.93
2.06
2.99
2.00
3.07

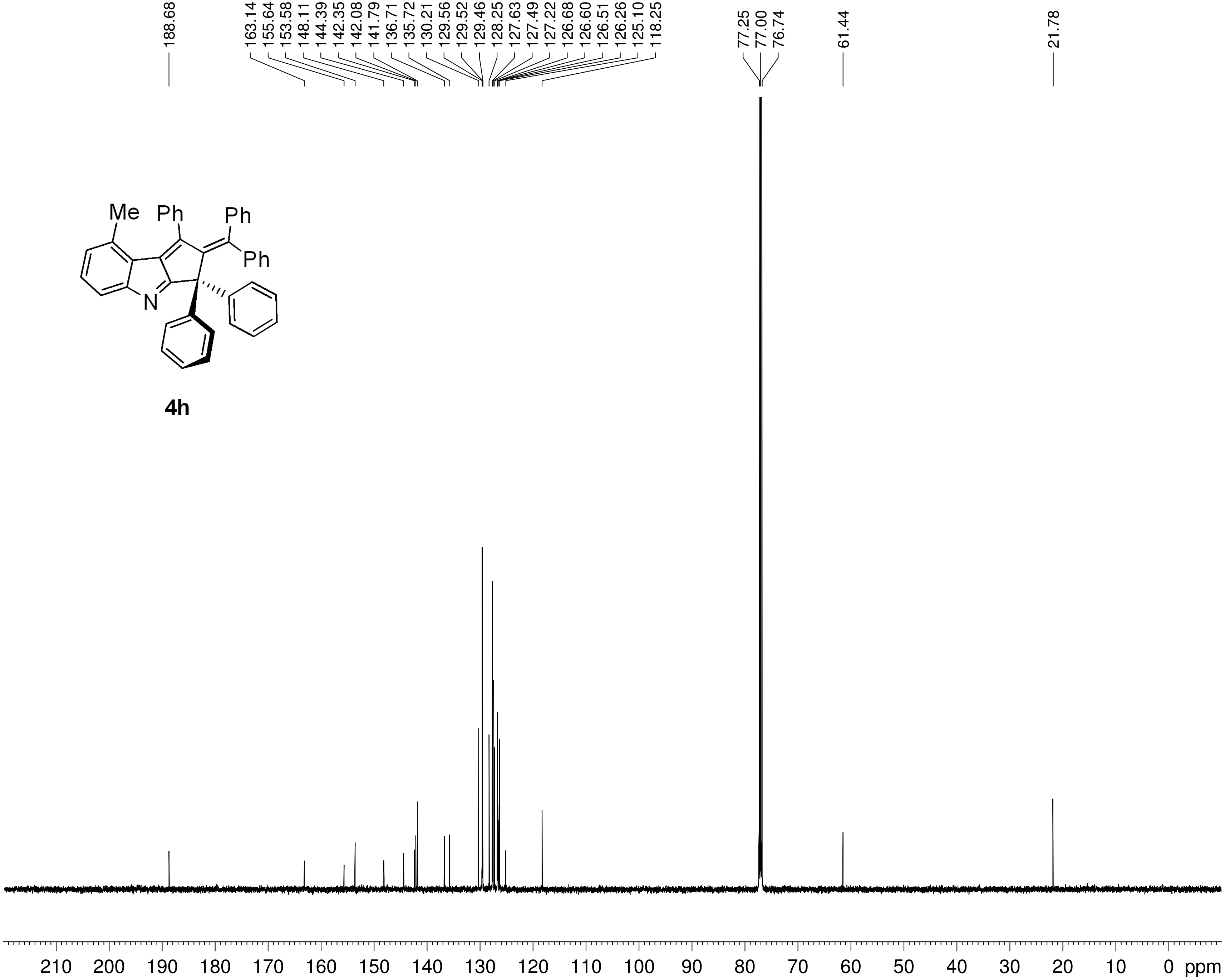


| | |
|---|---|
| NAME | m_wtf_891_1 |
| EXPNO | 11 |
| PROCNO | 1 |
| Date_ | 20240217 |
| Time | 19.04 h |
| INSTRUM | Avance NEO 500 |
| PROBHD | Z119470_0332 ( |
| PULPROG | zgpg30 |
| TD | 65536 |
| SOLVENT | CDCl3 |
| NS | 860 |
| DS | 4 |
| SWH | 30120.482 Hz |
| FIDRES | 0.919204 Hz |
| AQ | 1.0879476 sec |
| RG | 101 |
| DW | 16.600 usec |
| DE | 6.50 usec |
| TE | 295.6 K |
| D1 | 2.00000000 sec |
| D11 | 0.03000000 sec |
| TD0 | 1 |
| SFO1 | 125.7753938 MHz |
| NUC1 | 13C |
| P0 | 3.33 usec |
| P1 | 10.00 usec |
| SI | 32768 |
| SF | 125.7628233 MHz |
| WDW | EM |
| SSB | 0 |
| LB | 1.00 Hz |
| GB | 0 |
| PC | 1.40 |

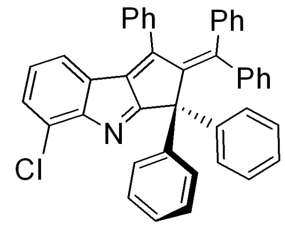


**4i**

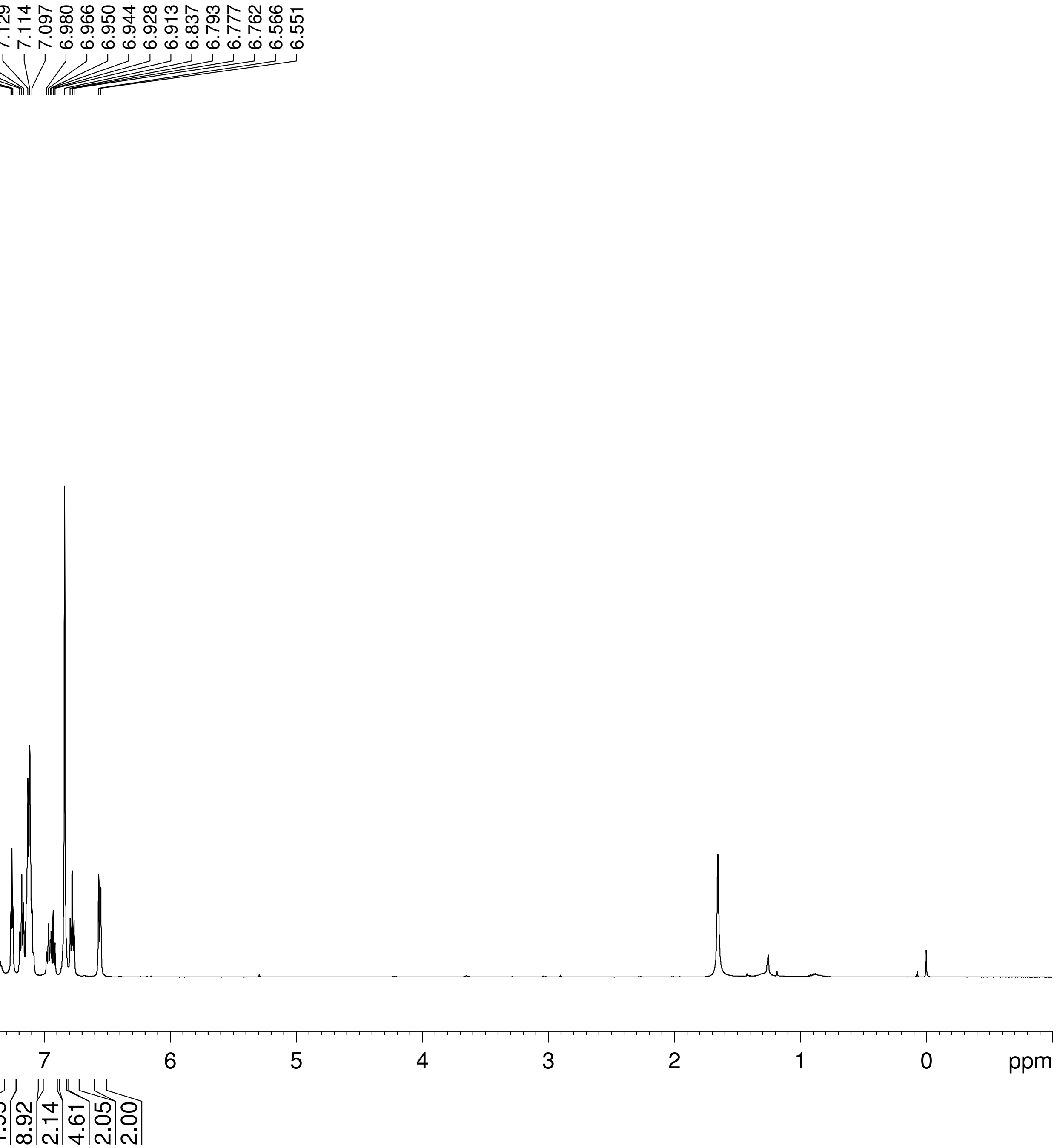


| | |
|---|---|
| NAME | wtf_891_2 |
| EXPNO | 10 |
| PROCNO | 1 |
| Date_ | 20240218 |
| Time | 15.55 h |
| INSTRUM | Avance NEO 500 |
| PROBHD | Z119470_0332 ( |
| PULPROG | zg30 |
| TD | 65536 |
| SOLVENT | CDCl3 |
| NS | 16 |
| DS | 2 |
| SWH | 10000.000 Hz |
| FIDRES | 0.305176 Hz |
| AQ | 3.2768500 sec |
| RG | 101 |
| DW | 50.000 usec |
| DE | 10.84 usec |
| TE | 295.0 K |
| D1 | 1.00000000 sec |
| TD0 | 1 |
| SFO1 | 500.1530884 MHz |
| NUC1 | 1H |
| P0 | 3.24 usec |
| P1 | 9.72 usec |
| SI | 65536 |
| SF | 500.1500144 MHz |
| WDW | EM |
| SSB | 0 |
| LB | 0.30 Hz |
| GB | 0 |
| PC | 1.00 |

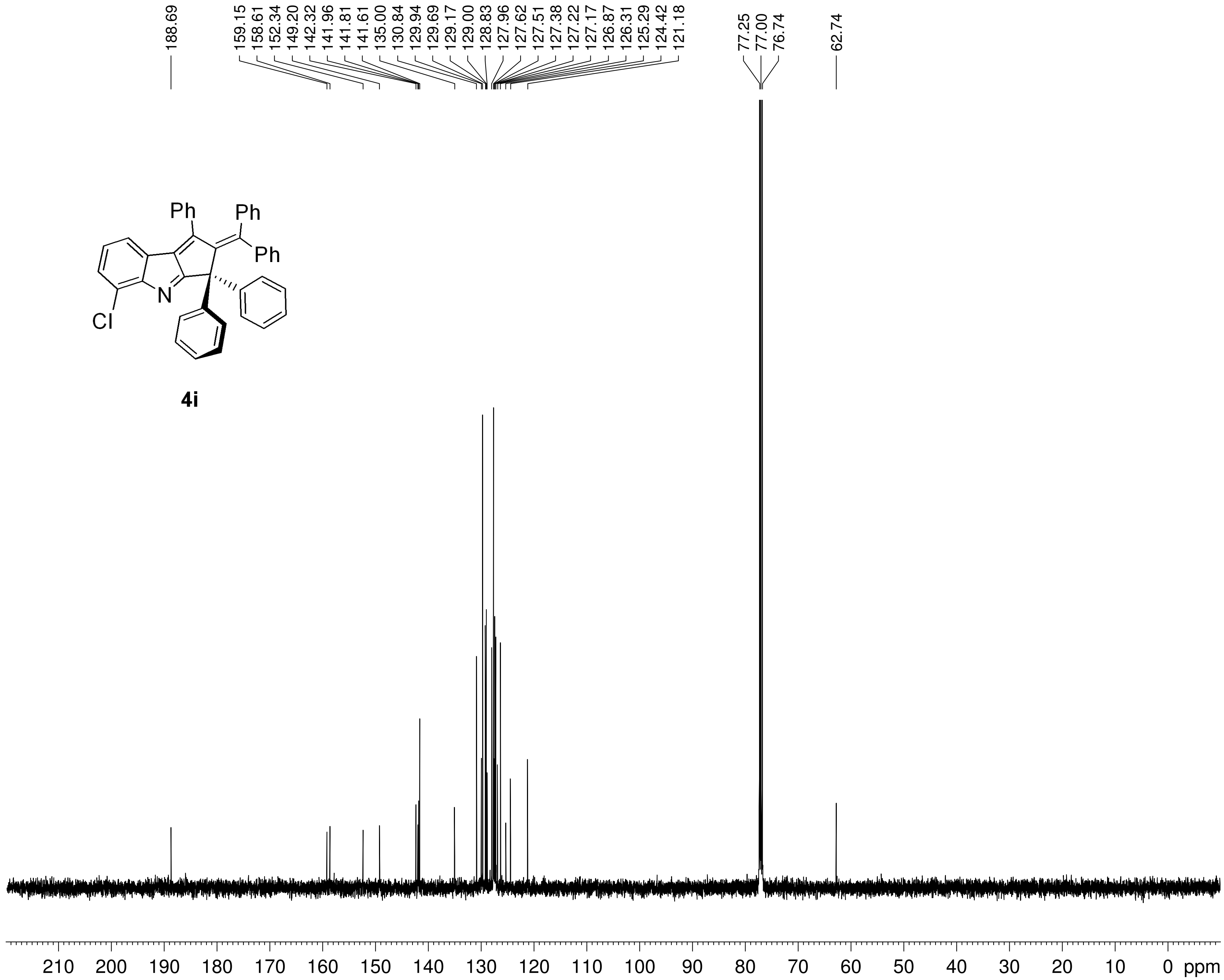


| | |
|---|---|
| NAME | wtf_891_2 |
| EXPNO | 11 |
| PROCNO | 1 |
| Date_ | 20240218 |
| Time | 16.27 h |
| INSTRUM | Avance NEO 500 |
| PROBHD | Z119470_0332 ( |
| PULPROG | zgpg30 |
| TD | 65536 |
| SOLVENT | CDCl3 |
| NS | 400 |
| DS | 4 |
| SWH | 30120.482 Hz |
| FIDRES | 0.919204 Hz |
| AQ | 1.0879476 sec |
| RG | 101 |
| DW | 16.600 usec |
| DE | 6.50 usec |
| TE | 295.8 K |
| D1 | 2.00000000 sec |
| D11 | 0.03000000 sec |
| TD0 | 1 |
| SFO1 | 125.7753938 MHz |
| NUC1 | 13C |
| P0 | 3.33 usec |
| P1 | 10.00 usec |
| SI | 32768 |
| SF | 125.7628231 MHz |
| WDW | EM |
| SSB | 0 |
| LB | 1.00 Hz |
| GB | 0 |
| PC | 1.40 |

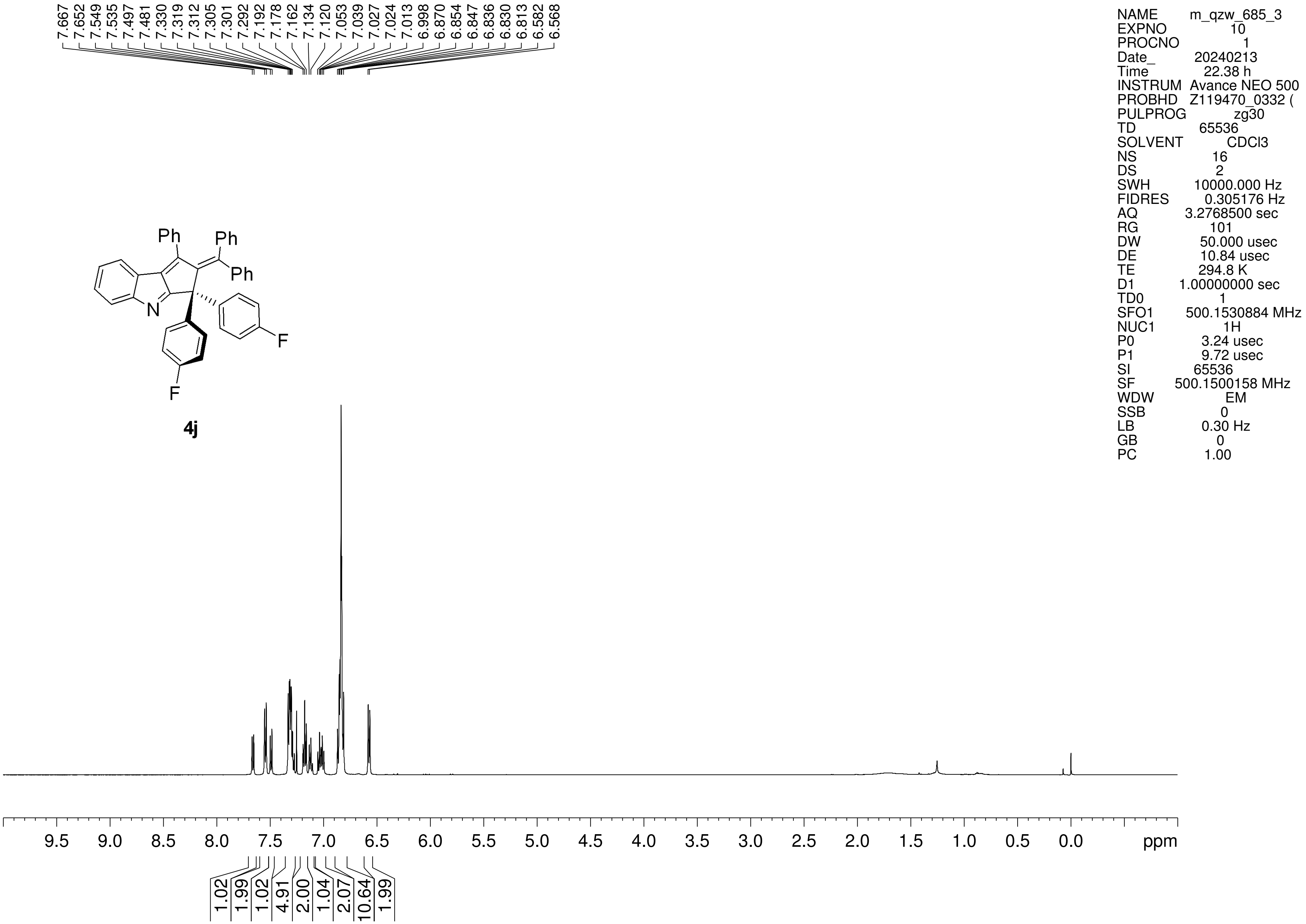
7.667
7.652
7.549
7.535
7.497
7.481
7.330
7.319
7.312
7.305
7.301
7.292
7.192
7.178
7.162
7.134
7.120
7.053
7.039
7.027
7.024
7.013
6.998
6.870
6.854
6.847
6.836
6.830
6.813
6.582
6.568
Ph
Ph
Ph
N
F
F
4j
NAME m_qzw_685_3
EXPNO 10
PROCNO 1
Date_ 20240213
Time 22.38 h
INSTRUM Avance NEO 500
PROBHD Z119470_0332 (
PULPROG zg30
TD 65536
SOLVENT CDCl3
NS 16
DS 2
SWH 10000.000 Hz
FIDRES 0.305176 Hz
AQ 3.2768500 sec
RG 101
DW 50.000 usec
DE 10.84 usec
TE 294.8 K
D1 1.00000000 sec
TD0 1
SFO1 500.1530884 MHz
NUC1 1H
P0 3.24 usec
P1 9.72 usec
SI 65536
SF 500.1500158 MHz
WDW EM
SSB 0
LB 0.30 Hz
GB 0
PC 1.00
9.5 9.0 8.5 8.0 7.5 7.0 6.5 6.0 5.5 5.0 4.5 4.0 3.5 3.0 2.5 2.0 1.5 1.0 0.5 0.0 ppm
1.02
1.99
1.02
4.91
2.00
1.04
2.07
10.64
1.99

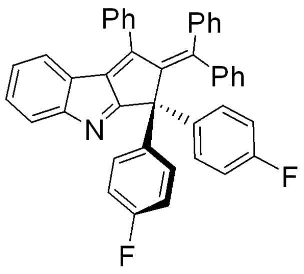


**4j**

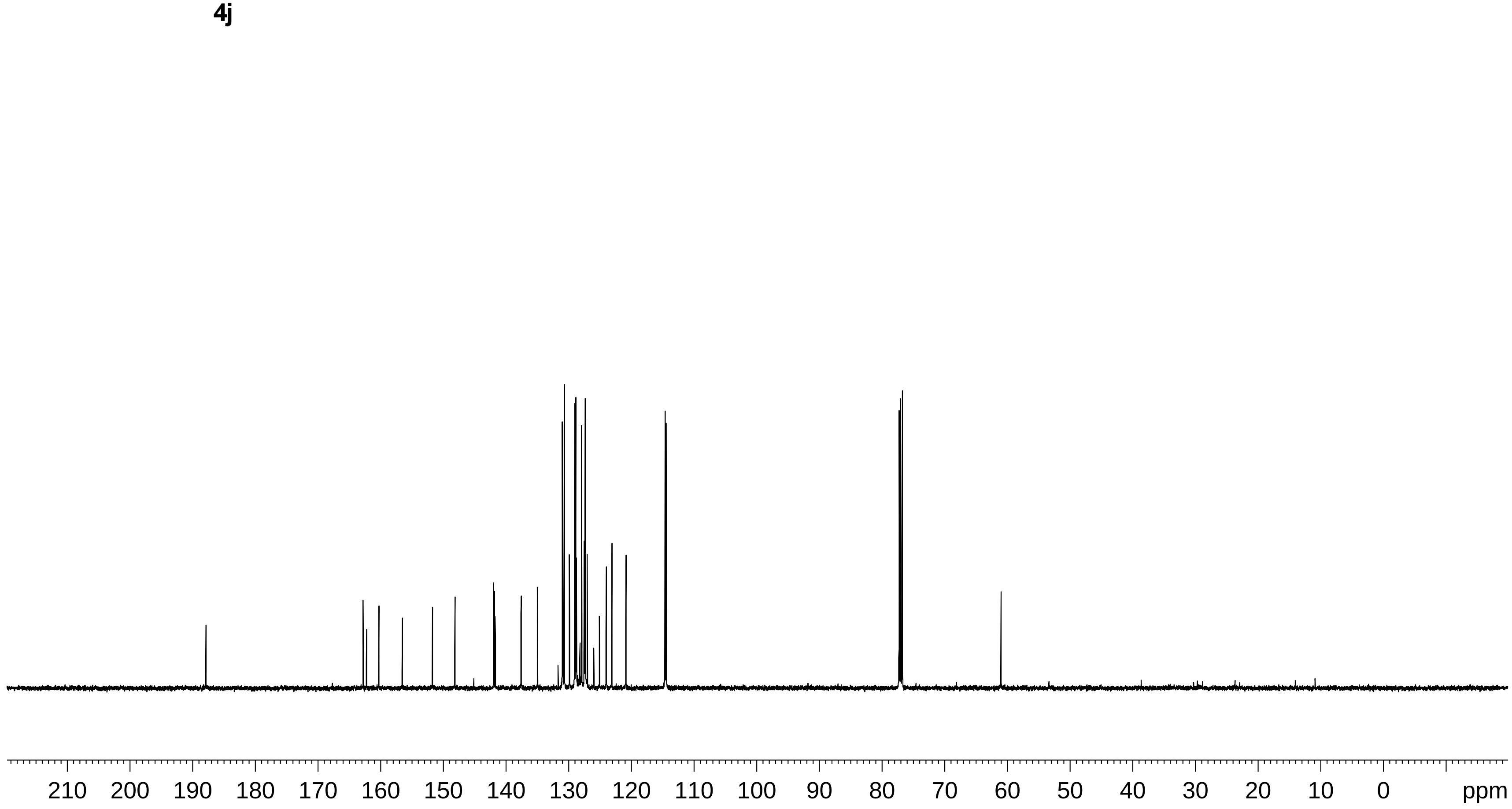


| | |
|---|---|
| NAME | qzw_685_3 |
| EXPNO | 14 |
| PROCNO | 1 |
| Date_ | 20210621 |
| Time | 5.16 h |
| INSTRUM | Avance NEO 500 |
| PROBHD | Z119470_0332 ( |
| PULPROG | zgpg30 |
| TD | 65536 |
| SOLVENT | CDCl3 |
| NS | 200 |
| DS | 4 |
| SWH | 30120.482 Hz |
| FIDRES | 0.919204 Hz |
| AQ | 1.0879476 sec |
| RG | 101 |
| DW | 16.600 usec |
| DE | 6.50 usec |
| TE | 296.1 K |
| D1 | 2.00000000 sec |
| D11 | 0.03000000 sec |
| TD0 | 1 |
| SFO1 | 125.7753938 MHz |
| NUC1 | 13C |
| P0 | 3.33 usec |
| P1 | 10.00 usec |
| SI | 32768 |
| SF | 125.7628339 MHz |
| WDW | EM |
| SSB | 0 |
| LB | 1.00 Hz |
| GB | 0 |
| PC | 1.40 |

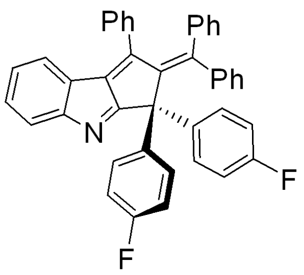


**4j**

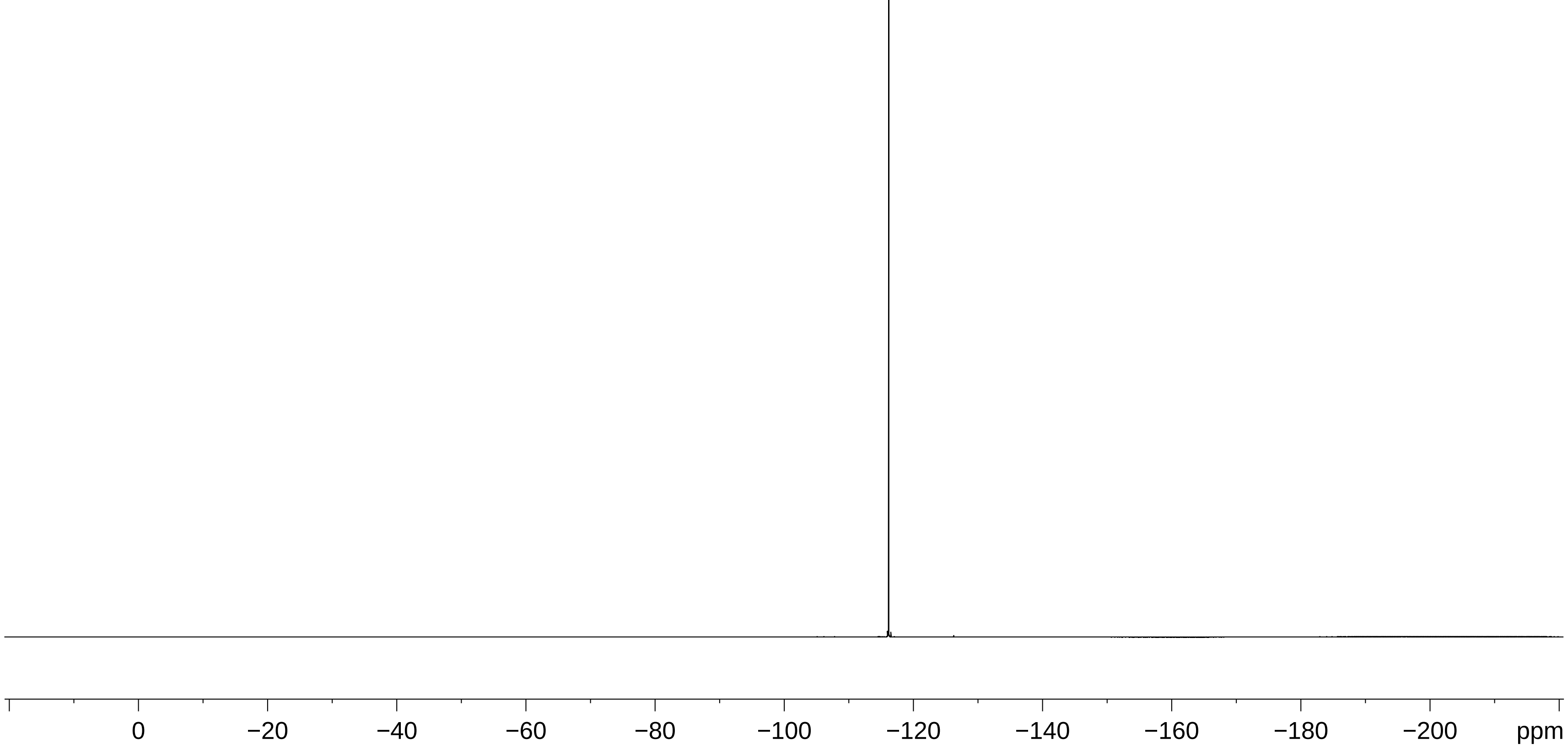


| | |
|---|---|
| NAME | qzw_685_3 |
| EXPNO | 15 |
| PROCNO | 1 |
| Date_ | 20210621 |
| Time | 5.22 h |
| INSTRUM | Avance NEO 500 |
| PROBHD | Z119470_0332 ( |
| PULPROG | zgig |
| TD | 131072 |
| SOLVENT | CDCl3 |
| NS | 4 |
| DS | 4 |
| SWH | 113636.367 Hz |
| FIDRES | 1.733953 Hz |
| AQ | 0.5767668 sec |
| RG | 101 |
| DW | 4.400 usec |
| DE | 6.50 usec |
| TE | 296.2 K |
| D1 | 1.00000000 sec |
| D11 | 0.03000000 sec |
| TD0 | 1 |
| SFO1 | 470.5641349 MHz |
| NUC1 | 19F |
| P1 | 15.00 usec |
| SI | 65536 |
| SF | 470.6111960 MHz |
| WDW | EM |
| SSB | 0 |
| LB | 0.30 Hz |
| GB | 0 |
| PC | 1.00 |

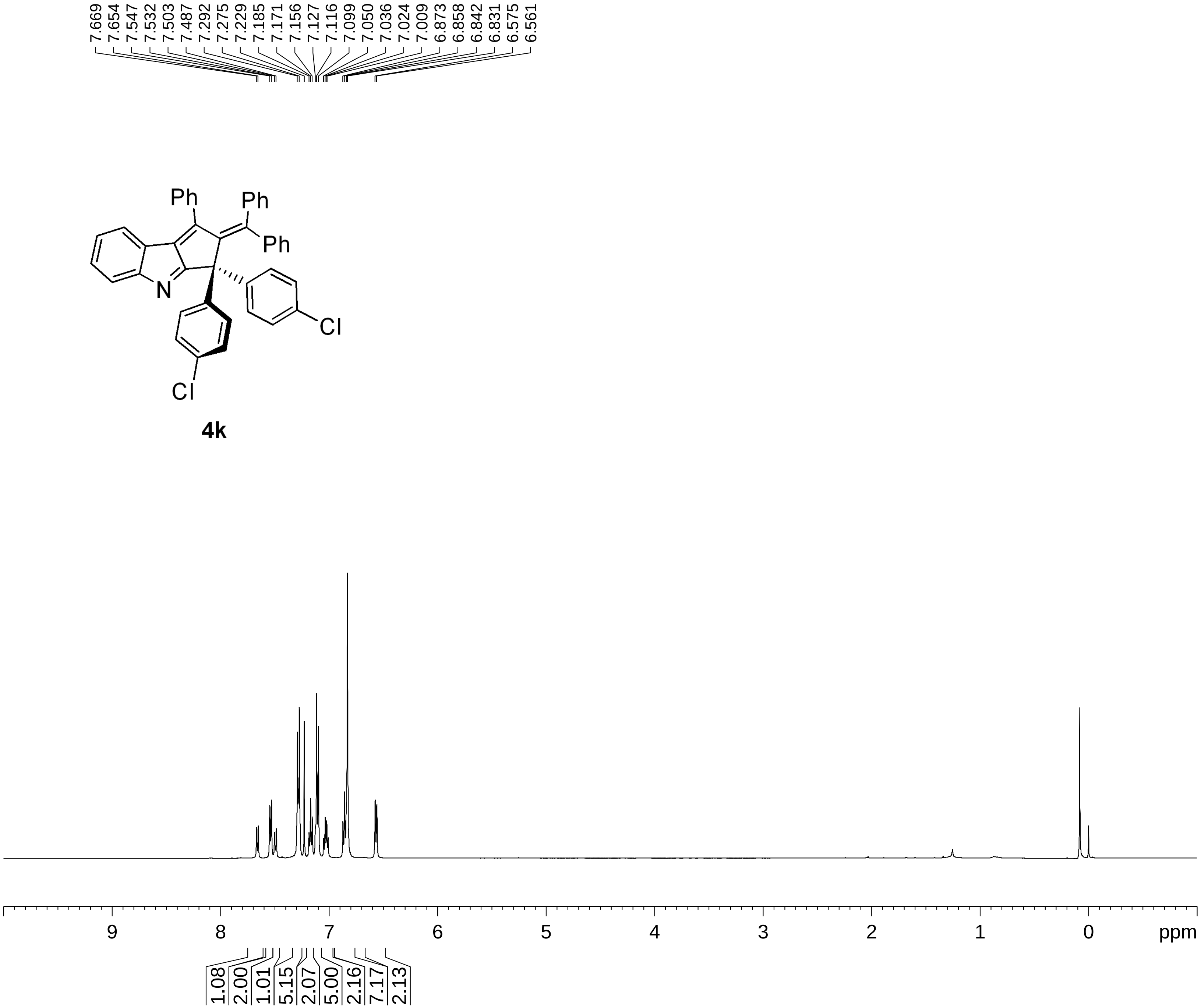


| | |
|---|---|
| NAME | qzw_684_3 |
| EXPNO | 30 |
| PROCNO | 1 |
| Date_ | 20210619 |
| Time | 20.06 h |
| INSTRUM | Avance NEO 500 |
| PROBHD | Z119470_0332 ( |
| PULPROG | zg30 |
| TD | 65536 |
| SOLVENT | CDCl3 |
| NS | 8 |
| DS | 2 |
| SWH | 10000.000 Hz |
| FIDRES | 0.305176 Hz |
| AQ | 3.2768500 sec |
| RG | 67.0968 |
| DW | 50.000 usec |
| DE | 10.84 usec |
| TE | 296.2 K |
| D1 | 1.00000000 sec |
| TD0 | 1 |
| SFO1 | 500.1530884 MHz |
| NUC1 | 1H |
| P0 | 3.24 usec |
| P1 | 9.72 usec |
| SI | 65536 |
| SF | 500.1500273 MHz |
| WDW | EM |
| SSB | 0 |
| LB | 0.30 Hz |
| GB | 0 |
| PC | 1.00 |

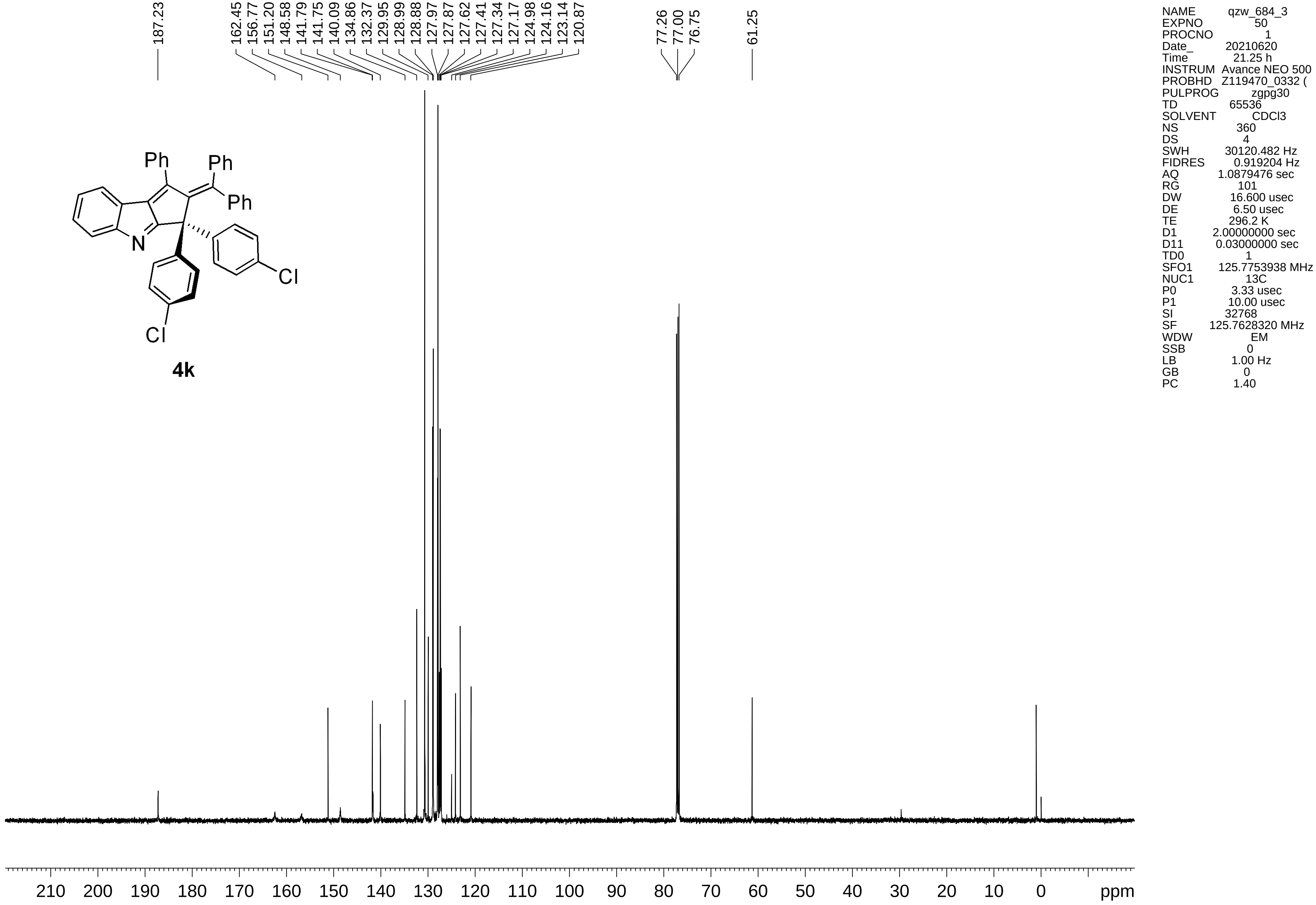
187.23
162.45
156.77
151.20
148.58
141.79
141.75
140.09
134.86
132.37
129.95
128.99
128.88
127.97
127.87
127.62
127.41
127.34
127.17
124.98
124.16
123.14
120.87
77.26
77.00
76.75
61.25
Ph
Ph
Ph
N
Cl
Cl
4k
NAME qzw_684_3
EXPNO 50
PROCNO 1
Date_ 20210620
Time 21.25 h
INSTRUM Avance NEO 500
PROBHD Z119470_0332 (
PULPROG zgpg30
TD 65536
SOLVENT CDCl3
NS 360
DS 4
SWH 30120.482 Hz
FIDRES 0.919204 Hz
AQ 1.0879476 sec
RG 101
DW 16.600 usec
DE 6.50 usec
TE 296.2 K
D1 2.00000000 sec
D11 0.03000000 sec
TD0 1
SFO1 125.7753938 MHz
NUC1 13C
P0 3.33 usec
P1 10.00 usec
SI 32768
SF 125.7628320 MHz
WDW EM
SSB 0
LB 1.00 Hz
GB 0
PC 1.40
210 200 190 180 170 160 150 140 130 120 110 100 90 80 70 60 50 40 30 20 10 0 ppm

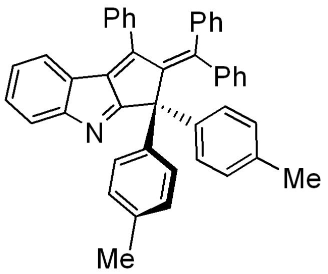


**4l**

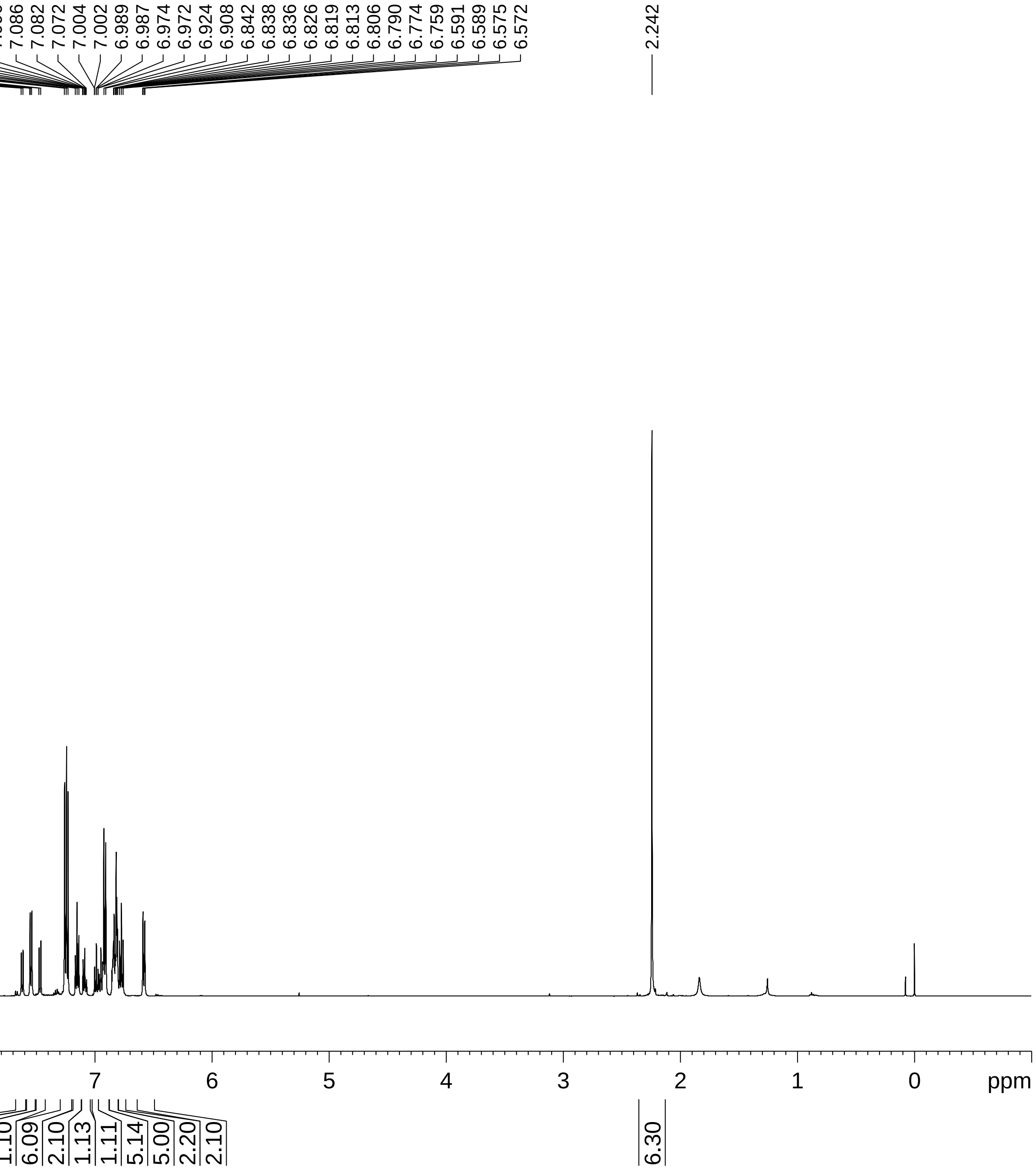


NAME qzw_684_2
EXPNO 40
PROCNO 1
Date_ 20210625
Time 5.05 h
INSTRUM Avance NEO 500
PROBHD Z119470_0332 (
PULPROG zg30
TD 65536
SOLVENT CDCl3
NS 8
DS 2
SWH 10000.000 Hz
FIDRES 0.305176 Hz
AQ 3.2768500 sec
RG 80
DW 50.000 usec
DE 10.84 usec
TE 296.2 K
D1 1.00000000 sec
TD0 1
SFO1 500.1530884 MHz
NUC1 1H
P0 3.24 usec
P1 9.72 usec
SI 65536
SF 500.1500268 MHz
WDW EM
SSB 0
LB 0.30 Hz
GB 0
PC 1.00

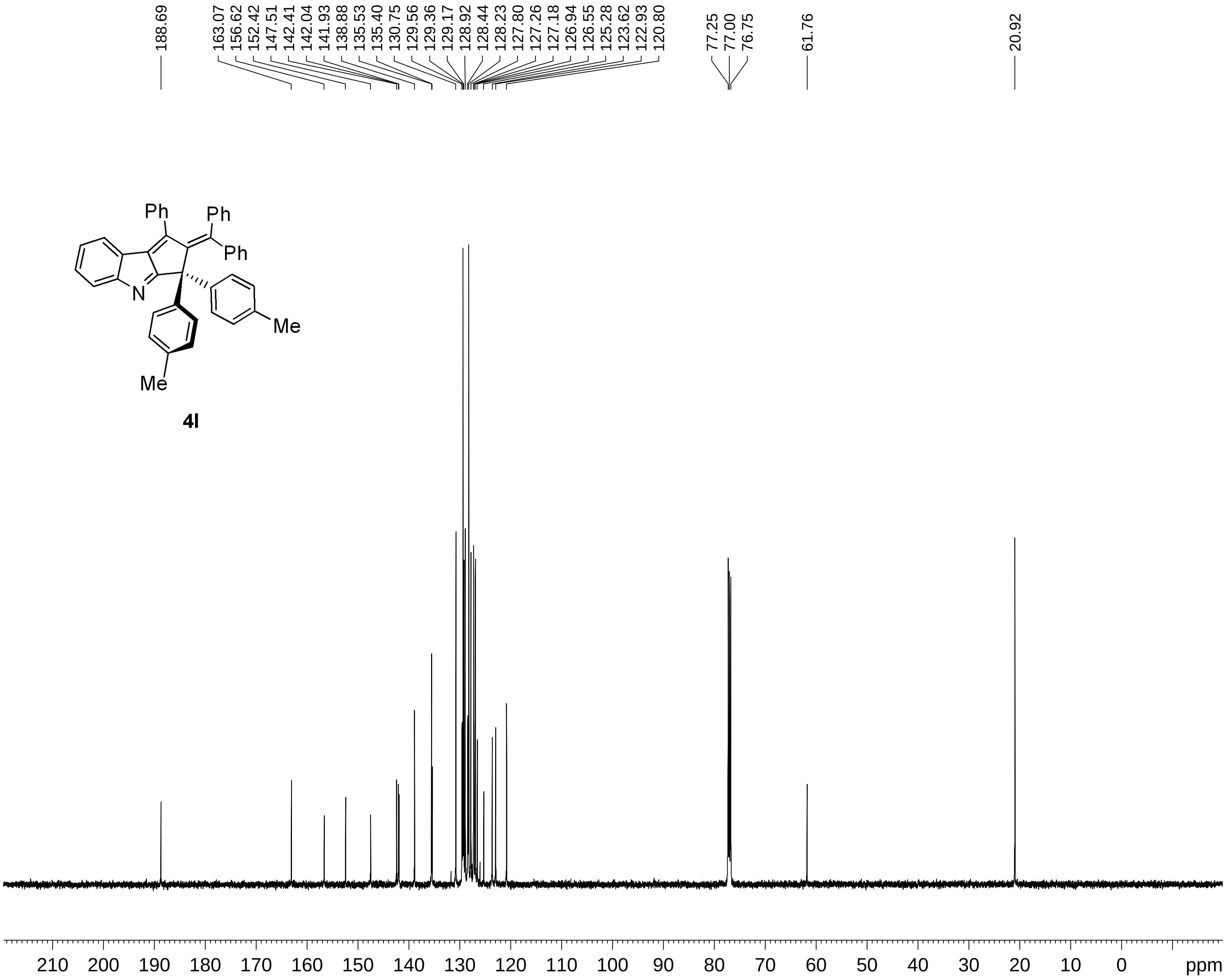


**4l**

| | |
|---|---|
| NAME | qzw_684_2 |
| EXPNO | 31 |
| PROCNO | 1 |
| Date_ | 20210621 |
| Time | 19.14 h |
| INSTRUM | Avance NEO 500 |
| PROBHD | Z119470_0332 ( |
| PULPROG | zgpg30 |
| TD | 65536 |
| SOLVENT | CDCl3 |
| NS | 400 |
| DS | 4 |
| SWH | 30120.482 Hz |
| FIDRES | 0.919204 Hz |
| AQ | 1.0879476 sec |
| RG | 101 |
| DW | 16.600 usec |
| DE | 6.50 usec |
| TE | 296.2 K |
| D1 | 2.00000000 sec |
| D11 | 0.03000000 sec |
| TD0 | 1 |
| SFO1 | 125.7753938 MHz |
| NUC1 | 13C |
| P0 | 3.33 usec |
| P1 | 10.00 usec |
| SI | 32768 |
| SF | 125.7628334 MHz |
| WDW | EM |
| SSB | 0 |
| LB | 1.00 Hz |
| GB | 0 |
| PC | 1.40 |

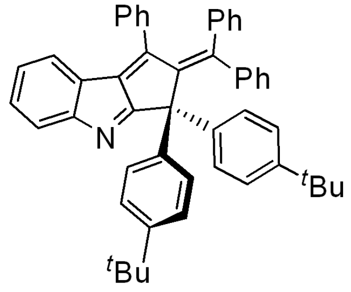


**4m**

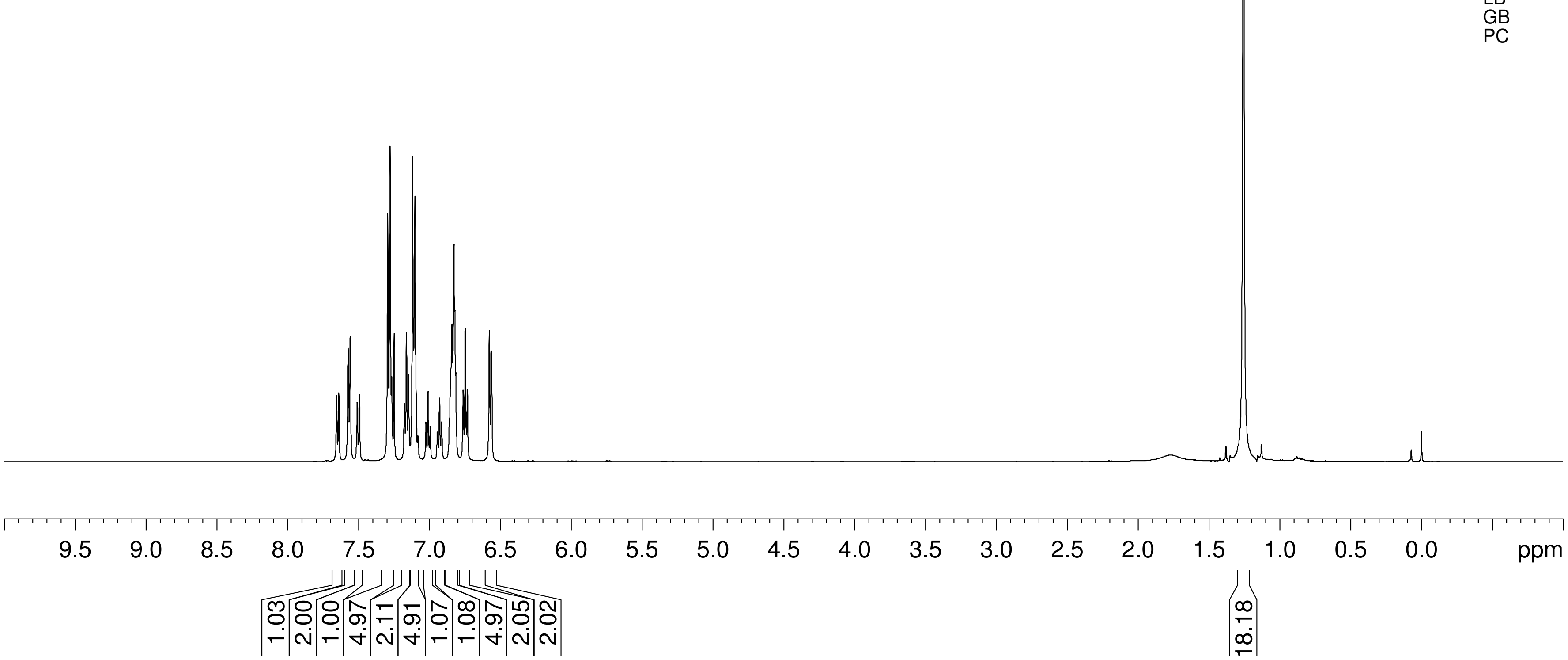


| | |
|---|---|
| NAME | m_qzw_684_4 |
| EXPNO | 10 |
| PROCNO | 1 |
| Date_ | 20240213 |
| Time | 22.11 h |
| INSTRUM | Avance NEO 500 |
| PROBHD | Z119470_0332 ( |
| PULPROG | zg30 |
| TD | 65536 |
| SOLVENT | CDCl3 |
| NS | 16 |
| DS | 2 |
| SWH | 10000.000 Hz |
| FIDRES | 0.305176 Hz |
| AQ | 3.2768500 sec |
| RG | 101 |
| DW | 50.000 usec |
| DE | 10.84 usec |
| TE | 294.4 K |
| D1 | 1.00000000 sec |
| TD0 | 1 |
| SFO1 | 500.1530884 MHz |
| NUC1 | 1H |
| P0 | 3.24 usec |
| P1 | 9.72 usec |
| SI | 65536 |
| SF | 500.1500170 MHz |
| WDW | EM |
| SSB | 0 |
| LB | 0.30 Hz |
| GB | 0 |
| PC | 1.00 |

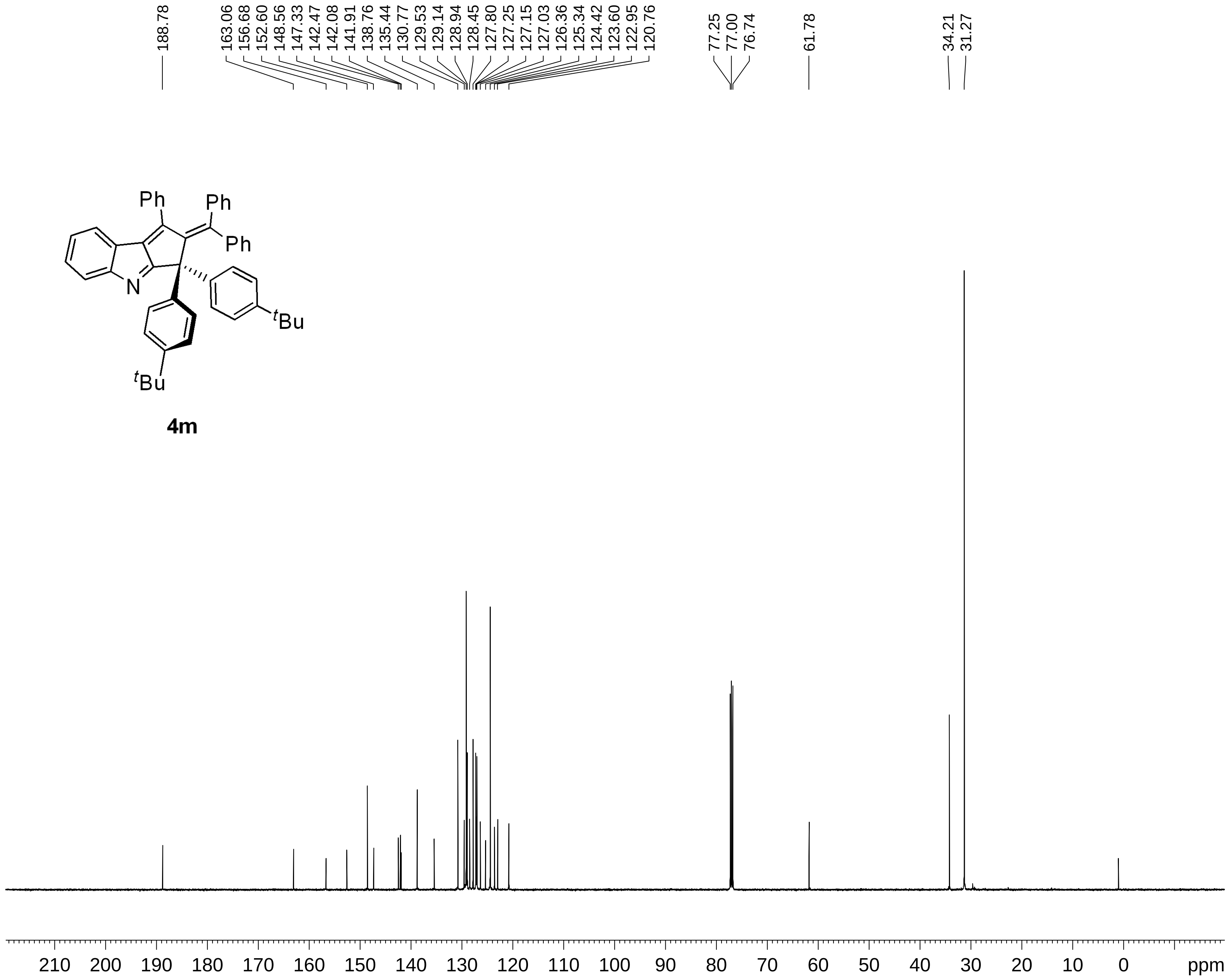


| | |
|---|---|
| NAME | qzw_684_4 |
| EXPNO | 80 |
| PROCNO | 1 |
| Date_ | 20210709 |
| Time | 7.51 h |
| INSTRUM | Avance NEO 500 |
| PROBHD | Z119470_0332 ( |
| PULPROG | zgpg30 |
| TD | 65536 |
| SOLVENT | CDCl3 |
| NS | 480 |
| DS | 4 |
| SWH | 30120.482 Hz |
| FIDRES | 0.919204 Hz |
| AQ | 1.0879476 sec |
| RG | 101 |
| DW | 16.600 usec |
| DE | 6.50 usec |
| TE | 296.1 K |
| D1 | 2.00000000 sec |
| D11 | 0.03000000 sec |
| TD0 | 1 |
| SFO1 | 125.7753938 MHz |
| NUC1 | 13C |
| P0 | 3.33 usec |
| P1 | 10.00 usec |
| SI | 32768 |
| SF | 125.7628334 MHz |
| WDW | EM |
| SSB | 0 |
| LB | 1.00 Hz |
| GB | 0 |
| PC | 1.40 |

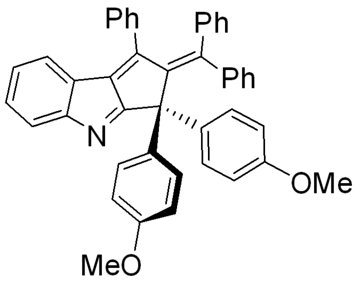


**4n**

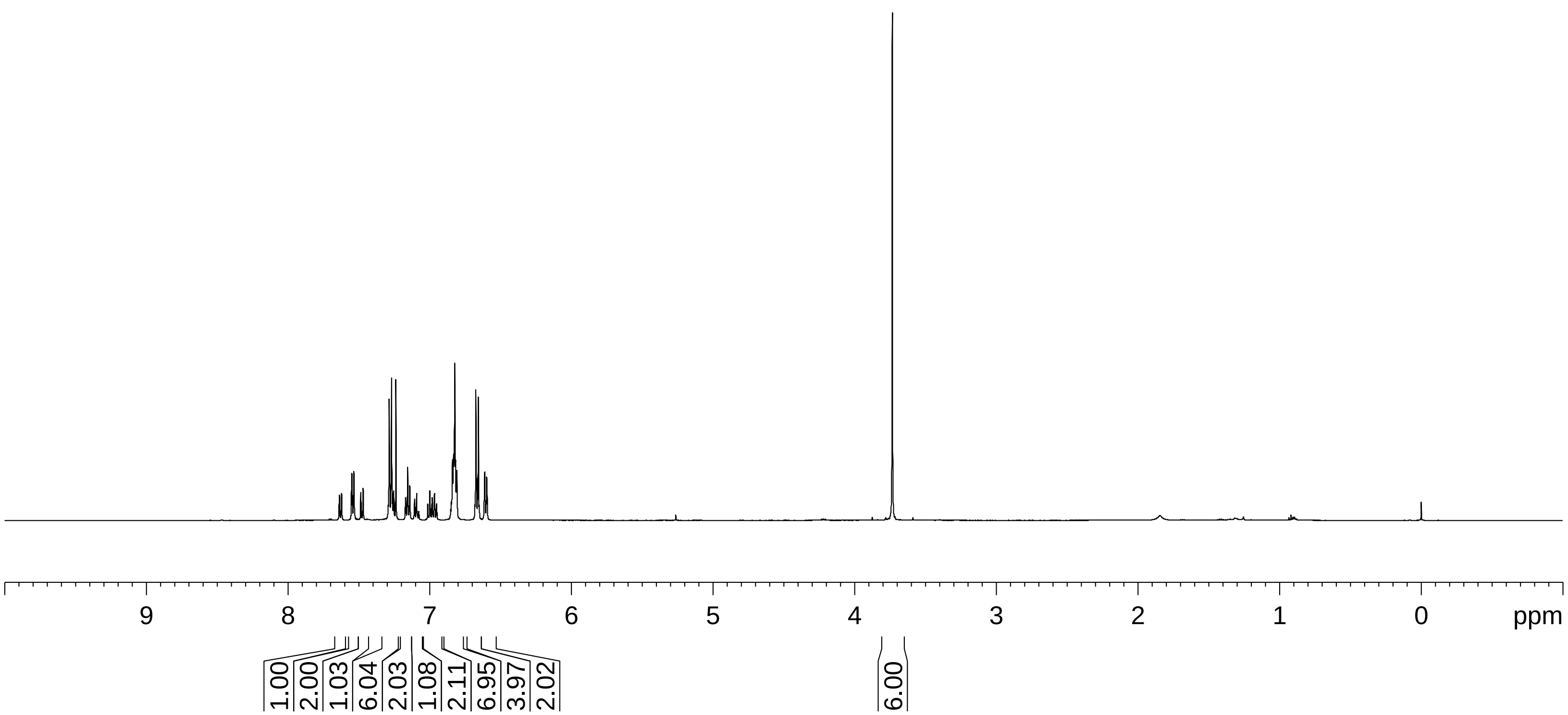


| | |
|---|---|
| NAME | qzw_684_1 |
| EXPNO | 10 |
| PROCNO | 1 |
| Date_ | 20210620 |
| Time | 6.41 h |
| INSTRUM | Avance NEO 500 |
| PROBHD | Z119470_0332 ( |
| PULPROG | zg30 |
| TD | 65536 |
| SOLVENT | CDCl3 |
| NS | 8 |
| DS | 2 |
| SWH | 10000.000 Hz |
| FIDRES | 0.305176 Hz |
| AQ | 3.2768500 sec |
| RG | 83.2 |
| DW | 50.000 usec |
| DE | 10.84 usec |
| TE | 296.1 K |
| D1 | 1.00000000 sec |
| TD0 | 1 |
| SFO1 | 500.1530884 MHz |
| NUC1 | 1H |
| P0 | 3.24 usec |
| P1 | 9.72 usec |
| SI | 65536 |
| SF | 500.1500226 MHz |
| WDW | EM |
| SSB | 0 |
| LB | 0.30 Hz |
| GB | 0 |
| PC | 1.00 |

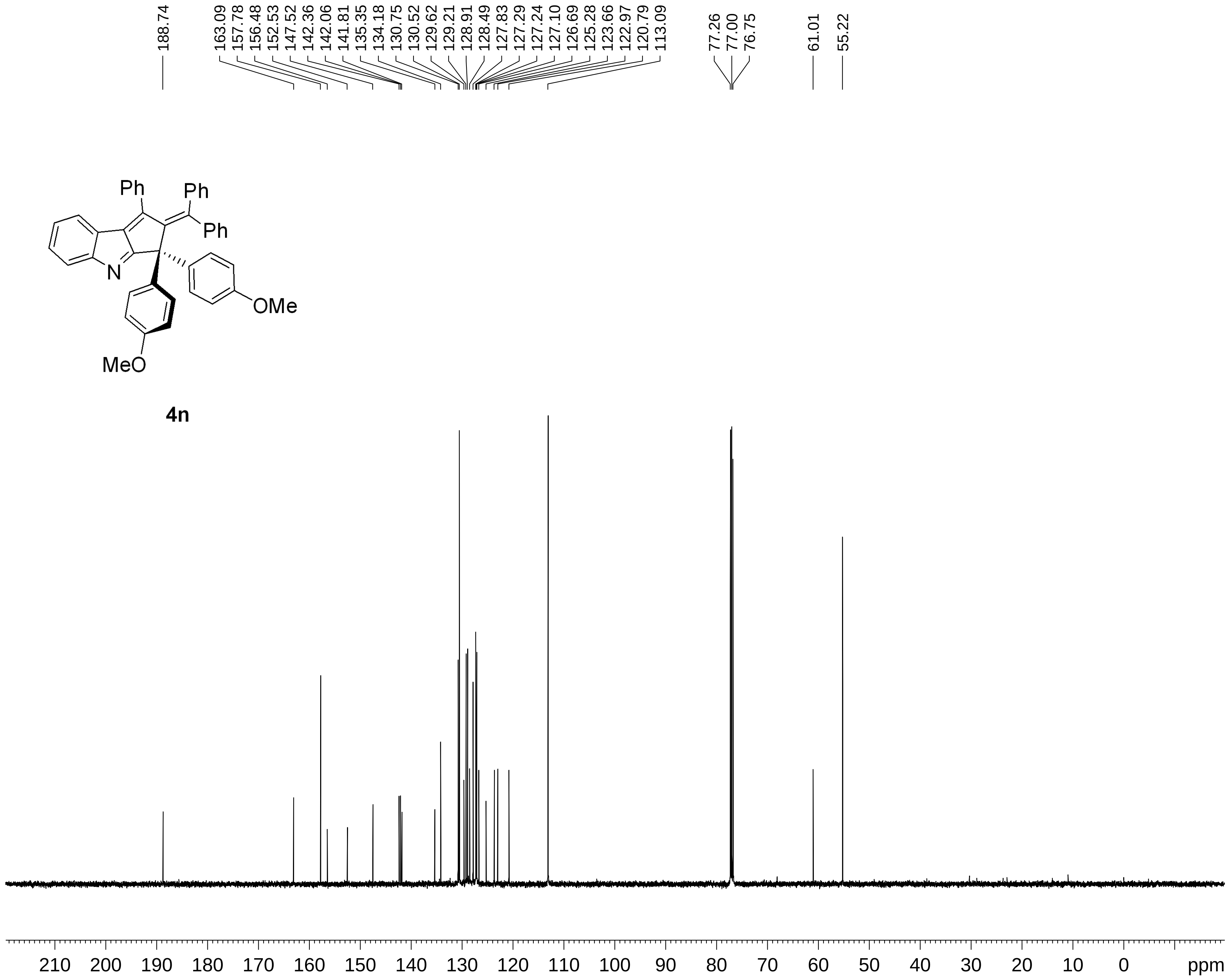


| | |
|---|---|
| NAME | qzw_684_1 |
| EXPNO | 11 |
| PROCNO | 1 |
| Date_ | 20210620 |
| Time | 6.52 h |
| INSTRUM | Avance NEO 500 |
| PROBHD | Z119470_0332 ( |
| PULPROG | zgpg30 |
| TD | 65536 |
| SOLVENT | CDCl3 |
| NS | 200 |
| DS | 4 |
| SWH | 30120.482 Hz |
| FIDRES | 0.919204 Hz |
| AQ | 1.0879476 sec |
| RG | 101 |
| DW | 16.600 usec |
| DE | 6.50 usec |
| TE | 296.1 K |
| D1 | 2.00000000 sec |
| D11 | 0.03000000 sec |
| TD0 | 1 |
| SFO1 | 125.7753938 MHz |
| NUC1 | 13C |
| P0 | 3.33 usec |
| P1 | 10.00 usec |
| SI | 32768 |
| SF | 125.7628288 MHz |
| WDW | EM |
| SSB | 0 |
| LB | 1.00 Hz |
| GB | 0 |
| PC | 1.40 |

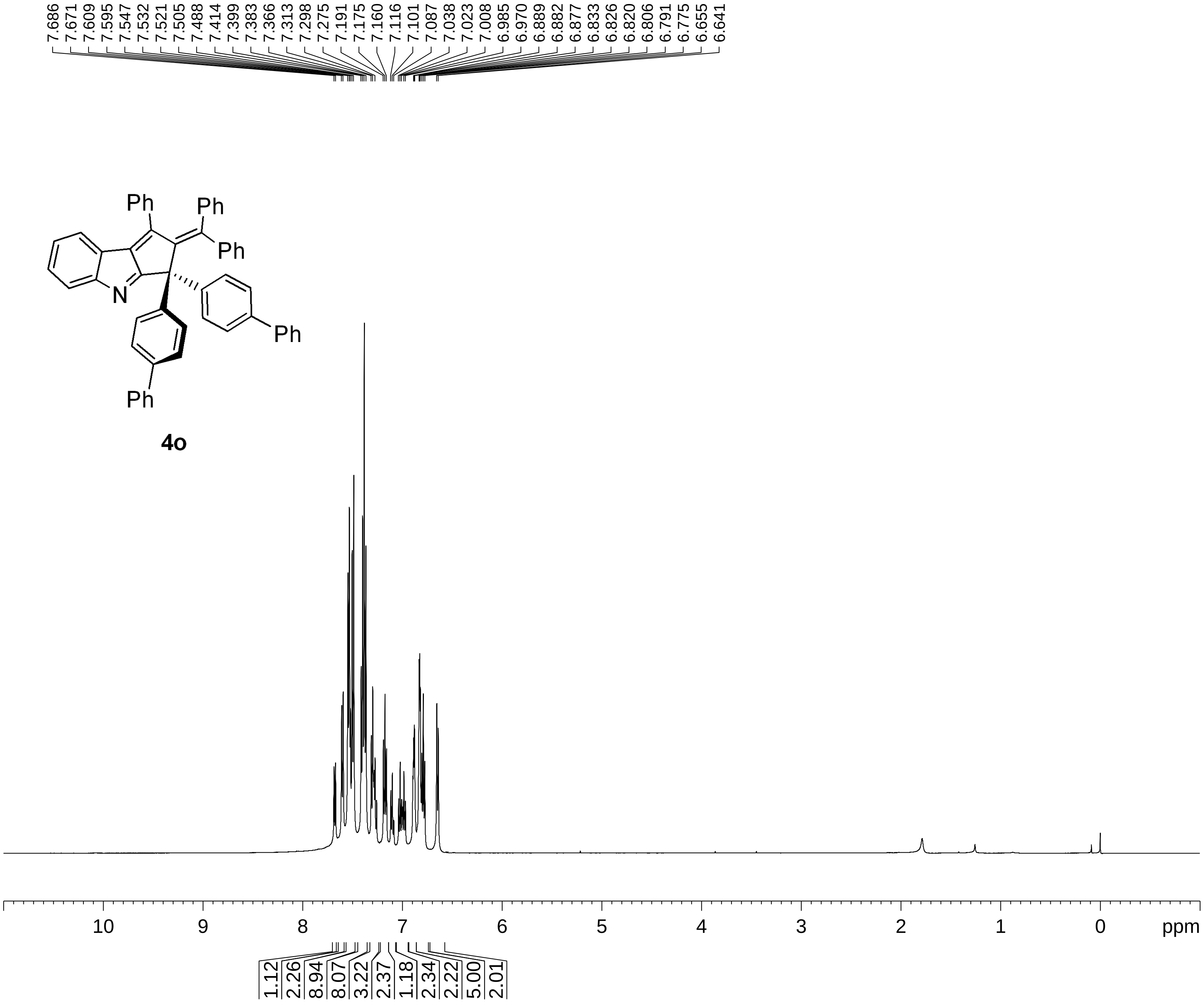

**4o**



NAME qzw_685_2
EXPNO 20
PROCNO 1
Date_ 20210621
Time 19.18 h
INSTRUM Avance NEO 500
PROBHD Z119470_0332 (
PULPROG zg30
TD 65536
SOLVENT CDCl3
NS 4
DS 2
SWH 10000.000 Hz
FIDRES 0.305176 Hz
AQ 3.2768500 sec
RG 61.1765
DW 50.000 usec
DE 10.84 usec
TE 296.1 K
D1 1.00000000 sec
TD0 1
SFO1 500.1530884 MHz
NUC1 1H
P0 3.24 usec
P1 9.72 usec
SI 65536
SF 500.1500468 MHz
WDW EM
SSB 0
LB 0.30 Hz
GB 0
PC 1.00

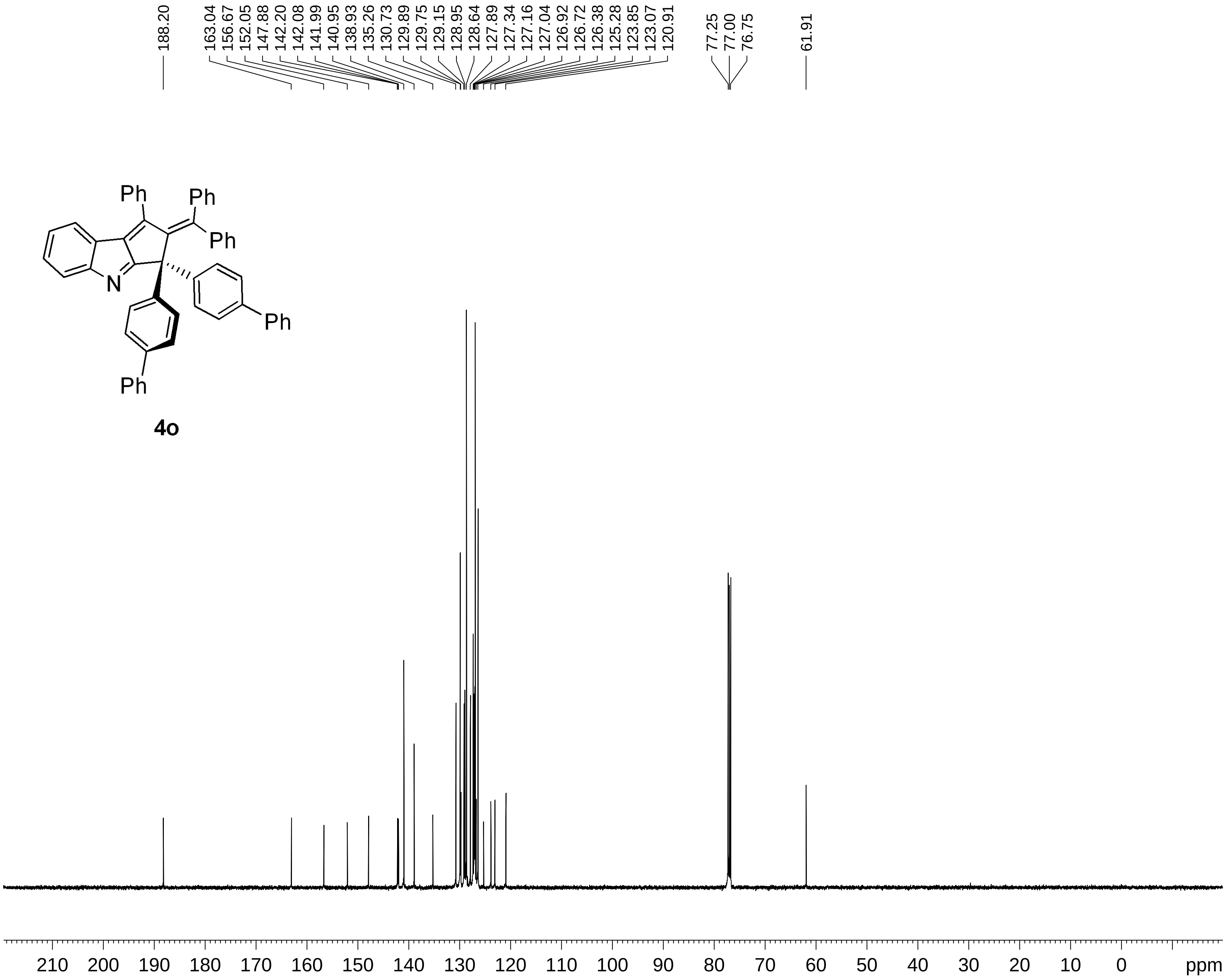


**4o**

| | |
|---|---|
| NAME | qzw_685_2 |
| EXPNO | 21 |
| PROCNO | 1 |
| Date_ | 20210621 |
| Time | 19.40 h |
| INSTRUM | Avance NEO 500 |
| PROBHD | Z119470_0332 ( |
| PULPROG | zgpg30 |
| TD | 65536 |
| SOLVENT | CDCl3 |
| NS | 400 |
| DS | 4 |
| SWH | 30120.482 Hz |
| FIDRES | 0.919204 Hz |
| AQ | 1.0879476 sec |
| RG | 101 |
| DW | 16.600 usec |
| DE | 6.50 usec |
| TE | 296.2 K |
| D1 | 2.00000000 sec |
| D11 | 0.03000000 sec |
| TD0 | 1 |
| SFO1 | 125.7753938 MHz |
| NUC1 | 13C |
| P0 | 3.33 usec |
| P1 | 10.00 usec |
| SI | 32768 |
| SF | 125.7628366 MHz |
| WDW | EM |
| SSB | 0 |
| LB | 1.00 Hz |
| GB | 0 |
| PC | 1.40 |

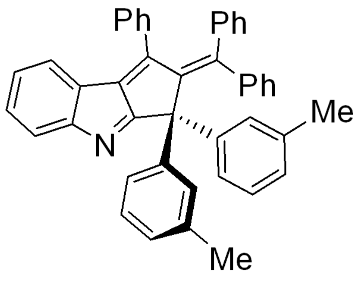


**4p**

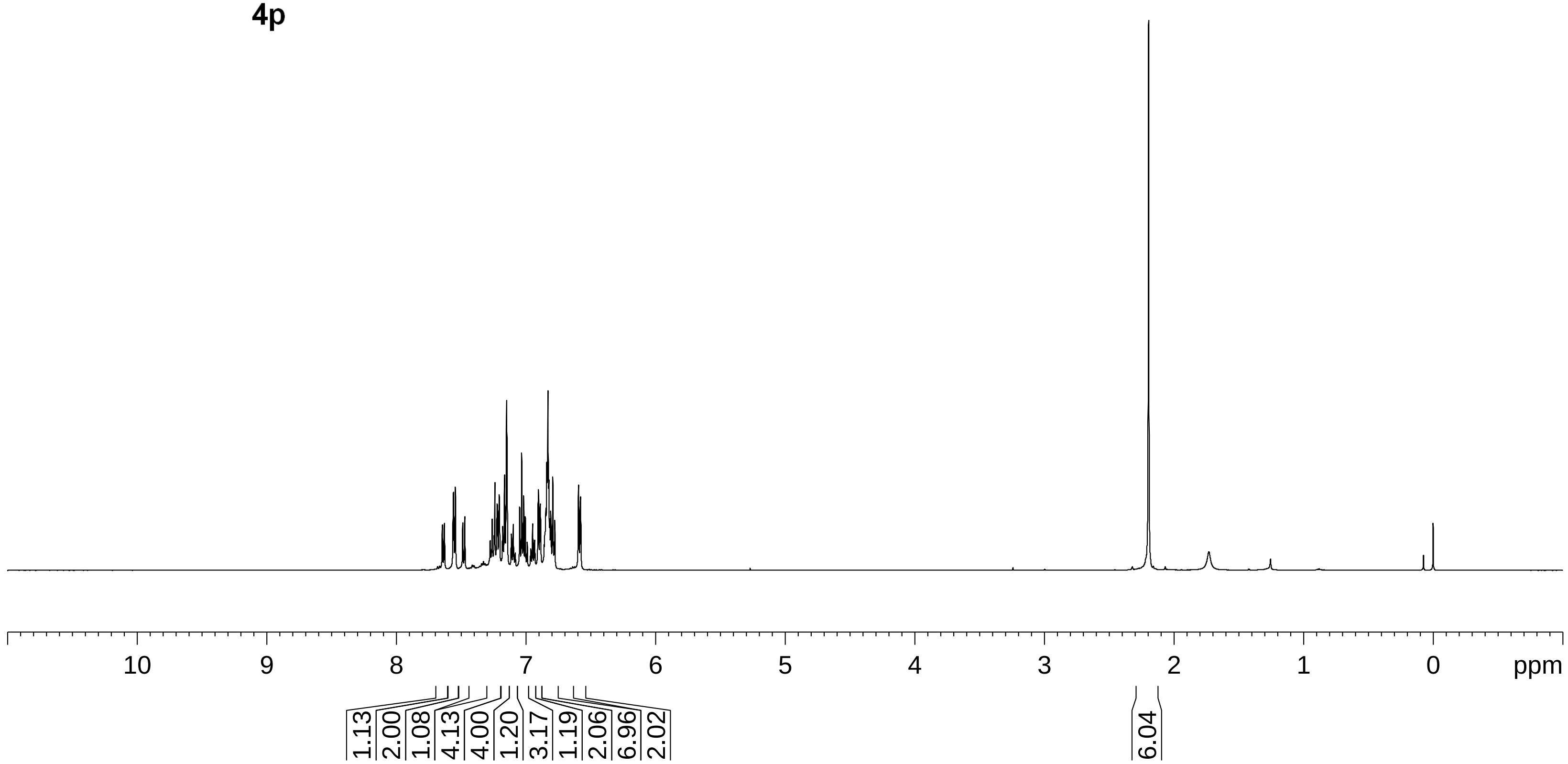


| | |
|---|---|
| NAME | qzw_685_4 |
| EXPNO | 20 |
| PROCNO | 1 |
| Date_ | 20210621 |
| Time | 18.41 h |
| INSTRUM | Avance NEO 500 |
| PROBHD | Z119470_0332 ( |
| PULPROG | zg30 |
| TD | 65536 |
| SOLVENT | CDCl3 |
| NS | 8 |
| DS | 2 |
| SWH | 10000.000 Hz |
| FIDRES | 0.305176 Hz |
| AQ | 3.2768500 sec |
| RG | 101 |
| DW | 50.000 usec |
| DE | 10.84 usec |
| TE | 296.1 K |
| D1 | 1.00000000 sec |
| TD0 | 1 |
| SFO1 | 500.1530884 MHz |
| NUC1 | 1H |
| P0 | 3.24 usec |
| P1 | 9.72 usec |
| SI | 65536 |
| SF | 500.1500222 MHz |
| WDW | EM |
| SSB | 0 |
| LB | 0.30 Hz |
| GB | 0 |
| PC | 1.00 |

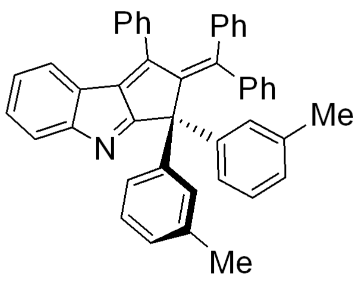


**4p**

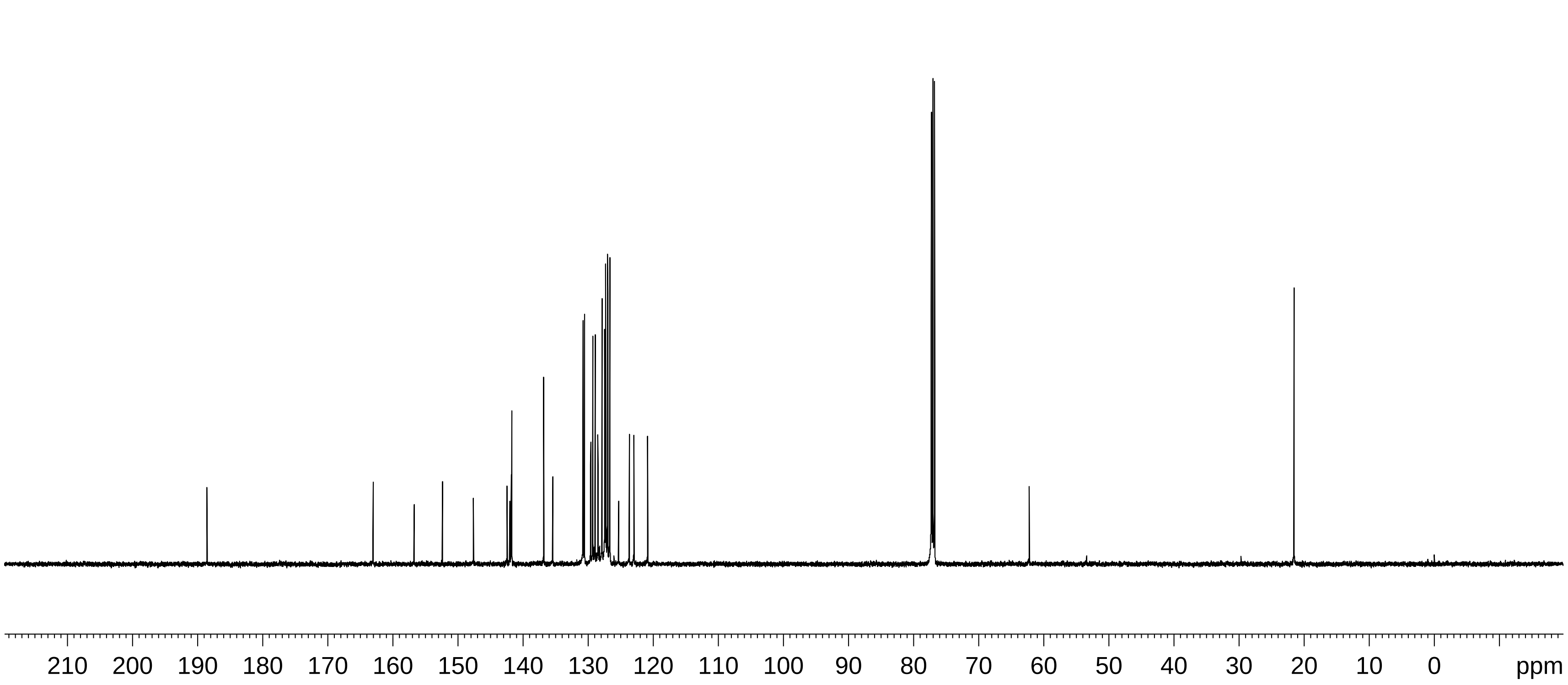


| | |
|---|---|
| NAME | qzw_685_4 |
| EXPNO | 11 |
| PROCNO | 1 |
| Date_ | 20210621 |
| Time | 5.59 h |
| INSTRUM | Avance NEO 500 |
| PROBHD | Z119470_0332 ( |
| PULPROG | zgpg30 |
| TD | 65536 |
| SOLVENT | CDCl3 |
| NS | 600 |
| DS | 4 |
| SWH | 30120.482 Hz |
| FIDRES | 0.919204 Hz |
| AQ | 1.0879476 sec |
| RG | 101 |
| DW | 16.600 usec |
| DE | 6.50 usec |
| TE | 296.2 K |
| D1 | 2.00000000 sec |
| D11 | 0.03000000 sec |
| TD0 | 1 |
| SFO1 | 125.7753938 MHz |
| NUC1 | 13C |
| P0 | 3.33 usec |
| P1 | 10.00 usec |
| SI | 32768 |
| SF | 125.7628284 MHz |
| WDW | EM |
| SSB | 0 |
| LB | 1.00 Hz |
| GB | 0 |
| PC | 1.40 |

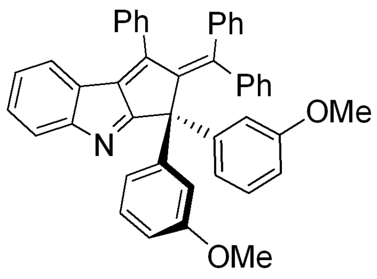


**4q**

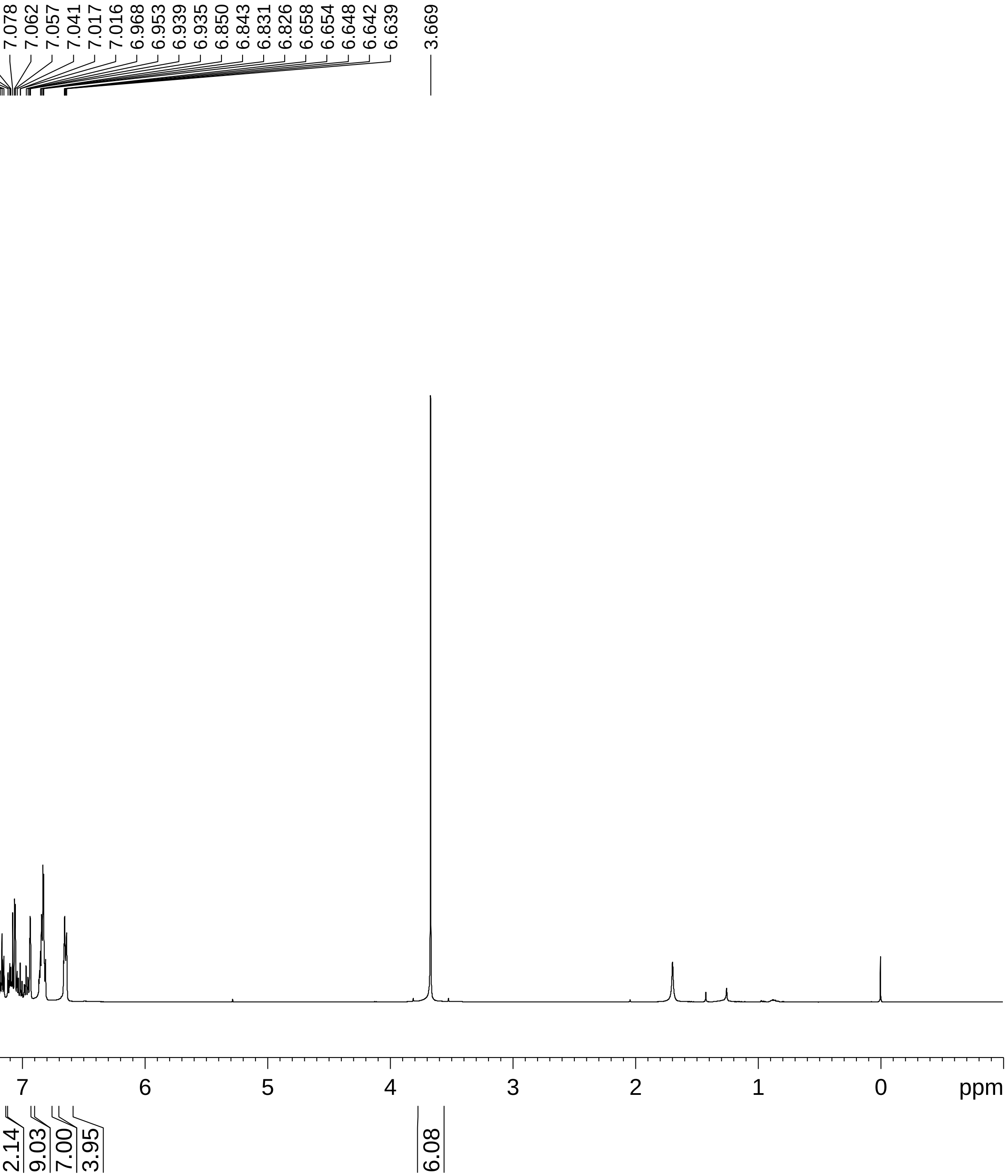


NAME qzw_684_5
EXPNO 10
PROCNO 1
Date_ 20210620
Time 6.19 h
INSTRUM Avance NEO 500
PROBHD Z119470_0332 (
PULPROG zg30
TD 65536
SOLVENT CDCl3
NS 16
DS 2
SWH 10000.000 Hz
FIDRES 0.305176 Hz
AQ 3.2768500 sec
RG 101
DW 50.000 usec
DE 10.84 usec
TE 296.2 K
D1 1.00000000 sec
TD0 1
SFO1 500.1530884 MHz
NUC1 1H
P0 3.24 usec
P1 9.72 usec
SI 65536
SF 500.1500168 MHz
WDW EM
SSB 0
LB 0.30 Hz
GB 0
PC 1.00

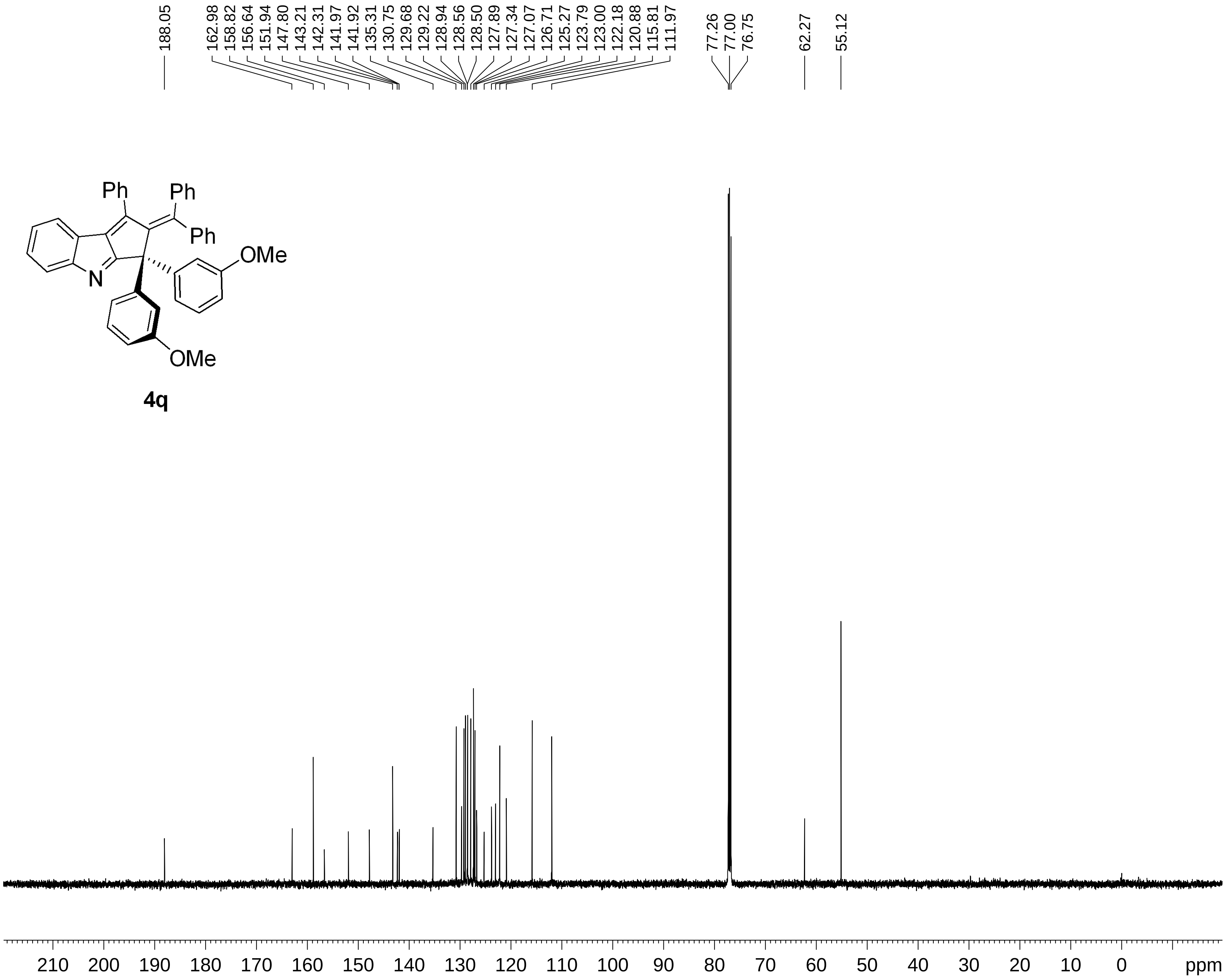


**4q**

| | |
|---|---|
| NAME | qzw_684_5 |
| EXPNO | 11 |
| PROCNO | 1 |
| Date_ | 20210620 |
| Time | 6.37 h |
| INSTRUM | Avance NEO 500 |
| PROBHD | Z119470_0332 ( |
| PULPROG | zgpg30 |
| TD | 65536 |
| SOLVENT | CDCl3 |
| NS | 320 |
| DS | 4 |
| SWH | 30120.482 Hz |
| FIDRES | 0.919204 Hz |
| AQ | 1.0879476 sec |
| RG | 101 |
| DW | 16.600 usec |
| DE | 6.50 usec |
| TE | 296.2 K |
| D1 | 2.00000000 sec |
| D11 | 0.03000000 sec |
| TD0 | 1 |
| SFO1 | 125.7753938 MHz |
| NUC1 | 13C |
| P0 | 3.33 usec |
| P1 | 10.00 usec |
| SI | 32768 |
| SF | 125.7628242 MHz |
| WDW | EM |
| SSB | 0 |
| LB | 1.00 Hz |
| GB | 0 |
| PC | 1.40 |

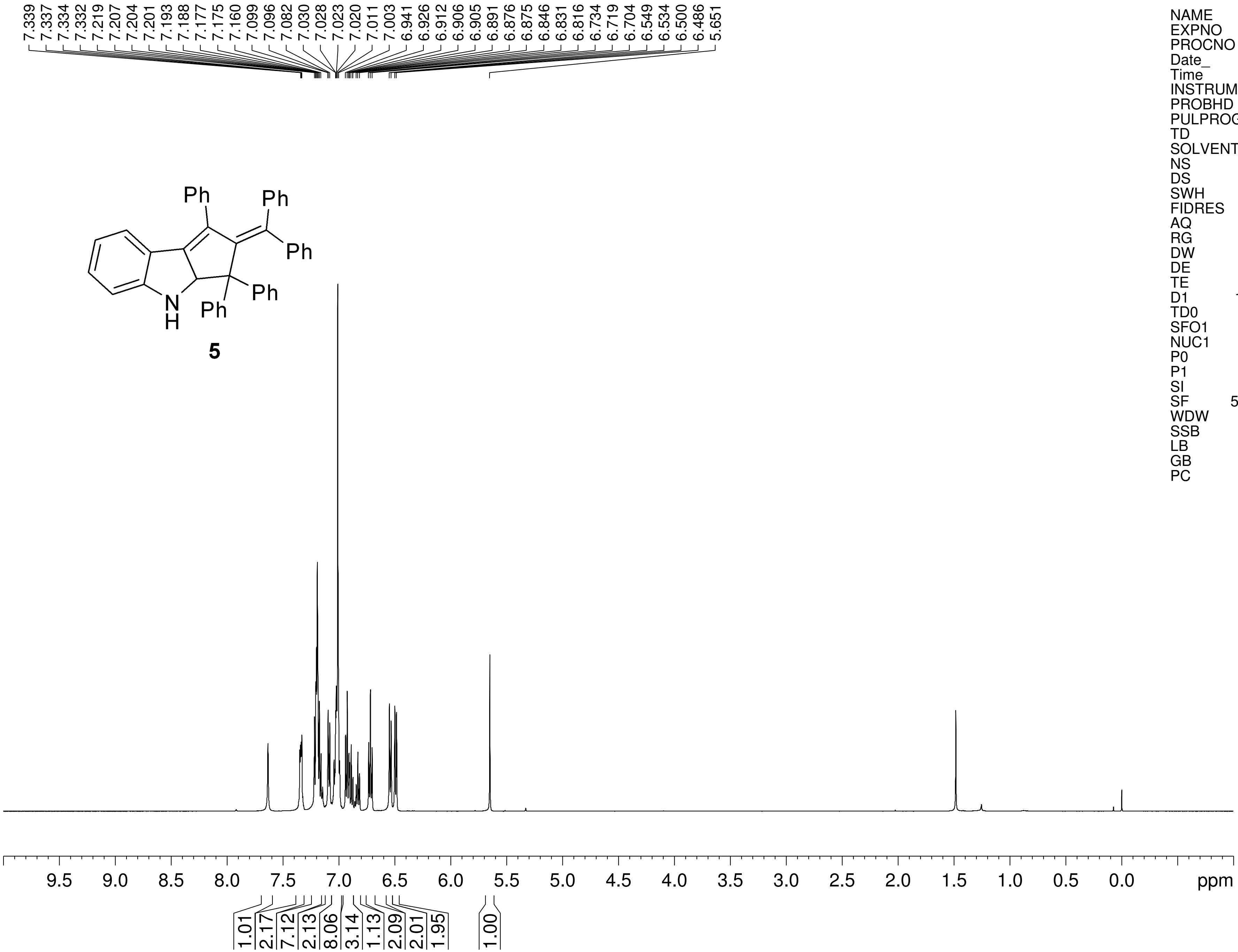


| | |
|---|---|
| NAME | m_qzw_721 |
| EXPNO | 10 |
| PROCNO | 1 |
| Date_ | 20240217 |
| Time | 22.09 h |
| INSTRUM | Avance NEO 500 |
| PROBHD | Z119470_0332 ( |
| PULPROG | zg30 |
| TD | 65536 |
| SOLVENT | CDCl3 |
| NS | 8 |
| DS | 2 |
| SWH | 10000.000 Hz |
| FIDRES | 0.305176 Hz |
| AQ | 3.2768500 sec |
| RG | 101 |
| DW | 50.000 usec |
| DE | 10.84 usec |
| TE | 294.9 K |
| D1 | 1.00000000 sec |
| TD0 | 1 |
| SFO1 | 500.1530884 MHz |
| NUC1 | 1H |
| P0 | 3.24 usec |
| P1 | 9.72 usec |
| SI | 65536 |
| SF | 500.1500330 MHz |
| WDW | EM |
| SSB | 0 |
| LB | 0.30 Hz |
| GB | 0 |
| PC | 1.00 |

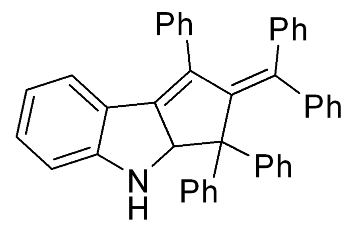


**5**

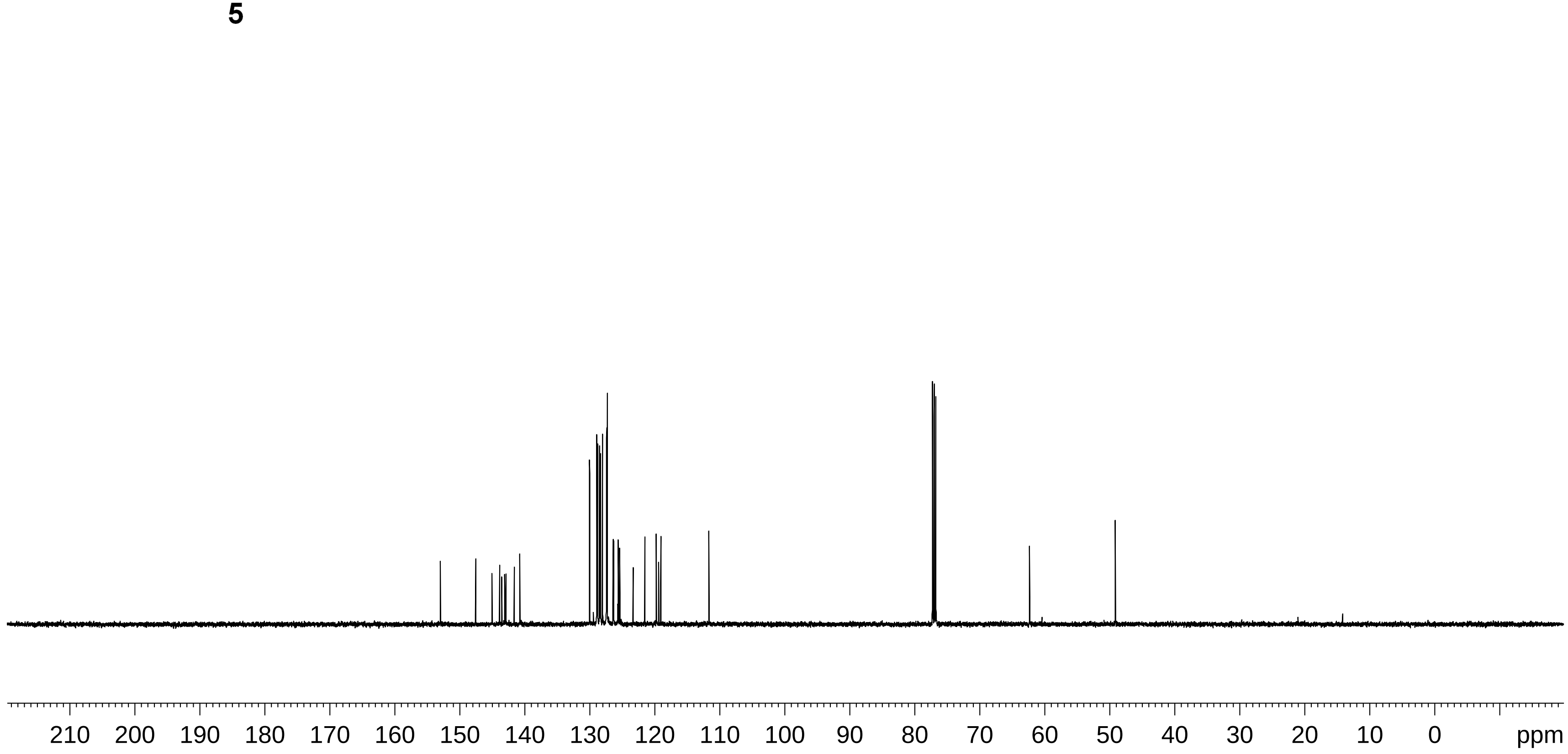


| | |
|---|---|
| NAME | qzw_721 |
| EXPNO | 11 |
| PROCNO | 1 |
| Date_ | 20210710 |
| Time | 6.43 h |
| INSTRUM | Avance NEO 500 |
| PROBHD | Z119470_0332 ( |
| PULPROG | zgpg30 |
| TD | 65536 |
| SOLVENT | CDCl3 |
| NS | 100 |
| DS | 4 |
| SWH | 30120.482 Hz |
| FIDRES | 0.919204 Hz |
| AQ | 1.0879476 sec |
| RG | 101 |
| DW | 16.600 usec |
| DE | 6.50 usec |
| TE | 296.1 K |
| D1 | 2.00000000 sec |
| D11 | 0.03000000 sec |
| TD0 | 1 |
| SFO1 | 125.7753938 MHz |
| NUC1 | 13C |
| P0 | 3.33 usec |
| P1 | 10.00 usec |
| SI | 32768 |
| SF | 125.7628325 MHz |
| WDW | EM |
| SSB | 0 |
| LB | 1.00 Hz |
| GB | 0 |
| PC | 1.40 |

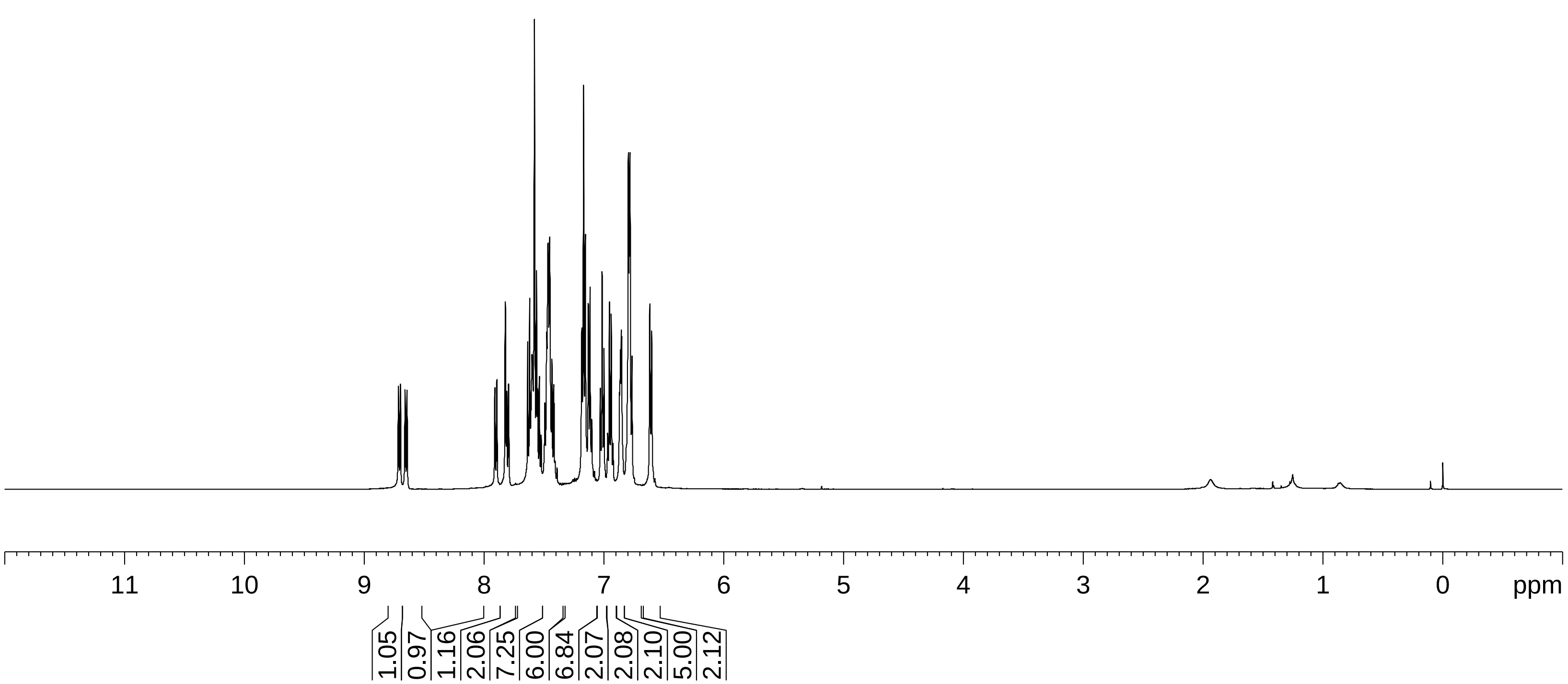


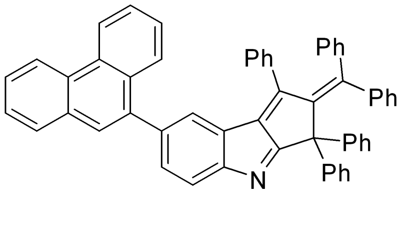


**6**

| | |
|---|---|
| NAME | qzw_751 |
| EXPNO | 10 |
| PROCNO | 1 |
| Date_ | 20210724 |
| Time | 20.05 h |
| INSTRUM | Avance NEO 500 |
| PROBHD | Z119470_0332 ( |
| PULPROG | zg30 |
| TD | 65536 |
| SOLVENT | CDCl3 |
| NS | 4 |
| DS | 2 |
| SWH | 10000.000 Hz |
| FIDRES | 0.305176 Hz |
| AQ | 3.2768500 sec |
| RG | 45.2174 |
| DW | 50.000 usec |
| DE | 10.84 usec |
| TE | 296.2 K |
| D1 | 1.00000000 sec |
| TD0 | 1 |
| SFO1 | 500.1530884 MHz |
| NUC1 | 1H |
| P0 | 3.24 usec |
| P1 | 9.72 usec |
| SI | 65536 |
| SF | 500.1500593 MHz |
| WDW | EM |
| SSB | 0 |
| LB | 0.30 Hz |
| GB | 0 |
| PC | 1.00 |

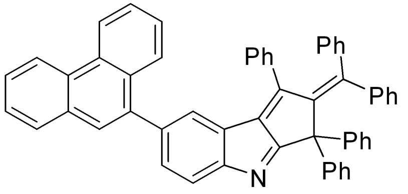

**6**

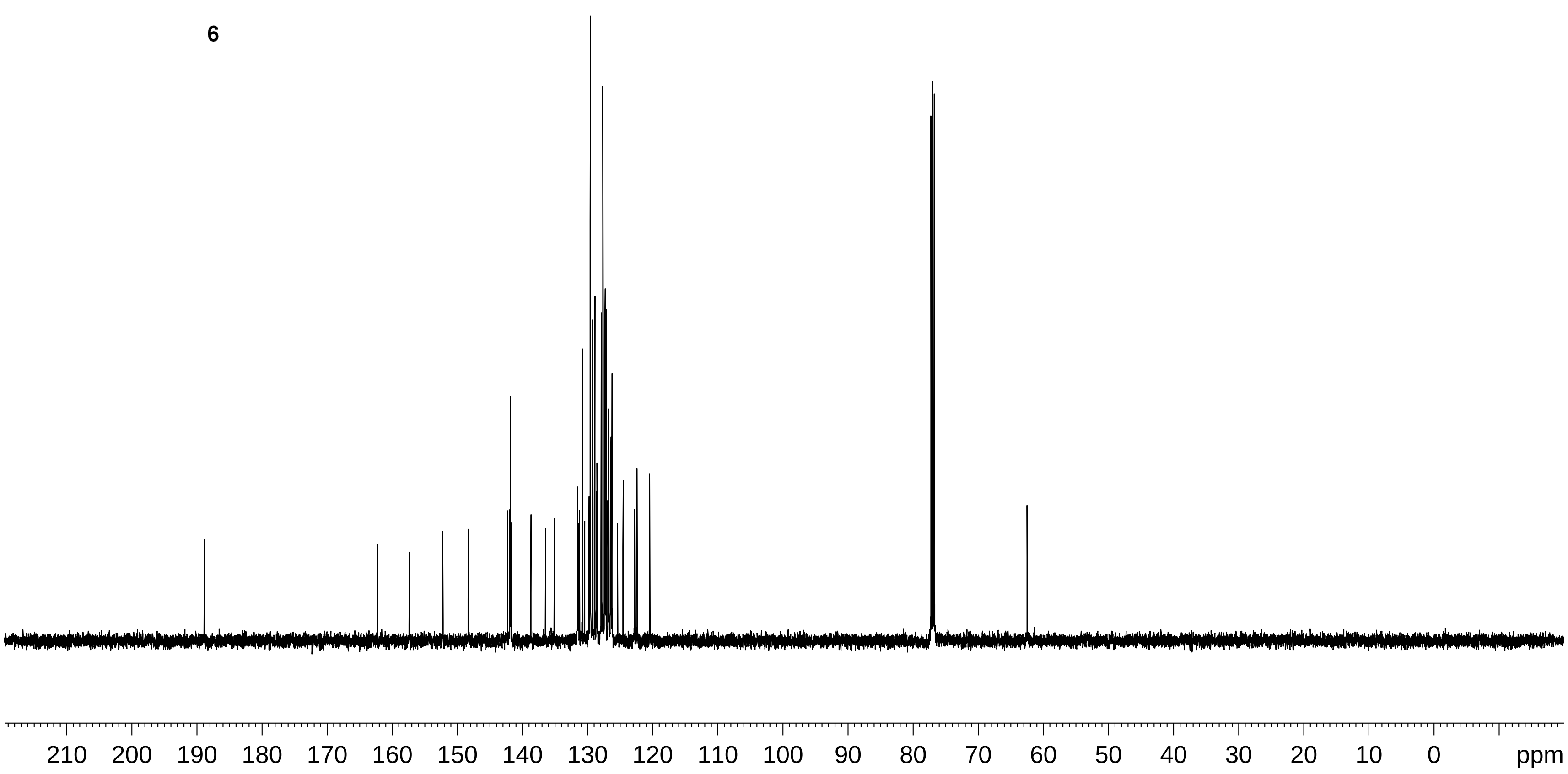


| | |
|---|---|
| NAME | qzw_751 |
| EXPNO | 11 |
| PROCNO | 1 |
| Date_ | 20210724 |
| Time | 20.08 h |
| INSTRUM | Avance NEO 500 |
| PROBHD | Z119470_0332 ( |
| PULPROG | zgpg30 |
| TD | 65536 |
| SOLVENT | CDCl3 |
| NS | 40 |
| DS | 4 |
| SWH | 30120.482 Hz |
| FIDRES | 0.919204 Hz |
| AQ | 1.0879476 sec |
| RG | 101 |
| DW | 16.600 usec |
| DE | 6.50 usec |
| TE | 296.1 K |
| D1 | 2.00000000 sec |
| D11 | 0.03000000 sec |
| TD0 | 1 |
| SFO1 | 125.7753938 MHz |
| NUC1 | 13C |
| P0 | 3.33 usec |
| P1 | 10.00 usec |
| SI | 32768 |
| SF | 125.7628426 MHz |
| WDW | EM |
| SSB | 0 |
| LB | 1.00 Hz |
| GB | 0 |
| PC | 1.40 |
